# δ-Bonding modulates the electronic structure of formally divalent nd^1^ rare earth arene complexes†

**DOI:** 10.1039/d4sc03005b

**Published:** 2024-08-05

**Authors:** Ross E. MacKenzie, Tomáš Hajdu, John A. Seed, George F. S. Whitehead, Ralph W. Adams, Nicholas F. Chilton, David Collison, Eric J. L. McInnes, Conrad A. P. Goodwin

**Affiliations:** a Centre for Radiochemistry Research, The University of Manchester Oxford Road Manchester M13 9PL UK; b Department of Chemistry, The University of Manchester Oxford Road Manchester M13 9PL UK; c Research School of Chemistry, The Australian National University Sullivans Creek Road Canberra 2601 Australia; d Photon Science Institute, The University of Manchester Oxford Road Manchester M13 9PL UK

## Abstract

Landmark advances in rare earth (RE) chemistry have shown that divalent complexes can be isolated with non-Aufbau 4f^*n*^{5d/6s}^1^ electron configurations, facilitating remarkable bonding motifs and magnetic properties. We report a series of divalent bis-tethered arene complexes, [RE(NHAr^iPr_6_^)_2_] (2RE; RE = Sc, Y, La, Sm, Eu, Tm, Yb; NHAr^iPr_6_^ = {N(H)C_6_H_3_-2,6-(C_6_H_2_-2,4,6-*i*Pr_3_)_2_}). Fluid solution EPR spectroscopy gives *g*_iso_ < 2.002 for 2Sc, 2Y, and 2La, consistent with formal nd^1^ configurations, calculations reveal metal–arene δ-bonding *via* mixing of nd_(*x*^2^−*y*^2^)_ valence electrons into arene π* orbitals. Experimental and calculated EPR and UV-Vis-NIR spectroscopic properties for 2Y show that minor structural changes markedly alter the metal d_(*x*^2^−*y*^2^)_ contribution to the SOMO. This contrasts 4f^*n*^{5d/6s}^1^ complexes where the valence d-based electron resides in a non-bonding orbital. Complexes 2Sm, 2Eu, 2Tm, and 2Yb contain highly-localised 4f^*n*+1^ ions with no appreciable metal–arene bonding by density functional calculations. These results show that the physicochemical properties of divalent rare earth arene complexes with both formal nd^1^ and 4f^*n*+1^ configurations are nuanced, may be controlled through ligand modification, and require a multi-pronged experimental and theoretical approach to fully rationalise.

## Abstract 2

Metal–arene contacts in divalent rare earth complexes can give rise formal 4f^*n*^ d(_*x*^2^−*y*^2^_) valence electron configurations where metal–arene δ-bonding modulates the electronic structure. Rare earth ions without d-contribution (*i.e.* f^*n*+1^) are unaffected.

## Graphical abstract

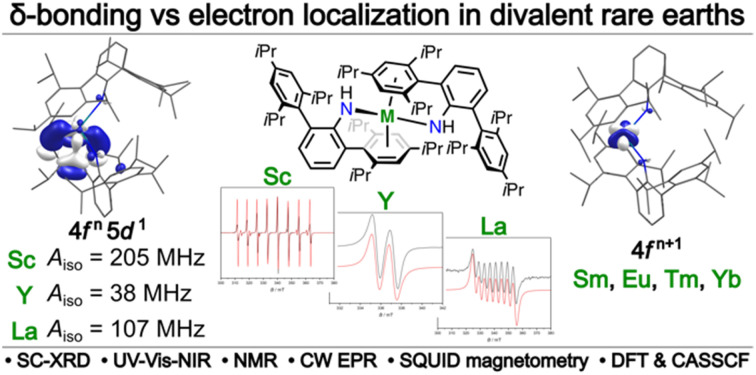

## Introduction

A hallmark of trivalent lanthanide ions is the relative insensitivity of valence 4f-orbitals to the coordination environment. While this gives rise to useful physical and optical properties, such as their narrow optical emission profiles and large magnetic moments from their unquenched orbital angular momentum, the weakness of this interaction also inhibits the extent to which their properties may be tailored through molecular design. Advancements in divalent molecular rare earth and lanthanide (Sc, Y, La–Lu; collectively Ln henceforth) chemistry have shown that in coordination environments such as [Ln(Cp^R^)_3_]^−^ or [Ln(Cp^R^)_2_] (Cp^R^ = substituted cyclopentadienide ligands), Ln(ii) complexes can be isolated with 4f^*n*^5d^1^ or 4f^*n*^{5d/6s}^1^ valence electron configurations, at least for La–Lu, while Sc and Y necessarily give 3d^1^ or 4d^1^ ions.^1–23^ Further work shows these non-Aufbau ions can be exploited as potential qubit candidates,^15–17^ exhibit record-setting magnetic properties,^18–20^ and have produced the first examples of molecular Ln–Ln bonding outside of endohedral fullerenes.^20–22^ Similar advancements have also been made with actinide elements.^24^ However, with few exceptions, design strategies employed to isolate examples of these ions use geometries (*e.g. C*_3h_ or *D*_5h_) that minimise or forbid, by symmetry, the mixing of 5d (or 5d/6s hybridised) valence electrons with ligand orbitals. This is useful in some applications,^15–17^ but in analogy to the poor tunability of 4f-orbitals in Ln(iii) ions, it limits the extent to which the electronic structures of 4f^*n*^5d^1^ or 4f^*n*^{5d/6s}^1^ Ln(ii) complexes may be controlled through ligand design.

Using ligand field principles commonplace in the d-block that also apply to 4f^*n*^5d^1^ Ln(ii) ions, we may instead target molecular designs where the electronic structure and physicochemical properties are more sensitive to changes in the coordination environment. Arene ligands provide a promising route towards these goals due to their ability to act as symmetry-allowed donors into vacant d-orbitals, and further stabilise lower oxidation states through back donation.^25–32^ Indeed, the only examples of formally zero-valent rare earth complexes are within [M(η^6^-C_6_R_6_)_2_] frameworks.^33–37^

Herein, we present a series of structurally analogous bis-tethered arene divalent rare earth complexes of the form [M(NHAr^iPr_6_^)_2_] (2M, M = Sc, Y, La, Sm, Eu, Tm, Yb; NHAr^iPr_6_^ = {N(H)C_6_H_3_-2,6-(C_6_H_2_-2,4,6-*i*Pr_3_)_2_}) – the synthesis and some properties of 2Y have been reported previously.^38^ In all cases, characterisation using SQUID magnetometry, solid and solution phase EPR spectroscopy, UV-Vis-NIR spectroscopy, density functional theory (DFT), and complete active space self-consistent field (CASSCF) calculations support the description of these as formal Ln(ii) complexes, demonstrating that this framework is robust across a range of Ln(ii) ion sizes and (formal) reduction potentials. While all seven divalent complexes display close metal–arene contacts, only 2Sc, 2Y, and 2La show mixing of a metal valence nd_(*x*^2^−*y*^2^)_ orbital with the arene π* to give δ-bonding interactions. Complexes 2Sm, 2Eu, 2Tm, and 2Yb instead adopt metal-localised 4f^*n*+1^ configurations in accordance with their large Ln(ii) 4f^*n*+1^ → 4f^*n*^5d^1^ promotional energies.^39,40^ Solid and solution phase UV-Vis-NIR and EPR for 2Y, combined with DFT calculations, reveals that the balance of metal *vs.* arene-centred spin-density is sensitive to small structural changes between these phases, suggesting the properties of divalent rare earth complexes with nd^1^ may be tuned through ligand design.

## Results and discussion

### Synthesis

**Scheme 1:**
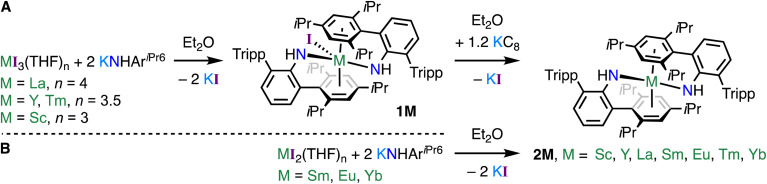
Synthesis of (Route A) [M(NHAr^iPr_6_^)_2_(I)] (1M, M = Sc, Y, La, Tm), and (Route B) [M(NHAr^iPr_6_^)_2_] (M = Sc, Y, La, Sm, Eu, Tm, Yb). Tripp = {C_6_H_2_-2,4,6-*i*Pr_3_}.

A reductive route was pursued for the synthesis of 2M (M = Sc, Y, La, and Tm; Scheme 1A), which began with the synthesis of trivalent [M(NHAr^iPr_6_^)_2_(I)] (1M, M = Sc, Y, La, Tm) precursors. Complexes 1M were synthesised by salt elimination between KNHAr^iPr_6_^ (ref. 41 and 42) and the relevant rare earth tri-iodide salt [MI_3_(THF)_*n*_] (*n* = 4, M = La; *n* = 3.5, M = Y, Tm; *n* = 3, M = Sc) in Et_2_O.^43^ Workup and crystallisation from toluene gave fair to good crystalline yields (47–62%). ^1^H NMR spectroscopy of diamagnetic 1Sc, 1Y, and 1La in d_6_-benzene show the M⋯C_6-arene_ interaction is dynamic in solution and that all four Tripp groups are in exchange (see ESI† for detailed assignments).^38^

Reduction (KC_8_, 1.2 equiv.) of 1M (M = Sc, Y, La, Tm) in Et_2_O gave, after workup and crystallization from Et_2_O, dark crystals of 2M in fair crystalline yields (shown as % yield ahead): 2Sc (red, 53%), 2Y (red/green, 55%), 2La (red/brown, 45%), and 2Tm (red/brown, 65%). Salt elimination between KNHAr^iPr_6_^ and MI_2_(THF)_2_ (M = Sm, Eu, Yb)^44^ in Et_2_O gave poor to excellent isolated crystalline yields of 2Sm (69%), 2Eu (20%), and 2Yb (82%) (Scheme 1B). Attempts to synthesise 1Sm and 1Yb using [MI_3_(THF)_*n*_] precursors were unsuccessful, leading to intractable mixtures (see ESI†), and we did not attempt the synthesis of 1Eu.

While the ^1^H NMR spectra of 1Sc, 1Y, and 1La showed the metal-bound and “terminal” {C_6_H_2_-2,4,6-*i*Pr_3_} (Tripp) groups were in exchange at room temperature, the spectrum of diamagnetic 2Yb in d_6_-benzene shows six doublets for the CH_3_-*i*Pr groups, along with a single N(H) resonance. Thus, 2Yb is *C*_2_ symmetric in solution and the Tripp groups do not appreciably exchange at room temperature. This does not appear to be due to steric hindrance as the Sc(iii) in 1Sc is smaller than Yb(ii) (6-coordinate radii: Yb(ii), 0.868 Å; Sc(iii), 0.745 Å). A variable temperature ^1^H NMR study in d_8_-toluene shows the CH_3_-*i*Pr peaks begin to coalesce at 308 K, but full equilibrium is not reached until *ca*. 358 K (Fig. S40 and S41†). The ^171^Yb–^1^H HMBC NMR spectrum gave two cross-peaks at *δ*_^171^Yb_ = −83 ppm (*δ*_^1^H_ = 3.53 and 7.02 ppm), showing the ^171^Yb couples to both the anilido proton and the Tripp 3,5-CH groups. The ^1^H NMR spectra of paramagnetic 2M complexes were uninformative, see the ESI† for all NMR spectra.

### Molecular structures

**Fig. 1:**
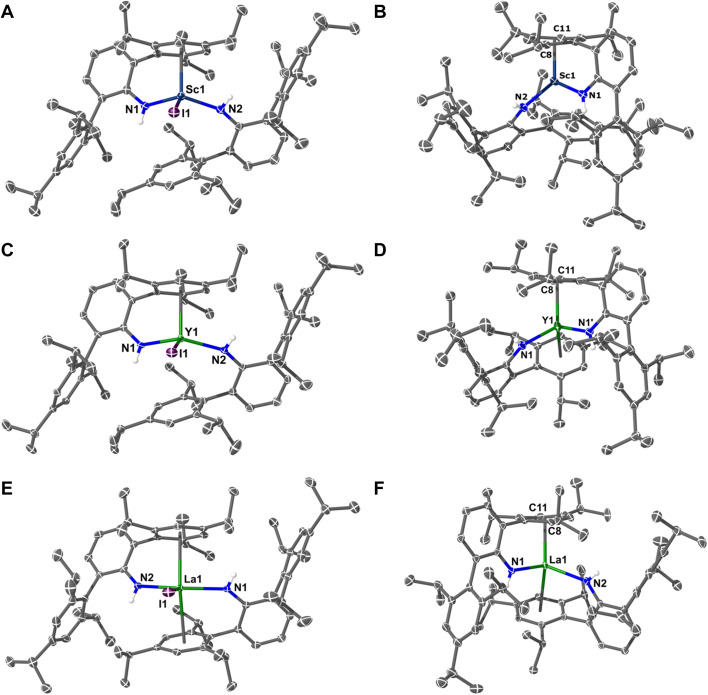
Molecular structures: (A) 1Sc; (B) 2Sc; (C) 1Y; (D) 2Y; (E) 1La; (F) 2La. Thermal ellipsoids have been set at 40% probability. H-atoms except those on N–H groups, solvents of crystallization, and disordered components have been removed for clarity.

Single crystal X-ray diffraction studies on 1M (M = Sc, Y, La, Tm) show all crystallize in the triclinic space group *P*1̄ (*Z*′ = 1) and are pseudo three-coordinate – see Fig. 1. In 1La, the metal is sandwiched almost equally between two Tripp groups (La⋯C_6-centroid_ = 2.8714(12) and 2.8783(12) Å; La⋯N_2_I plane deviation = 0.0042(15) Å). Conversely, 1Sc and 1Y have a single Tripp group close to the metal (M⋯C_6-centroid_: 1Sc, 2.3391(8) Å; and 1Y, 2.4949(9) Å) and hence are trigonal pyramidal. The structure of 1Y is comparable to recently reported [Y(NHAr^iPr_6_^)_2_(Cl)].^38^ See the ESI† for the structure of 1Tm, and a comparison of 1M complexes. In the following sections, we shall continue to use the term “C_6-centroid_”, noting it is not strictly appropriate to define a centroid for non-planar groups.

The molecular structures of 2Sc, 2Y, and 2La are also shown in Fig. 1 (see ESI† for 2Sm, 2Eu, 2Tm, and 2Yb). With the exception of 2Sc, all 2M complexes (M = Y, La, Sm, Eu, Tm, Yb) show two Tripp groups closely approaching the metal. When viewed along the C_6-centroid_⋯C_6-centroid_ axis these groups are either fully eclipsed due to crystallographic *C*_2_ symmetry (2Y, 2Eu, and 2Yb), or are pseudo-eclipsed (2Sc, 2La, 2Sm, and 2Tm). In 2Sc, only one Tripp group is close to the metal. The nature of this metal–arene interaction provides insight into the electronic structure *vis-à-vis* metal- or ligand-centred reduction.^45,46^ Complexes 2Sc and 2La crystallize with a whole molecule in the asymmetric unit, and in both one metal-bound Tripp group is non-planar, showing an “open book” deformation for which a “hinge angle” (∠_arene_) can be calculated – 11.43(11)° for 2Sc, and 12.9(3)° for 2La. In 2Sc, the next shortest M⋯C_6-centroid_ distance (3.8304(7) Å) is too long to constitute a strong interaction; but, in 2La the equivalent group is only *ca*. 0.4 Å further (M⋯C_6-centroid_ = 2.8348(12) Å) than the deformed arene ring, but is planar. Complex 2Sc is similar to the Ti(iv) analogue 2Ti,^46^ but the latter exhibits ∠_arene_ of 24.19(18)° and is diamagnetic by SQUID magnetometry, indicating the presence of a dianionic Tripp ring.

Complex 2Y is different to 2Sc and 2La as only half the molecule is present in the asymmetric unit (*Z*′ = 0.5), as previously reported;^38^ and also to that of 2U.^47^ The ∠_arene_ angle for both ligands in 2Y is 7.27(12)°, which compares well to 9.5(1)° in 2U.^47^ There is no clear trend in ∠_arene_ values (2Y < 2U < 2La < 2Sc) except that *C*_2_ symmetric complexes (2Y and 2U) have smaller ∠_arene_ angles,^38,47^ and that larger values (with a single deformed arene) correlate with a greater degree of metal electron localisation (see below). The Gd(0) complex, [Gd(C_6_H_3_-1,3,5-*t*Bu)_2_], also displays two symmetry-equivalent distorted arene rings (∠_arene_ = 3.1(3)°).^33^ The remaining divalent complexes, 2Sm, 2Eu, 2Tm, and 2Yb, are analogous to 2La, except only the latter shows arene deformation. Table 1 summarizes all 2M complexes now reported (M = Sc, Y, La, Sm, Eu, Tm, Yb, U).^38,47^

**Table 1:** Bond lengths (Å) and angles (°) for [M(NHAr^iPr_6_^)_2_] (2M, M = Sc, Y, La, Sm, Eu, Tm, Yb, U)^38,47^

(Å or °)		2Sc	2Y^a^	2La	2Sm	2Eu^b^	2Tm	2Yb^b^	2U
M–N	N(1)	2.0884(11)	2.2600(12)	2.395(3)	2.412(2)	2.411(4)	2.3060(17)	2.310(6)	2.330(2)
						2.414(5)^b^		2.294(6)^b^	
	N(2)	2.0678(10)	—a	2.434(3)	2.425(2)	—b	2.3169(18)	—b	—a
M–C_6-range_	Ring(1)	2.3913(12)–2.6304(14)	2.7276(14)–2.9273(15)	2.778(16)–2.971(9)	2.955(3)–3.160(3)	2.972(4)–3.176(5)	2.8015(19)–2.971(2)	2.840(6)–3.047(6)	2.723(3)–2.870(3)
	Ring(2)	—	—a	3.047(3)–3.240(3)	2.953(3)–3.201(3)	—b	2.819(2)–3.118(2)	—b	—a
M–C	C(8)	2.3913(12)	2.7684(14)	2.843(13)	—	—	—	—	2.731(3)
	C(11)	2.5418(13)	2.7859(15)	2.903(12)	—	—	—	—	2.723(3)
Arene fold angle		11.43(11)	7.27(12)	12.9(9)	—	—	—	—	9.3(2)

a The solid-state structure is *C*_2_-symmetric so there is a single metal and ligand per asymmetric unit.

b The solid-state structures show two half-molecules in the asymmetric unit, so M(1) and M(2) each have only one unique ligand.

### UV-Vis-NIR spectroscopy

**Fig. 2:**
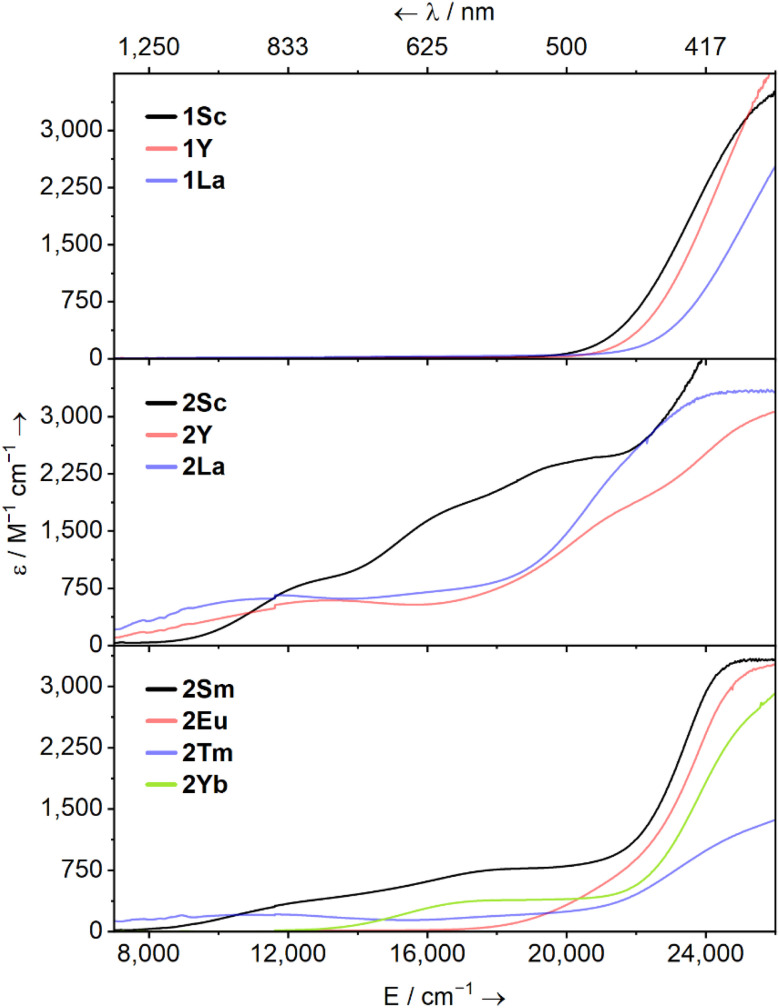
Solution UV-Vis-NIR spectra of [M(NHAr^iPr_6_^)(I)] (1M, M = Sc, Y, La; top) and [M(NHAr^iPr_6_^)] (2M, middle, M = Sc, Y, La; bottom, M = Sm, Eu, Tm, Yb) – as 1 mM solutions in Et_2_O between 7000–26 000 cm^−1^ (1429–385 nm) at ambient temperature.

UV-Vis-NIR spectra were collected for 1M (M = Sc, Y, La, Tm) and 2M (M = Sc, Y, La, Sm, Eu, Tm, Yb) at ambient temperature as 1 mM solutions in Et_2_O (Fig. 2, except for 1Tm). The spectra of 1Sc, 1Y, and 1La are uninformative, showing only a broad ligand-to-metal charge transfer (LMCT) process from *ca*. 20 000 cm^−1^ (500 nm) to well into the UV region, accounting for the intense yellow colour of all three in solution. The colours and spectra of 1Tm and 2Sm, 2Eu, 2Tm, and 2Yb are typical for these elements in their respective oxidation states (see ESI†).^13,14,48–51^

The spectrum of 2Sc shows three well-resolved absorptions at 13 400 cm^−1^ (756 nm, 805 M^−1^ cm^−1^), 17 400 cm^−1^ (575 nm, 1727 M^−1^ cm^−1^), and 21 300 cm^−1^ (469 nm, 2471 M^−1^ cm^−1^). The modest intensity of these peaks coupled with their energies suggests 3d → 3d transitions and/or metal-to-ligand charge transfer (MLCT) bands. For 2Y, two peaks are resolved at 13 700 cm^−1^ (729 nm, 594 M^−1^ cm^−1^) and 21 600 cm^−1^ (463 nm, 1787 M^−1^ cm^−1^), while a third tails in from above 26 000 cm^−1^ (385 nm), in agreement with the previous report.^38^ Finally, in 2La, a single clear peak is resolved at 12 300 cm^−1^ (816 nm, 650 M^−1^ cm^−1^), which we suggest is a 5d → 5d transition. A broad feature at *ca*. 16 000 cm^−1^ and a peak with a maximum at *ca*. 24 000 cm^−1^ (417 nm) can also be seen, but background absorption precludes accurately describing these.

### SQUID magnetometry

Direct current (DC) magnetic susceptibility data were collected for 1Tm, and 2M (M = Sc, Y, La, Sm, Eu, Tm) from 1.8 to 300 K under an applied field of 1 kOe. At 300 K, the *χ*_M_*T* (*χ*_M_ is the molar magnetic susceptibility) values for 1Tm (6.61 cm^3^ mol^−1^ K), 2Eu (7.27 cm^3^ mol^−1^ K), and 2Tm (2.47 cm^3^ mol^−1^ K) closely match theoretical values for 4f^*n*+1^ configurations for Tm(iii), 4f^12^ (^3^H_6_, 7.15 cm^3^ mol^−1^ K), Eu(ii), 4f^7^ (^8^S_7/2_, 7.88 cm^3^ mol^−1^ K), and Tm(ii), 4f^13^ (^2^F_7/2_, 2.57 cm^3^ mol^−1^ K). For 2Sm (4f^6^) the value at 300 K is 1.43 cm^3^ mol^−1^ K, which is in the range observed for Eu(iii) (4f^6^) complexes (1.3 to 1.5 cm^3^ mol^−1^ K),^52^ and is non-zero due to population of excited ^7^F_*J*_ states.^48–50^ Upon cooling to 1.8 K, *χ*_M_*T* for 2Sm lowers to 0.02 cm^3^ mol^−1^ K which is consistent with a ^7^F_0_ ground state; thus, a 4f^6^ configuration.

Complexes 2Sc, 2Y and 2La exhibit *χ*_M_*T* values at 300 K (0.31, 0.36, and 0.29 cm^3^ mol^−1^ K respectively), which are in reasonable agreement with the spin-only value for an *S* = 1/2 system (0.375 cm^3^ mol^−1^ K for *g* = 2.00), and hence with a formal d^1^ configuration. In each case, the magnetic moment is essentially invariant with temperature down to 8–10 K, where a sudden drop can be seen, though this varies across independently synthesised samples (see ESI† for more details).

### Electronic structure calculations

**Fig. 3:**
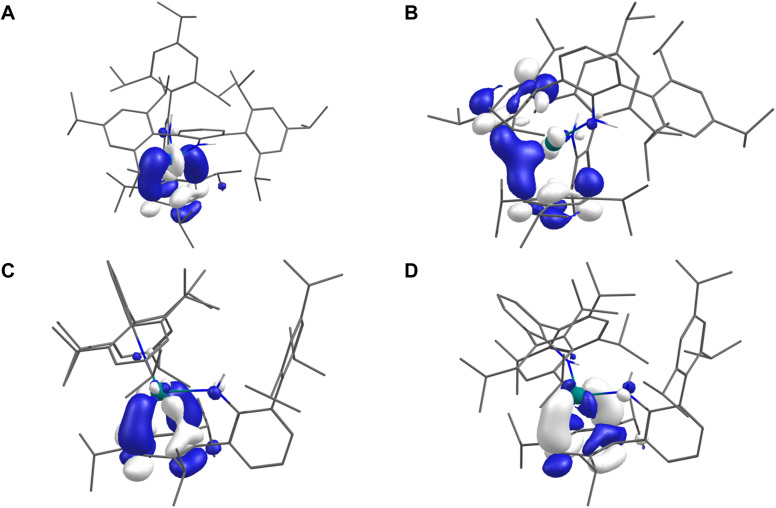
SOMOs of: (A) 2Sc; (B) 2Y; (C) 2La; (D) 2Y-Et_2_O (isovalues = 0.05) using geometries derived from single crystal X-ray diffraction with H-atoms optimised (2Sc, 2Y), select disordered C-atoms and all H-atoms optimised (2La), or all atom positions optimised (2Y-Et_2_O). H-atoms except those of the N(H) group omitted for clarity.

Unrestricted Kohn–Sham density-functional theory (DFT) calculations were performed on 2Sc, 2Y, 2La, 2Tm (*S* = 1/2), 2Sm (*S* = 3), 2Eu (*S* = 7/2), and 2Yb (*S* = 0) using partially geometry-optimised structures (see ESI† for full details). Löwdin population and spin analyses of 2Sm, 2Eu, 2Yb, and 2Tm are consistent with experimental data and describe all four as 4f^*n*+1^ Ln(ii) ions with metal-localised valence electrons. Fig. 3A and C shows the SOMOs of 2Sc and 2La, which depict M–arene δ-bonds comprised of 36% 3d (with 4% 4s) and 14% 5d (with 10% 4f and 1% 6s) metal character, respectively, with the bound Tripp ring making up 41% (2Sc) and 56% (2La) – the remainder is diffused over the rest of the molecule. Defining the M⋯Tripp direction as *z*, the d-orbital contribution is described as d_(*xy*/*x*^2^−*y*^2^)_ (the superficial resemblance to d_*x*^2^_ or d_*y*^2^_ is due to a small degree of d_*z*^2^_ mixing in this axis system) – this is comparable to the e_2g_ MO of bis-benzene transition metal complexes in *D*_6h_ symmetry.^53^

In the case of 2Y (Fig. 3B), the SOMO is delocalised across the symmetry equivalent metal-bound Tripp groups such that the SOMO composition is 14% Y (12% 4d, 0.5% 5s), while the bound Tripp rings sum to 64.6% – the SOMO resembles a delocalised δ-bonding interaction. These results agree with previous work on 2Y,^38^ but it is an outlier compared to 2Sc and 2La. Full geometry optimisation of 2Y using the lower-symmetry structure of 2La as the starting point was performed in the gas phase and using an Et_2_O solvent model (2Y-Et_2_O henceforth). Both calculations produced geometries that have only one metal-bound Tripp group deformed (*i.e.* like 2Sc and 2La) and are true local minima on the potential energy surface. Fig. 3D shows the SOMO of 2Y-Et_2_O, and Löwdin population analysis shows it to be more metal-localised (22% 4d, 2% 5s, and 1% 4f – total 25%) than in the *C*_2_-symmetric 2Y (14%), which is accompanied by a corresponding decrease in Tripp contributions to the SOMO – 65% in 2Y (over two Tripp groups) and 56% in 2Y-Et_2_O (over a single Tripp group). The Löwdin spin populations at the metal in 2Y-Et_2_O (0.245) and 2Y (0.142) reflect these differences.

**Fig. 4:**
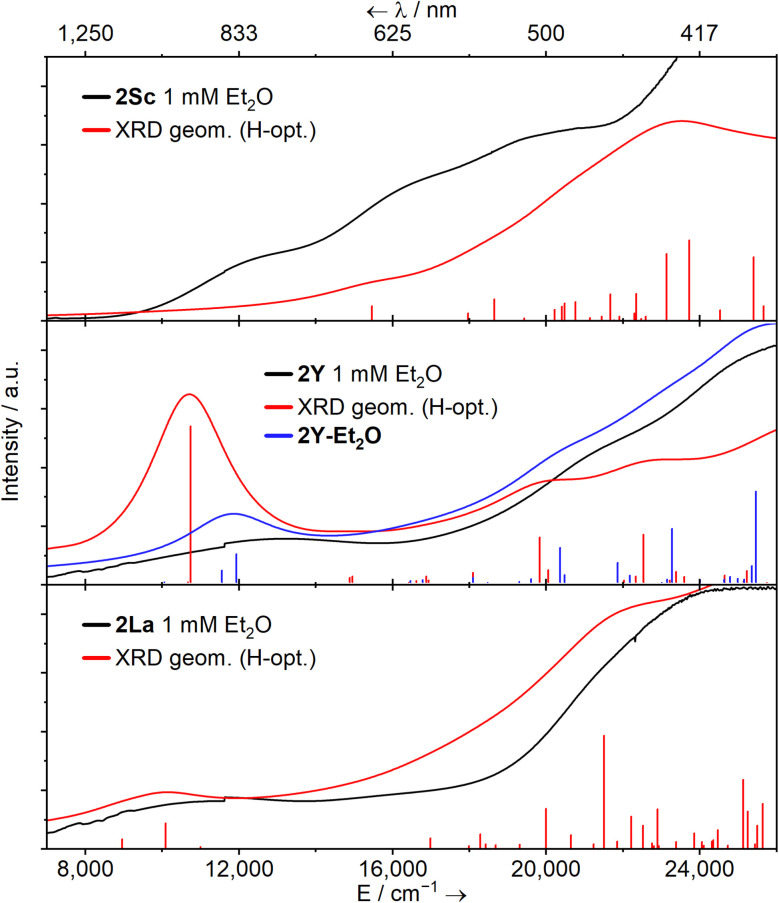
Experimental (1 mM, Et_2_O, black), calculated sTD-DFT transitions (red and blue vertical lines), and simulated spectra (red and blue solid lines, with Gaussian broadening and a linewidth factor of 
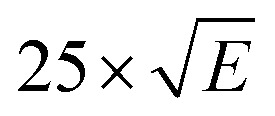
) UV-Vis-NIR spectra for complexes 2Sc, 2Y (and 2Y-Et_2_O) and 2La calculated transitions were performed using a solvent model accounting for the dielectric constant (*ε*) and refractive index (*n*_D_) of Et_2_O.

Time-dependent DFT (TD-DFT) and simplified TD-DFT (sTD-DFT) calculations were employed to model the UV-Vis-NIR spectra of 2Sc, 2Y, 2La, and 2Y-Et_2_O; here we focus on sTD-DFT with TPSSh for consistency with prior art (Fig. 4),^11,12,16,23,54–56^ see ESI (Fig. S102–S111†) for more details. Experimental features of 2Sc and 2La are well represented, and the Natural Transition Orbitals (NTOs) suggests the broad features in the spectrum of 2Sc are 3d → 3d transitions, the lowest energy of which resembles a 3d_(*xy*/*x*^2^−*y*^2^)_ → 3d_(*x*^2^−*y*^2^/*xy*)_ transition maintaining the δ-bonding interaction. For 2La, the lowest energy feature is a 5d_(*xy*/*x*^2^−*y*^2^)_ → 5d_(*x*^2^−*y*^2^/*xy*)_ transition, and the next two lowest energy features are a combination of MLCT and 5d → 5d transitions. There is poor agreement with all methods for 2Y (Fig. 4 middle panel, red line), however, calculations for 2Y-Et_2_O are substantially better (Fig. 4 middle panel, blue line); this suggests that the structure of 2Y in Et_2_O solution is similar to the solid-state structures of 2Sc and 2La. The lowest energy feature in 2Y-Et_2_O is comprised of two components, a 4d_(*xy*/*x*^2^−*y*^2^)_ → 4d_(*x*^2^−*y*^2^/*xy*)_ transition and an MLCT process.

Complete active space self-consistent field (CASSCF) calculations (see ESI†) on 2M confirm the DFT and experimental results: 2Sc, 2Y and 2La exhibit nd^1^ ground states with significant orbital mixing with the arene ligand(s), and 2Sm, 2Eu, 2Tm and 2Yb have 4f^*n*+1^ ground states. Calculations for the lowest-lying excitations in 2Sc, 2Y and 2La including multi-configurational pair-density functional theory (MC-PDFT) corrections for dynamic correlation show nd/arene → nd/arene excitations in the UV-Vis-NIR range, in good agreement with the experimental spectra. The character of these excitations is broadly in line with that found using (s)TD-DFT, where the lowest-lying excitations for 2Sc are mostly localised to one side of the molecule and resemble d → d transitions, while some for 2La and 2Y-Et_2_O are combined MLCT and 5d → 5d transitions to the opposite Tripp ring.

### EPR spectroscopy

Continuous wave (c. w.) EPR spectroscopy was used to study the orbitally non-degenerate species 2Sc, 2Y and 2La. All three are EPR active as polycrystalline solids and in solution (1 mM Et_2_O or *n*Pr_2_O); spectra of 2Y in Et_2_O have been reported previously.^38^ We find better resolved frozen solution spectra in *n*Pr_2_O than in Et_2_O (although the spectra are consistent; see ESI†).

X-band spectra of powders at room temperature show features around *g* = 2, consistent with the formal M(ii) oxidation states. There is partial resolution of the metal hyperfine for 2Sc (^45^Sc, *I* = 7/2, 100% abundant) and 2Y (^89^Y, *I* = 1/2, 100%) but is unresolved for 2La (^139^La, *I* = 7/2, 100%). For 2Sc, a hyperfine octet is observed, with *g* = 2.000 and *A* = 145 MHz, for 2Y we observe a hyperfine doublet (estimated *A* = 14 MHz) with *g*_⊥_ = 2.005 and *g*_‖_ = 1.995, and for 2La, we observe *g*_⊥_ = 2.019 and *g*_‖_ = 1.958. There are small changes in these parameters upon cooling to 5 K, without any improvement in resolution; the limited resolution of the powder spectra is indicative of intermolecular magnetic interactions.

**Fig. 5:**
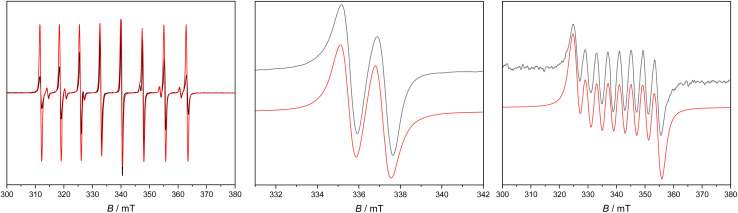
X-band c. w. EPR spectrum of 1 mM 2Sc in Et_2_O at 250 K (left), 2Y in *n*Pr_2_O at 180 K (middle), and 2La in Et_2_O at 200 K (right). Black, experimental; red, simulations with parameters in the text.

Fluid solution spectra of 2Y and 2La (Fig. 5 and Table 2) give a hyperfine doublet (*A*_iso_ = 46 MHz, *g*_iso_ = 1.9995) and octet (*A*_iso_ = 112 MHz, *g*_iso_ = 1.998), respectively. For 2Sc (Fig. 5 and Table 2) we also obtain an octet (*A*_iso_ = 205 MHz, *g*_iso_ = 1.989), but there is a second minor octet spectrum which differs subtly in the magnitude of the hyperfine (*A*_iso_ = 186 MHz), suggesting two Sc(ii) species in solution with a relative abundance of *ca*. 12 : 1 (similar features have been observed recently in a different Sc(ii) system^23^). In each case *g*_iso_ < *g*_e_, consistent with the formal d^1^ configuration. The isotropic part of the hyperfine interaction derives from s-orbital spin density, and from theoretical values of the hyperfine interaction for unit population of the valence s-orbitals^57^ we estimate 7.3% (2Sc), 3.7% (2Y) and 2.0% (2La) s-orbital character of the SOMO; these are in good agreement with DFT calculations (3.6%, 1.8%, and 1.0% s-orbital character, or 3.8%, 2.0%, 1.1% Löwdin s-orbital spin populations). In summary, 2Sc has the largest metal valence s-orbital spin density, then 2Y > 2La.

**Table 2:** Experimental (frozen solution) and calculated EPR parameters for 2Sc, 2Y and 2La. Calculated hyperfine coupling constants in MHz; experimental *A*_iso_ calculated as (*A*_‖_ + 2*A*_⊥_)/3. The calculated *g*_1_/*A*_1_ axes are along the M-bound arene direction

		*g*_1_	*g*_2_	*g*_3_	*A*_1_	*A*_2_	*A*_3_	*A*_iso_
2Sc	Exp.	1.990	2.002		195	210		205
	Calc.	1.990	2.009	2.015	159	171	184	171
2Y	Exp.	1.986	2.004		−36	−39		−38
2Y-Et_2_O	Calc.	1.983	2.004	2.006	−47	−49	−50	−48
2La	Exp.	1.952	2.005		100	110		107
	Calc.	1.954	1.971	1.993	101	106	108	105

**Fig. 6:**
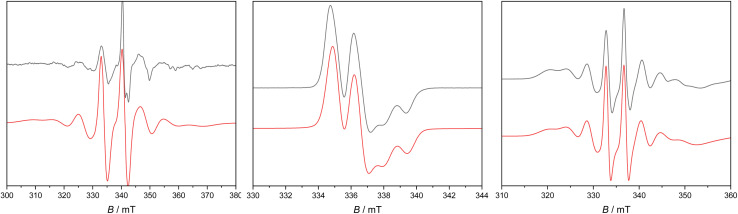
X-band c. w. EPR spectrum of 1 mM 2Sc (left) in Et_2_O at 130 K, 2Y (middle) and 2La (right) in *n*Pr_2_O at 60 K. Black experimental, red simulations.

Frozen solutions gave well-resolved spectra in each case (Fig. 6 and Table 2). For 2Sc there is a dominant perpendicular hyperfine coupling *A*_⊥_ ≈ 210 MHz (*g*_⊥_ = 2.002), from which we can determine *A*_‖_ ≈ 195 MHz (using *A*_iso_ = 205 MHz from the fluid spectra), and by simulation we find *g*_‖_ = 1.99. However, these parameters are not well defined as there is evidence of a second species. For 2Y and 2La the spectra appear axially symmetric giving *g*_⊥_ = 2.004, *g*_‖_ = 1.986, with a near isotropic metal hyperfine of *A*_⊥_ = −39, *A*_‖_ = −36 MHz for 2Y, while for 2La we find *g*_⊥_ = 2.005, *g*_‖_ = 1.952 with *A*_⊥_ = 110, *A*_‖_ = 100 MHz.

The anisotropy of the *g*-values for 2Sc, 2Y and 2La is due to the significant d-orbital character of the SOMO. The *g*_⊥_ > *g*_‖_ pattern indicates a dominant d_*x*^2^−*y*^2^_ contribution (where *z* is the M-bound arene direction), while the greater deviation of *g*_‖_ from *g*_e_ in the series 2La > 2Y > 2Sc is in keeping with greater spin–orbit coupling for the heavier elements,^58^ and also the trend in the lowest energy excited states (see above). A simple analysis of the anisotropic metal hyperfine interaction to give the metal d_*x*^2^−*y*^2^_ contribution to the SOMO [*A*_‖_ − *A*_⊥_ = (−6/7)*a*^2^P_d_, where *a* is the d_*x*^2^−*y*^2^_ coefficient of the SOMO and values of P_d_ are tabulated in ref. 57] gives 7.3% for 2Sc, 5.6% for 2Y and 4.9% for 2La. DFT calculations give much larger d-orbital contributions of 36%, 20%, and 14%, respectively (see above), despite also showing reasonable agreement with the measured hyperfine coupling constants (Table 2), implying an inadequacy in the simple analysis above; we have previously noted similar discrepancies in related [M^II^L_3_]^−^ systems (M = Sc, Y, La, Lu).^17^

Indeed, the EPR parameters and electronic structures of 2Sc, 2Y and 2La contrast to those related d^1^ [M^II^L_3_]^−^ species where the trigonal crystal field instead stabilises the d_*z*^2^_ (defined by the *C*_3_ axis) orbital, or a d/s hybrid giving rise to characteristic *g*_⊥_ < *g*_‖_ (≈*g*_e_) patterns.^2,16,17^ The electronic structures of the present compounds have more in common with [Sc(Cp^ttt^)_2_],^23^ where DFT calculations give a d_*x*^2^−*y*^2^_-dominated SOMO, and a similar *g*_⊥_ > *g*_‖_ pattern can be observed from the frozen solution EPR data. The hyperfine coupling in 2Y and 2La is much weaker than in the trigonal M(ii) cyclopentadienyl species: for example, [Y(Cp^R^)_3_]^−^ with various substituents [*e.g.* (Cp^R^)_3_ = Cp′_3_, Cp^t^_3_, {Cp′′_2_(C_5_H_5_)}] have |*A*_iso_| = 98–130 MHz;^17,59,60^ and [La(Cp^R^)_3_]^−^ (Cp^R^ = Cp′, Cp′′, Cp^tt^) have |*A*_iso_| = 390–640 MHz.^2,17,61^ Hence, there is greater metal character in the SOMOs of [M(Cp^R^)_3_]^−^ than in 2M. Comparing the present compounds with more symmetric sandwich compounds, [Y(Cp^iPr_5_^)_2_] and [La(Cp^iPr_5_^)_2_] have larger magnitude |*A*_iso_| = 505 and 2000 MHz, respectively,^14^ while curiously [Sc(Cp^ttt^)_2_] has smaller magnitude |*A*_iso_| = 83 MHz,^23^ although it has been reported that [Sc(Cp*)_2_] (not structurally authenticated) has |*A*_iso_| = 824 MHz.^23^

## Conclusions

In summary, we have reported a series of room-temperature stable crystalline divalent rare earth bis-tethered arene complexes of the form [M(NHAr^iPr_6_^)_2_] (2M; M = Sc, Y, La, Sm, Eu, Tm, Yb). In the case of Sc and La, these represent the second examples of neutral divalent complexes with these elements and are amongst just a few in any charge state. All 2M complexes feature close metal–arene contacts, which in 2Sc, 2Y, and 2La, results in an “open book” arene deformation suggestive of charge transfer, whereas in 2Sm, 2Eu, 2Tm, and 2Yb the equivalent arene groups remain planar. SQUID magnetometry and UV-Vis-NIR spectroscopy demonstrate 2Sm, 2Eu, 2Tm, and 2Yb are examples of 4f^*n*+1^ ions, and quantum chemical calculations show that the metal-localised 4f orbitals do not interact with the arene π-orbitals.

Solution-phase c. w. EPR spectroscopy of 2Sc, 2Y, and 2La are consistent with formal nd^1^ ions, where the SOMO has nd_(*x*^2^−*y*^2^)_ character with delocalisation of the spin onto the Tripp groups. Quantum chemical and *ab initio* calculations further support this description and reveal mixing between metal nd_(*x*^2^−*y*^2^)_ and arene–π orbitals to give δ-bonds, which explain the arene deformation in their structures.

The electronic structures of nd^1^2Sc, 2Y, and 2La closely resemble those of bis-benzene transition metal complexes; and, going forward, we posit that these rare nd_(*x*^2^−*y*^2^)_ configurations afford as-yet unexplored opportunities to tune the physicochemical properties of divalent rare earth ions with formal {5d/6s}^1^ valence electron configurations. This, along with work to probe the limits of the bis-{NHAr^iPr_6_^} framework to stabilise other divalent f-block ions, remains an active area of research in our laboratory.

## Data availability

The following CCDC references contain the ESI† crystal data for this article: 1Sc (2266263), 1Y (2266261), 1La (2266235), 1Tm (2266255), 1Yb (2266257), 1Y^β^ (2295306), 1La^β^ (2295307), 2Sc (2266264), 2Y (2266262), 2La (2266236), 2Sm (2266243), 2Eu (2266244), 2Tm (2266256), 2Yb (2266258), and 3 (2282041). Raw experimental data (NMR, ATR-IR, UV-Vis-NIR, SQUID Magnetometry) and computational inputs/outputs can be found freely at DOI: https://doi.org/10.48420/25245760.

## Author contributions

C. A. P. G. provided the original concept. R. E. M. synthesised and characterised the complexes. R. E. M. and C. A. P. G. collected, solved, and refined the single crystal XRD data. G. F. S. W. performed final refinement and validation of XRD data. J. A. S. performed SQUID magnetometry measurements and interpreted the data. T. H. performed EPR spectroscopy measurements, E. J. L. M. and D. C. supervised the EPR measurements and data interpretation. C. A. P. G. performed DFT calculations and interpretation, N. F. C. performed CASSCF and MC-PDFT calculations and interpreted the data. C. A. P. G. wrote the manuscript with contributions from all other authors.

## Conflicts of interest

There are no conflicts to declare.

## Supplementary Material

SC-OLF-D4SC03005B-s001

SC-OLF-D4SC03005B-s002
