# Supplementary material for: δ-Bonding modulates the electronic structure of formally divalent nd1 rare earth arene complexes
Source: Chem Sci. 2024 Aug 5;15(37):15160–9. doi: 10.1039/d4sc03005b (PMC11361033; doi:10.1039/d4sc03005b)
Supplement: SC-015-D4SC03005B-s001 [file SC-015-D4SC03005B-s001.pdf]

Supplementary Information (SI) for Chemical Science.
This journal is © The Royal Society of Chemistry 2024

Supporting Information for:

δ-Bonding Modulates the Electronic Structure of Formally Divalent n*d*^{1} Rare Earth Arene Complexes

Ross E. MacKenzie,[1,2] Tomáš Hajdu,[2] John A. Seed,[1,2] George F. S. Whitehead,[2] Ralph W. Adams,[2] Nicholas F. Chilton,[2,3] David Collison,[2] Eric J. L. McInnes,[2] Conrad A. P. Goodwin*[1,2]

[1] Centre for Radiochemistry Research, The University of Manchester, Oxford Road, Manchester, M13 9PL (UK).

[2] Department of Chemistry, The University of Manchester, Oxford Road, Manchester, M13 9PL (UK).

[3] Research School of Chemistry, The Australian National University, Sullivans Creek Road, Canberra, 2601 (Australia).

*Correspondence: conrad.goodwin@manchester.ac.uk

Table of Contents

S1. Experimental Details 3
Equipment, materials, and solvents 3
A note on NMR spectroscopy of paramagnetic samples 4
Synthesis of **1M** (M = Sc, Y, La, Tm, Yb) 8
Synthesis of **2M** (M = Sc, Y, La, Tm) 12
Synthesis of **2M** (M = Sm, Eu, Yb) 14
S2. Crystallography 19
General considerations 19
S3. Molecular structures 27
Complexes **1M** (M = Sc, Y, La, Tm, Yb) 27
Bond metrics for **1M** (M = Sc, Y, La, Tm, Yb), and [Y(NHAr^{iPr6})$_2$(Cl)] 32
Complexes **2M** (M = Sc, Y, La, Sm, Eu, Tm, Yb) 33
Bond metrics for **2M** (M = Sc, Y, La, Sm, Eu, Tm, Yb) 40
A discussion of M–N bonding in **1M** and **2M** 41
S4. NMR spectroscopy plots 43
NMR spectra of **1M** (M = Sc, Y, La, Tm, Yb) 43
NMR spectra of **2M** (M = Sc, Y, La, Sm, Eu, Tm, Yb) 48
Magnetic moments determined by NMR spectroscopy (Evans method) 57
S5. ATR-IR spectra 58
ATR-IR spectra of **1M** (M = Sc, Y, La, Tm, Yb) and **2M** (M = Sc, Y, La, Sm, Eu, Tm, Yb) 58
Comparative ATR-IR spectra for **1M** (M = Sc, Y, La) and **2M** (M = Sc, Y, La, Sm, Eu, Tm, Yb) 64
S6. UV-Vis-NIR spectra 67
UV-Vis-NIR spectra of **1M** (M = Sc, Y, La, Tm, Yb) 67
UV-Vis-NIR spectra of**2M** (M = Sc, Y, La, Sm, Eu, Tm, Yb) 70
Comparison of UV-Vis-NIR spectra between **1M** and **2M** series. 74
S7. EPR Spectroscopy 78
EPR spectroscopy data and discussion 78
S8. SQUID Magnetometry 85
Coupling schemes 86
Magnetometry data and discussion for **1Tm**, **2Sm**, **2Eu**, and **2Tm** 89
Magnetometry data and discussion for **2Sc**, **2Y**, and **2La** 101
S9. Density Functional Theory calculations 108
Density Functional Theory – General considerations 108
Notes on DFT geometry optimisations 109
TD-DFT methodology 114
TD-DFT vs sTD-DFT with **2Sc**, **2Y**, and **2La** 115
Additional geometry optimisation of **2Y** to produce **2Y-Et$_2$O**, and TD-DFT screening 120
Natural transition orbitals for **2Sc**, **2Y-Et$_2$O**, and **2La** 122
Molecular orbital compositions and isosurfaces 125
Calculated EPR coupling constants and g-matrices for **2Sc**, **2Y**, **2Y-Et$_2$O**, and **2La** 133
S10. CASSCF calculations 135
S11. Calculated coordinates 141
Constrained optimisations 141
Full optimisations 164
S12. References 187

S1. Experimental Details

Equipment, materials, and solvents

Unless otherwise described, all syntheses and manipulations were conducted under BOC PureShield argon (99.995%) with rigorous exclusion of oxygen and water using Schlenk line and glove box techniques in an MBraun Lab Star™. 3 Å molecular sieves were activated by heating for 8 hours at 300°C, 10^{-3} mbar. THF, *n*-hexane, *n*-pentane, Et_2O, and toluene were degassed by sparging and dried by passage through neutral allumina collumns (INERT Corp.). THF was then degassed *in vacuo* and stored over 3 Å molecular sieves for 7 days before use. *n*-hexane, *n*-pentane, Et_2O, and toluene were degassed *in vacuo* and stored over a K mirror and used immediately. d_6-benzene (Merck) was dried by refluxing over K metal for 4-5 days followed by vacuum transfer and storage in a J. Youngs valve appended vessel. $[LaI_3(THF)_4]$ and $YI_3(THF)_{3.5}$ were prepared as described previously,[1] $[ScI_3(THF)_3]$, $[TmI_3(THF)_{3.5}]$, and $[YbI_3(THF)_{3.5}]$ synthesised analogously. $MI_2(THF)_2$ (M = Sm, Yb) were prepared as described previously from M metal and freshly recrystallised (Et_2O) 1,2-diiodoethane (Alfa Aesar) in THF,[2] which gave crystalline $[MI_2(THF)_5]$ which were partially desolvated *in vacuo* (10^{-3} mbar, 8 hours) – the Eu analogue was prepared the same way. $KNHAr^{iPr6}$ was prepared from H_2NAr^{iPr6},[3] and BnK in toluene. Exemplar ^1H and $^{13}C\{^1H\}$ NMR spectra of $KNHAr^{iPr6}$ are provided in **Figure S1** and **Figure S2**. KC_8 was synthesised by heating graphite (Merck, <20 µm, dried at 300°C, 10^{-3} mbar) and $^1/_8$ molar equivalents of K^0 (Merck) under Ar. All glassware, and glass-fiber filter discs, were stored in an oven (150 °C), and glassware was further dried *in vacuo* (10^{-3} mbar) with heating from a butane flame. Solution phase UV-Vis-NIR spectra were collected at ambient temperature using a PerkinElmer Lambda 1050 UV-Vis-NIR spectrometer. The solution was contained in a low volume (1 mL) screw-capped quartz cuvette (10 × 4 mm path length fluorescence cell). ATR FT-IR spectra of microcrystalline samples were collected using a Bruker ALPHA II FT-IR spectrometer equipped with a Platinum ATR module with diamond window. NMR spectroscopic data collection was performed on either a Bruker Avance III (400 MHz), Bruker Advance III HD (400 MHz), or Bruker Ascend (700 MHz) between 295 K to 299 K. Elemental microanalyses (C/H/N) were carried out by Martin

Jennings and Anne Davies at the University of Manchester. Variable-temperature magnetic moment data were recorded in an applied direct current (DC) field of 1 kOe on a Quantum Design MPMS3 superconducting quantum interference device magnetometer using recrystallised powdered samples. Measurements were performed in dc scan mode using 40 mm scan length and 6 s scan time. Samples were carefully checked for purity and data reproducibility between independently prepared batches. Samples were crushed with a mortar and pestle under an argon atmosphere and immobilised in an eicosane matrix within 400 MHz Wilmad borosilicate NMR tubes to prevent sample reorientation during measurements. The tube was flame-sealed under dynamic vacuum (1 × 10^{-3} mbar) to a length of approximately 4 cm and mounted in the centre of a drinking straw, with the straw fixed to the end of an MPMS 3 sample rod. Care was taken to ensure complete thermalisation of the sample before each data point was measured by employing delays at each temperature point as well as a slow cooling rate (0.5 $K{\cdot}min^{-1}$). The sample was held at 1.8 K for 180 minutes before isothermal magnetization measurements to account for slow thermal equilibration of the sample. Diamagnetic corrections for lattice solvents were applied using tabulated Pascal constants. The magnetic susceptibility was corrected for the intrinsic diamagnetism of the sample estimated as the molecular weight ($g{\cdot}mol^{-1}$) multiplied by −0.4-1.0 × 10^{-6} cm^3 $K{\cdot}mol^{-1}$ or in some instances (**1Tm**, **2Tm**, **2Sm**) we have corrected for the $\{NHAr^{iPr6}\}_2$ component by performing DC magnetic susceptibility measurements from 1.8 to 300 K on a diamagnetic analogue complex, $[Ca(NHAr^{iPr6})_2]{\cdot}(Et_2O)$ (2Ca) [§] and subtracting from the measured magnetic susceptibility. Measurements were corrected for the effect of the blank sample holders (flame-sealed Wilmad NMR tube and straw) and eicosane matrix.

A note on NMR spectroscopy of paramagnetic samples

All spectra were referenced to internal solvent residuals (^1H and ^{13}C) or externally to 10% TMS in d_3-chloroform *via* Equation S1, which is the IUPAC recommended convention.

[§] To be reported separately.

Equation S1. $$\Delta\ (Hz) = \frac{SR^{1H}}{SF^{1H}} \times\ SF^{NUC}$$

Where SR^{1H} is the spectrum reference frequency (in Hz) of a reference ^{1}H NMR spectrum collected with TMS set to 0 ppm collected under the same experimental conditions; SF^{1H} is the spectrometer frequency (in MHz) for the ^{1}H nucleus; SF^{NUC} is the spectrometer frequency (in MHz) of the nucleus in question. The answer is given in Hz.

Paramagnetic samples become magnetised in the presence of an external magnetic field, such as that of an NMR spectrometer. The level of magnetization will approximately follow Curie's law when saturation of magnetization is not reached ($\mu_B \leq k_BT$). The magnetic response of a sample is proportional to: (i) sample temperature; (ii) external field strength; and (iii) sample concentration. This necessarily affects the reproducibility of the chemical shifts given for paramagnetic samples – a sample run at a different concentration, or a different field strength, or at a different temperature, will produce a different paramagnetic contribution to the observed chemical shift. Moreover, the direction that an individual chemical shift will change (upfield or downfield) cannot easily be predicted.[4] Finally, modern convention to reference chemical shift relative to solvent residual peaks further complicates the comparison of multiple samples as the factors listed above will also change the absolute shift of the solvent peak (relative to the spectrometer proton frequency) as the susceptibility of solvent molecules may differ from the ligand atoms surrounding a paramagnetic ion. Though, solvent effects even in diamagnetic NMR samples can vary chemical shift by several ppm for some nuclei.[5]

Considering these caveats, we report our data as it is output from experiment with rounding to two decimal places as this is convention. We defer to the expertise of the reader to interpret the data reported here in a way that is appropriate for their needs.

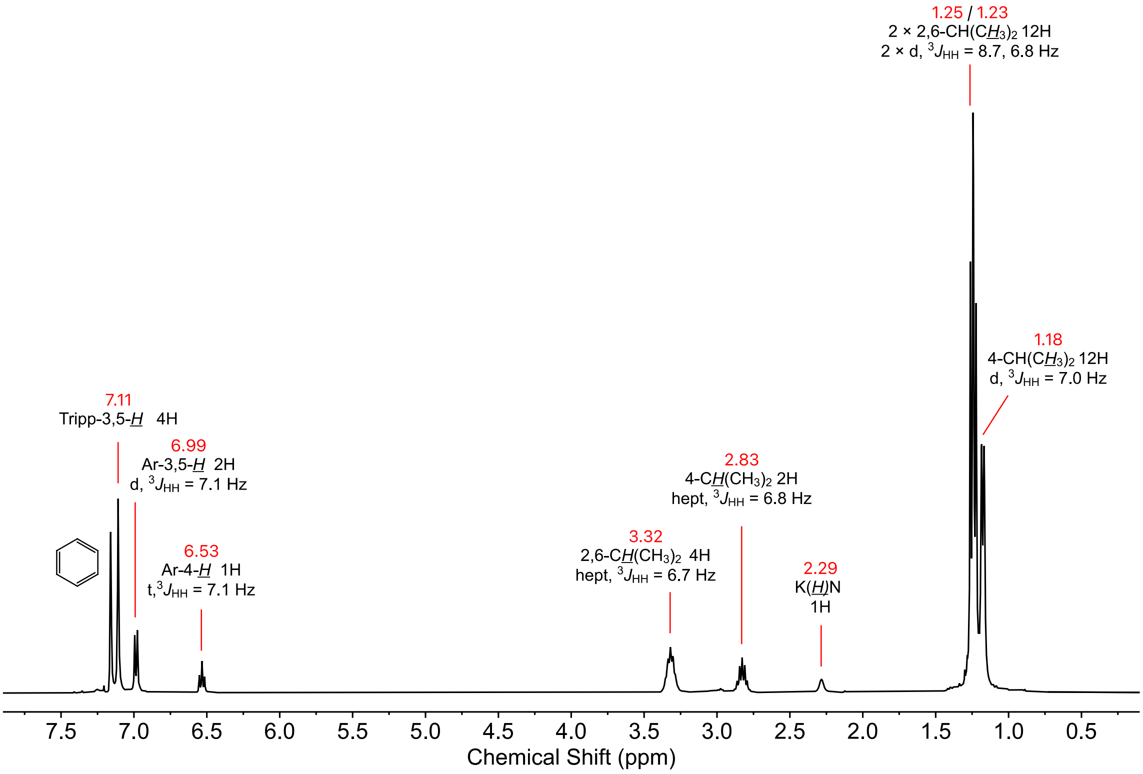

Figure S1. ^{1}H NMR spectrum of KNHAr^{iPr6} in d_6-benzene.

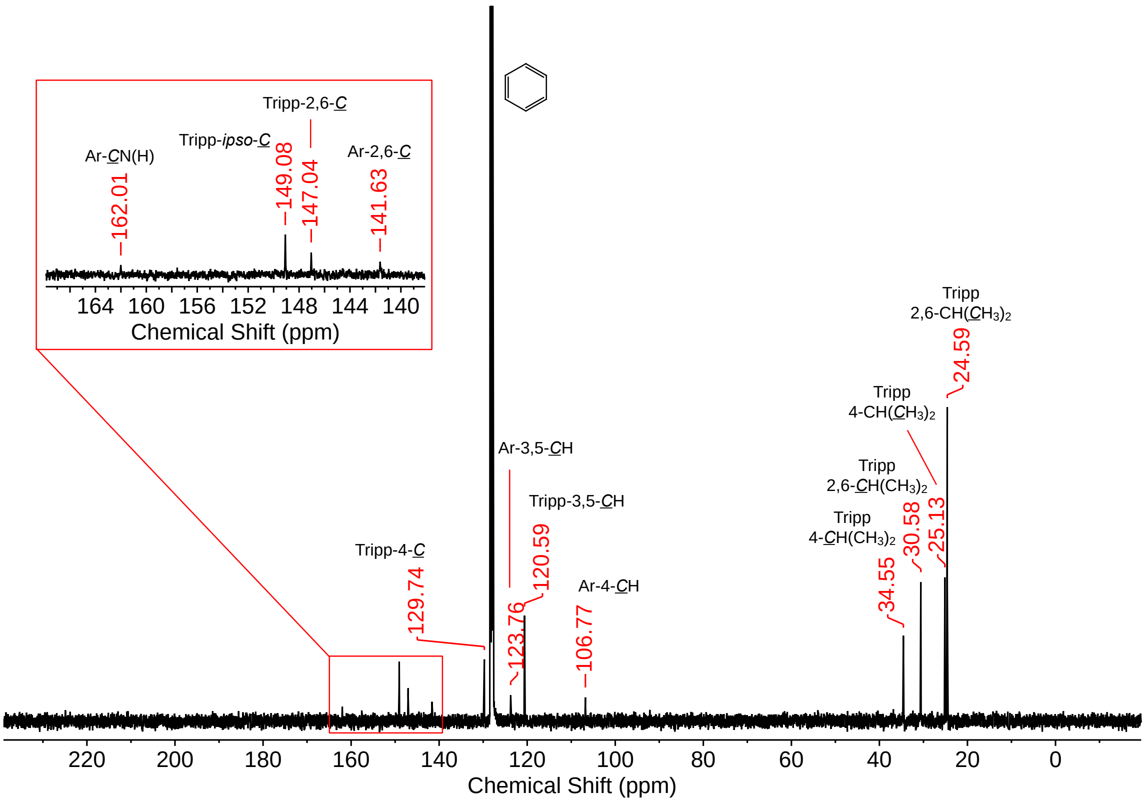

Figure S2. $^{13}C\{^{1}H\}$ NMR spectrum of KNHAr^{iPr6} in d_6-benzene.

Table S1. Compound numbering, formula, reaction scale (by metal quantity used), yield, and then % yield where appropriate.

	Scale (mmol metal)	Formula	Yield (g)	% yield
**1Sc**	2	$[Sc(NHAr^{iPr6})_2(I)]\cdot(C_7H_8)_2$	1.329	57
**1Y**	2	$[Y(NHAr^{iPr6})_2(I)]\cdot(C_7H_8)_2$	1.510	62
**1La**	2	$[La(NHAr^{iPr6})_2(I)]\cdot(C_7H_8)_2$	1.360	54
**1Tm**	2	$[Tm(NHAr^{iPr6})_2(I)]\cdot(C_7H_8)_2$	1.218	47
**1Yb**	1	$[Yb(NHAr^{iPr6})_2(I)]\cdot(C_7H_8)_2$	–	–
**2Sc**	0.5	$[Sc(NHAr^{iPr6})_2]$	0.277	53
**2Y**	0.5	$[Y(NHAr^{iPr6})_2]\cdot(OC_4H_{10})$	0.298	55
**2La**	0.5	$[La(NHAr^{iPr6})_2]\cdot(OC_4H_{10})$	0.257	45
**2Sm**	1	$[Sm(NHAr^{iPr6})_2]\cdot(OC_4H_{10})$	0.7955	69
**2Eu**	1	$[Eu(NHAr^{iPr6})_2]$	0.216	20
**2Tm**	0.5	$[Tm(NHAr^{iPr6})_2]\cdot(OC_4H_{10})$	0.376	65
**2Yb**	1	$[Yb(NHAr^{iPr6})_2]$	0.960	82
**3**	–	$HN{=}C_6H_3$-2,6-$(C_6H_3$-2,4,6-$i Pr_3)$-4-{HN-C_6H_3-2,6-$(C_6H_3$-2,4,6-$iPr_3)_2$	–	–

Note: Yields are representative of a single iteration only, and are intended only to indicate what might be expected from these unoptimised reactions.

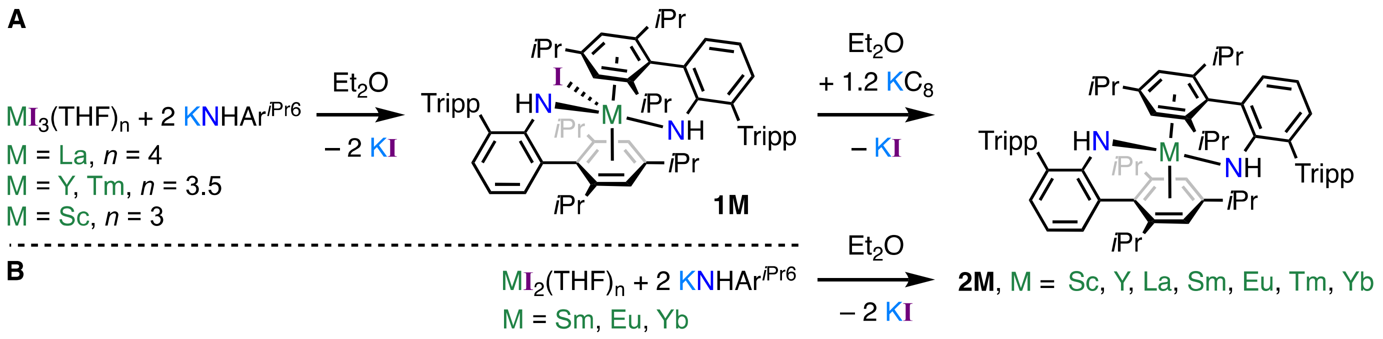

Scheme 1. Synthesis of the complexex herein, denoting the numbering scheme.

Synthesis of ***1M*** *(M = Sc, Y, La, Tm, Yb)*

Synthesis of [Sc(NHAr^{iPr6})$_2$(I)]·(C$_7$H$_8$)$_2$ (1Sc). Et_2O (50 mL) was added to a pre-cooled (–98°C) stirring mixture of solid [ScI_3(THF)$_3$] (1.616 g, 2 mmol) and KNHAr^{iPr6} (2.072 g, 4 mmol, 2 equiv.) in a glass Schlenk vessel equipped with a PTFE-coated stirrer bar. The mixture was allowed to warm to room temperature and quickly became yellow with a fine white precipitate, presumed to be KI. After stirring at room temperature overnight (16 hours), the volatiles were removed *in vacuo* (10^{-3} mbar) which left a bright yellow powder. Toluene (20 mL) was added and briefly (< 1 min) refluxed with manual agitation to loosen solids from the vessel walls. The bright yellow solution and fine white solids were allowed to settle before filtration through a glass microfibre filter disc. Concentration of the bright yellow supernatant to *ca*. 1 mL gave a large quantity of yellow solids which were heated into solution and allowed to cool slowly to room temperature to give large yellow blocks of **1Sc**. This mixture was stored at 5°C for 2 hours followed by –30°C for 16 hours to increase the yield. Crystals were isolated by decanting the supernatant followed by drying *in vacuo* (10^{-3} mbar, 2 hours). A second crop was obtained in a similar fashion (combined yield = 1.329 g, 57%).

Elemental analysis on $C_{72}H_{100}IN_2Sc\cdot(C_7H_8)_n$ for n = 2 calc. (%): C = 76.529, H = 8.663, N = 2.076; for n = 0 calc. (%): C = 74.201, H = 8.649, N = 2.404; found (%): C = 69.90, H = 8.44, N = 2.07 – low carbon values were found across duplicate samples.

^{1}H NMR (d_6-benzene, 400.13 MHz 298 K): δ = 7.33 (s, 8H, Tripp-3,5-*H*), 6.97 (d, $^3J_{HH}$ = 7.3 Hz, 4H, Ar-3,5-*H*), 6.69, (t, $^3J_{HH}$ = 7.4 Hz, 2H, Ar-4-*H*), 5.64 (s, 2H, ScN(*H*)), 3.01 (hept, $^3J_{HH}$ = 7.0 Hz, 8H, Tripp-2,6-C*H*(CH$_3$)$_2$), 2.66 (hept, $^3J_{HH}$ = 7.2 Hz, 4H, Tripp-4-C*H*(CH$_3$)$_2$), 1.40 (d, $^3J_{HH}$ = 6.9 Hz, 24H, Tripp-4-CH(C*H*$_3$)$_2$), 1.32 (d, $^3J_{HH}$ = 6.9 Hz, 24H, Tripp-2,6-CH(C*H*$_3$)$_2$), 1.11 (d, $^3J_{HH}$ = 6.9 Hz, 24H, Tripp-2,6-CH(C*H*$_3$)$_2$).

^{13}C{^{1}H} NMR (d_6-benzene, 100.62 MHz, 298 K): δ = 155.18 (Ar-*C*N(H)-Sc), 149.69 (Tripp-*ipso*-*C*), 148.71 (Tripp-2,6-*C*), 148.08 (Ar-2,6-*C*), 131.59 (Tripp-4-*C*), 127.10 (Ar-3,5-*C*H), 123.47 (Tripp-3,5-*C*H), 116.32 (Ar-4-*C*H), 34.03 (Tripp-4-*C*H(CH$_3$)$_2$), 31.28 (Tripp-2,6-*C*H(CH$_3$)$_2$), 25.96 (Tripp-4-CH(*C*H$_3$)$_2$), 24.34 (Tripp-2,6-CH(*C*H$_3$)$_2$), 24.10 (Tripp-2,6-CH(*C*H$_3$)$_2$).

UV-Vis-NIR (Et_2O): λ_{max} (cm^{-1}; ε) = A broad feature peak extends from ~500 nm (20,000 cm^{-1}) into the UV region, and beyond our spectral range.

FT-IR (ATR, microcrystalline): cm^{-1} = 3,482 (vs), 3,383 (vs), 3,284 (vs), 3,198 (vs), 3,052 (s), 3,021 (vs), 2,957 (vw), 2,927 (w), 2,867 (w), 2,754 (vs), 1,761 (vs), 1,603 (m), 1,585 (s), 1,574 (s), 1,566 (s), 1,541 (vs), 1,459 (vw), 1,441 (w), 1,406 (vw), 1,381 (vw), 1,361 (vw), 1,319 (w), 1,258 (vw), 1,243 (vw), 1,233 (vw), 1,188 (s), 1,163 (m), 1,153 (s), 1,126 (s), 1,097 (w), 1,077 (vw), 1,056 (m), 1,036 (m), 1,007 (m), 956 (s), 939 (m), 923 (s), 900 (s), 876 (vw), 863 (vw), 845 (w), 830 (w), 795 (w), 777 (m), 752 (vw), 730 (m), 695 (s), 668 (w), 652 (w), 635 (m), 619 (w), 602 (w), 586 (vw), 551 (m), 534 (m), 514 (w), 504 (m), 487 (m), 473 (m), 464 (m), 452 (m), 442 (m), 430 (m), 419 (m).

Synthesis of [Y(NHAr^{iPr6})$_2$(I)]·(C_7H_8)$_2$ (1Y). $YI_3(THF)_{3.5}$ (1.382 g, 2 mmol), KNHAr^{iPr6} (2.072 g, 4 mmol, 2.0 equiv.). Bright yellow solution and 2 crops of bright yellow blocks (yield = 1.510 g, 62%). Elemental analysis on $C_{72}H_{100}IN_2Y\cdot(C_7H_8)_n$ for n = 2 calc. (%): C = 74.115, H = 8.389, N = 2.010; for n = 0 calc. (%): C = 71.505, H = 8.334, N = 2.316; found (%): C =73.85, H = 8.54, N = 2.01.

^{1}H NMR (d_6-benzene, 400.13 MHz 298 K): δ = 7.35 (s, 8H, Tripp-3,5-*H*), 7.01 (d, $^3J_{HH}$ = 7.2 Hz, 4H, Ar-3,5-*H*), 6.69, (t, $^3J_{HH}$ = 7.4 Hz, 2H, Ar-4-*H*), 4.71 (s, 2H, YN(*H*)), 3.04 (hept, $^3J_{HH}$ = 6.6 Hz, 8H, Tripp-2,6-C*H*(CH_3)$_2$), 2.51 (hept, $^3J_{HH}$ = 6.6 Hz, 4H, Tripp-4-C*H*(CH_3)$_2$), 1.47 (d, $^3J_{HH}$ = 6.8 Hz, 24H, Tripp-4-CH(C*H*$_3$)$_2$), 1.29 (d, $^3J_{HH}$ = 6.8 Hz, 24H, Tripp-2,6-CH(C*H*$_3$)$_2$), 1.13 (d, $^3J_{HH}$ = 6.6 Hz, 24H, Tripp-2,6-CH(C*H*$_3$)$_2$).

^{13}C{^{1}H} NMR (d_6-benzene, 100.62 MHz, 298 K): δ = 156.34 (d, $^2J_{YC}$ = 3.6 Hz, Ar-*C*N(H)-Y), 149.72 (Tripp-*ipso*-*C*), 148.79 (Tripp-2,6-*C* + Ar-2,6-*C*), 131.59 (Tripp-4-*C*), 126.07 (Ar-3,5-*C*H), 123.69 (Tripp-3,5-*C*H), 114.92 (Ar-4-*C*H), 33.92 (Tripp-4-*C*H(CH_3)$_2$), 31.14 (Tripp-2,6-*C*H(CH_3)$_2$), 26.31 (Tripp-4-CH(*C*H$_3$)$_2$), 24.43 (Tripp-2,6-CH(*C*H$_3$)$_2$), 23.94 (Tripp-2,6-CH(*C*H$_3$)$_2$).

^{89}Y-^{1}H HMBC NMR (d_6-benzene, 34 MHz / 700 MHz, 298 K): δ = 516.43 / 4.63 (*Y*N(*H*)).

UV-Vis-NIR (Et_2O): λ_{max} (cm^{-1}; ε) = A broad feature extends from ~500 nm (20,000 cm^{-1}) into the UV region, and beyond our spectral range.

FT-IR (ATR, microcrystalline): cm^{-1} = 3,036 (s), 3,007 (s), 2,957 (vw), 2,925 (vw), 2,865 (w), 2,781 (m), 2,738 (s), 1,837 (vs), 1,735 (s), 1,589 (s), 1,541 (vs), 1,496 (vs), 1,445 (vw), 1,393 (m), 1,369 (w), 1,307 (w), 1,262 (m), 1,254 (m), 1,209 (vs), 1,178 (vs), 1,149 (m), 1,124 (s), 1,093 (w), 1,052

(w), 972 (s), 939 (s), 890 (vw), 843 (m), 828 (m), 810 (s), 758 (m), 744 (m), 725 (vs), 697 (vw), 617 (w), 592 (w), 567 (w), 538 (vw), 528 (vw), 508 (vw), 478 (w), 467 (vw), 436 (vw), 407 (vw).

Synthesis of [La(NHAr^{iPr6})$_2$(I)]·(C_7H_8)$_2$ (1La). [LaI_3(THF)$_4$] (1.444 g, 2 mmol), KNHAr^{iPr6} (2.072 g, 4 mmol, 2.0 equiv.). Bright yellow solution and 2 crops of bright yellow blocks (yield = 1.360 g, 54%). Elemental analysis on $C_{72}H_{100}IN_2La$·(C_7H_8)$_n$ for *n* = 2 calc. (%): C = 71.548, H = 8.099, N = 1.940; for *n* = 0 calc. (%): C = 68.666, H = 8.003, N = 2.224; found (%): C = 67.77, H = 8.04, N = 1.89.

^{1}H NMR (d_6-benzene, 400.13 MHz, 298 K): δ = 7.35 (m, 8H, Tripp-3,5-*H*), 7.04 (m, 4H, Ar-3,5-*H*), 6.68, (td, $^3J_{HH}$ = 7.4, 3.4 Hz, 2H, Ar-4-*H*), 4.89 (s, 2H, LaN(*H*)), 3.25–2.70 (m, 8H, Tripp-2,6-C*H*(CH_3)$_2$), 1.52 (s, 24H, Tripp-4-CH(C*H*$_3$)$_2$), 1.34–1.25 (m, 24H, Tripp-2,6-CH(C*H*$_3$)$_2$), 1.12 (s, 24H, Tripp-2,6-CH(C*H*$_3$)$_2$).

^{13}C{^{1}H} NMR (d_6-benzene, 100.62 MHz, 298 K): δ = 158.85 (s, Ar-*C*N(H)-La), 121.51 (Tripp-3,5-*C*H), 113.54 (Ar-4-*C*H), 30.97 (br. Tripp-4-*C*H(CH_3)$_2$ + Tripp-2,6-*C*H(CH_3)$_2$), 26.76 (Tripp-4-CH(*C*H$_3$)$_2$), 24.81 (Tripp-2,6-CH(*C*H$_3$)$_2$), 24.38 (Tripp-2,6-CH(*C*H$_3$)$_2$) – no further features could be conclusively identified. Given the M–H coupling observed in **2Yb** (*vide infra*), we tentatively suggest that the broadness of the ^{1}H NMR spectrum for **1La**, and the missing ^{13}C peaks in the ^{13}C{^{1}H} NMR spectrum, are the result of coupling to the quadrupolar ^{139}La (*I* = $^7/_2$, >99.9% nat. abund.).

UV-Vis-NIR (Et_2O): λ_{max} (cm^{-1}; *ε*) = A broad feature extends from ~500 nm (20,000 cm^{-1}) into the UV region, and beyond our spectral range.

FT-IR (ATR, microcrystalline): cm^{-1} = 3,481 (vw), 3,379 (vw), 2,957 (vs), 2,927 (s), 2,865 (m), 1,759 (vw), 1,603 (vw), 1,583 (vw), 1,459 (s), 1,441 (s), 1,410 (vs), 1,381 (m), 1,361 (s), 1,328 (vw), 1,317 (w), 1,258 (vs), 1,158 (vw), 1,097 (s), 1,071 (vs), 1,056 (m), 1,036 (w), 1,019 (w), 1,005 (m), 939 (vw), 921 (vw), 892 (w), 876 (s), 847 (s), 830 (s), 795 (s), 777 (m), 748 (vs), 721 (vw), 662 (w), 652 (w), 608 (w), 571 (m), 547 (w), 518 (vw), 479 (w), 464 (m), 458 (m), 442 (m), 425 (s), 419 (vs).

Synthesis of [Tm(NHAr^{iPr6})$_2$(I)]·(C_7H_8)$_2$ (1Tm). TmI_3(THF)$_{3.5}$ (1.1339 g, 2 mmol), KNHAr^{iPr6} (2.072 g, 4 mmol, 2.0 equiv.). Bright yellow solution and 2 crops of bright yellow blocks (yield = 1.218 g, 47%).

Elemental analysis on $C_{72}H_{100}IN_2Tm\cdot(C_7H_8)_n$ for n = 2 calc. (%): C = 70.091, H = 7.934, N = 1.901; for n = 0 calc. (%): C = 67.067, H = 7.817, N = 2.173; found (%): C = 69.85, H = 7.82, N = 1.81.

^{1}H NMR (d_6-benzene, 400.13 MHz, 298 K): δ = 3.03 (br. s, FWHM = 114 Hz), 2.12 (br. s, FWHM = 36 Hz), 1.28 (br. s, FWHM = 23 Hz), –38.44 (br. s, FWHM = 79 Hz).

UV-Vis-NIR (Et_2O): λ_{max} (cm^{-1}; ε) = 801 (12,485, 26), 786 (12,723, 44), 776 (12,884, 29), 761 (13,149, 27), 742 (13,475, 27), 707 (14,145, 26), 696 (14,372, 31), 687 (14,556, 51), 677 (14,761, 32), 667 (14,997, 31).

Magnetic moment (Evans method, d_6-benzene, 298 K): μ_{eff} = 6.06(2) μB.

FT-IR (ATR, microcrystalline): cm^{-1} = 3,294 (vs), 3,023 (vs), 2,957 (vw), 2,925 (w), 2,865 (w), 2,754 (vs), 1,603 (s), 1,578 (s), 1,541 (vs), 1,494 (vs), 1,459 (vw), 1,408 (vw), 1,381 (vw), 1,361 (vw), 1,321 (w), 1,289 (s), 1,254 (vw), 1,241 (vw), 1,188 (s), 1,163 (m), 1,153 (s), 1,130 (vs), 1,097 (m), 1,075 (vw), 1,030 (s), 1,005 (m), 980 (vs), 956 (vs), 937 (m), 923 (s), 900 (s), 876 (w), 857 (w), 847 (vw), 832 (vw), 795 (s), 777 (s), 750 (vw), 728 (vw), 695 (w), 664 (w), 654 (w), 623 (w), 590 (w), 580 (w), 561 (m), 553 (m), 524 (m), 501 (m), 483 (m), 464 (vw), 440 (s), 421 (m), 405 (m).

Synthesis of [Yb(NHAr^{iPr6})$_2$(I)]·(C_7H_8)$_2$ (1Yb), and isolation of HN=C_6H_3-2,6-(2,4,6-*i*Pr_3)-4-{HN-C_6H_3-2,6-(2,4,6-*i*Pr_3)$_2$ (3). Et_2O (50 mL) was added to a pre-cooled (–98°C) stirring mixture of solid $YbI_3(THF)_{3.5}$ (0.770 g, 1 mmol) and KNHAr^{iPr6} (1.082 g, 2 mmol, 2.0 equiv.) in a glass Schlenk vessel euipped with a PTFE-coated stirrer bar. The mixture was allowed to warm to room temperature and quickly became bright green with a fine white precipitate, presumed to be KI. After stirring at room temperature overnight (16 hours), the volatiles were removed *in vacuo* (10^{-3} mbar) which left a bright green powder. Toluene (15 mL) was added and briefly (< 1 min) refluxed with manual agitation to loosen solids from the vessel walls. The bright green solution and fine white solids were allowed to settle before filtration through a glass microfibre filter disc. Concentration of the bright green supernatant to *ca*. 3 mL gave a small quantity of green solids which were heated into solution and allowed to cool slowly to room temperature, and then stored at 5°C for 16 hours. A crop of colourless crystals, **3**, was isolated first by decanting the dark teal supernatant and the colourless crystals were dried *in vacuo* (yield = 0.130 g), though these were contaminated with traces of **1Yb** which could

not be removed by fractional crystallization. The supernatant was concentrated to *ca.* 1 mL and stored at –30°C for 16 hours which gave a mixed crop of colorless (**3**) and teal (**1Yb**) plates, followed by a subsequent crop upon further storage at –30°C (yield for the combined second and third crops = 0.258 g).

*Synthesis of **2M** (M = Sc, Y, La, Tm)*

The synthesis of [M(NHAr^{iPr6})$_2$] complexes where M = Sc, Y, La, and Tm was performed by low-temperature reduction of the correspondong trivalent **1M** complexes in Et_2O, followed by crystallisation from the same solvent, or *n*-hexane in the case of **2Sc**. All handling of **2M** (M = Sc, Y, La, and Tm) was performed in silylated glassware using glass-coated stirrer bars.

Synthesis of [Sc(NHAr^{iPr6})$_2$] (2Sc). Et_2O (15 mL) was added to a pre-cooled (–98°C) stirring mixture of solid **1Sc** (0.583 g, 0.5 mmol) and KC_8 (0.083 g, 0.6 mmol, 1.2 equiv.) in a glass Schlenk vessel equipped with a glass-coated stirrer bar. The mixture was allowed to slowly warm to room temperature while submerged in a cooling bath, during which time it became significantly darker, from a yellow mixture to a dark brown/black mixture. After stirring for 2 hours, the cooling bath had warmed to *ca*. 0°C, at which point the vessel was removed and allowed to warm up to room temperature with stirring for 15-20 minutes. The fine grey suspension, presumably a mixture of graphite, KI, and excess KC_8, was allowed to settle at room temperature, and the dark solution was filtered through a glass microfibre filter disc. The volatiles were removed *in vacuo* (10^{-3} mbar) which left a slightly sticky dark blue powder. Hexane (3 mL) was added with manual agitation to loosen solids from the vessel walls. The dark blue solution was filtered through a glass microfibre filter disc, concentrated to *ca.* 1 mL, and stored at –30°C for 16 hours which afforded a crop of dichroic red/green planks which were dried *in vacuo* (10^{-3} mbar, 1 hour). Yield = 0.277 g, 53%.

Elemental analysis on $C_{72}H_{100}N_2Sc$ calc. (%): C = 83.268, H = 9.705, N = 2.697; found (%): C = 79.89, H = 9.60, N = 2.54.

^{1}H NMR (d_6-benzene, 400.13 MHz, 298 K): Only peaks attributable to diamagnetic impurities could be identified.

UV-Vis-NIR (Et_2O): λ_{max} (cm^{-1}; ε) = 756 (13,412, 805), 575 (17,394, 1,820), 469 (21,315, 2,471) – note that these peaks are fairly broad. The positions and ε values here result from fitting each broad feature to a single Gaussian curve.

FT-IR (ATR, microcrystalline): cm^{-1} = 3,334 (vs), 3,042 (vs), 3,023 (vs), 2,957 (vw), 2,925 (w), 2,865 (w), 2,750 (vs), 2,721 (vs), 2,705 (vs), 1,605 (s), 1,583 (s), 1,566 (s), 1,527 (vs), 1,459 (w), 1,408 (vw), 1,381 (vw), 1,361 (vw), 1,317 (w), 1,264 (vw), 1,248 (vw), 1,188 (s), 1,163 (m), 1,126 (s), 1,100 (m), 1,073 (vw), 1,001 (m), 956 (s), 937 (m), 923 (s), 873 (vw), 855 (vw), 847 (vw), 830 (w), 789 (w), 769 (s), 746 (vw), 699 (s), 662 (w), 594 (w), 547 (w), 522 (m), 506 (w), 493 (m), 483 (m), 467 (m), 440 (m), 420 (m).

Data for [Y(NHAr^{iPr6})$_2$]·(Et_2O) (2Y). As for **2Sc**, except the complex was crystallised from the Et_2O supernatant, rather than following extraction with *n*-hexane. **1Y** (0.605 g, 0.5 mmol), KC_8 (0.081 g, 0.6 mmol, 1.2 equiv.). Red/brown solution and 1 crop of red/brown planks. Yield = 0.298 g, 55%).

Elemental analysis on $C_{72}H_{100}N_2Y\cdot(OC_4H_{10})_n$ for n = 1 calc. (%): C = 78.922, H = 9.586, N = 2.422; for n = 0 calc. (%): C = 79.888, H = 9.311, N = 2.588; found (%): C = 77.59, H = 9.54, N = 2.37.

^{1}H NMR (d_6-benzene, 400.13 MHz, 298 K): Only peaks attributable to diamagnetic impurities could be identified.

UV-Vis-NIR (THF): λ_{max} (cm^{-1}; ε) = 729 (13,709, 599), 463 (21,589, 1,787).

FT-IR (ATR, microcrystalline): cm^{-1} = 3,479 (vs), 3,325 (vs), 3,065 (vs), 3,039 (vs), 3,017 (vs), 2,957 (vw), 2,925 (vw), 2,865 (vw), 1,605 (vs), 1,585 (s), 1,566 (s), 1,509 (vs), 1,480 (s), 1,459 (w), 1,410 (vw), 1,379 (vw), 1,358 (vw), 1,319 (w), 1,303 (s), 1,285 (m), 1,260 (vw), 1,184 (s), 1,157 (w), 1,102 (vw), 1,081 (w), 1,063 (vw), 1,019 (m), 1,005 (m), 954 (s), 937 (m), 923 (s), 900 (s), 873 (w), 845 (vw), 830 (w), 793 (vw), 746 (vw), 697 (s), 674 (s), 654 (w), 604 (w), 592 (w), 561 (w), 547 (w), 493 (m), 464 (m), 446 (s), 434 (m), 423 (m), 415 (m), 403 (m).

Data for [La(NHAr^{iPr6})$_2$]·(Et$_2$O) (2La). As for **2Sc**, except the complex was crystallised from the Et_2O supernatant, rather than following extraction with *n*-hexane. **1La** (0.605 g, 0.5 mmol), KC_8 (0.081 g, 0.6 mmol, 1.2 equiv.). Red/brown solution and 1 crop of red/brown planks. Yield = 0.257 g, 45%).

Elemental analysis on $C_{72}H_{100}N_2La\cdot(OC_4H_{10})_n$ for n = 1 calc. (%): C = 75.651, H = 9.189, N = 2.322; for n = 0 calc. (%): C = 76.361, H = 8.900, N = 2.474; found (%): C = 74.69, H = 8.99, N = 2.30.

^{1}H NMR (d_6-benzene, 400.13 MHz, 298 K): δ = 7.92 (br. s, FWHM = 22 Hz), 7.39 (s, FWHM = 16 Hz), 3.03 (br. s, FWHM = 57 Hz), 2.48 (br. s, FWHM = 97 Hz), 1.35 (br. s, FWHM = 10 Hz), 1.32 (br. s, FWHM = 10 Hz).

UV-Vis-NIR (THF): λ_{max} (cm^{-1}; ε) = 816 (12,253, 650).

FT-IR (ATR, microcrystalline): cm^{-1} = 3,479 (vs), 3,386 (vs), 3,317 (vs), 3,073 (vs), 3,041 (vs), 3,015 (vs), 2,955 (vw), 2,925 (w), 2,865 (vw), 2,809 (vs), 2,772 (vs), 1,605 (s), 1,585 (m), 1,568 (s), 1,539 (s), 1,457 (w), 1,408 (vw), 1,379 (vw), 1,358 (vw), 1,319 (vw), 1,289 (m), 1,254 (vw), 1,219 (w), 1,188 (m), 1,167 (m), 1,153 (m), 1,114 (w), 1,100 (w), 1,069 (vw), 1,021 (s), 1,005 (m), 995 (s), 937 (m), 921 (s), 886 (s), 876 (vw), 843 (vw), 830 (vw), 789 (w), 779 (m), 746 (vw), 709 (s), 682 (s), 658 (w), 637 (s), 621 (w), 571 (vw), 547 (w), 514 (m), 491 (m), 481 (w), 467 (w), 444 (m), 432 (s), 419 (w).

*Synthesis of **2M** (M = Sm, Eu, Yb)*

As crystalline divalent halides of the form [MI_2(THF)$_5$] (M = Sm, Eu, Yb) are readily synthesised and desolvated to a form that approximately corresponds to MI_2(THF)$_2$ based upon mass analysis, we have used these in the synthesis of **2M** (M = Sm, Eu, Yb).

Synthesis of [Sm(NHAr^{iPr6})$_2$]·(OC_4H_{10}) (2Sm). Et_2O (50 mL) was added to a pre-cooled (–98°C) stirring mixture of solid SmI_2(THF)$_2$ (0.5482 g, 1 mmol) and KNHAr^{iPr6} (1.082 g, 2 mmol, 2 equiv.) in a glass Schlenk vessel equipped with a PTFE-coated stirrer bar. The mixture was allowed to warm to room temperature and quickly became dark green with a fine white precipitate, presumed to be

KI. After stirring at room temperature overnight (16 hours), the volatiles were removed *in vacuo* (10^{-3} mbar) which left a dark green powder. Et_2O (20 mL) was added and briefly (< 1 min) refluxed with manual agitation to loosen solids from the vessel walls. The dark green solution and fine white solids were allowed to settle before filtration through a glass microfibre filter disc. Concentration of the dark green supernatant to *ca*. 2 mL gave a large quantity of dark green solids which were heated gently into solution and allowed to cool slowly to room temperature, then stored at –30°C for 16 hours to give large dichroic red/green blocks of **2Sm**. Crystals were isolated by decanting the supernatant followed by drying *in vacuo* (10^{-3} mbar, 2 hours). A second crop was obtained in a similar fashion (combined yield = 0.7955 g, 69%).

Elemental analysis on $C_{72}H_{100}N_2Sm{\cdot}(OC_4H_{10})_n$ for n = 1 calc. (%): C = 74.940, H = 9.102, N = 2.300; for n = 0 calc. (%): C = 75.596, H = 8.811, N = 2.449; found (%): C = 74.68, H = 9.19, N = 2.16.

^{1}H NMR (d_6-benzene, 400.13 MHz, 298 K): δ = 34.36 (s, 4H, FWHM = 34 Hz, Tripp-3,5-*H*), 9.06 (s, 2H, SmN(*H*)), 5.96 (d, 2H, $^3J_{HH}$ = 7.4 Hz, Ar-3,5-*H*), 5.74 (s, 4H, Tripp-3,5-*H*), 4.61 (br. s, 12H, FWHM = 121 Hz, Tripp-CH(C*H*$_3$)$_2$), 4.34 (t, 2H, $^3J_{HH}$ = 7.0 Hz, Ar-4-*H*), 3.22 (s, 12H, Tripp-CH(C*H*$_3$)$_2$), 2.93 (m, 6H, Tripp-C*H*(CH$_3$)$_2$), 1.82 (d, 12H, $^3J_{HH}$ = 6.91 Hz, Tripp-CH(C*H*$_3$)$_2$), 1.25 (m, 12H, Tripp-CH(C*H*$_3$)$_2$), –1.59 (br. m, 12H, FWHM = 37 Hz, Tripp-CH(C*H*$_3$)$_2$), –1.80 (br. s, 12H, FWHM = 16 Hz, Tripp-CH(C*H*$_3$)$_2$), –2.54 (d, 2H, $^3J_{HH}$ = 6.1 Hz, Ar-3,5-*H*), –3.24 (br. m, 2H, FWHM = 76, Tripp-C*H*(CH$_3$)$_2$), –6.77 (br. m, 4H, FWHM = 124 Hz, Tripp-C*H*(CH$_3$)$_2$).

^{13}C{^{1}H} NMR (d_6-benzene, 100.62 MHz, 298 K): δ = 168.68, 146.26, 140.22, 114.64, 112.99, 103.53, 101.22, 96.13, 48.15, 45.37, 43.16, 34.77, 25.25, 23.63, 22.51.

Magnetic moment (Evans method, d_6-benzene, 298 K): μ_{eff} = 3.12(1) μB.

UV-Vis-NIR (Et_2O): 811 (12,337, 364), 532 (18,784, 774).

FT-IR (ATR, microcrystalline): cm^{-1} = 3,379 (vs), 3,304 (vs), 3,052 (vs), 3,012 (vs), 2,955 (vw), 2,927 (w), 2,902 (w), 2,865 (w), 2,799 (vs), 2,600 (vs), 1,605 (vs), 1,583 (w), 1,562 (s), 1,459 (w), 1,408 (vw), 1,381 (vw), 1,361 (vw), 1,332 (vw), 1,315 (w), 1,264 (vw), 1,245 (vw), 1,186 (s), 1,167 (m), 1,155 (m), 1,128 (vs), 1,104 (m), 1,067 (vw), 1,003 (w), 935 (m), 923 (m), 904 (s), 876 (vw), 847 (vw), 832 (vw), 802 (s), 793 (m), 779 (m), 744 (vw), 734 (vw), 701 (s), 672 (s), 658 (w), 625 (w), 586 (m), 571 (w), 557 (w), 551 (w), 518 (s), 506 (s), 495 (s), 473 (m), 458 (m), 436 (s), 425 (m), 409 (s).

Data for [Eu(NHAr^{iPr6})$_2$] (2Eu). $EuI_2(THF)_2$ (0.5499 g, 1 mmol), KNHAr^{iPr6} (1.082 g, 2 mmol, 2 equiv.). Orange solution and 2 crop of orange planks. Yield = 0.216 g, 20%).

Elemental analysis on $C_{72}H_{100}N_2Eu$ calc. (%): C = 75.490, H = 8.799, N = 2.445; found (%): C = 75.12, H = 8.87, N = 2.48.

^{1}H NMR (d_6-benzene, 400.13 MHz, 298 K): δ = Only peaks attributable to diamagnetic impurities could be identified.

Magnetic moment (Evans method, d_6-benzene, 298 K): μ_{eff} = 7.37(2) μB.

UV-Vis-NIR (Et_2O): λ_{max} (cm^{-1}; ε) = A broad feature extends from ~625 nm (20,000 cm^{-1}) into the UV region, and beyond our spectral range.

FT-IR (ATR, microcrystalline): cm^{-1} = 3,479 (vw), 3,384 (vw), 2,955 (vs), 2,927 (m), 2,865 (s), 1,583 (m), 1,564 (vw), 1,459 (s), 1,408 (vs), 1,381 (s), 1,361 (s), 1,332 (s), 1,315 (m), 1,264 (vs), 1,245 (s), 1,186 (vw), 1,167 (vw), 1,153 (vw), 1,104 (m), 1,067 (vs), 1,021 (vw), 1,003 (s), 937 (w), 923 (w), 906 (vw), 873 (s), 847 (s), 832 (s), 802 (w), 793 (w), 779 (m), 744 (vs), 734 (vs), 672 (vw), 658 (m), 625 (m), 588 (w), 569 (m), 551 (m), 524 (vw), 512 (vw), 491 (vw), 483 (w), 473 (w), 460 (w), 450 (w), 440 (m), 423 (m), 419 (vs), 403 (w).

Data for [Yb(NHAr^{iPr6})$_2$] (2Yb). $YbI_2(THF)_2$ (0.5708 g, 1 mmol), KNHAr^{iPr6} (1.082 g, 2 mmol, 2 equiv.). Dark green/blue solution and 2 crop of blue blocks. Yield = 0.9596 g, 82%).

Elemental analysis on $C_{72}H_{100}N_2Yb$ calc. (%): C = 74.125, H = 8.640, N = 2.401; found (%): C = 73.92, H = 9.19, N = 2.18.

^{1}H NMR (d_6-benzene, 400.13 MHz, 298 K): δ = 7.30 (s, 4H, Tripp-3,5-*H*), 7.15 (d, $^3J_{HH}$ = 6.6 Hz, 2H, Ar-3,5-*H*), 7.02 (s, 4H, Tripp-3,5-*H*), 6.86 (d, $^3J_{HH}$ = 7.2 Hz, 2H, Ar-3,5-*H*), 6.63, (2 × t, $^3J_{HH}$ = 7.3 Hz, 6.8 Hz, 2H, Ar-4-*H*), 3.55 (s, 2H, YbN(*H*)), 3.10 (apparent d. hept, 8H, Tripp-2,6-C*H*(CH_3)$_2$), 2.90 (apparent d. hept, 8H, Tripp-2,6-C*H*(CH_3)$_2$), 1.43 (d, $^3J_{HH}$ = 6.4 Hz, 12H, Tripp-2,6-CH(C*H*$_3$)$_2$), 1.39 (d, $^3J_{HH}$ = 6.4 Hz, 12H, Tripp-2,6-CH(C*H*$_3$)$_2$), 1.30 (d, $^3J_{HH}$ = 6.4 Hz, 12H, Tripp-2,6-CH(C*H*$_3$)$_2$), 1.15 (d, $^3J_{HH}$ = 6.4 Hz, 12H, Tripp-2,6-CH(C*H*$_3$)$_2$), 1.01 (d, $^3J_{HH}$ = 7.4 Hz, 12H, Tripp-4-CH(C*H*$_3$)$_2$), 0.98 (d, $^3J_{HH}$ = 7.4 Hz, 12H, Tripp-4-CH(C*H*$_3$)$_2$).

$^{13}C\{^{1}H\}$ NMR (d_6-benzene, 100.62 MHz, 298 K): δ = 160.71 (s, Ar-<u>*C*</u>N(H)-Yb), 150.65 (Tripp-2,6-<u>*C*</u>), 148.99 (Tripp-*ipso*-<u>*C*</u>), 148.45 (Tripp-2,6-<u>*C*</u>), 147.66 (Tripp-*ipso*-<u>*C*</u>), 143.94 (Ar-2,6-<u>*C*</u>), 136.86 (Tripp-4-<u>*C*</u>), 131.42 (Tripp-3,5-<u>*C*</u>H), 127.89 (Ar-<u>*C*</u> quat.), 126.20 (Ar-3,5-<u>*C*</u>H), 122.00 (Ar-3,5-<u>*C*</u>H), 120.82 (Tripp-3,5-<u>*C*</u>H), 110.05 (Ar-4-<u>*C*</u>H), 35.13 (Tripp-4-<u>*C*</u>H(CH_3)$_2$), 33.08 (Tripp-4-<u>*C*</u>H(CH_3)$_2$), 25.81 (Tripp-4-CH(<u>*C*</u>H_3)$_2$), 25.21 (Tripp-2,6-CH(<u>*C*</u>H_3)$_2$), 24.93 (Tripp-2,6-CH(<u>*C*</u>H_3)$_2$), 24.74 (Tripp-2,6-CH(<u>*C*</u>H_3)$_2$), 23.62 (Tripp-2,6-CH(<u>*C*</u>H_3)$_2$) – the remaining two Tripp-*i*Pr ^{13}C peaks could not be conclusively determined, though we note that several of the listed peaks are somewhat broader than expected, and may be overlapping sets of resonances.

^{171}Yb-^{1}H HMBC NMR (d_6-benzene, 69.98 MHz / 399.92 MHz, 298 K): δ = –83.02 / 3.53 (<u>*Yb*</u>N(<u>*H*</u>)), –83.02 / 7.02 (<u>*Yb*</u>-Tripp-3,5-<u>*H*</u>).

UV-Vis-NIR (THF): λ_{max} (cm^{-1}; ε) = 536 (18,643, 387).

FT-IR (ATR, microcrystalline): cm^{-1} = 3,320 (vs), 3,055 (vs), 2,957 (vw), 2,925 (vw), 2,865 (w), 2,803 (vs), 1,605 (vs), 1,583 (w), 1,564 (s), 1,556 (s), 1,457 (w), 1,408 (vw), 1,381 (vw), 1,361 (vw), 1,332 (vw), 1,315 (m), 1,276 (vw), 1,264 (vw), 1,241 (w), 1,186 (vs), 1,165 (m), 1,153 (m), 1,126 (vs), 1,100 (w), 1,069 (vw), 1,001 (w), 956 (s), 931 (m), 904 (vs), 886 (w), 873 (vw), 847 (vw), 832 (vw), 799 (s), 777 (m), 736 (vw), 672 (s), 658 (w), 629 (w), 580 (m), 567 (w), 549 (m), 530 (s), 520 (s), 508 (s), 495 (s), 485 (s), 471 (m), 458 (m), 446 (s), 423 (m), 411 (m).

Data for [Tm(NHAr^{i}Pr$_6$)$_2$]·(Et$_2$O) (2Tm). As for **2Sc**, except the complex was crystallised from the Et_2O supernatant, rather than following extraction with *n*-hexane. **1Tm** (0.6445 g, 0.5 mmol), KC_8 (0.083 g, 0.6 mmol, 1.2 equiv.). Red solution and 1 crop of red planks. Yield = 0.376 g, 65%).

Elemental analysis on $C_{72}H_{100}N_2Tm\cdot(OC_4H_{10})_n$ for n = 1 calc. (%): C = 73.814, H = 8.966, N = 2.265; for n = 0 calc. (%): C = 74.388, H = 8.670, N = 2.410; found (%): C = 70.94, H = 8.59, N = 2.19..

^{1}H NMR (d_6-benzene, 400.13 MHz, 298 K): δ = 26.42 (br. s, FWHM = 90 Hz), 3.28 (br. s, FWHM = 56 Hz), 3.00 (br. s, FWHM = 102 Hz), 1.26 (br. s, FWHM = 34 Hz), –2.00 (br. s, FWHM = 63 Hz), –15.43 (br. s, FWHM = 56 Hz), –47.67 (br. s, FWHM = 150 Hz).

Magnetic moment (Evans method, d_6-benzene, 298 K): μ_{eff} = 6.06(2) μB.

UV-Vis-NIR (THF): λ_{max} (cm^{-1}; ε) = 891 (11,224, 207), *ca*. 386 (25,882, 1,305).

FT-IR (ATR, microcrystalline): cm^{-1} = 3,482 (vs), 3,384 (vs), 2,955 (vw), 2,927 (vw), 2,900 (vw), 2,867 (vw), 2,749 (vs), 2,717 (vs), 1,603 (vs), 1,585 (s), 1,566 (vs), 1,541 (vs), 1,461 (vw), 1,441 (w), 1,410 (vw), 1,383 (vw), 1,363 (vw), 1,332 (w), 1,317 (m), 1,262 (vw), 1,233 (w), 1,184 (vs), 1,167 (m), 1,153 (s), 1,106 (w), 1,069 (vw), 1,036 (s), 1,003 (w), 939 (m), 921 (s), 876 (vw), 847 (vw), 830 (w), 795 (m), 777 (s), 744 (vw), 699 (s), 674 (s), 656 (w), 633 (w), 582 (m), 569 (w), 559 (m), 551 (m), 526 (s), 510 (s), 497 (s), 479 (m), 467 (m), 450 (m), 438 (m), 423 (w), 407 (w).

S2. Crystallography

General considerations

Data for **1La**[b], **1Y**, **1Yb**, **2Sm**, **2Yb**, and **3** were collected using a Rigaku XtaLAB Synergy DW diffractometer, equipped with a PhotonJet Cu Kα radiation source (λ = 1.54184 Å), using a 4-circle κ goniometer, a HyPix-6000HE hybrid pixel array detector operating in shutterless mode and an Oxford Cryosystems Cryostream 800 nitrogen flow gas system at a temperature of 100K. Data for **1Sc**, **1Tm**, **2Sc**, **2Y**, **2La**, **2Eu**, and **2Tm** were collected using a Rigaku FR-X DW diffractometer, equipped with an FR-X high-intensity rotating anode Cu Kα radiation source (λ = 1.54184 Å) and VariMAXTM microfocus optics, using an AFC-11 4-circle κ goniometer, a HyPix-6000HE hybrid pixel array detector operating in shutterless mode and an Oxford Cryosystems Cryostream 800 plus nitrogen flow gas system at a temperature of 100K. Crystals of **1La** were examined using an Oxford Diffraction Supernova diffractometer, furnished with a CCD area detector and a mirror-monochromated Mo Kα radiation (λ = 0.71073 Å) at a temperature of 100 K. All data were integrated and reduced using Rigaku Oxford Diffraction CryAlisPro v1.171.42. Intensities were integrated from data recorded from ω, or ω and φ rotation at the frame width and exposure times outlined in **Table S2**. The crystal data for all complexes is outlined in **Table S3** to **Table S7**.

Table S2. Data collection parameters and CCDC reference codes for all structures herein.

	Formula	Frame width (°)	Exposure time (s)	CCDC ref.
**1Sc**	$[Sc(NHAr^{iPr6})_2(I)]\cdot(C_7H_8)_2$	0.5	1 / 2	2266263
**1Y**	$[Y(NHAr^{iPr6})_2(I)]\cdot(C_7H_8)_2$	0.5	10	2266261
**1La**	$[La(NHAr^{iPr6})_2(I)]\cdot(C_7H_8)_2$	1.0	20.41	2266235
**1Tm**	$[Tm(NHAr^{iPr6})_2(I)]\cdot(C_7H_8)_2$	0.5	0.5 / 1	2266255
**1Yb**	$[Yb(NHAr^{iPr6})_2(I)]\cdot(C_7H_8)_2$	0.5	13.43	2266257
**1Y** $^{\beta}$	$[Y(NHAr^{iPr6})_2(I)]\cdot{}^1/_2(C_6H_{14})$	0.5	8 / 30	2295306
**1La** $^{\beta}$	$[La(NHAr^{iPr6})_2(I)]\cdot(C_6H_{14})$	0.5	20	2295307
**2Sc**	$[Sc(NHAr^{iPr6})_2]$	0.5	2 / 7	2266264
**2Y**	$[Y(NHAr^{iPr6})_2(I)]\cdot(OC_4H_{10})$	0.5	3 / 12	2266262
**2La**	$[La(NHAr^{iPr6})_2(I)]\cdot(OC_4H_{10})$	0.5	0.5 / 1	2266236
**2Sm**	$[Sm(NHAr^{iPr6})_2(I)]\cdot(OC_4H_{10})$	0.5	1.05 / 4.19	2266243
**2Eu**	$[Eu(NHAr^{iPr6})_2(I)]$	0.5	1.5 / 3	2266244
**2Tm**	$[Tm(NHAr^{iPr6})_2(I)]\cdot(OC_4H_{10})$	0.5	1 / 2	2266256
**2Yb**	$[Yb(NHAr^{iPr6})_2(I)]$	0.6	3.5 / 13.5	2266258
**3**	HN=C_6H_3-2,6-(2,4,6-*i*Pr_3)-4-{HN-C_6H_3-2,6-(2,4,6-*i*Pr_3)$_2$	0.5	5 / 20	2282041

CrysAlisPro[6] was used for final unit cell determination and parameters were refined from the observed positions of all strong reflections in each data set. An analytical absorption correction was applied.[6] The Olex2[7] GUI was used for structure solution and refinement utilizing the ShelX software packages.[8, 9] The structures were solved using ShelXT[8]; the datasets were refined by ShelXL[9] using full-matrix least-squares on all unique F^2 values, with anisotropic displacement parameters for all non-hydrogen atoms, and with constrained riding hydrogen geometries; U_{iso}(H) was set at 1.2 (1.5 for methyl groups if applicable) times *U*eq of the parent atom. The largest features in final difference syntheses were close to heavy atoms and were of no chemical significance. Olex2[7] combined with POV-Ray,[10] and Gimp[11] were employed for molecular graphics. The following CCDC references contain the supplementary crystal data for this article: **1Sc** (2266263), **1Y** (2266261), **1La** (2266235), **1Tm** (2266255), **1Yb** (2266257), **1Y** $^{\beta}$ (2295306), **1La**$^{\beta}$ (2295307), **2Sc** (2266264), **2Y** (2266262), **2La** (2266236), **2Sm** (2266243), **2Eu** (2266244), **2Tm** (2266256), **2Yb** (2266258), and

3 (2282041).[**,12] These data can be obtained free of charge from the Cambridge Crystallographic Data Centre via www.ccdc.cam.ac.uk/data_request/cif.

The combined error from two individual metrics that have their own associated errors (estimated standard deviation, or standard uncertainty used interchangeably here) can be calculated as the root of the sum of the square of each error (**Equation S2**). This is not strictly appropriate for combining more than two individual errors.[13]

Equation S2. $$\sigma_{tot} = \sqrt{{\sigma_1}^2 + {\sigma_2}^2}$$

The combined error for the numerical average for multiple (independent) bond lengths, such as the five independent M–C bonds in an M–($\eta^5C_5H_5$) complex, is calculated using the alternate weighted standard deviation from Parsons and Clegg (**Equation S3**).[13]

Equation S3. $$\sigma_{tot} = 1/\sqrt{\sum_{1\to n} W_n} \qquad W_n = 1/{\sigma_n}^2$$

[**] Complex **3** has previously been reported (ETOTIT), see ref. [14].

Table S3. Crystallographic data for **1Sc**, **1Y**, and **1Y** $^{\beta}$.

	**1Sc**	**1Y**	**1Y** $^{\beta}$
Identification code	rcapg37	ccapg101	ccapg64
Formula	$C_{72}H_{100}IN_2Sc\cdot(C_7H_8)_2$	$C_{72}H_{100}IN_2Y\cdot(C_7H_8)_2$	$C_{72}H_{100}IN_2Y\cdot{}^1/_2(C_6H_{14})$
Fw	1349.66	1393.61	1252.43
Temperature / K	100.00(10)	100.00(10)	100.00(10)
Crystal system	triclinic	triclinic	monoclinic
Space group	$P\bar{1}$	$P\bar{1}$	$P2_1/n$
a / Å	13.7201(3)	13.8601(4)	14.79590(10)
b / Å	15.2477(4)	15.1451(4)	23.5264(2)
c / Å	19.1530(4)	19.2635(5)	19.43130(10)
α / °	89.686(2)	90.423(2)	90
β / °	70.204(2)	110.358(2)	97.2910(10)
γ / °	87.286(2)	92.402(2)	90
Volume / Å^3	3765.56(16)	3786.70(18)	6709.23(8)
Z	2	2	4
ρ_{calcg} / cm^3	1.190	1.222	1.240
μ / mm^{-1}	4.374	1.222	5.137
F(000)	1440	1476	2652.0
Crystal size / mm^3	0.194 × 0.14 × 0.119	0.488 × 0.177 × 0.157	0.086 x 0.039 x 0.025
Radiation	Cu Kα (λ = 1.54184)	Mo Kα (λ = 0.71073)	Cu Kα (λ = 1.54184)
2Θ range / °	5.804 to 152.172	4.51 to 58.262	5.928 to 160.248
Index ranges	–17 ≤ h ≤ 17, –19 ≤ k ≤ 16, –16 ≤ l ≤ 24	–18 ≤ h ≤ 18, –20 ≤ k ≤ 20, –25 ≤ l ≤ 26	–10 ≤ h ≤ 18, –29 ≤ k ≤ 29, –24 ≤ l ≤ 24
No. reflections	42340	54617	55670
Unique reflections	15011 [R_{int} = 0.0225, R_σ = 0.0241]	20193 [R_{int} = 0.0407, R_σ = 0.0584]	14218 [R_{int} = 0.0424, R_σ = 0.0349]
Data / restraints / parameters	15011 / 319 / 894	20193 / 88 / 841	14218 / 53 / 757
GOOF on F^2	1.143	1.007	1.103
Final R indexes [I ≥ 2σ (I)][a]	R_1 = 0.0319, wR_2 = 0.0883	R_1 = 0.0393, wR_2 = 0.0807	R_1 = 0.0355, wR_2 = 0.0913
Final R indexes [all data]	R_1 = 0.0328, wR_2 = 0.0889	R_1 = 0.0663, wR_2 = 0.0876	R_1 = 0.0404, wR_2 = 0.0935
Largest diff. (peak / hole) / e Å^{-3}	0.52 / –1.41	0.76 / –0.44	0.76 / –1.32

[a] $R = \sum||F_O| - |F_C||/\sum|F_O|$; $R_W = [\sum w(F_O^2 - F_C^2)^2/\sum w(F_O^2)^2]^{0.5}$; $S = [\sum w(F_O^2 - F_C^2)^2/(\text{no. data} - \text{no. params})]^{0.5}$ for all data.

Table S4. Crystallographic data for **1La**, **1La** $^{\beta}$, and **1Tm**.

	**1La**	**1La** $^{\beta}$	**1Tm**
Identification code	acapg2	ccapg116	lcapg28
Formula	$C_{72}H_{100}IN_2La\cdot(C_7H_8)_2$	$C_{72}H_{100}IN_{2La}\cdot(C_6H_{14})$	$C_{72}H_{100}IN_2Tm\cdot(C_7H_8)_2$
Fw	1443.61	1345.52	1473.63
Temperature / K	100.00(10)	100.00(10)	100.00(10)
Crystal system	triclinic	monoclinic	triclinic
Space group	$P\bar{1}$	$P2_1$/c	$P\bar{1}$
a / Å	14.2175(7)	14.2996(2)	13.8471(3)
b / Å	14.8730(5)	15.6187(2)	15.1748(3)
c / Å	18.6528(7)	32.5420(5)	19.2531(3)
α / °	91.655(3)	90	90.1824(14)
β / °	103.113(4)	97.0977(13)	110.2896(17)
γ / °	95.397(3)	90	92.5177(16)
Volume / Å^3	3819.2(3)	7212.30(18)	3790.18(13)
Z	2	4	2
ρ_{calcg} / cm^3	1.255	1.239	1.291
μ / mm^{-1}	1.007	1.061	5.707
F(000)	1512	2824	1536
Crystal size / mm^3	0.191 × 0.133 × 0.088	0.165 × 0.123 × 0.099	0.15 × 0.118 × 0.033
Radiation	Mo Kα (λ = 0.71073)	Mo Kα (λ = 0.71073)	Cu Kα (λ = 1.54184)
2Θ range / °	6.652 to 58.194	4.428 to 64.828	4.894 to 152.408
Index ranges	–17 ≤ h ≤ 19, –20 ≤ k ≤ 18, –24 ≤ l ≤ 24	-20 ≤ h ≤ 19, -18 ≤ k ≤ 23, -47 ≤ l ≤ 47	–17 ≤ h ≤ 17, –18 ≤ k ≤ 19, –24 ≤ l ≤ 24
No. reflections	32806	89261	56904
Unique reflections	17374 [R_{int} = 0.0360, R_σ = 0.0649]	21805 [R_{int}= 0.0376, R_σ = 0.0373]	15108 [R_{int} = 0.0529, R_σ = 0.0428]
Data / restraints / parameters	17374 / 1278 / 1038	21805 / 1 / 771	15108 / 313 / 893
GOOF on F^2	1.024	1.045	1.108
Final R indexes [I ≥ 2σ (I)][a]	R_1 = 0.0381, wR_2 = 0.0710	R_1 = 0.0312, wR_2= 0.0670	R_1 = 0.0373, wR_2 = 0.1018
Final R indexes [all data]	R_1 = 0.0619, wR_2 = 0.0804	R_1 = 0.0442, wR_2= 0.0705	R_1 = 0.0398, wR_2 = 0.1033
Largest diff. (peak / hole) / e Å^{-3}	1.03 / –0.77	0.68 / –0.58	1.13 / –2.31

[a] $R = \sum||F_O| - |F_C||/\sum|F_O|$; $R_W = [\sum w(F_O^2 - F_C^2)^2/\sum w(F_O^2)^2]^{0.5}$; $S = [\sum w(F_O^2 - F_C^2)^2/(\text{no. data} - \text{no. params})]^{0.5}$ for all data.

Table S5. Crystallographic data for **1Yb**, **2Sc**, and **2Y**.

	**1Yb**	**2Sc**	**2Y**
Identification code	ccapg114	rcapg37	ccapg101
Formula	$C_{72}H_{100}IN_2Yb\cdot(C_7H_8)_2$	$C_{72}H_{100}N_2Sc$	$C_{72}H_{100}N_2Y\cdot(OC_4H_{10})$
Fw	1477.74	1038.49	1156.56
Temperature / K	100.00(10)	100.00(10)	100.00(10)
Crystal system	triclinic	triclinic	monoclinic
Space group	$P\bar{1}$	$P\bar{1}$	$C2/c$
a / Å	13.8450(3)	12.8855(3)	18.0918(3)
b / Å	15.1906(3)	13.5895(4)	16.7202(3)
c / Å	19.2184(3)	20.8228(4)	23.1781(4)
α / °	90.2040(15)	75.703(2)	90
β / °	110.2846(16)	89.2794(16)	107.8036(19)
γ / °	92.5150(16)	62.710(2)	90
Volume / $Å^3$	3786.81(13)	3118.52(14)	6675.6(2)
Z	2	2	4
ρ_{calcg} / cm^3	1.296	1.106	1.151
μ / mm^{-1}	1.686	1.307	1.549
F(000)	1538	1134	2508
Crystal size / mm^3	0.302 × 0.122 × 0.084	0.1 × 0.093 × 0.042	0.107 × 0.068 × 0.025
Radiation	Mo Kα (λ = 0.71073)	Cu Kα (λ = 1.54184)	Cu Kα (λ = 1.54184)
2Θ range / °	6.09 to 58.26	4.41 to 152.218	7.368 to 152.742
Index ranges	–18 ≤ h ≤ 18, –20 ≤ k ≤ 20, –26 ≤ l ≤ 26	–16 ≤ h ≤ 14, –17 ≤ k ≤ 16, –25 ≤ l ≤ 26	–22 ≤ h ≤ 22, –19 ≤ k ≤ 20, –29 ≤ l ≤ 28
No. reflections	62723	67098	34382
Unique reflections	20369 [R_{int} = 0.0366, R_σ = 0.0426]	12749 [R_{int} = 0.0360, R_σ = 0.0284]	6910 [R_{int}= 0.0266, R_σ = 0.0234]
Data / restraints / parameters	20369 / 313 / 893	12749 / 872 / 843	6910 / 0 / 378
GOOF on F^2	1.019	1.116	1.091
Final R indexes [I ≥ 2σ (I)][a]	R_1 = 0.0295, wR_2 = 0.0605	R_1 = 0.0391, wR_2= 0.1046	R_1 = 0.0330, wR_2 = 0.0869
Final R indexes [all data]	R_1 = 0.0418, wR_2 = 0.0637	R_1 = 0.0420, wR_2= 0.1063	R_1 = 0.0349, wR_2= 0.0879
Largest diff. (peak / hole) / e $Å^{-3}$	0.92 / –0.62	0.31 / –0.50	0.54 / –0.65

[a] $R = \sum||F_O| - |F_C||/\sum|F_O|$; $R_W = [\sum w(F_O^2 - F_C^2)^2/\sum w(F_O^2)^2]^{0.5}$; $S = [\sum w(F_O^2 - F_C^2)^2/(\text{no. data} - \text{no. params})]^{0.5}$ for all data.

Table S6. Crystallographic data for **2La**, **2Sm**, and **2Eu**.

	**2La**	**2Sm**	**2Eu**
Identification code	acapg2	ccapg119	lcapg36
Formula	$C_{72}H_{100}N_2La\cdot(OC_4H_{10})$	$C_{72}H_{100}N_2Sm\cdot(OC_4H_{10})$	$C_{72}H_{100}N_2Eu\cdot(OC_4H_{10})_{1.5}$
Fw	1206.56	1218.00	1256.67
Temperature / K	100.00(10)	100.00(10)	100.00(10)
Crystal system	triclinic	monoclinic	monoclinic
Space group	$P\bar{1}$	$P2_1/c$	$C2$
a / Å	13.2755(2)	13.35080(10)	22.4535(3)
b / Å	24.2918(3)	24.2551(2)	16.0659(2)
c / Å	21.4153(3)	21.4353(2)	20.2346(2)
α / °	90	90	90
β / °	96.7512(15)	96.7280(10)	106.3783(13)
γ / °	90	90	90
Volume / $Å^3$	6858.27(18)	6839.48(10)	7003.18(17)
Z	4	4	4
ρ_{calcg} / cm^3	1.169	1.174	1.192
μ / mm^{-1}	5.124	6.705	6.733
F(000)	2580.0	2600.0	2688.0
Crystal size / mm^3	0.28 × 0.169 × 0.092	0.28 × 0.169 × 0.092	0.101 × 0.066 × 0.066
Radiation	Cu Kα (λ = 1.54184)	Cu Kα (λ = 1.54184)	Cu Kα (λ = 1.54184)
2Θ range / °	6.704 to 151.474	5.524 to 160.018	6.864 to 151.928
Index ranges	$-16 \leq h \leq 16$, $-28 \leq k \leq 30$, $-26 \leq l \leq 26$	$-16 \leq h \leq 16$, $-25 \leq k \leq 30$, $-27 \leq l \leq 27$	$-28 \leq h \leq 26$, $-20 \leq k \leq 19$, $-24 \leq l \leq 25$
No. reflections	65139	58015	26464
Unique reflections	14067 [R_{int} = 0.0310, R_σ = 0.0273]	14568 [R_{int} = 0.0402, R_σ = 0.0360]	11576 [R_{int} = 0.0318, R_σ = 0.0462]
Data / restraints / parameters	14067 / 1152 / 960	14568 / 63 / 772	11576 / 184 / 843
GOOF on F^2	1.128	1.083	1.063
Final R indexes [$I \geq 2\sigma$ (I)][a]	R_1 = 0.0451, wR_2 = 0.1151	R_1 = 0.0376, wR_2 = 0.0920	R_1 = 0.0399, wR_2 = 0.1034
Final R indexes [all data]	R_1 = 0.0477, wR_2 = 0.1164	R_1 = 0.0454, wR_2 = 0.0954	R_1 = 0.0422, wR_2 = 0.1046
Largest diff. (peak / hole) / e $Å^{-3}$	1.35 / –1.25	0.48 / –0.93	1.50 / –1.20

[a] $R = \sum||F_O| - |F_C||/\sum|F_O|$; $R_W = [\sum w(F_O^2 - F_C^2)^2/\sum w(F_O^2)^2]^{0.5}$; $S = [\sum w(F_O^2 - F_C^2)^2/(\text{no. data} - \text{no. params})]^{0.5}$ for all data.

Table S7. Crystallographic data for **2Tm**, **2Yb**, and **3**.

	**2Tm**	**2Yb**	**3**
Identification code	lcapg33	ccapg88	ccapg117
Formula	$C_{72}H_{100}N_2Tm\cdot(OC_4H_{10})$	$C_{72}H_{100}N_2Yb\cdot(OC_4H_{10})_{1.5}$	$C_{72}H_{100}N_2$
Fw	1236.58	1277.75	993.53
Temperature / K	100.00(10)	100.00(10)	100.00(10)
Crystal system	monoclinic	monoclinic	monoclinic
Space group	$P2_1$/c	*C*2	$P2_1$/n
a / Å	13.1863(2)	22.4353(2)	11.91000(10)
b / Å	24.2869(3)	16.06930(10)	18.09160(10)
c / Å	21.3806(3)	20.02640(10)	29.8982(2)
	90	90	90
β / °	95.8291(15)	106.4940(10)	95.9430(10)
	90	90	90
Volume / $Å^3$	6811.84(18)	6922.80(9)	6407.57(8)
Z	4	4	4
ρ_{calcg} / cm^3	1.206	1.226	1.030
μ / mm^{-1}	2.751	2.825	0.430
F(000)	2628	2716	2184
Crystal size / mm^3	0.12 × 0.075 × 0.029	0.083 x 0.076 x 0.061	0.33 × 0.095 × 0.065
Radiation	Cu Kα (λ = 1.54184)	Cu Kα (λ = 1.54184)	Cu Kα (λ = 1.54184)
2Θ range / °	5.524 to 152.89	4.602 to 160.082	5.718 to 160.83
Index ranges	$-15 \le h \le 16$, $-27 \le k \le 29$, $-26 \le l \le 26$	$-28 \le h \le 28$, $-20 \le k \le 20$, $-25 \le l \le 25$	$-15 \le h \le 15$, $-23 \le k \le 23$, $-37 \le l \le 38$
No. reflections	63671	56201	28090
Unique reflections	14064 [R_{int} = 0.0425, R_σ = 0.0400]	14716 [R_{int} = 0.0385, R_σ = 0.318]	28090 [R_{int} = N/A, R_σ = 0.0226]
Data / restraints / parameters	14064 / 240 / 850	14716 / 342 / 867	28090 / 4607 / 1080
GOOF on F^2	1.074	1.130	1.063
Final R indexes [$I \ge 2\sigma(I)$][a]	R_1 = 0.0318, wR_2 = 0.0801	R_1 = 0.0413, wR_2 = 0.1016	R_1 = 0.0947, wR_2 = 0.2741
Final R indexes [all data]	R_1 = 0.0362, wR_2 = 0.0823	R_1 = 0.0427, wR_2 = 0.1022	R_1 = 0.1081, wR_2 = 0.2875
Largest diff. (peak / hole) / e $Å^{-3}$	0.75 / –0.88	1.71 / –2.06	0.27 / –0.29

[a] $R = \sum||F_O| - |F_C||/\sum|F_O|$; $R_W = [\sum w(F_O^2 - F_C^2)^2/\sum w(F_O^2)^2]^{0.5}$; $S = [\sum w(F_O^2 - F_C^2)^2/(\text{no. data} - \text{no. params})]^{0.5}$ for all data.

S3. Molecular structures

*Complexes **1M** (M = Sc, Y, La, Tm, Yb)*

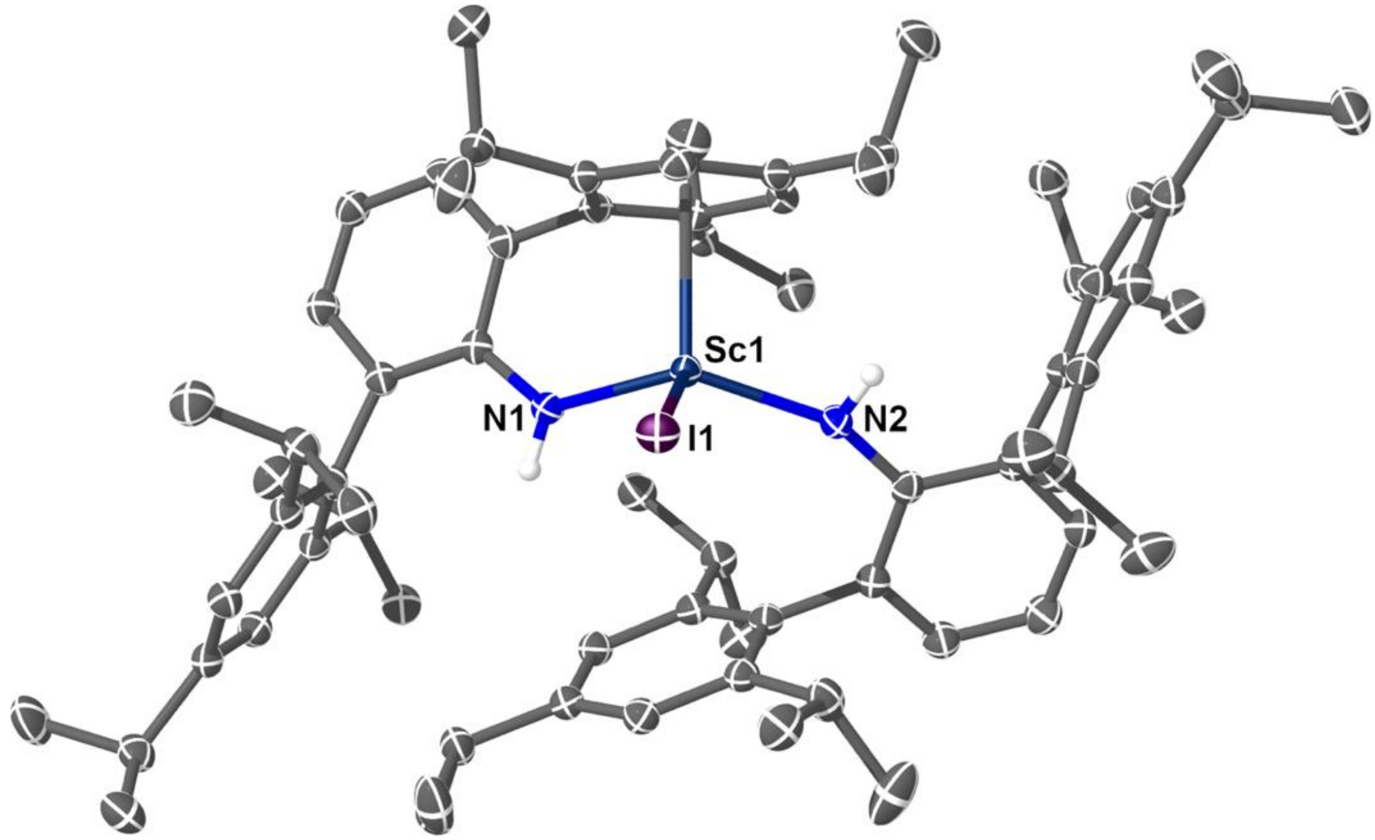

Figure S3. Molecular structure of **1Sc**. Ellipsoids set at 50% probability and H-atoms removed for clarity (operations: X, Y, Z).

Sc(1)–I(1) = 2.7666(3) Å; Sc(1)–N(1) = 2.0909(15) Å; Sc(1)–N(2) = 2.0643 Å; Sc(1)–C_{range} = 2.6282(17)–2.8116(18) Å; Sc(1)–$C_{6\text{-centroid}}$ = 2.3391(8) Å; N(1)–Sc(1)–N(2) = 130.51(6) °.

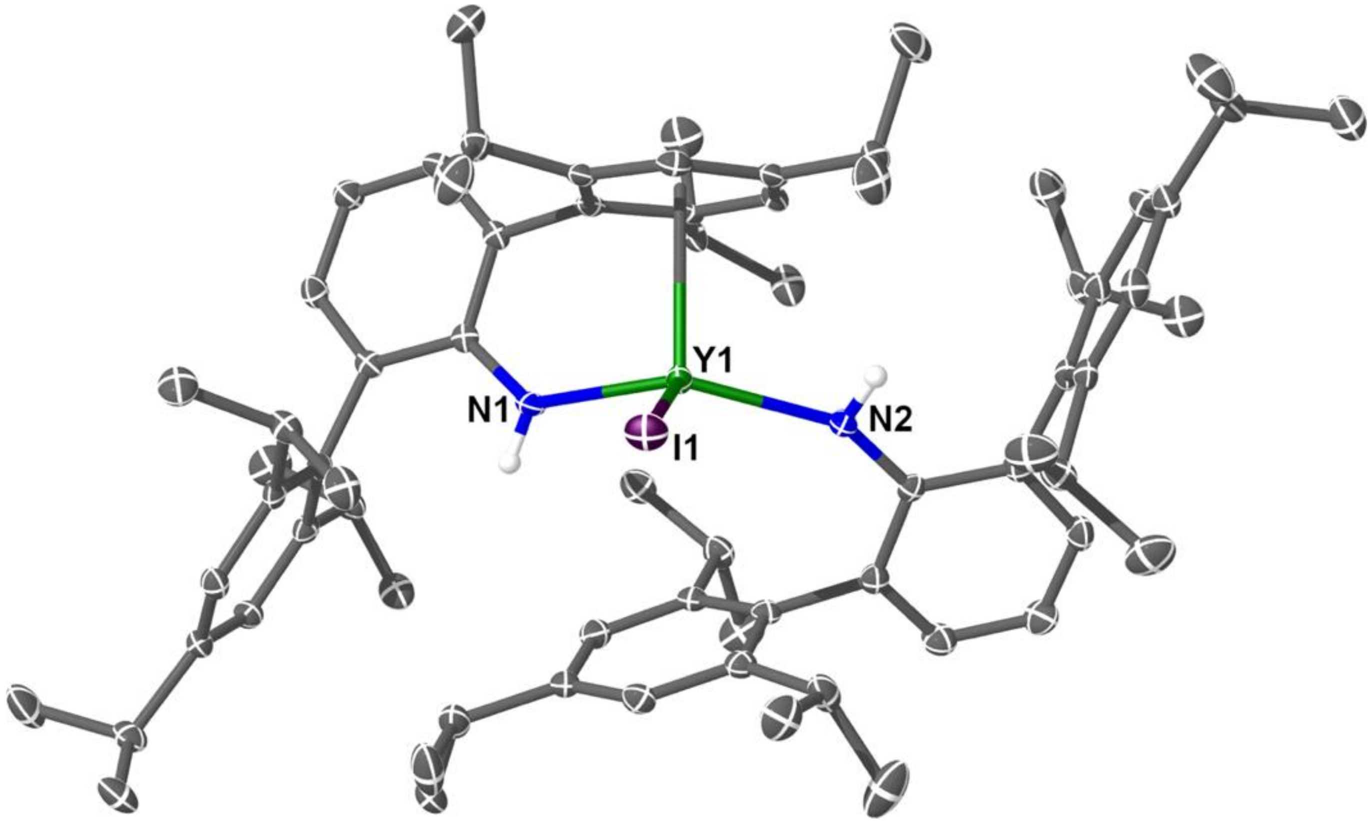

Figure S4. Molecular structure of **1Y**. Ellipsoids set at 50% probability and H-atoms removed for clarity (operations: X, Y, Z).

Y(1)–I(1) = 2.9108(2) Å; Y(1)–N(1) = 2.2452(17) Å; Y(1)–N(2) = 2.2167(17) Å; Y(1)–C_{range} = 2.7912(19)–2.919(2) Å; Y(1)–$C_{6\text{-centroid}}$ = 2.4949(9) Å; N(1)–Y(1)–N(2) = 135.67(6)°.

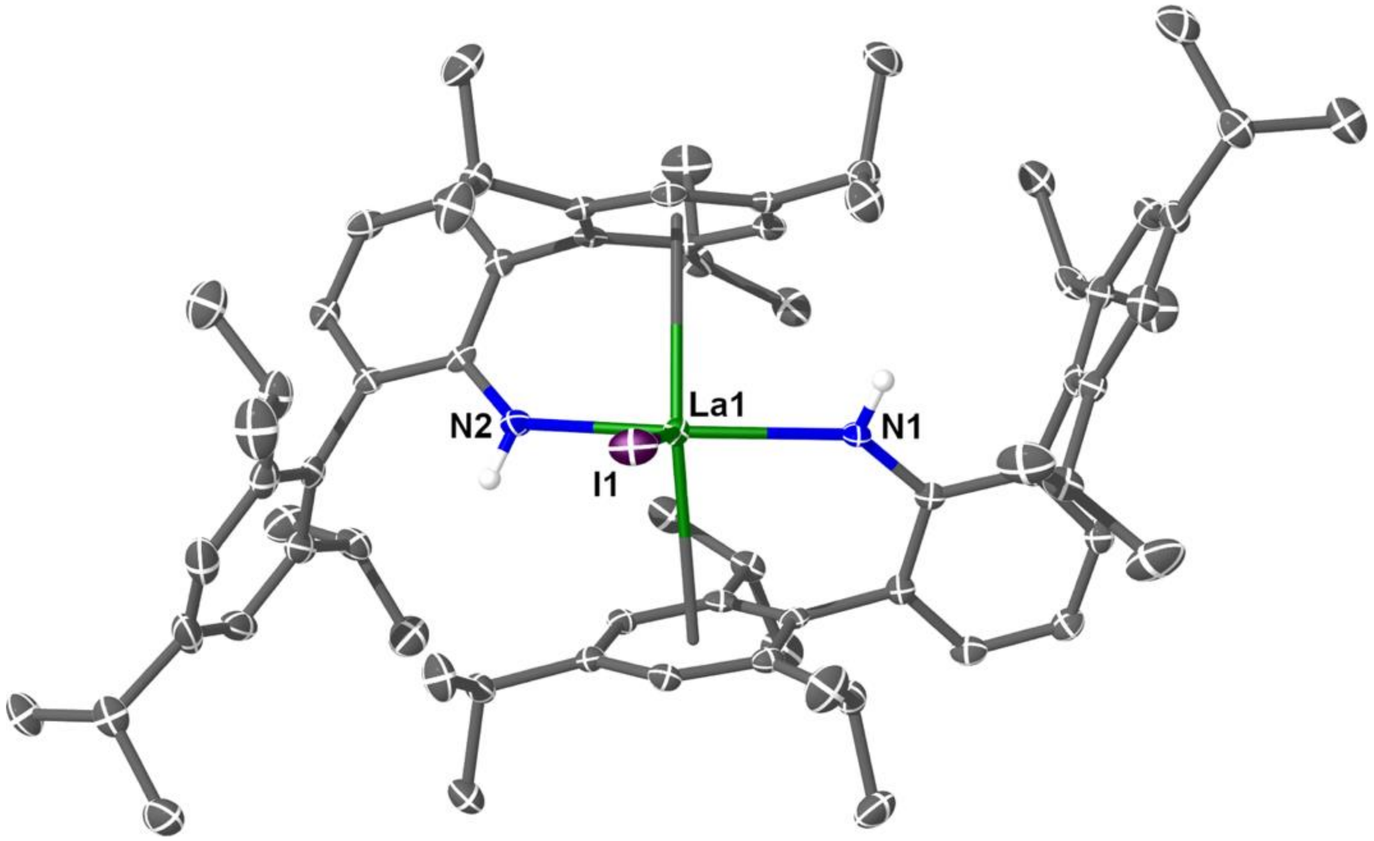

Figure S5. Molecular structure of **1La**. Ellipsoids set at 50% probability, H-atoms and two toluene molecules of crystallization removed for clarity (operations: X, Y, Z).

La(1)–I(1) = 3.0866(3) Å; La(1)–N(1) = 2.402(2) Å; La(1)–N(2) = 2.399(2) Å; La(1)–$C_{6\text{-range}}$ = 3.132(3)–3.249(3) Å; La(1)–$C_{6\text{-range}}$ = 3.113(3)–3.270(3) Å; La(1)–$C_{6\text{-centroid}}$ = 2.8714(12) Å; La(1)–$C_{6\text{-centroid}}$ = 2.8783(12) Å; N(1)–La(1)–N(2) = 148.31(8)°; $C_{6\text{-centroid}}$–La(1)–$C_{6\text{-centroid}}$ = 155.06(4)°

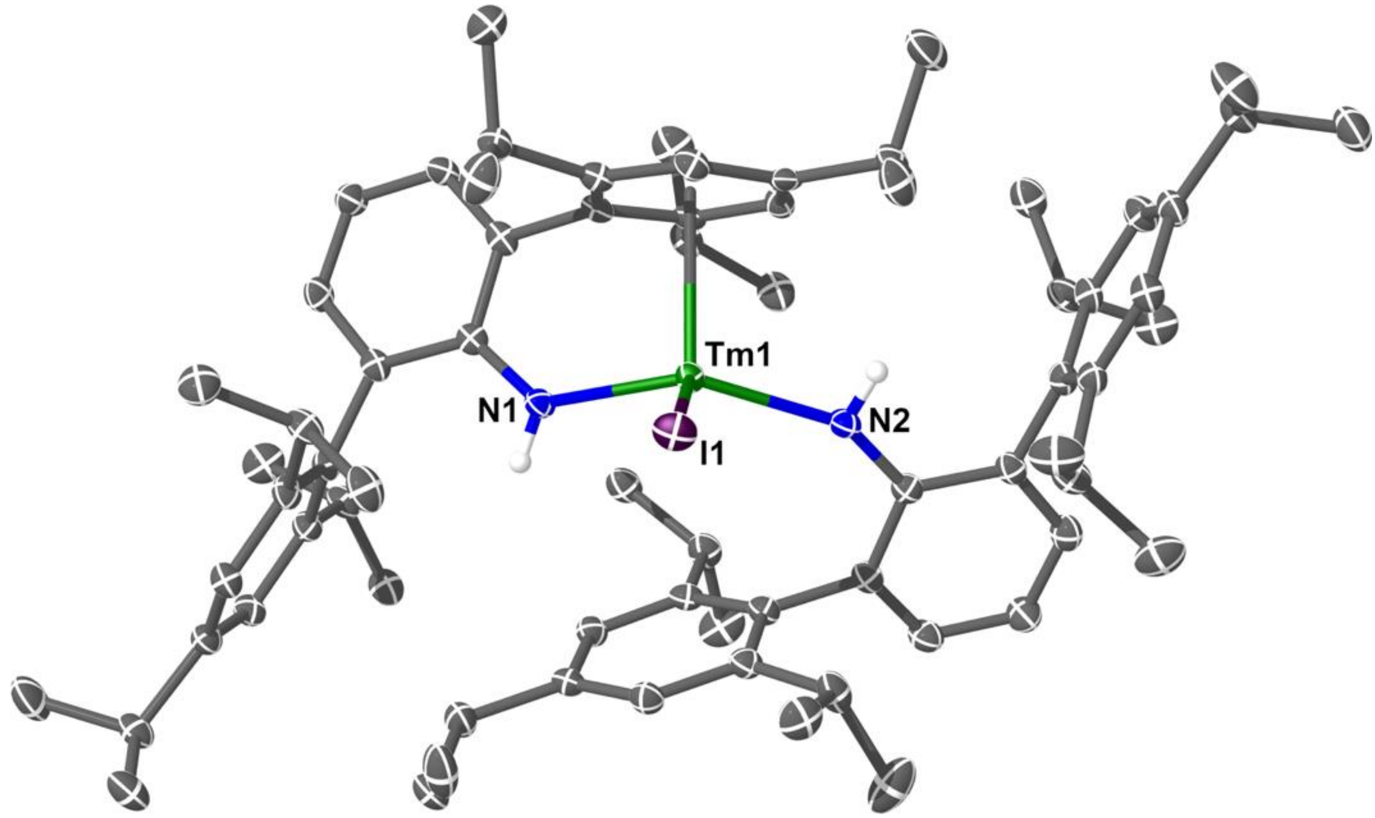

Figure S6. Molecular structure of **1Tm**. Ellipsoids set at 50% probability, H-atoms and two toluene molecules of crystallization removed for clarity (operations: X, Y, Z).

Tm(1)–I(1) = 2.8734(2) Å; Tm(1)–N(1) = 2.214(2) Å; Tm(1)–N(2) = 2.188(2) Å; Tm(1)–$C_{6\text{-range}}$ = 2.759(3)–2.903(3) Å; Tm(1)–$C_{6\text{-centroid}}$ = 2.4586(12) Å; Tm(1)–$C_{6\text{-centroid}}$ = 3.7587(12)Å; N(1)–Tm(1)–N(2) = 134.23°.

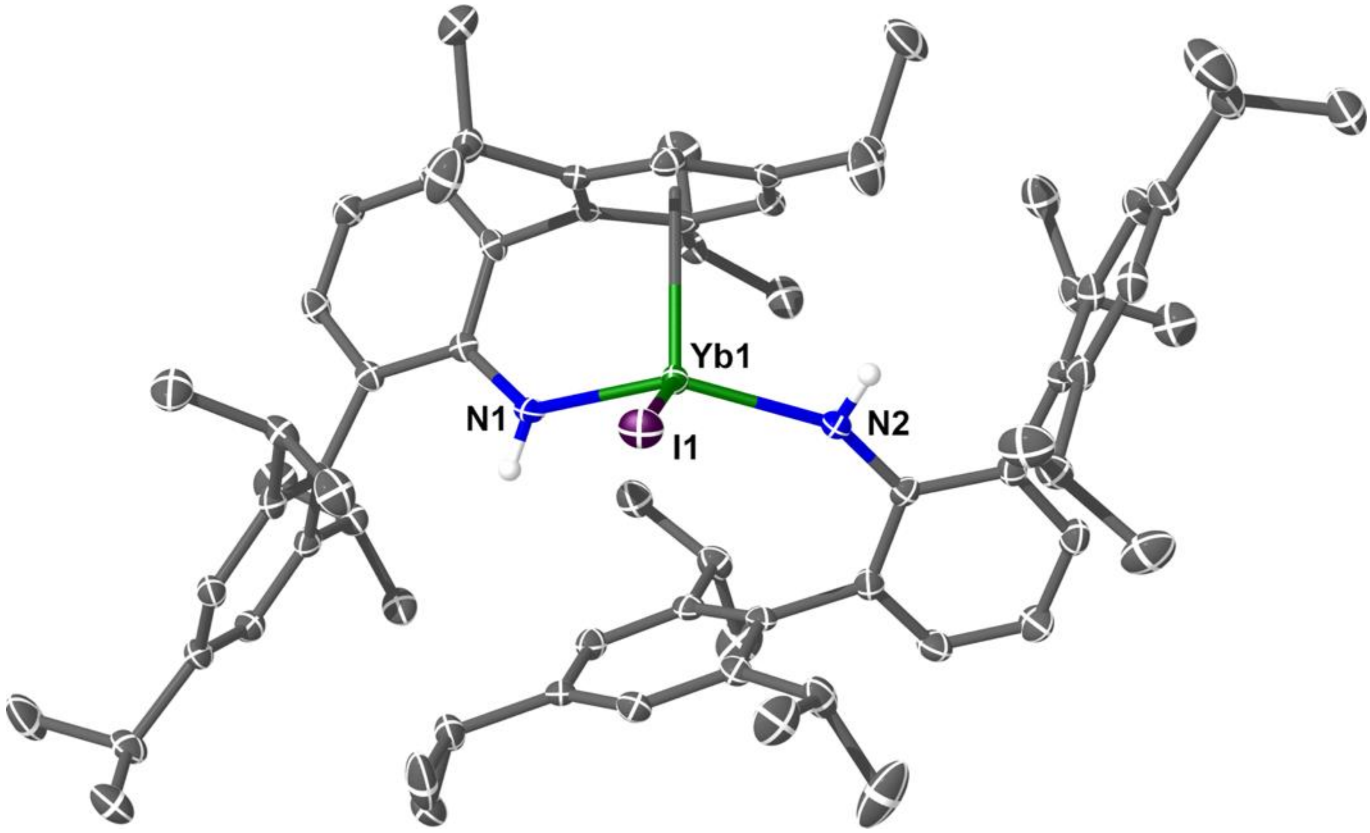

Figure S7. Molecular structure of **1Yb**. Ellipsoids set at 50% probability, H-atoms and two toluene molecules of crystallization removed for clarity (operations: X, Y, Z).

Yb(1)–I(1) = 2.85885(18) Å; Yb(1)–N(1) = 2.2019(18) Å; Yb(1)–N(2) = 2.1824(17) Å; Yb(1)–$C_{6\text{-range}}$ = 2.7505(19)–2.896(2) Å; Yb(1)–$C_{6\text{-centroid}}$ = 2.4572(9) Å; Yb(1)–$C_{6\text{-centroid}}$ = 3.7431(10) Å; N(1)–Yb(1)–N(2) = 134.96(7)°.

*Bond metrics for **1M** (M = Sc, Y, La, Tm, Yb), and [Y(NHAr^{iPr6})$_2$(Cl)]*

Table S8. Bond lengths (Å) and angles (°) for [M(NHAr^{iPr6})$_2$(I)] (**1M**, M = Sc, Y, La, Tm, Yb).

		**1Sc**	**1Y**	**1La**	**1Tm**	**1Yb**
**M–I**	M(1)	2.7666(3)	2.9108(2)	3.0866(3)	2.8734(2)	2.85885(18)
**M–N**	N(1)	2.0909(15)	2.2452(17)	2.402(2)	2.214(2)	2.2019(18)
	N(2)	2.0643(15)	2.2167(17)	2.399(2)	2.188(2)	2.1824(17)
**M–C$_{6\text{-range}}$**	Ring(1)	2.6282(17)–2.8116(18)	2.7912(19)–2.919(2)	3.132(3)–3.249(3)	2.759(3)–2.903(3)	2.7505(19)–2.896(2)
	Ring(2)	–	–	3.113(3)–3.270(3)	–	–
**M–C$_{6\text{-centroid}}$**	Ring(1)	2.3391(8)	2.4949(9)	2.8714(12)	2.4586(12)	2.4572(9)
	Ring(2)	–	–	2.8783(12)	–	–
**N–M–N**	M(1)	130.51(6)	135.67(6)	148.31(8)	134.23(8)	134.96(7)
**C$_{6\text{-centroid}}$–M–C$_{6\text{-centroid}}$**	M(1)	–	–	155.06(4)	–	–

Table S9. Bond lengths (Å) and angles (°) for [Y(NHAr^{iPr6})$_2$(X)] (X = Cl, I).

		**1Y**	[Y(NHAr^{iPr6})$_2$(Cl)] ††
**M–X**	M(1)	**X** = I; 2.9108(2)	**X** = Cl; 2.5071(8)
**M–N**	N(1)	2.2452(17)	2.249(2)
	N(2)	2.2167(17)	2.213(2)
**M–C$_{6\text{-range}}$**	Ring(1)	2.7912(19)–2.919(2)	2.782(3)–2.920(3)
	Ring(2)	–	–
**M–C$_{6\text{-centroid}}$**	Ring(1)	2.4949(9)	2.4952(14)
	Ring(2)	3.6894(9)	3.6987(13)
**N–M–N**	M(1)	135.67(6)	133.75(10)

†† [Y(NHAr^{iPr6})$_2$(Cl)] has been reported previously, the reported values are here for comparison.[14]

*Complexes **2M** (M = Sc, Y, La, Sm, Eu, Tm, Yb)*

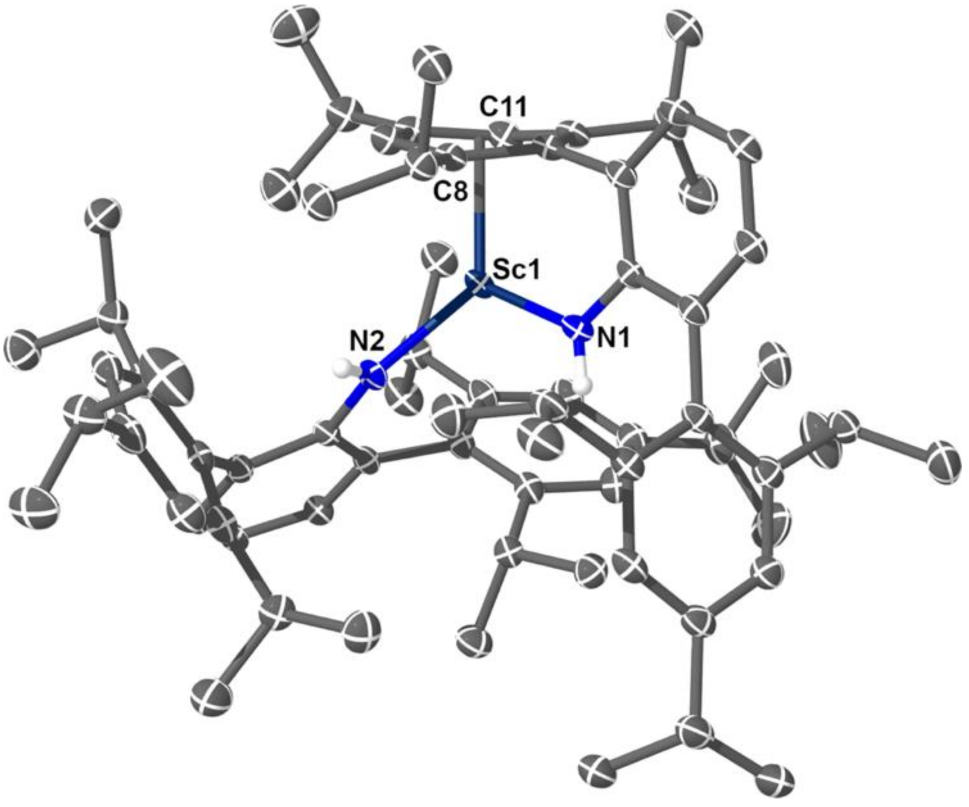

Figure S8. Molecular structure of **2Sc**. Ellipsoids set at 50% probability and H-atoms removed for clarity (operations: X, Y, Z).

Sc(1)–N(1) = 2.0884(11) Å; Sc(1)–N(2) = 2.2600(12) Å; Sc(1)–C_{range} = 2.3913(12)–2.6304(14) Å; Sc(1)–$C_{6\text{-centroid}}$ = 2.1006(7) Å; Sc(1)–C(8) = 2.3913(12) Å; Sc(1)–C(11) = 2.5418(13) Å; N(1)–Sc(1)–N(2) = 106.49(4)°; Arene fold angle ($\angle_{arene}$) = 11.43(11) °.

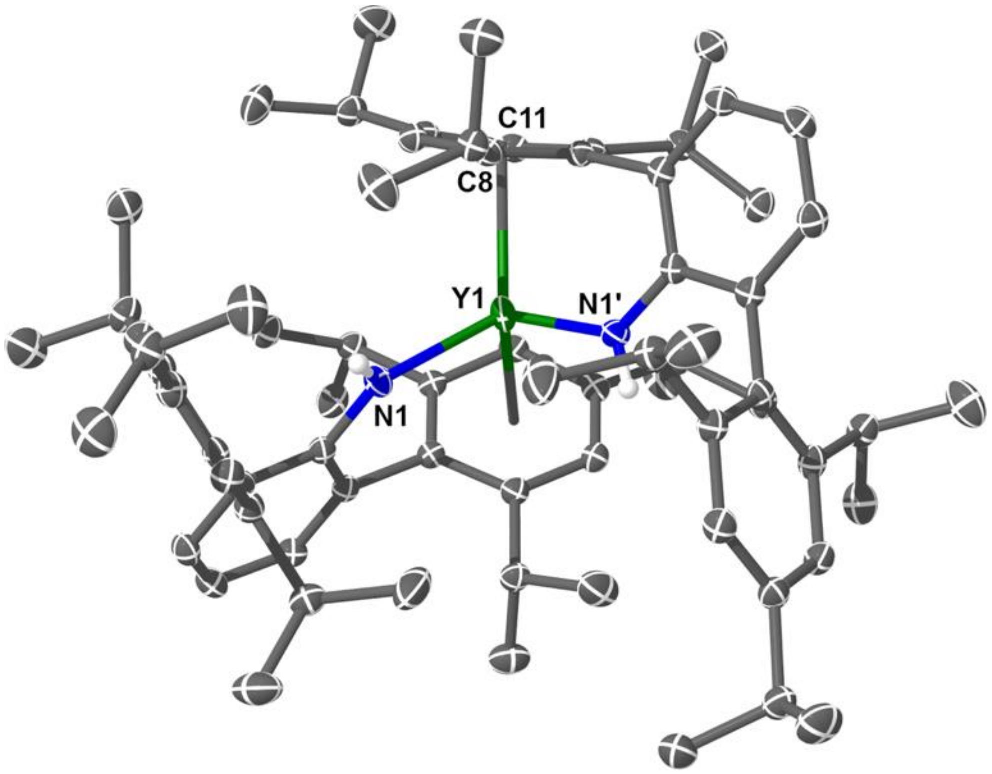

Figure S9. Molecular structure of **2Y**. Ellipsoids set at 50% probability, H-atoms and an Et_2O of crystallization removed for clarity (operations: X, Y, Z; 1–X, +Y, $^{3}/_{2}$–Z).

Y(1)–N(1) = 2.2600(12) Å; Y(1)–C_{range} = 2.7276(14)–2.9273(15) Å; Y(1)–$C_{6\text{-centroid}}$ = 2.4481(6) Å; Y(1)–C(8) = 2.7684(14) Å; Y(1)–C(11) = 2.7859(15) Å; N(1)–Y(1)–N(1') = 101.87(7) °; $C_{6\text{-centroid}}$–Y(1)–$C_{6'\text{-centroid}}$ = 133.40(3) Å; Arene fold angle ($\angle_{arene}$) = 7.27(12) ° on both metal-bound arene rings.

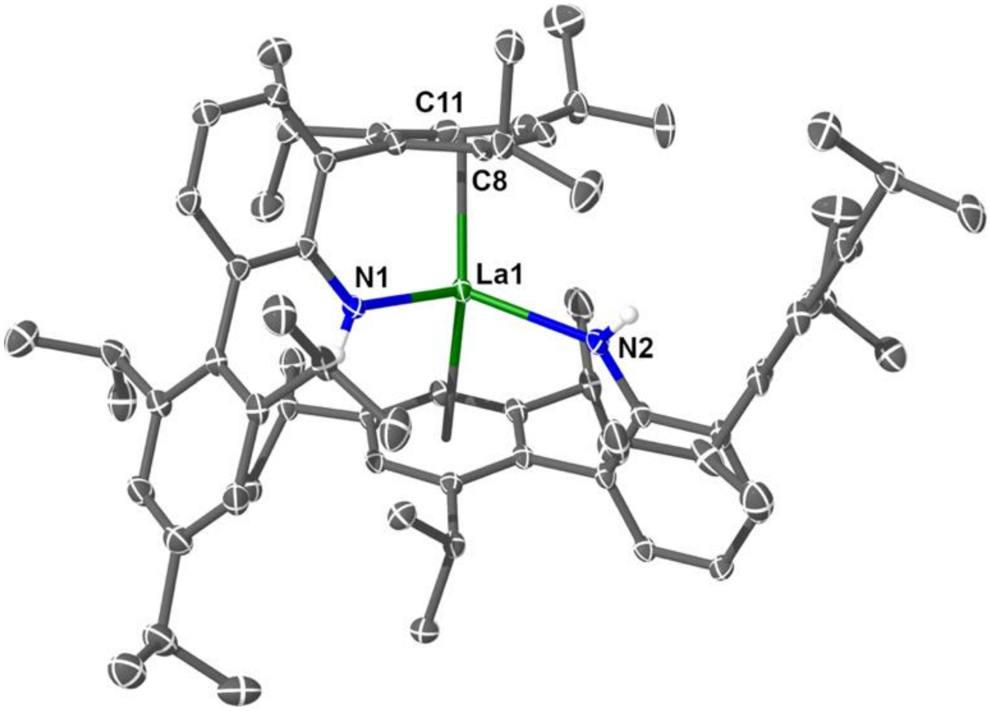

Figure S10. Molecular structure of **2La**. Ellipsoids set at 50% probability, H-atoms and an Et_2O of crystallization removed for clarity (operations: X, Y, Z).

La(1)–N(1) = 2.395(3) Å; La(1)–N(2) = 2.434(3) Å; La(1)–$C_{6\text{-range}}$ = 2.778(16)–2.971(9) Å; La(1)–$C_{6\text{-range}}$ = 3.047(3)–3.240(3) Å; La(1)–$C_{6\text{-centroid}}$ = 2.479(5) Å; La(1)–$C_{6\text{-centroid}}$ = 2.8348(12) Å; La(1)–C(8) = 2.843(13) Å; La(1)–C(11) = 2.903(12) Å; N(1)–La(1)–N(2) = 113.60(10) °; $C_{6\text{-centroid}}$–La(1)–$C_{6\text{-centroid}}$ = 151.47(12) Å; Arene fold angle ($\angle_{arene}$) = 12.9(9) °.

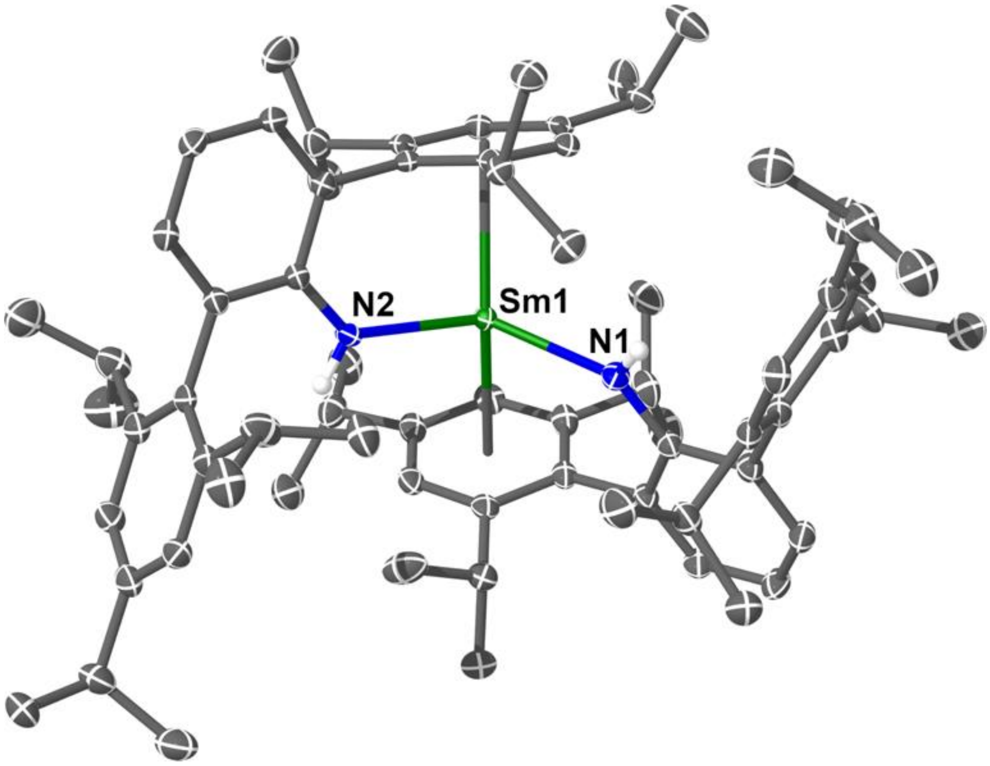

Figure S11. Molecular structure of **2Sm**. Ellipsoids set at 50% probability, H-atoms and an Et_2O of crystallization removed for clarity (operations: X, Y, Z).

Sm(1)–N(1) = 2.412(2) Å; Sm(1)–N(2) = 2.425(2) Å; Sm(1)–$C_{6\text{-range}}$ = 2.955(3)–3.160(3) Å; Sm(1)–$C_{6\text{-range}}$ = 2.953(3)–3.201(3) Å; Sm(1)–$C_{6\text{-centroid}}$ = 2.7032(12) Å; Sm(1)–$C_{6\text{-centroid}}$ = 2.7265(12) Å; N(1)–Sm(1)–N(2) = 115.25(9)°; $C_{6\text{-centroid}}$–Sm(1)–$C_{6\text{-centroid}}$ = 153.70(4)°.

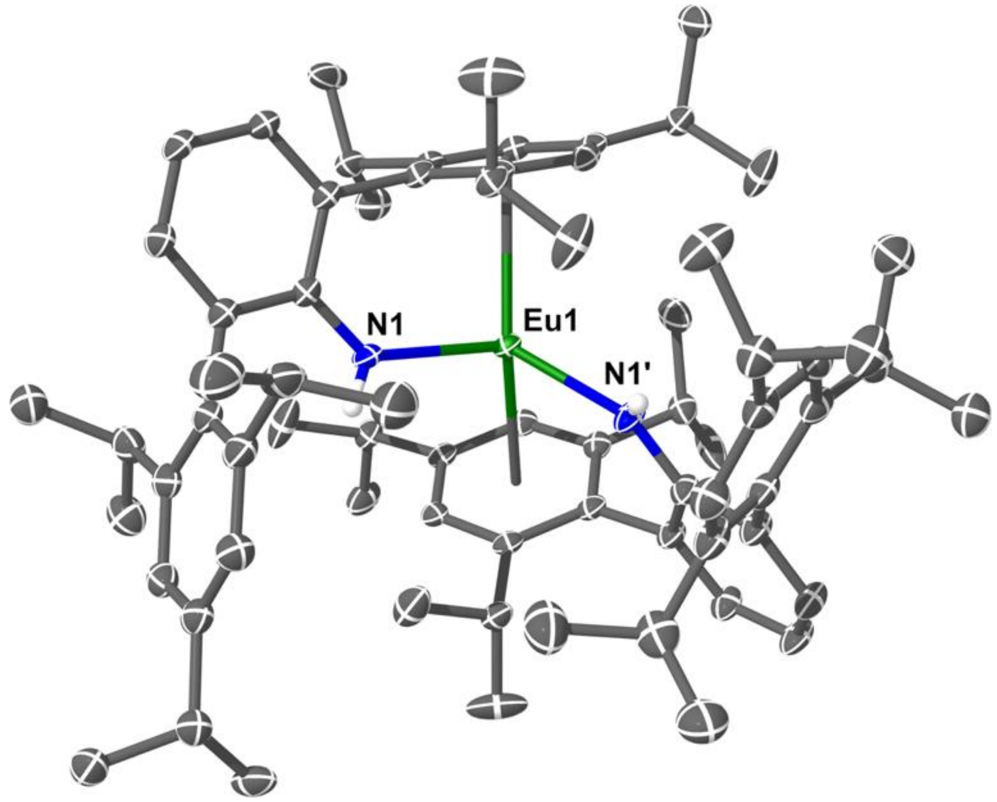

Figure S12. Molecular structure of **2Eu**. Ellipsoids set at 50% probability and H-atoms removed for clarity along with a second molecule of **2Eu** (operations: X, Y, Z; 1–X, +Y, 2–Z).

Eu(1)–N(1) = 2.411(4) Å; Eu(1)–$C_{6\text{-range}}$ = 2.972(4)–3.176(5) Å; Eu(1)–$C_{6\text{-centroid}}$ = 2.7604(17) Å; N(1)–Eu(1)–N(1') = 107.5(2)°; $C_{6\text{-centroid}}$–Eu(1)–$C_{6'\text{-centroid}}$ = 147.87(10)°.

Eu(2)–N(2) = 2.414(5) Å; Eu(2)–$C_{6\text{-range}}$ = 2.947(4)–3.214(4) Å; Eu(2)–$C_{6\text{-centroid}}$ = 2.7343(16) Å; N(2)–Eu(2)–N(2') = 108.2(2)°; $C_{6\text{-centroid}}$–Eu(2)–$C_{6'\text{-centroid}}$ = 148.88(10)°.

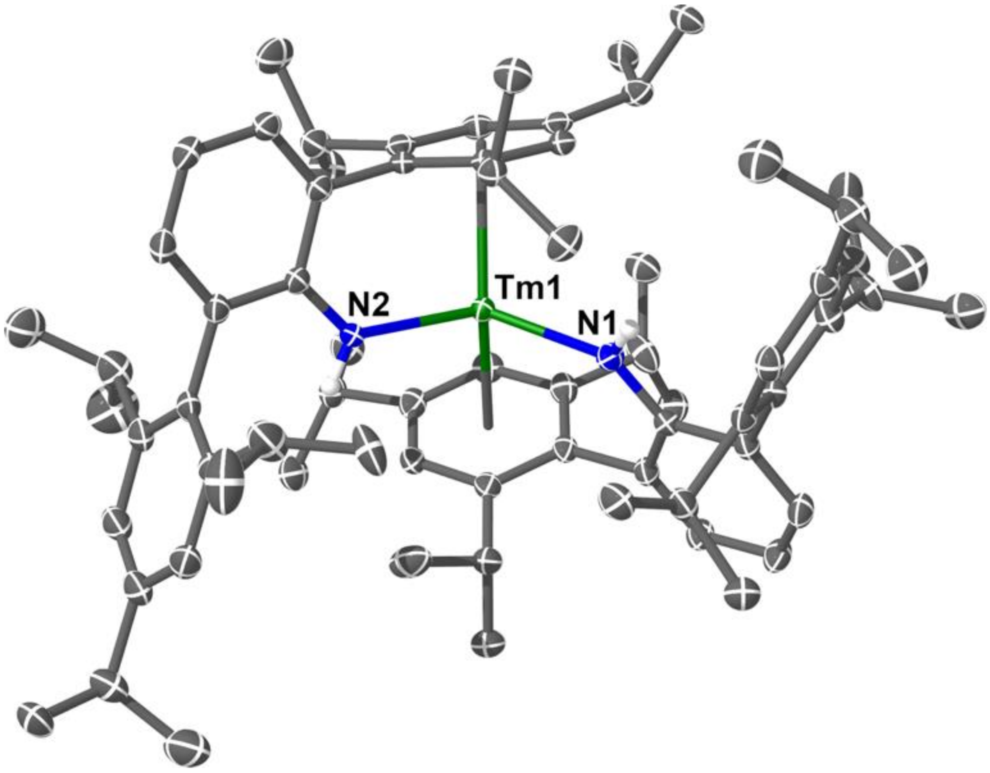

Figure S13. Molecular structure of **2Tm**. Ellipsoids set at 50% probability, H-atoms and an Et_2O of crystallization removed for clarity (operations: X, Y, Z).

Tm(1)–N(1) = 2.3060(17) Å; Tm(1)–N(2) = 2.3169(18) Å; Tm(1)–$C_{6\text{-range}}$ = 2.8015(19)–2.971(2) Å; Tm(1)–$C_{6\text{-range}}$ = 2.819(2)–3.118(2) Å; Tm(1)–$C_{6\text{-centroid}}$ = 2.5579(9) Å; Tm(1)–$C_{6\text{-centroid}}$ = 2.5969(9) Å; N(1)–Tm(1)–N(2) = 116.45(7)°; $C_{6\text{-centroid}}$–Tm(1)–$C_{6\text{-centroid}}$ = 148.37(3)°.

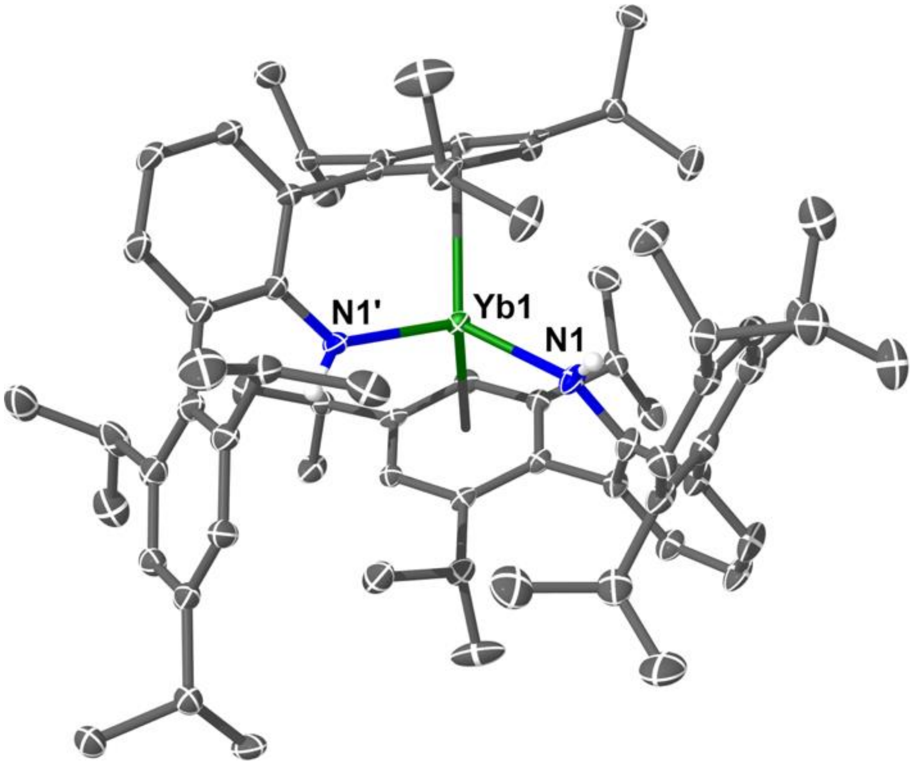

Figure S14. Molecular structure of **2Yb**. Ellipsoids set at 50% probability and H-atoms removed for clarity along with a second molecule of **2Yb** (operations: X, Y, Z; 1–X, +Y, 2–Z).

Yb(1)–N(1) = 2.310(6) Å; Yb(1)–$C_{6\text{-range}}$ = 2.840(6)–3.047(6) Å; Yb(1)–$C_{6\text{-centroid}}$ = 2.629(2) Å; N(1)–Yb(1)–N(1') = 108.5(3)°; $C_{6\text{-centroid}}$–Yb(1)–$C_{6}'_{\text{-centroid}}$ = 145.63(14)°.

Yb(2)–N(2) = 2.294(6) Å; Yb(2)–$C_{6\text{-range}}$ = 2.897(6)–3.181(7) Å; Yb(2)–$C_{6\text{-centroid}}$ = 2.659(3) Å; N(2)–Yb(2)–N(2') = 107.1(3)°; $C_{6\text{-centroid}}$–Yb(2)–$C_{6}'_{\text{-centroid}}$ = 144.68(13)°.

*Bond metrics for **2M** (M = Sc, Y, La, Sm, Eu, Tm, Yb)*

Table S10. Bond lengths (Å) and angles (°) for [M(NHAr^{iPr6})$_2$] (**2M**, M = Sc, Y, La, Sm, Eu, Tm, Yb).

	**2Sc**	**2Y** [A]	**2La**	**2Sm**	**2Eu** [B]	**2Tm**	**2Yb** [B]
**M–N** N(1) M(1)	2.0884(11)	2.2600(12)	2.395(3)	2.412(2)	2.411(4)	2.3060(17)	2.310(6)
M(2)					2.414(5) [B]		2.294(6) [B]
N(2)	2.0678(10)	– [A]	2.434(3)	2.425(2)	– [B]	2.3169(18)	– [B]
**M–C$_{6\text{-range}}$** Ring(1)	2.3913(12)–2.6304(14)	2.7276(14)–2.9273(15)	2.778(16)–2.971(9)	2.955(3)–3.160(3)	2.972(4)–3.176(5)	2.8015(19)–2.971(2)	2.840(6)–3.047(6)
Ring(2)	–	– [A]	3.047(3)–3.240(3)	2.953(3)–3.201(3)	– [B]	2.819(2)–3.118(2)	– [B]
**M–C** C(8)	2.3913(12)	2.7684(14)	2.843(13)	–	–	–	–
C(11)	2.5418(13)	2.7859(15)	2.903(12)	–	–	–	–
**M–C$_{6\text{-centroid}}$** Ring(1)	2.1006(7)	2.4481(6)	2.479(5)	2.7032(12)	2.7604(17)	2.5579(9)	2.629(2)
					2.7343(16) [B]		2.659(3) [B]
Ring(2)	–	– [A]	2.8348(12)	2.7265(12)	– [B]	2.5969(9)	– [B]
**N–M–N** M(1)	106.49(4)	101.87(7)	113.60(10)	115.25(9)	107.5(2)	116.45(7)	108.5(3)
**C$_{6\text{-centroid}}$–M–C$_{6\text{-centroid}}$** M(1)	–	133.40(3)	151.47(12)	153.70(4)	148.88(10)	148.37(3)	145.63(14)
M(2)					147.87(10)		144.68(13)
**Arene fold angle**	11.43(11)	7.27(12)	12.9(9)	–	–	–	–

[A] The solid-state structure is C_2-symmetric so there is a single metal and ligand per asymmetric unit; [B] The solid-state structures show two half-molecules in the asymmetric unit, so M(1) and M(2) each have only one unique ligand.

*A discussion of M–N bonding in **1M** and **2M***

While the two M–N bond lengths in **1La** are statistically indistinguishable, in both **1Sc** and **1Y** one is shorter than the other by a significant amount (Δ_{M-N} in **1Sc** = 0.0266(15) Å; and in **1Y** = 0.0285(17) Å where the number in parenthesis is the root sum of squares of their respective standard uncertainties). This reflects the different M⋯$C_{6\text{-arene}}$ contacts present in **1Sc**, and **1Y**, versus those in **1La**.

As seen with **1Sc**, complex **2Sc** shows two Sc–N distances where one is shorter and one longer (2.0678(10) and 2.0884(11) Å), but each long and short pair between **1Sc** and **2Sc** is statistically indistinguishable at the 3σ level. In both **1Sc** and **2Sc** it is the longer of the two Sc–N distances which derives from the ligand with shortest M–C contacts, in **2Sc** it is this arene ring which is deformed. In **2Sc** the N–Sc–N angle (106.49(4)°) is much more acute than **1Sc** (130.51(6)°). When comparing **1Y** to **2Y**, we see that the Y–N in **2Y** (2.2600(12) Å) is longer than both Y–N distances in **1Y** (2.2167(17) and 2.2452(17) Å). In **2La** one La–N distance (2.395(3) Å) is statistically indistinguishable from both La–N distances in **1La** (2.399(2) Å, 2.402(2) Å), while the other (2.434(3) Å) is significantly longer. As opposed to the situation with **2Sc**, in **2La** it is the shorter of the La–N distances which is from the {NHAr^{iPr6}} ligand bearing the deformed Tripp group and the shortest M–C contacts (range: 2.778(16)–2.971(9) Å, *vs* 3.047(3)–3.240(3) Å for the opposing Tripp group). There is limited capacity for the flanking Tripp groups to flex towards or away from the metal without also affecting the M–N distance, and so a trend can be seen whereby if the metal is very small and has one close Tripp (M⋯$C_{6\text{-arene}}$) interaction, as with **1Sc**, **1Y**, and **2Sc**, the M–N distance to this {NHAr^{iPr6}} ligand increases to compensate, presumably to avoid clashing with the second ligand. In the case of **2La**, **2U**,[14] and to some extent **2Y**, the metal is much larger and so as the M⋯$C_{6\text{-arene}}$ distance contracts, the metal is still able to sit well within the pocket defined by the Tripp $C_{6\text{-arene}}$ and N(H) groups. This can be seen in the $C_{6\text{-centroid}}$–M–N angles which increase from **2La** (93.70(14)°) → **2U** (94.40(6)°) → **2Y** (95.64(4)°), while **2Sc** is significantly larger at 104.84(4)°. Complex **2Y** sits somewhat in the middle of the range as it can accommodate close contacts to two Tripp groups, but both Y–N distances are longer than in precursor **1Y**.

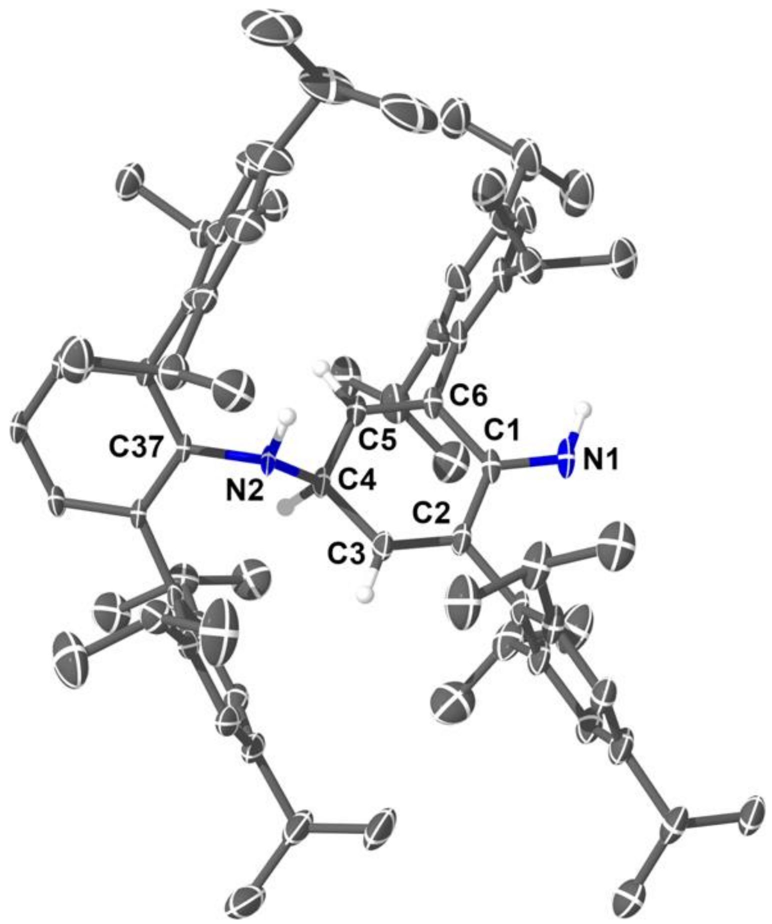

Figure S15. Molecular structure of **3**. Ellipsoids set at 50% probability and H-atoms removed for clarity (operations: X, Y, Z).

N(1)–C(1) = 1.288(3) Å; N(2)–C(4) = 1.479(3) Å; N(2)–C(37) = 1.418(2) Å; C(2)–C(3) = 1.342(3) Å; C(3)–C(4) = 1.488(3) Å; C(4)–C(5) = 1.498(3) Å; C(5)–C(6) = 1.340(3) Å; C(4)–N(2)–C(37) = 116.19(18)°.

S4. NMR spectroscopy plots

*NMR spectra of **1M** (M = Sc, Y, La, Tm, Yb)*

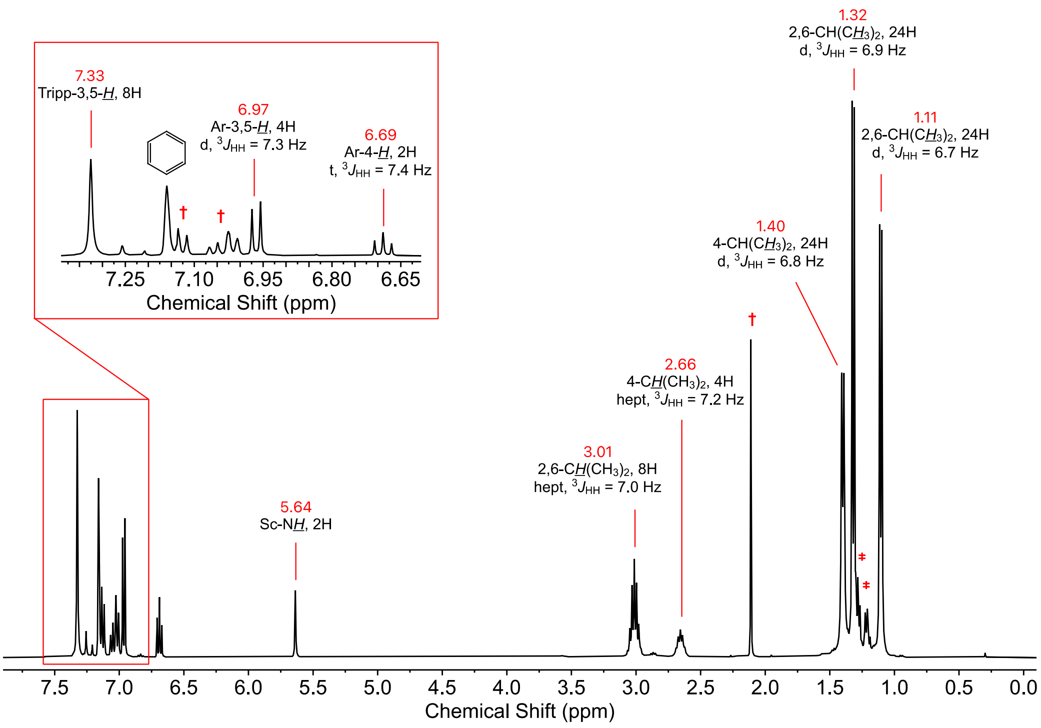

Figure S16. ^{1}H NMR spectrum of [Sc(NHAr^{iPr6})$_2$(I)] (**1Sc**) in d_6-benzene. ‡ and † denote residual *n*-hexane and toluene, respectively.

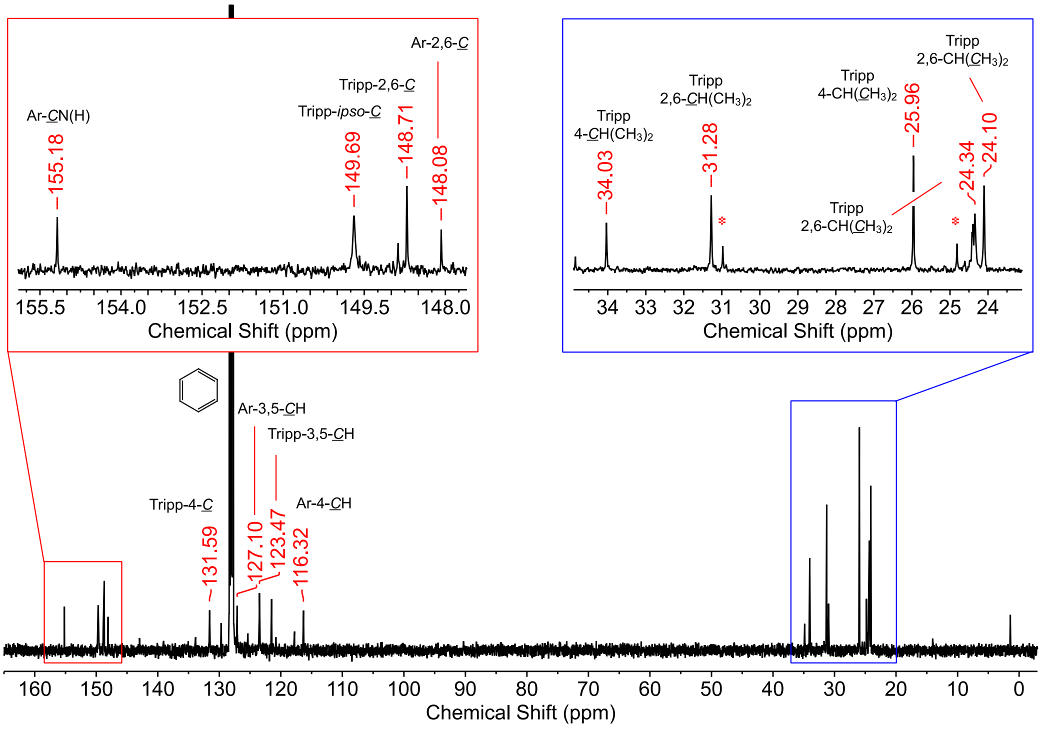

Figure S17. $^{13}C\{^{1}H\}$ NMR spectrum of [Sc(NHAr^{iPr6})$_2$(I)] (**1Sc**) in d_6-benzene. ‡ and † denote residual *n*-hexane and toluene, respectively.

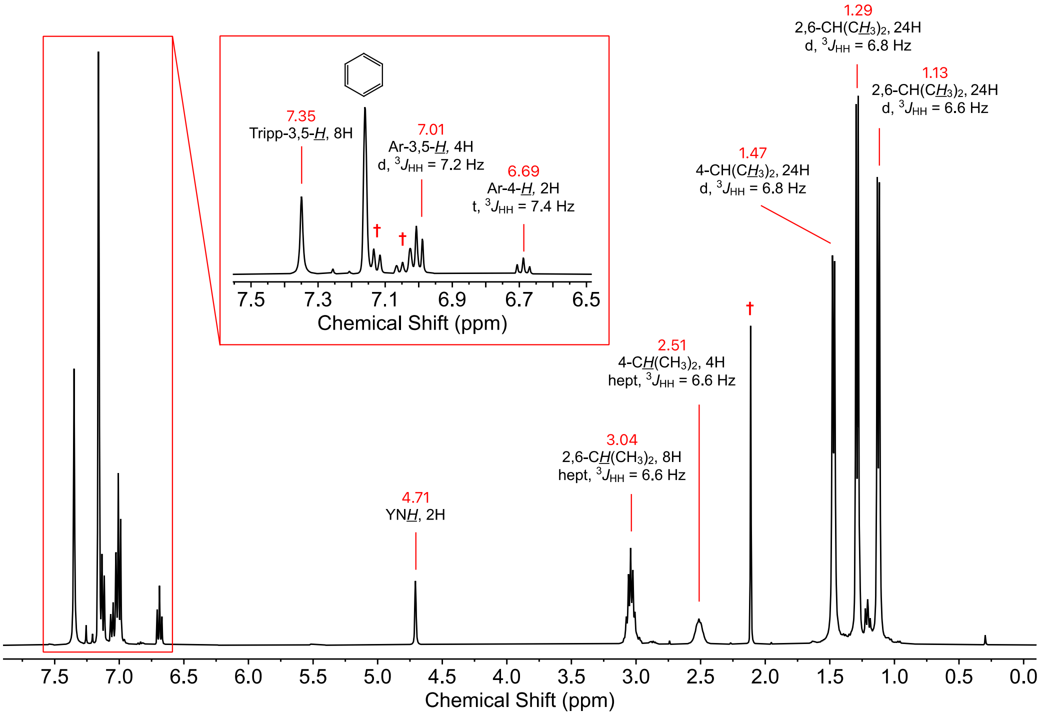

Figure S18. ^{1}H NMR spectrum of [Y(NHAr^{iPr6})$_2$(I)] (**1Y**) in d_6-benzene. † denotes residual toluene.

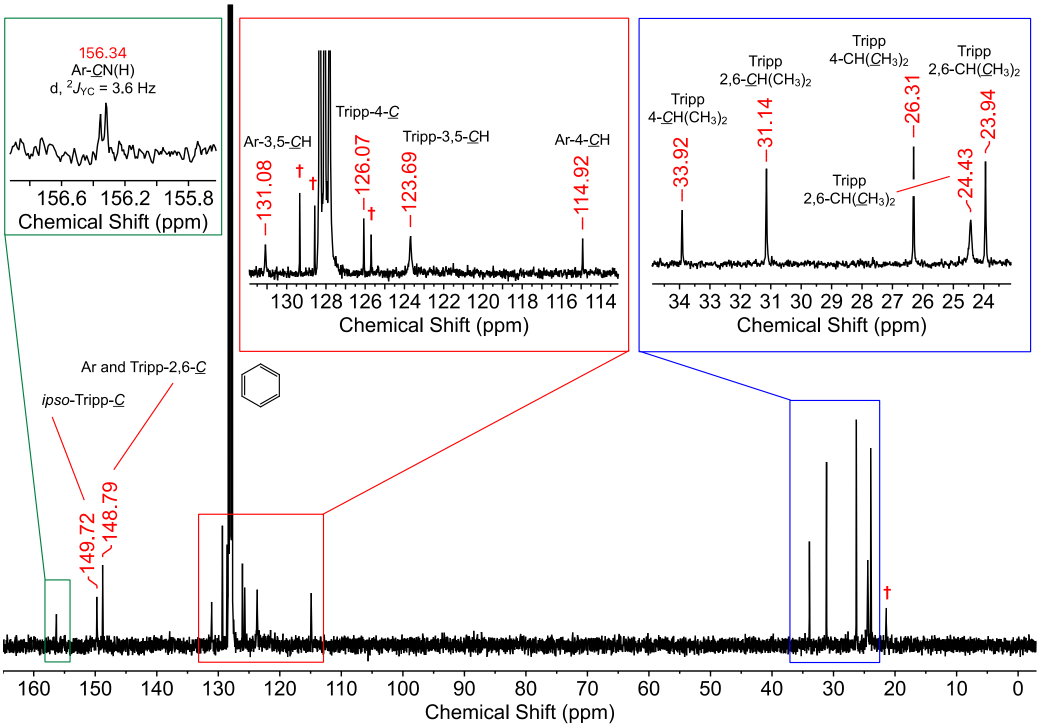

Figure S19. ^{13}C{^{1}H} NMR spectrum of [Y(NHAr^{iPr6})$_2$(I)] (**1Y**) in d_6-benzene. † denotes residual toluene.

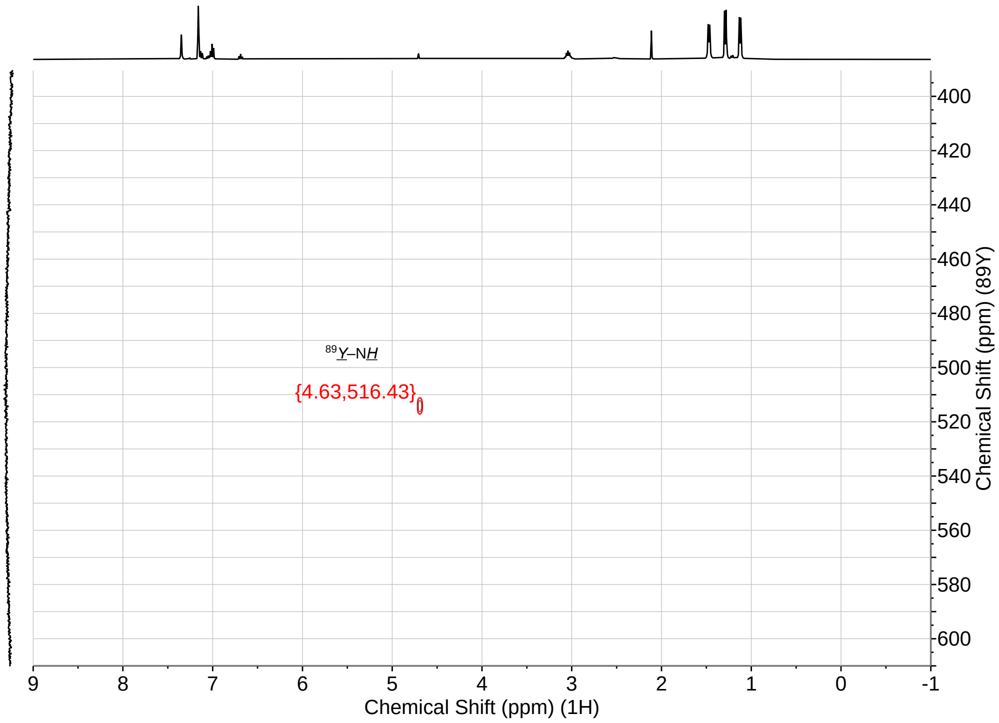

Figure S20. ^{1}H-^{89}Y HMBC NMR spectrum of [Y(NHAr^{iPr6})$_2$(I)] (**1Y**) in d_6-benzene. Exponential (–3.0 Hz) and Gaussian (2.0 Hz) line broadening were applied to both domains.

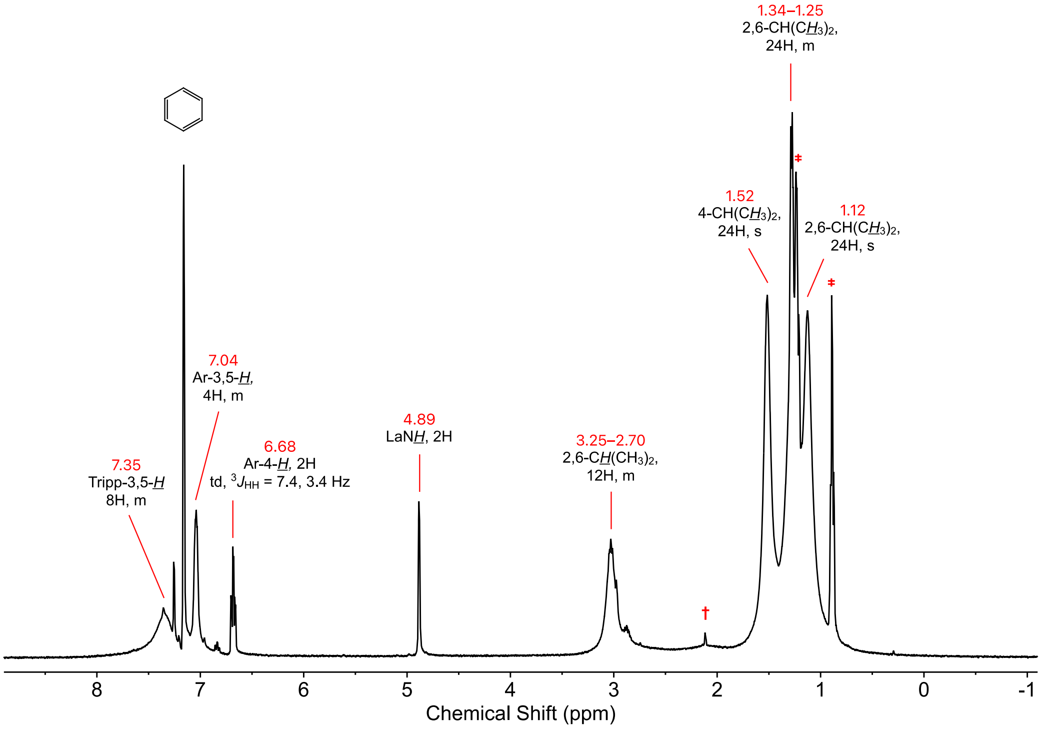

Figure S21. ^{1}H NMR spectrum of [La(NHAr^{iPr6})$_2$(I)] (**1La**) in d_6-benzene. ‡ and † denote residual *n*-hexane and toluene, respectively.

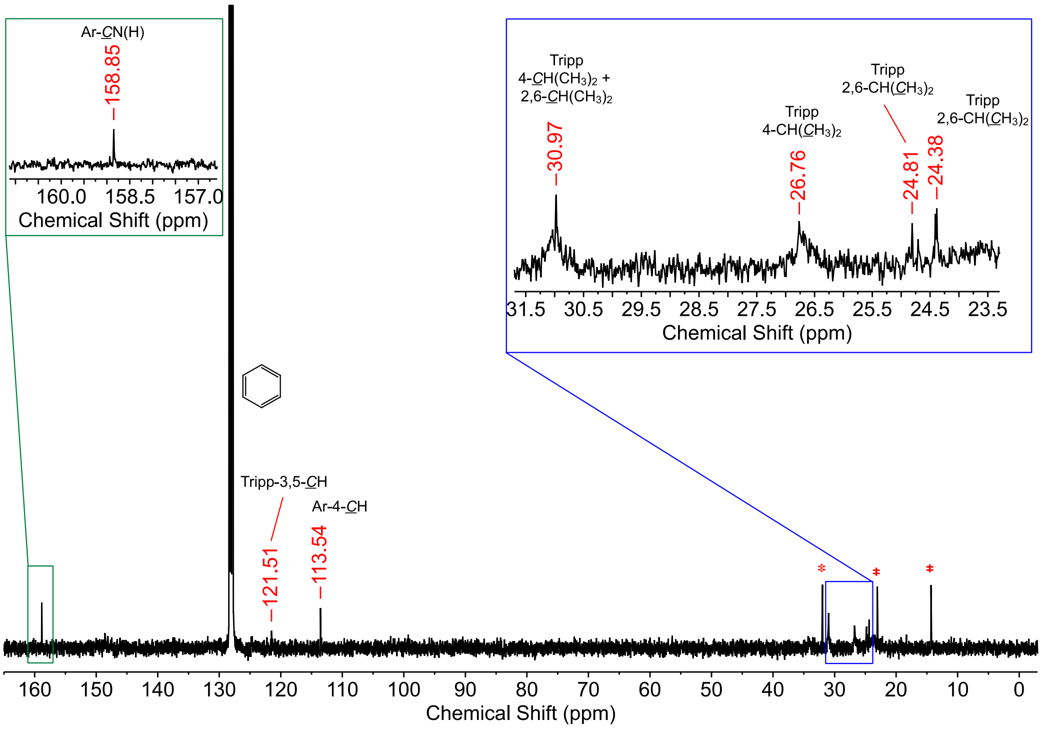

Figure S22. $^{13}C\{^1H\}$ NMR spectrum of $[La(NHAr^{iPr6})_2(I)]$ (**1La**) in d_6-benzene. ‡ denotes residual *n*-hexane.

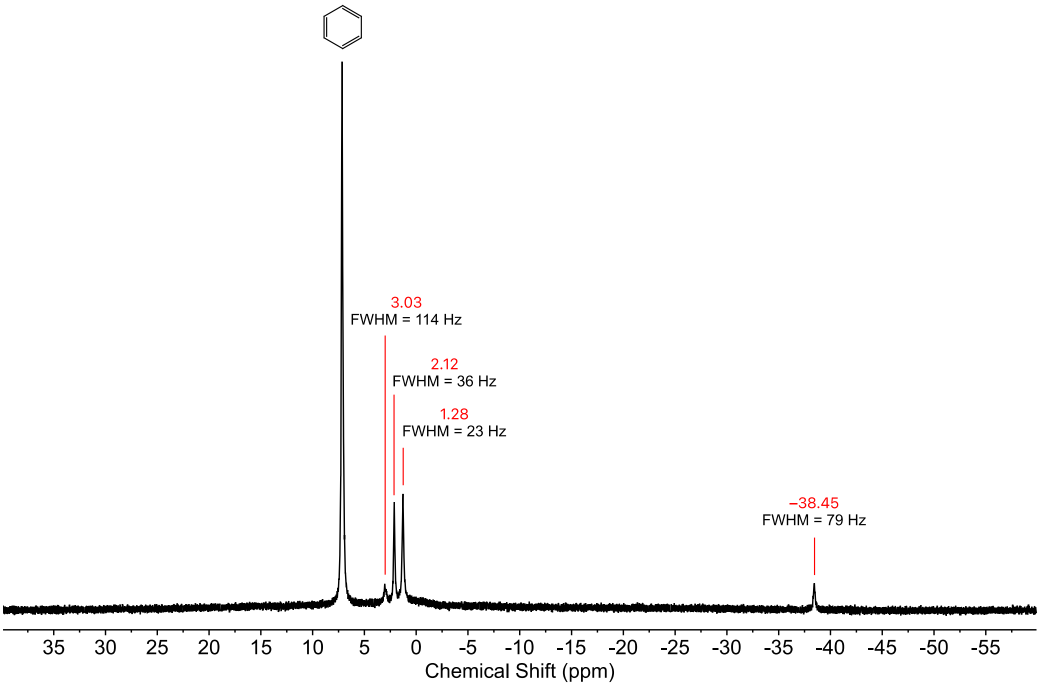

Figure S23. ^1H NMR spectrum of $[Tm(NHAr^{iPr6})_2(I)]$ (**1Tm**) in d_6-benzene from +40 to –60 ppm, showing the only observable peaks.

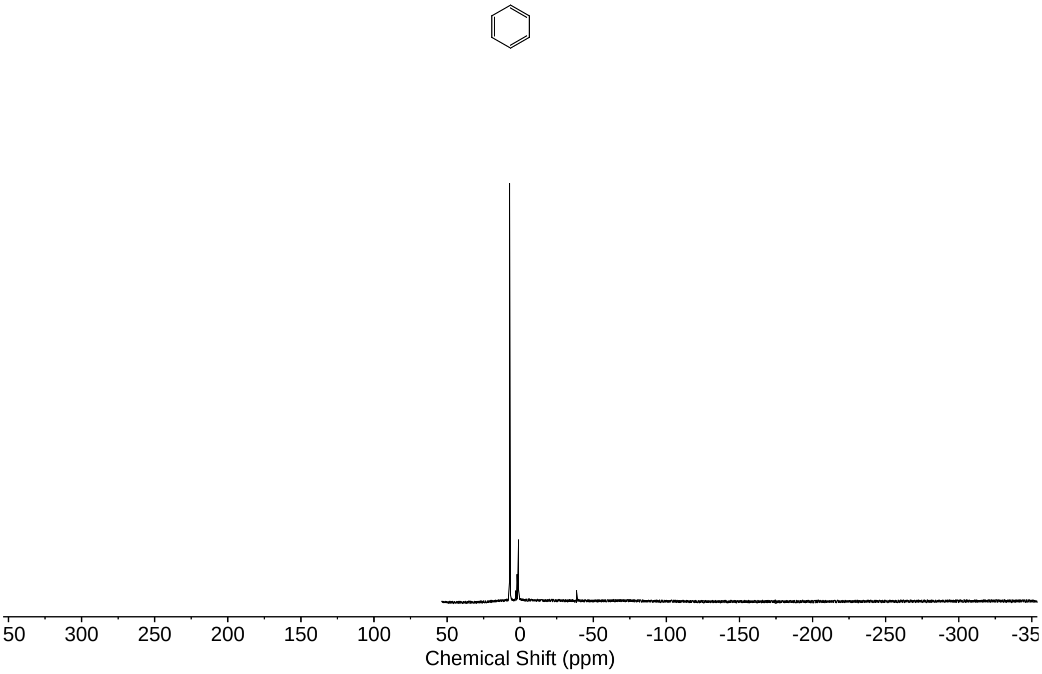

Figure S24. ^{1}H NMR spectra of [Tm(NHAr^{iPr6})$_2$(I)] (**1Tm**) in d_6-benzene from +345 to –345 ppm, showing the only observable peaks.

*NMR spectra of **2M** (M = Sc, Y, La, Sm, Eu, Tm, Yb)*

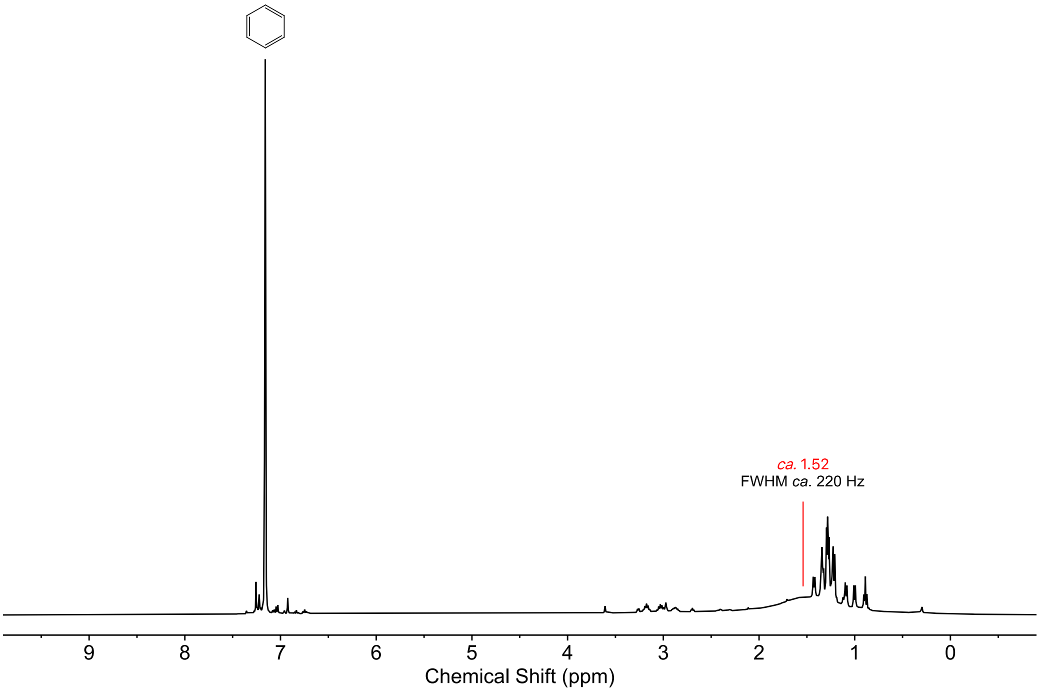

Figure S25. ^{1}H NMR spectrum of [Sc(NHAr^{iPr6})$_2$] (**2Sc**) in d_6-benzene from +10 to –1 ppm, showing the only observable peaks.

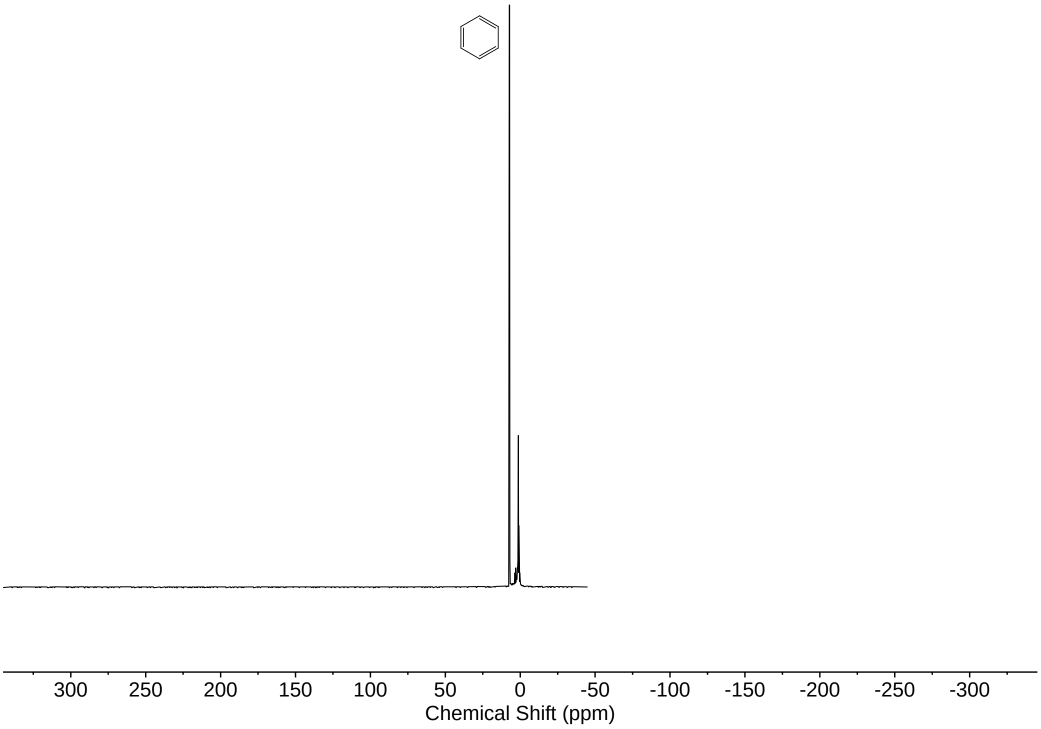

Figure S26. ^{1}H NMR spectra of [Sc(NHAr^{iPr6})$_2$] (**2Sc**) in d_6-benzene from +345 to –345 ppm, showing the only observable peaks.

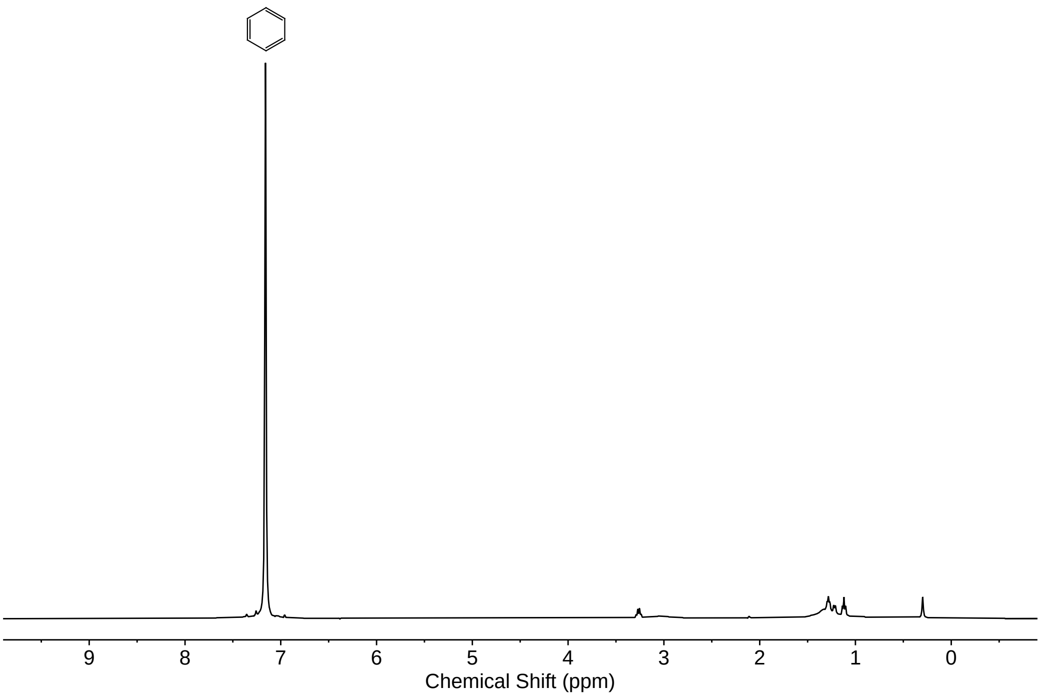

Figure S27. ^{1}H NMR spectrum of [Y(NHAr^{iPr6})$_2$] (**2Y**) in d_6-benzene from +10 to –1 ppm, showing the only observable peaks.

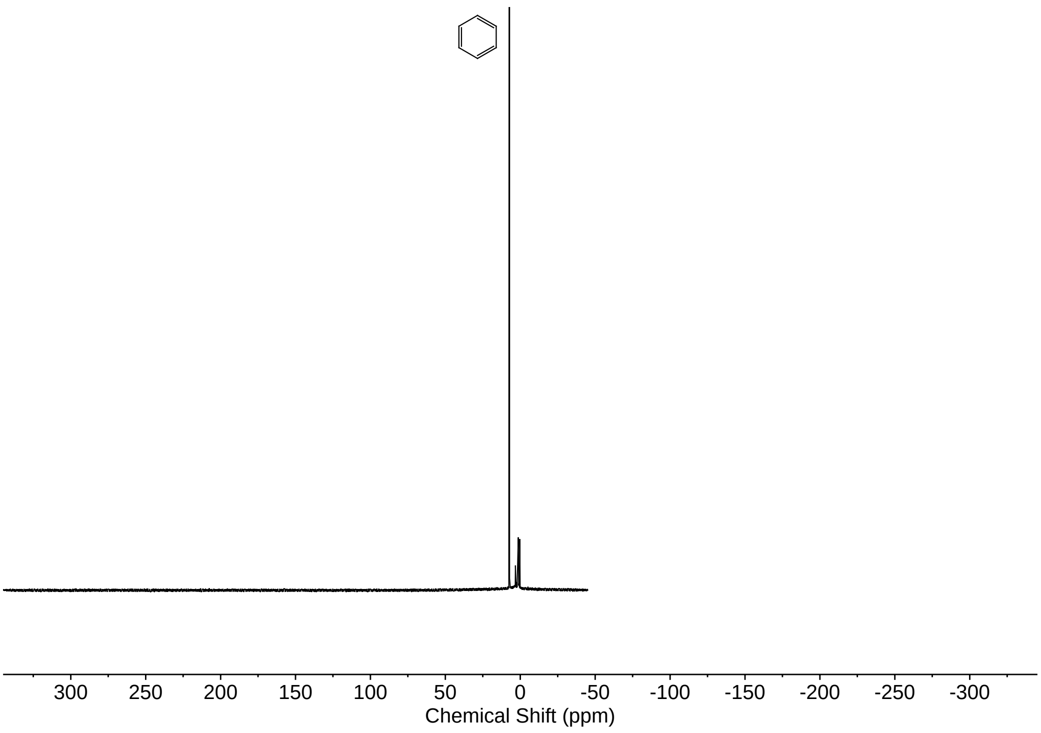

Figure S28. ^{1}H NMR spectra of [Y(NHAr^{iPr6})$_2$] (**2Y**) in d_6-benzene from +345 to –345 ppm, showing the only observable peaks.

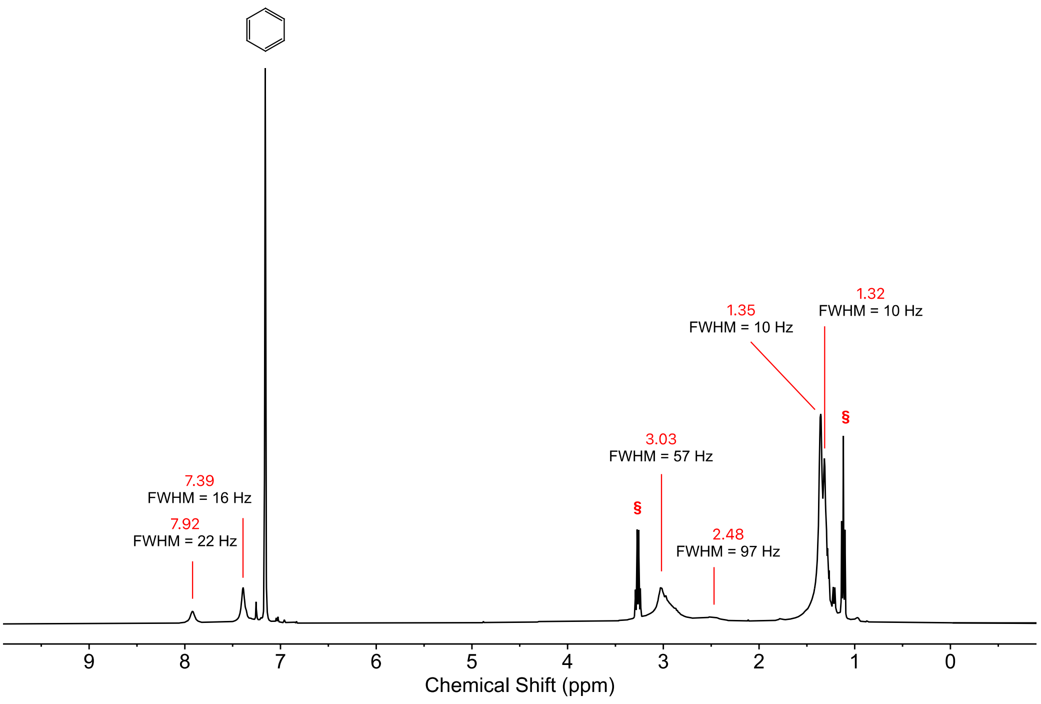

Figure S29. ^{1}H NMR spectrum of [La(NHAr^{iPr6})$_2$] (**2La**) in d_6-benzene from +10 to –1 ppm, showing the only observable peaks. § denotes residual Et_2O from crystallization.

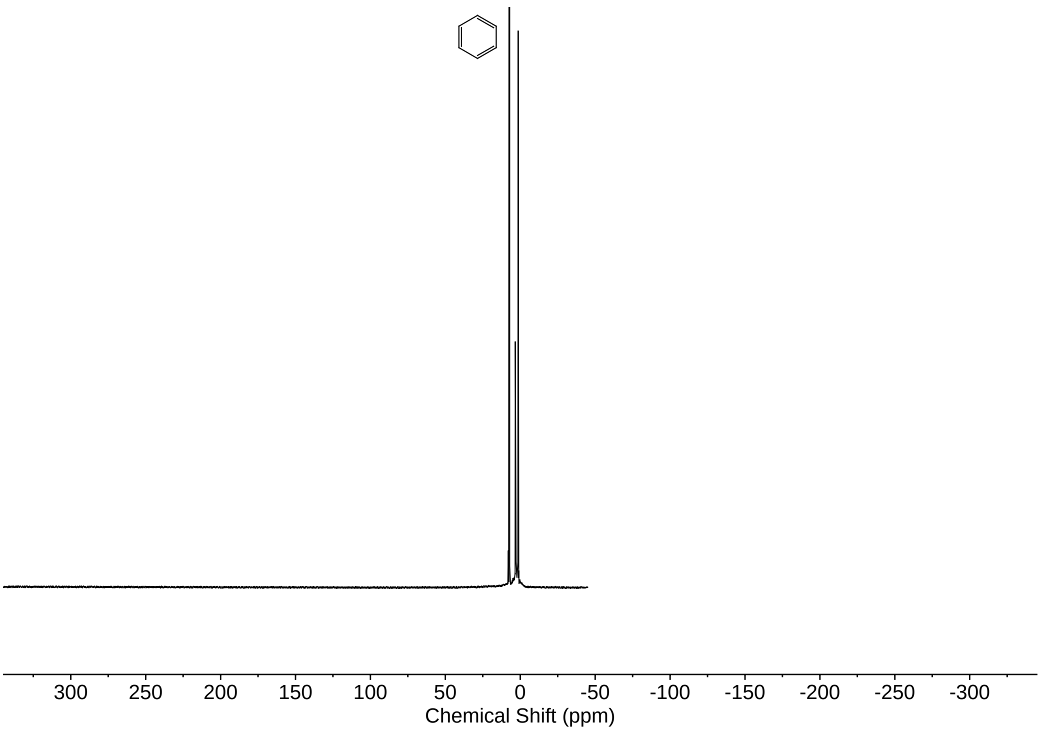

Figure S30. ^{1}H NMR spectra of [La(NHAr^{iPr6})$_2$] (**2La**) in d_6-benzene from +345 to –345 ppm, showing the only observable peaks.

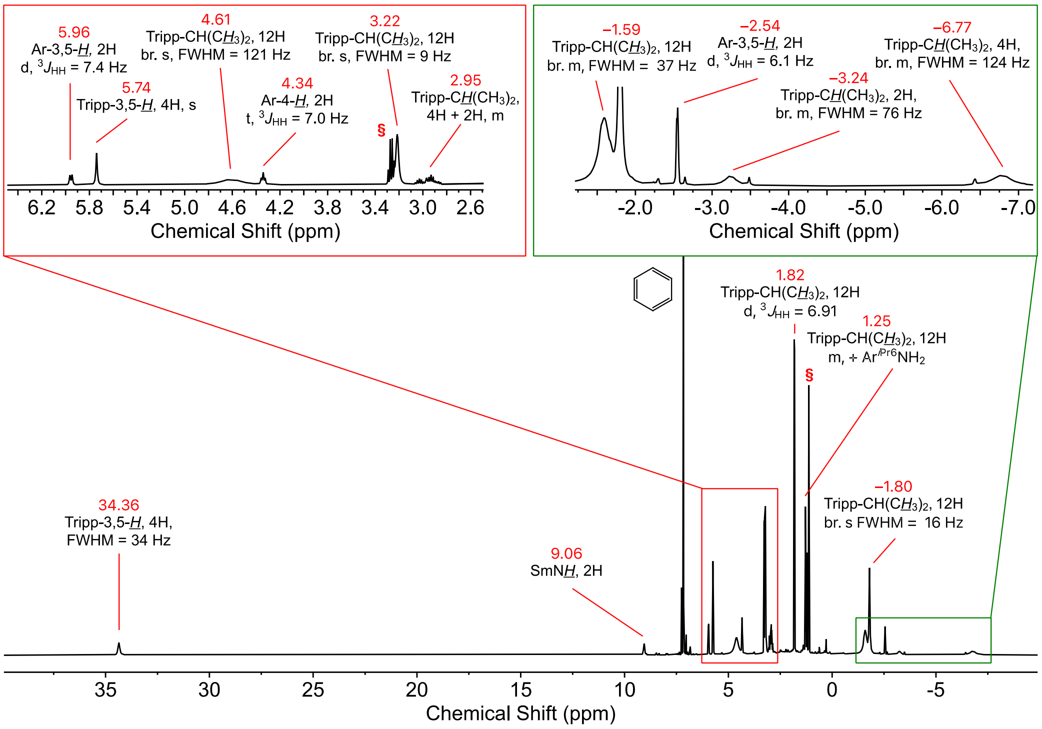

Figure S31. ^{1}H NMR spectrum of [Sm(NHAr^{iPr6})$_2$] (**2Sm**) in d_6-benzene from +40 to –10 ppm, showing the only observable peaks. § denotes residual Et_2O from crystallization. Trace NH_2Ar^{iPr6} impurities are not marked, for clarity, as they often somewhat overlap the peaks for **2Sm**.

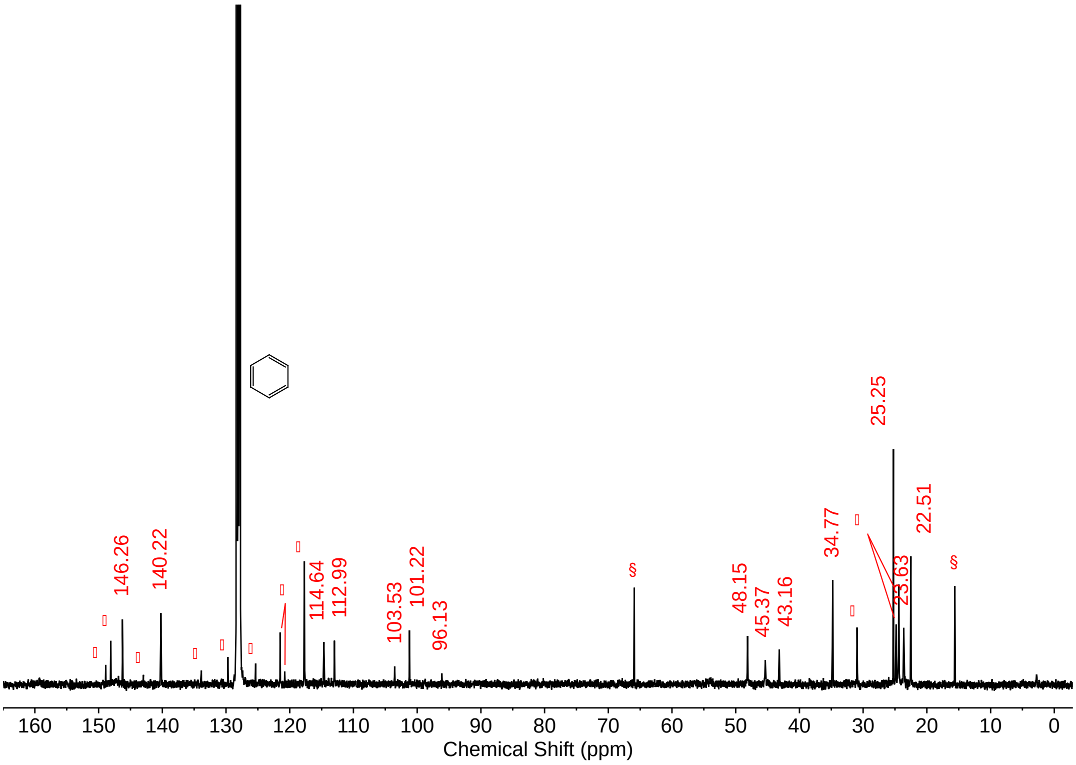

Figure S32. ^{13}C{^{1}H} NMR spectrum of [Sm(NHAr^{iPr6})$_2$] (**2Sm**) in d_6-benzene from +345 to –345 ppm, showing the only observable peaks. § denotes residual Et_2O from crystallization, and ★ denotes trace NH_2Ar^{iPr6} impurities.

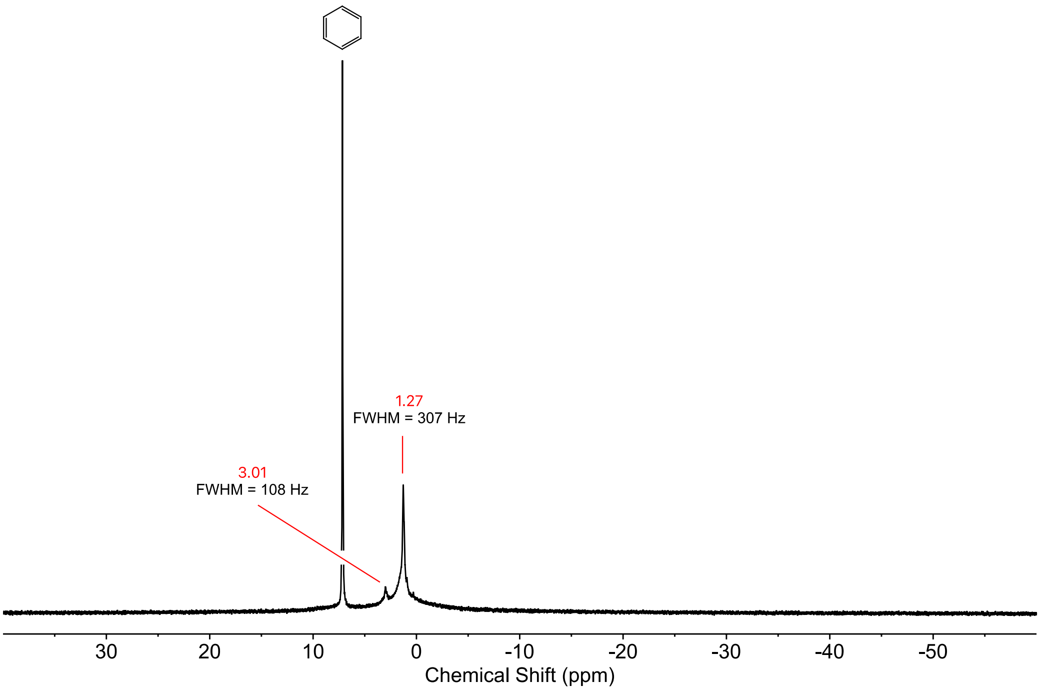

Figure S33. ^{1}H NMR spectrum of [Eu(NHAr^{iPr6})$_2$] (**2Eu**) in d_6-benzene from +40 to –60 ppm, showing the only observable peaks.

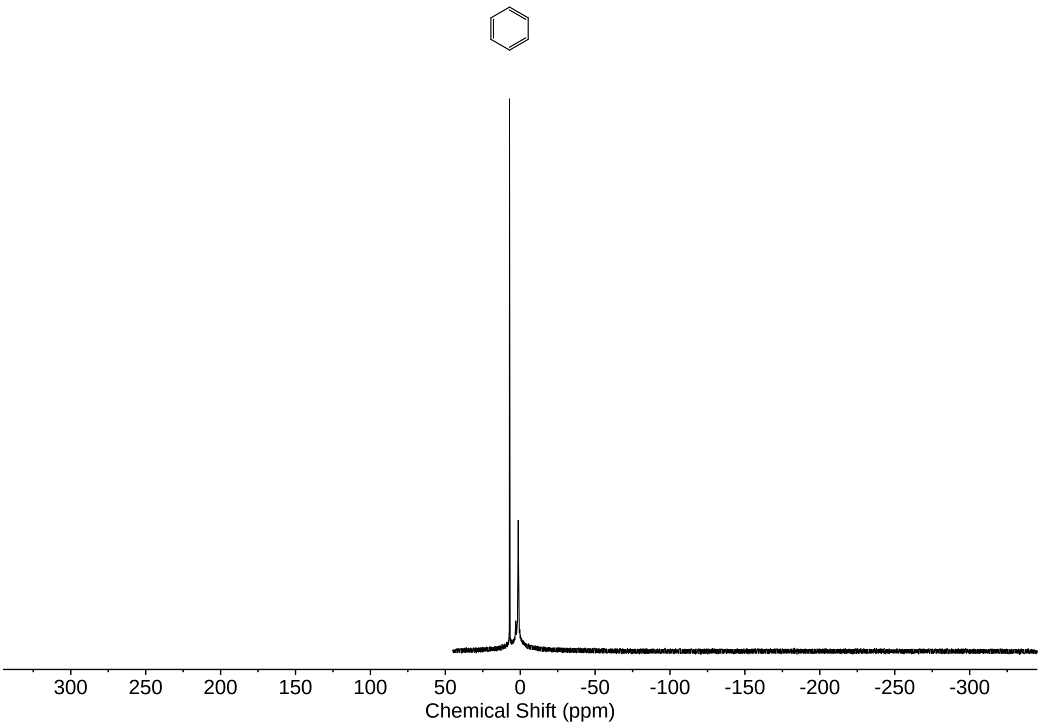

Figure S34. ^{1}H NMR spectra of [Eu(NHAr^{iPr6})$_2$] (**2Eu**) in d_6-benzene from +345 to –345 ppm, showing the only observable peaks.

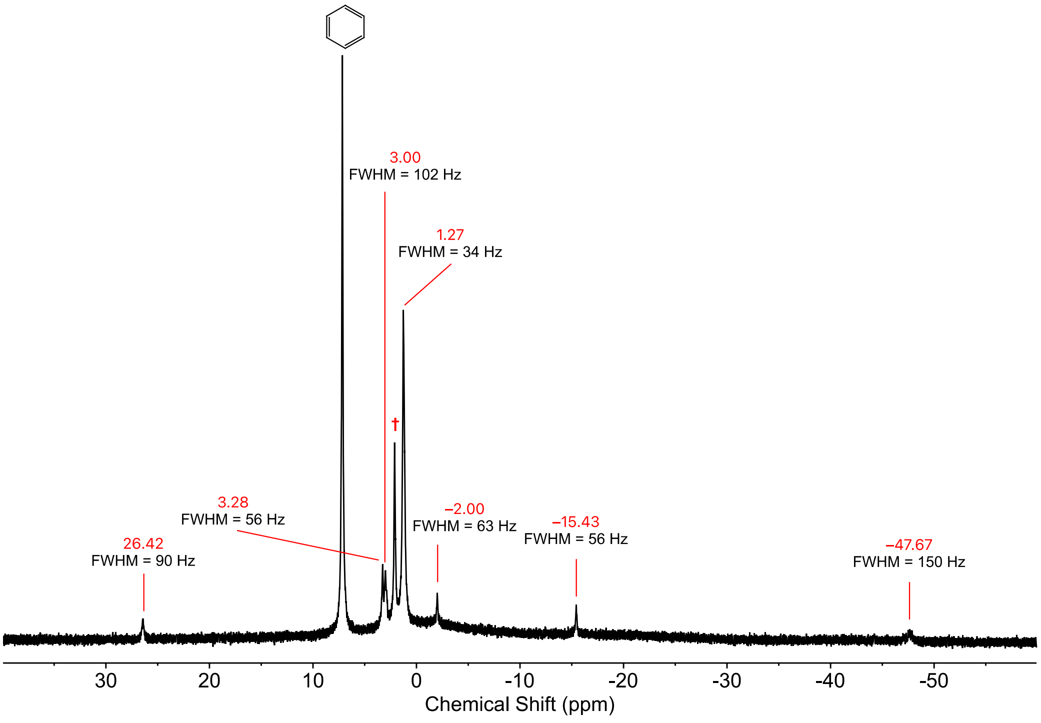

Figure S35. ^{1}H NMR spectrum of [Tm(NHAr^{iPr6})$_2$] (**2Tm**) in d_6-benzene from +40 to –60 ppm, showing the only observable peaks.

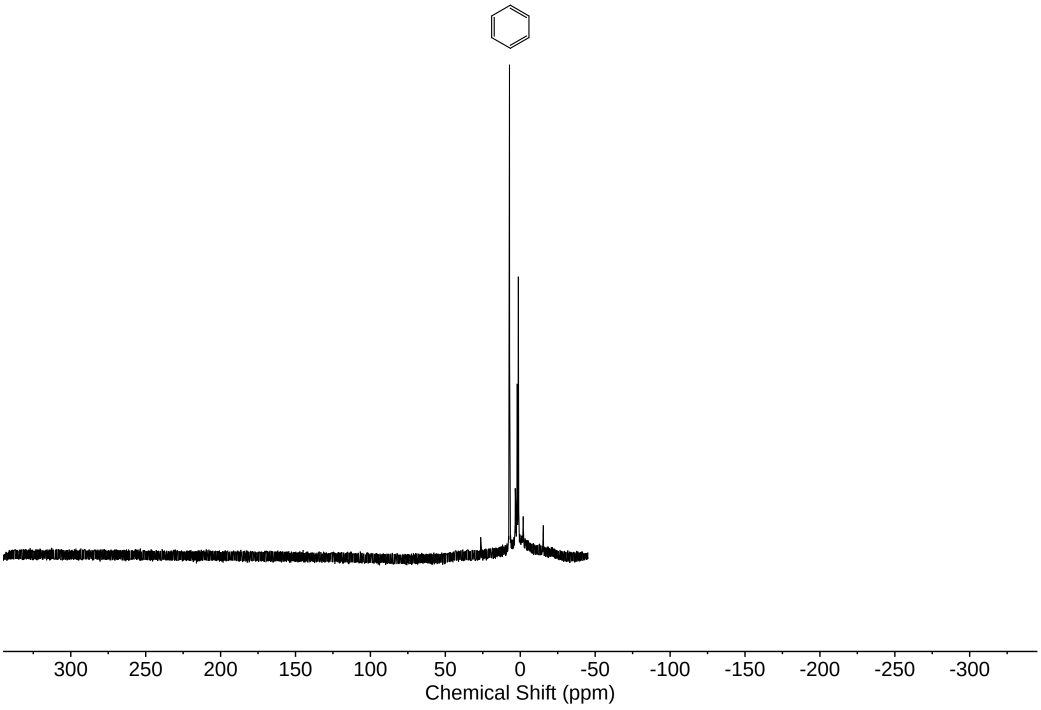

Figure S36. ^{1}H NMR spectra of [Tm(NHAr^{iPr6})$_2$] (**2Tm**) in d_6-benzene from +345 to –345 ppm, showing the only observable peaks.

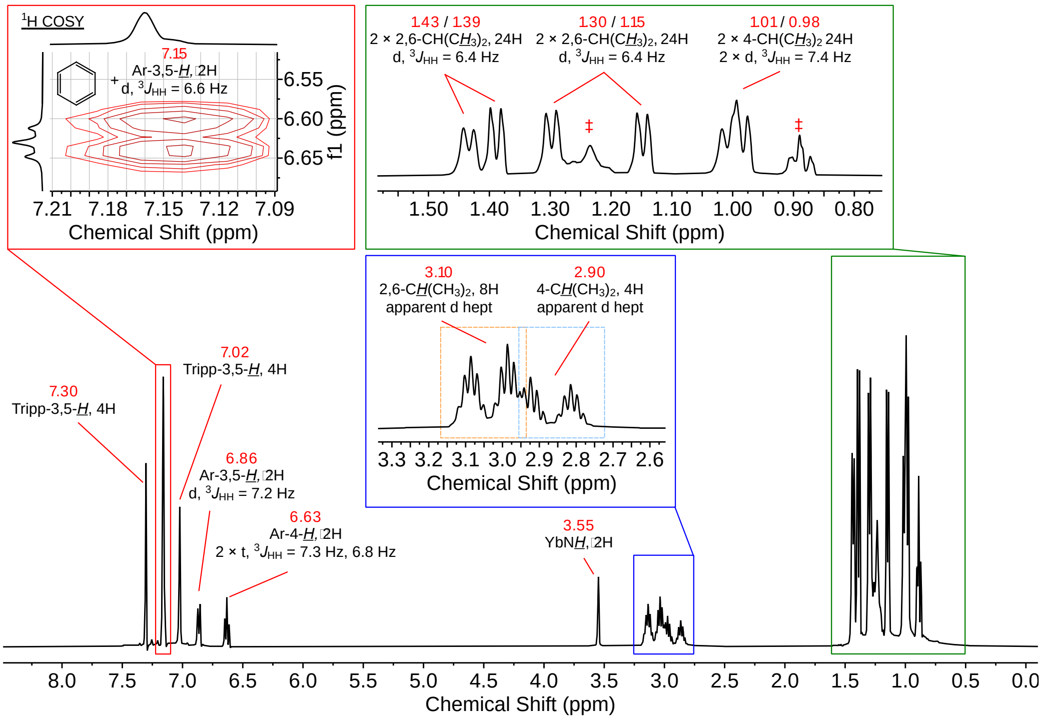

Figure S37. ^{1}H NMR spectrum of $[Yb(NHAr^{iPr6})_2]$ (**2Yb**) in *d*6-benzene. ‡ denotes residual *n*-hexane. Exponential (–3.0 Hz) and Gaussian (2.0 Hz) line broadening were applied.

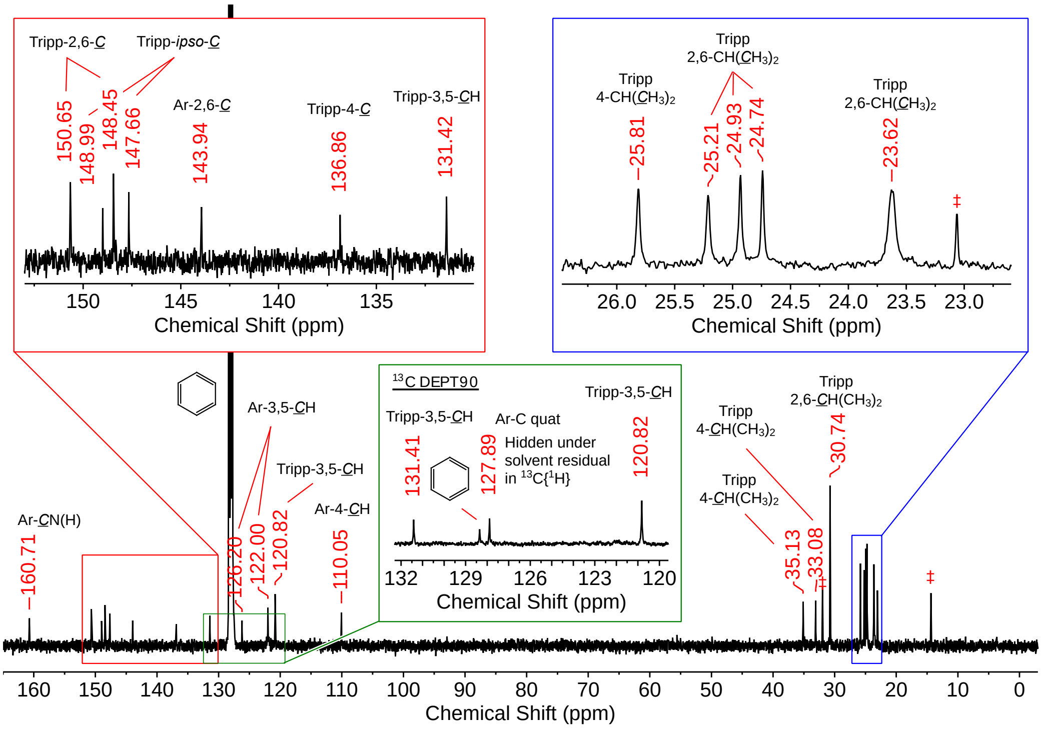

Figure S38. $^{13}C\{^{1}H\}$ NMR spectrum of $[Yb(NHAr^{iPr6})_2]$ (**2Yb**) in *d*6-benzene. ‡ denotes residual *n*-hexane. The central inset shows a section from a ^{13}C DEPT90 experiment to reveal the quaternary Ar-C atom resonance coincides with the ^{13}C chemical shift for *d*6-benzene.

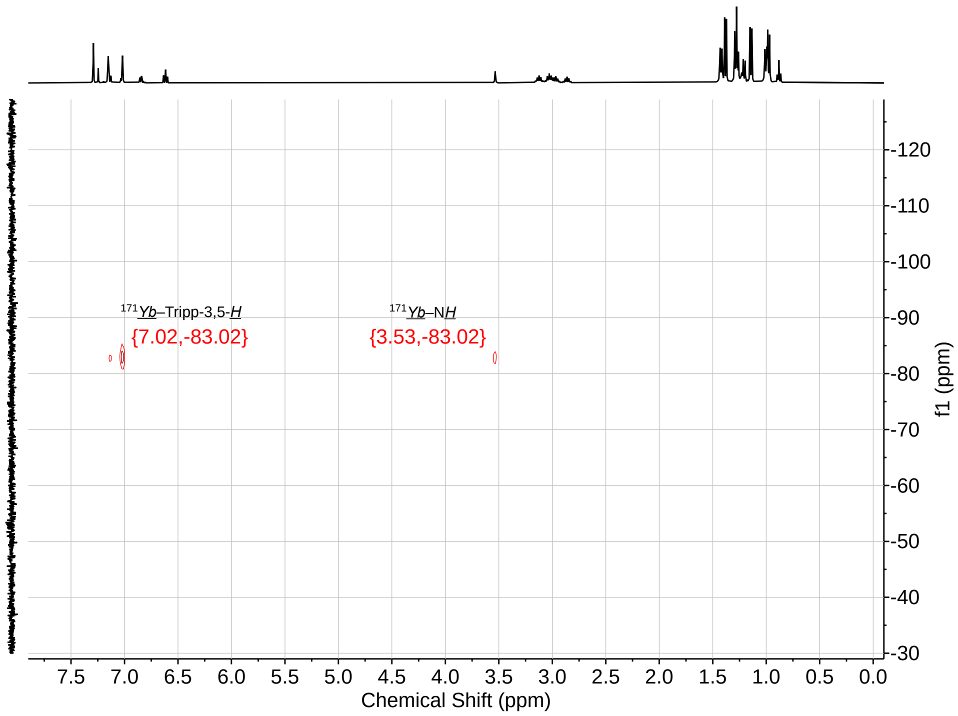

Figure S39. ^{1}H-^{171}Yb HMBC NMR spectrum of [Yb(NHAr^{iPr6})$_2$] (**2Yb**) in d_6-benzene. Exponential (–3.0 Hz) and Gaussian (2.0 Hz) line broadening were applied to both domains.

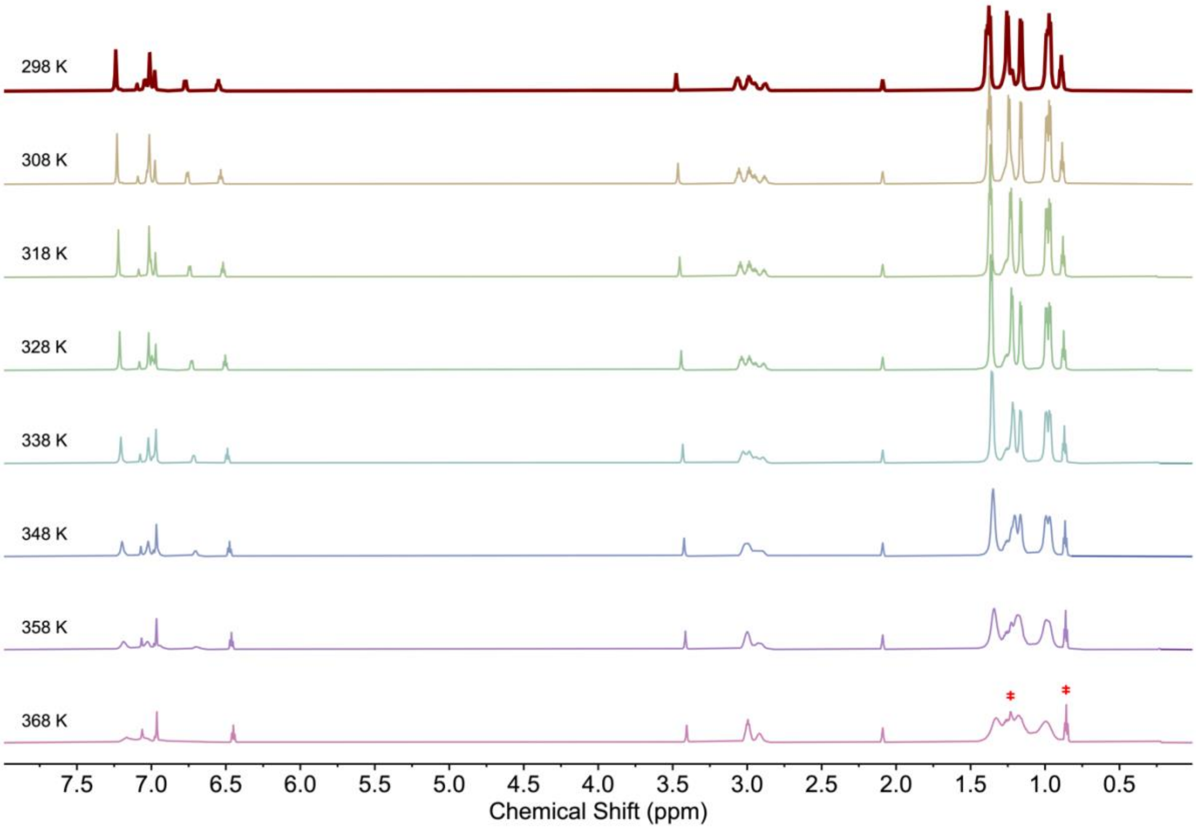

Figure S40. Variable-temperature ^{1}H NMR spectra of [Yb(NHAr^{iPr6})$_2$] (**2Yb**) in d_8-toluene showing the full range of observed peaks. ‡ denotes residual *n*-hexane.

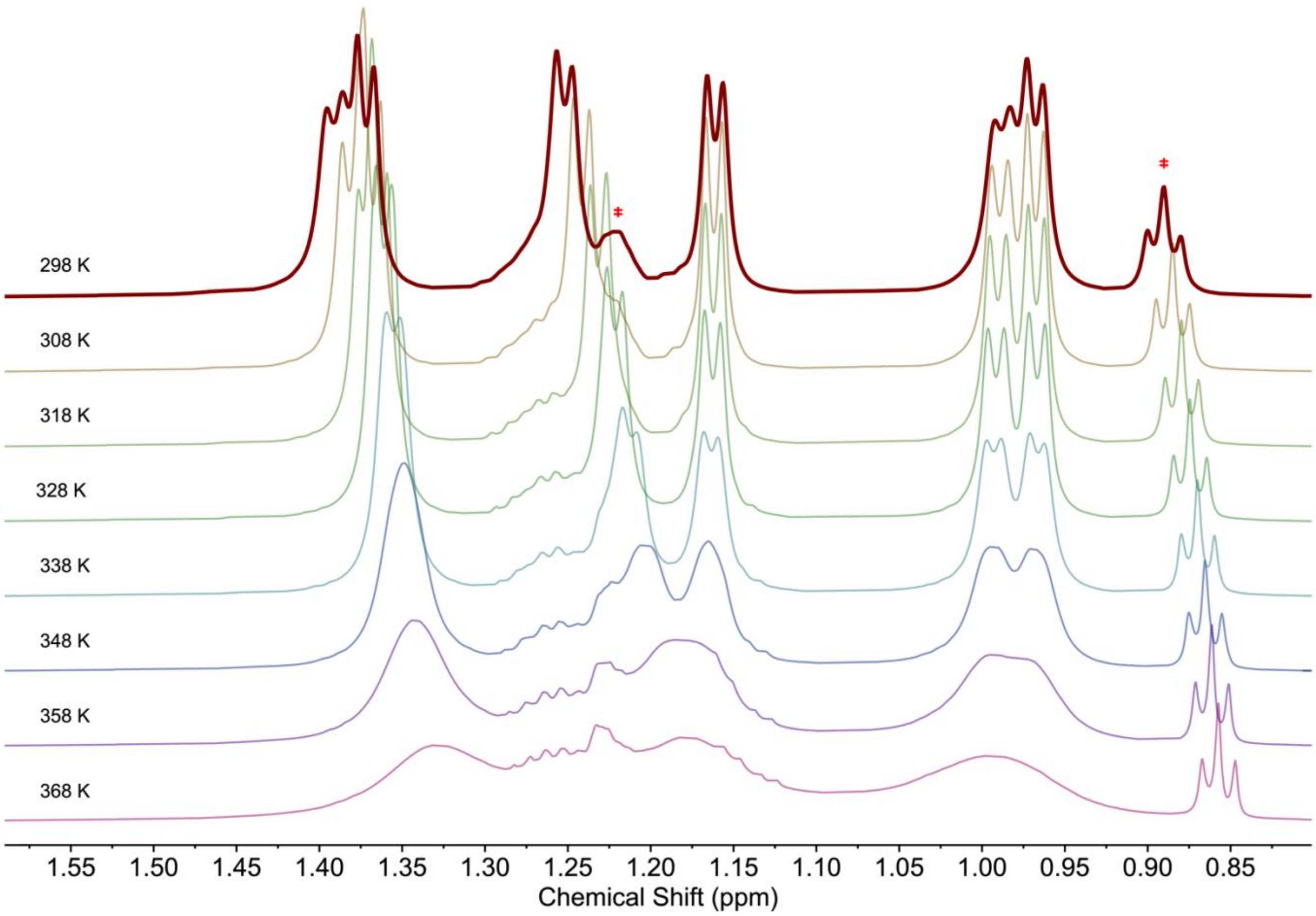

Figure S41. Variable-temperature ^{1}H NMR spectra of [Yb(NHAr^{iPr6})$_2$] (**2Yb**) in d_8-toluene showing the region between 1.60 ppm to 0.80 ppm, which shows that between 298 K and 368 K, the bound and terminal Tripp-*i*Pr groups are in dynamic exchange. ‡ denotes residual *n*-hexane.

Magnetic moments determined by NMR spectroscopy (Evans method)

Table S11. Data for the determination of the magnetic moments of complexes **1Tm** and **2M** (M = Sm, Eu, Tm).

Sample / peak	μ_{eff} / B.M mol^{-1}	mass of sample / g [A]	mass of solvent / g	M_r / g mol^{-1}	Δ peak / Hz [B]
**1Tm** [Tm(NHAr^{iPr6})$_2$(I)]	6.06(2)	0.0174	0.5813	1289.41	553.74
**2Sm** [Sm(NHAr^{iPr6})$_2$]	3.12(1)	0.0175	0.5338	1143.93	163.07
**2Eu** [Eu(NHAr^{iPr6})$_2$]	7.37(2)	0.0215	0.05813	1145.54	1156.00
**2Tm** [Tm(NHAr^{iPr6})$_2$]	3.51(1)	0.0173	0.6248	1162.51	176.38

[A] The small masses engender large errors in this methodology, the results should be cautiously interpreted along with other data. [B] Spectrometer frequency 400.130 MHz. Diamagnetic correction of M_r / −2,000,000 applied. ρd_6-benzene = 0.950 g mL^{-1}.

S5. ATR-IR spectra

*ATR-IR spectra of **1M** (M = Sc, Y, La, Tm, Yb) and **2M** (M = Sc, Y, La, Sm, Eu, Tm, Yb)*

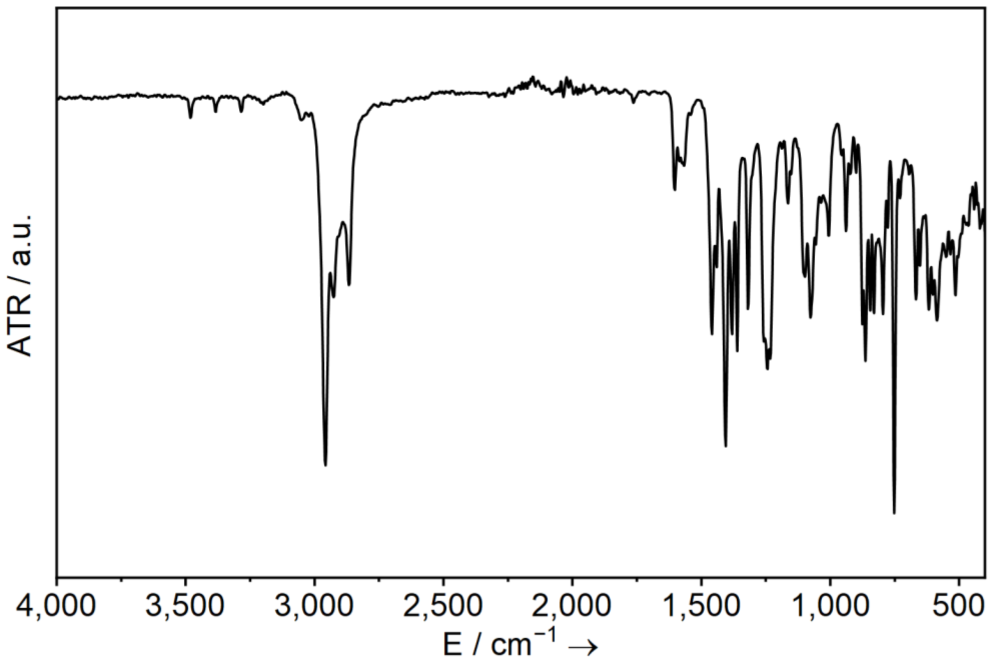

Figure S42. Crystalline ATR-IR spectrum of [Sc(NHAr^{iPr6})$_2$(I)] (**1Sc**).

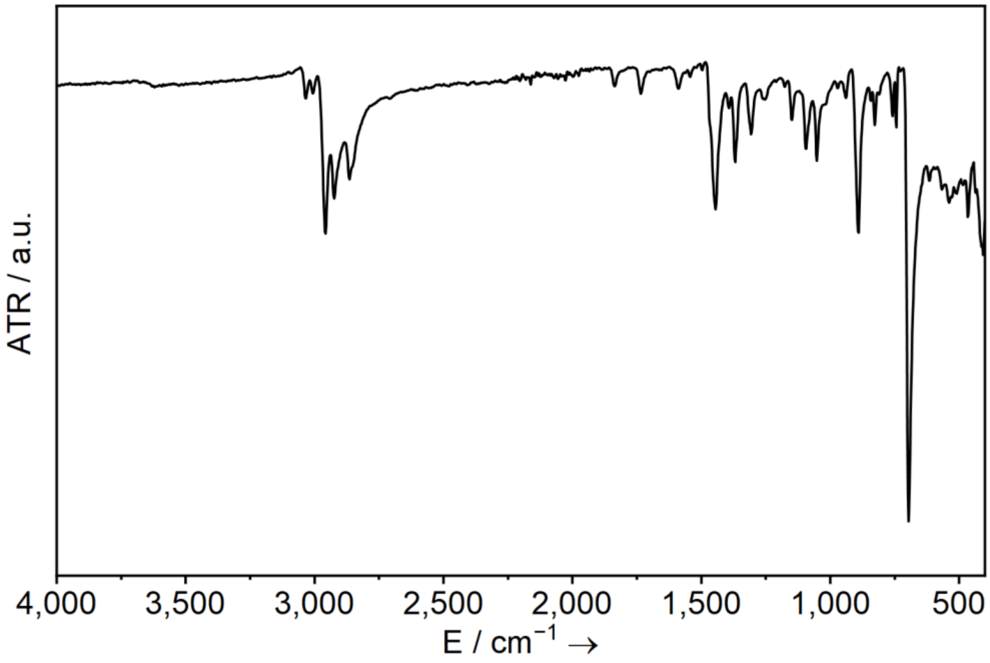

Figure S43. Crystalline ATR-IR spectrum of [Y(NHAr^{iPr6})$_2$(I)] (**1Y**).

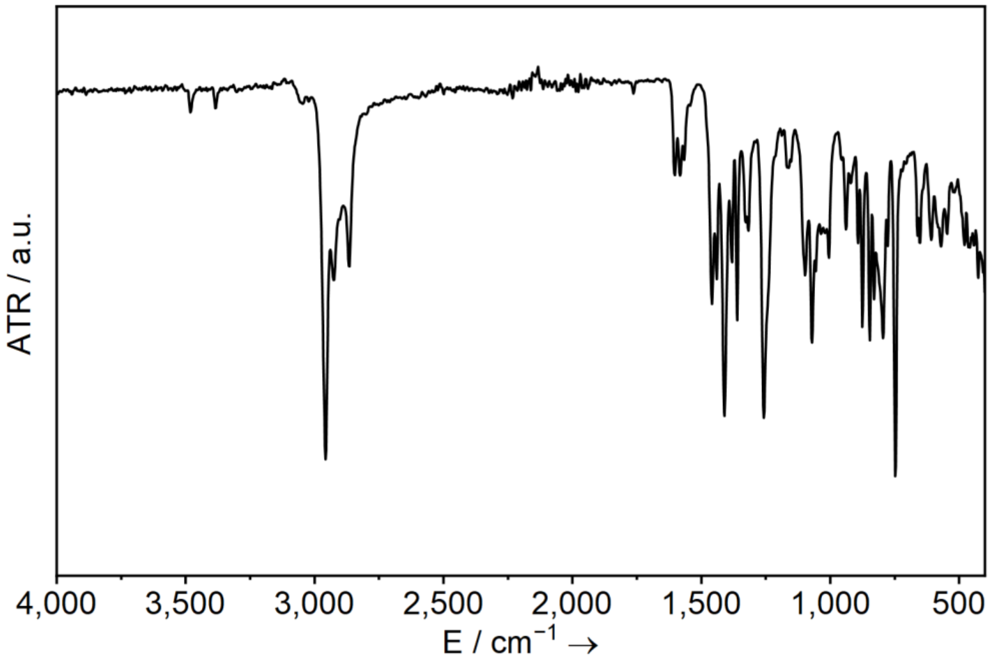

Figure S44. Crystalline ATR-IR spectrum of [La(NHAr^{iPr6})$_2$(I)] (**1La**).

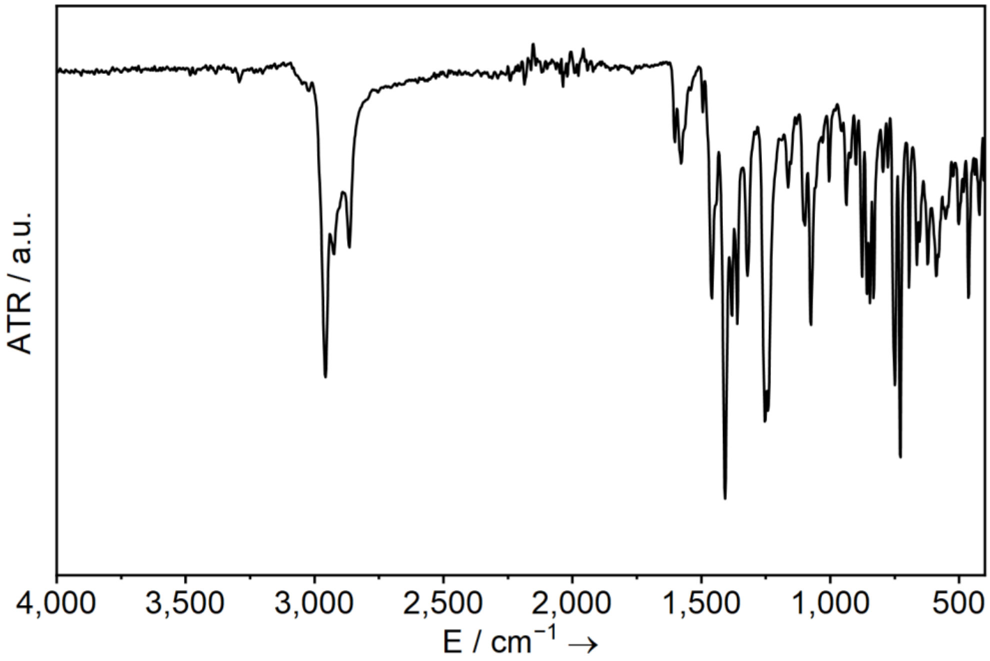

Figure S45. Crystalline ATR-IR spectrum of [Tm(NHAr^{iPr6})$_2$(I)] (**1Tm**).

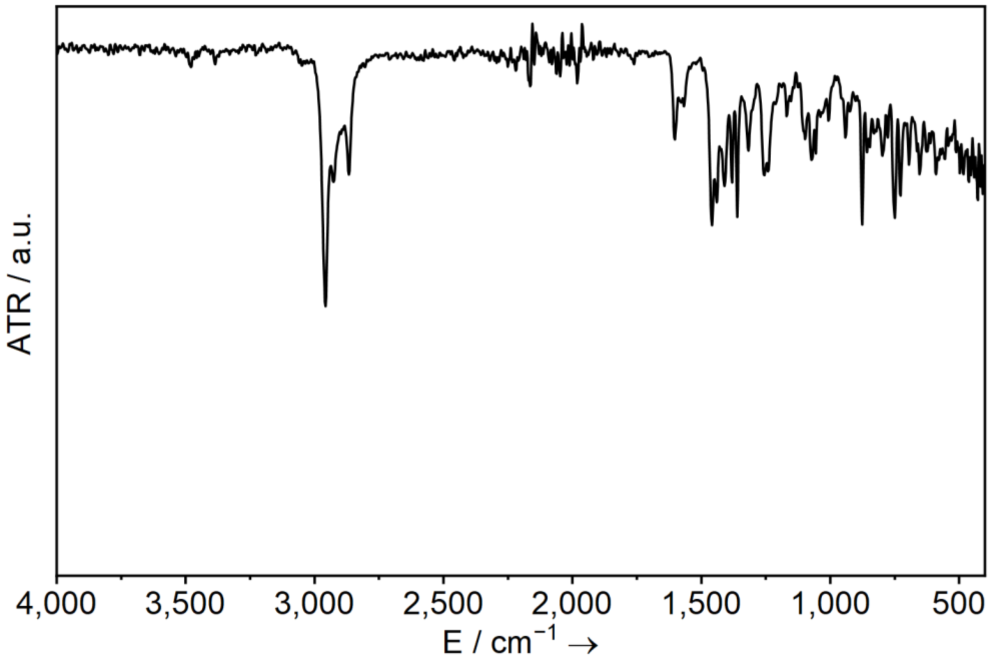

Figure S46. Crystalline ATR-IR spectrum of [Yb(NHAr^{iPr6})$_2$(I)] (**1Yb**).

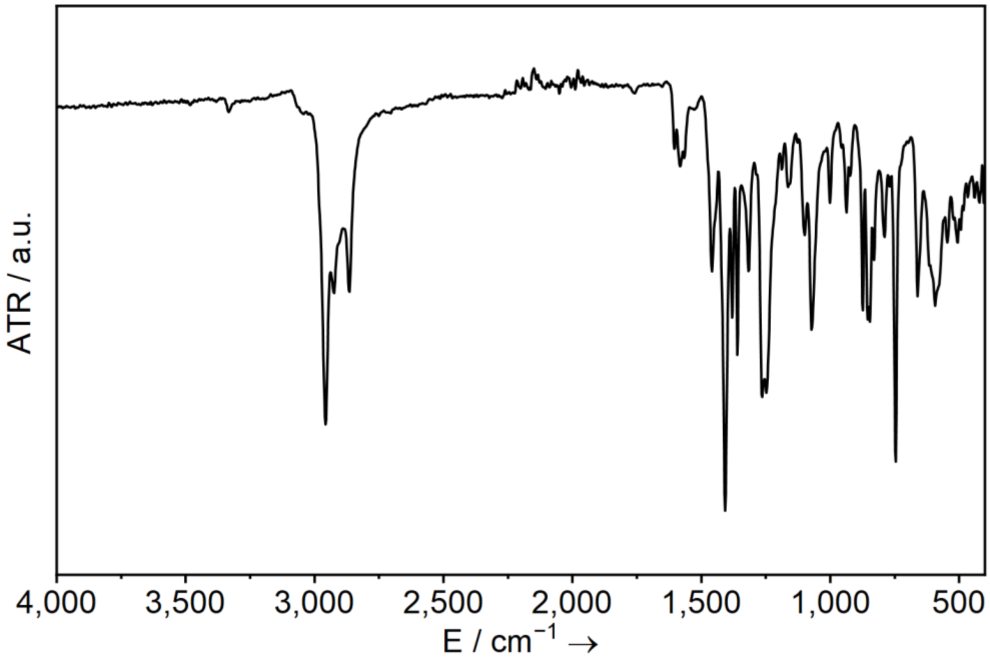

Figure S47. Crystalline ATR-IR spectrum of [Sc(NHAr^{iPr6})$_2$] (**2Sc**).

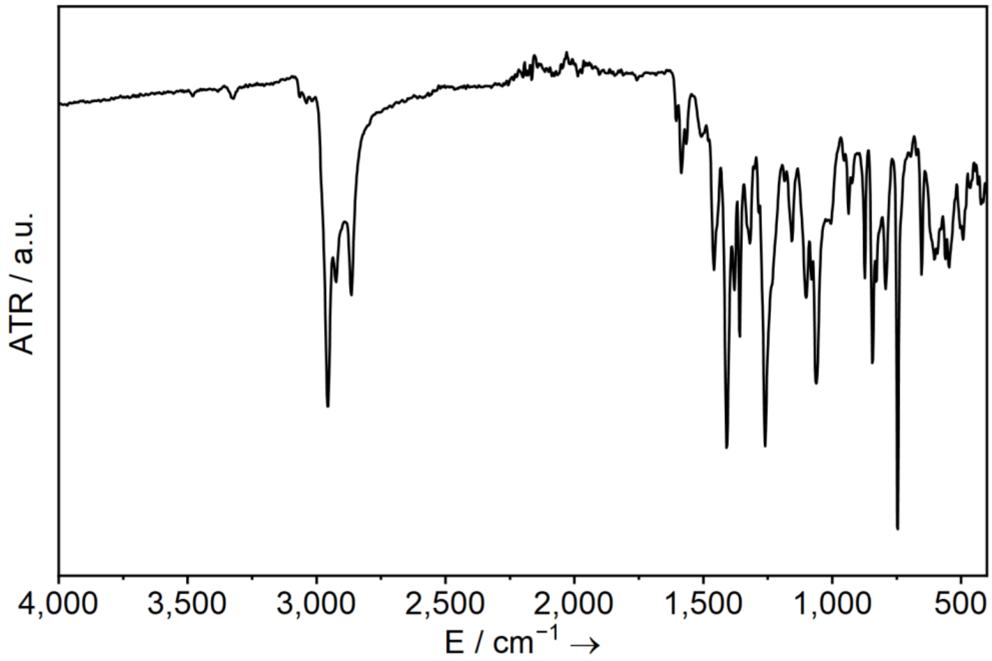

Figure S48. Crystalline ATR-IR spectrum of [Y(NHAr^{iPr6})$_2$] (**2Y**).

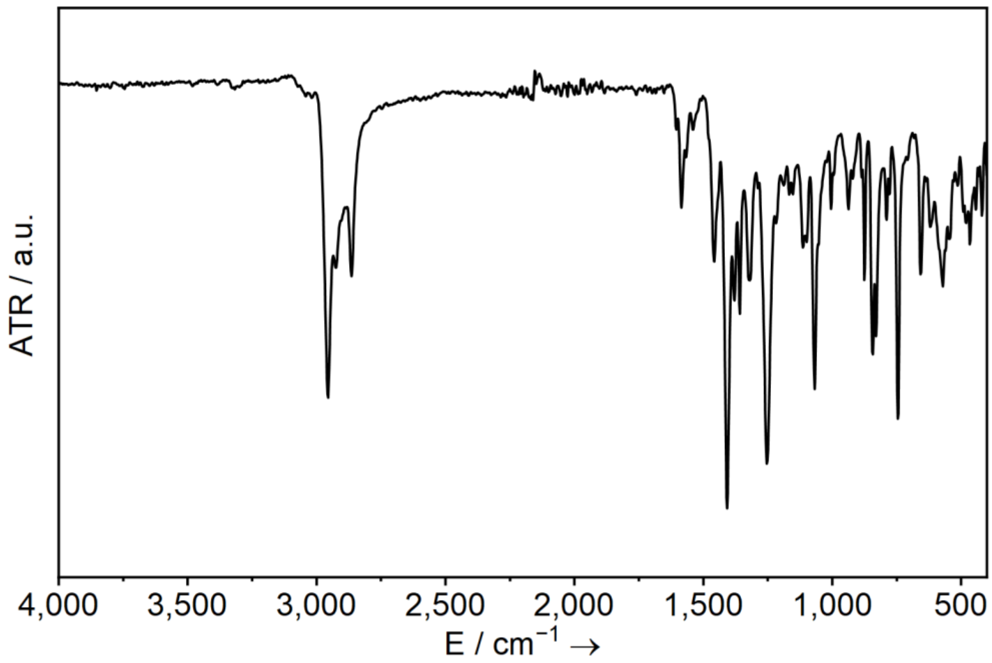

Figure S49. Crystalline ATR-IR spectrum of [La(NHAr^{iPr6})$_2$] (**2La**).

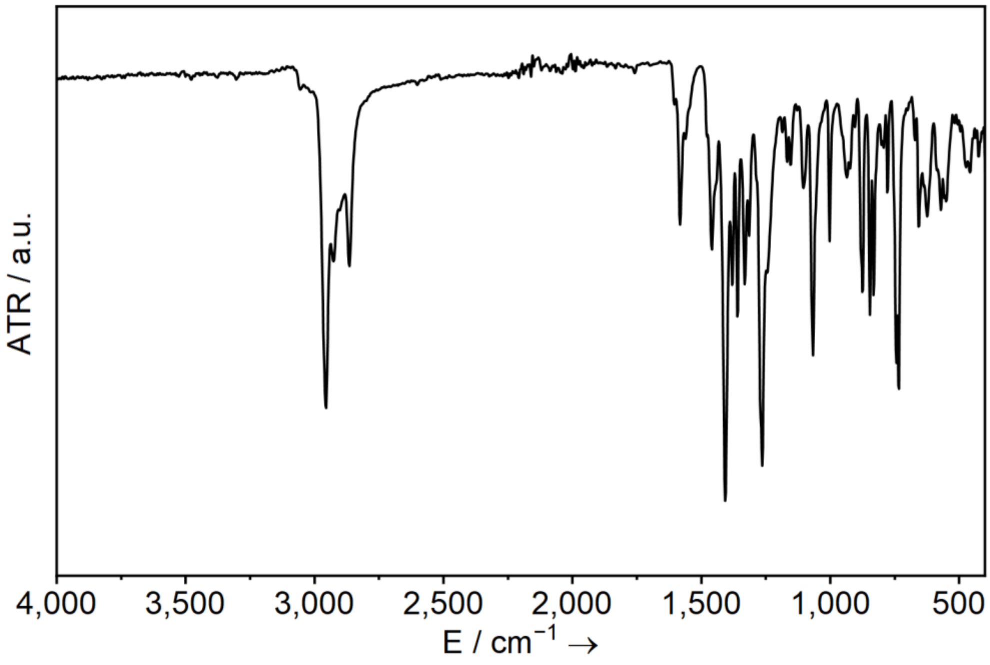

Figure S50. Crystalline ATR-IR spectrum of [Sm(NHAr$^{i\mathrm{Pr6}}$)$_2$] (**2Sm**).

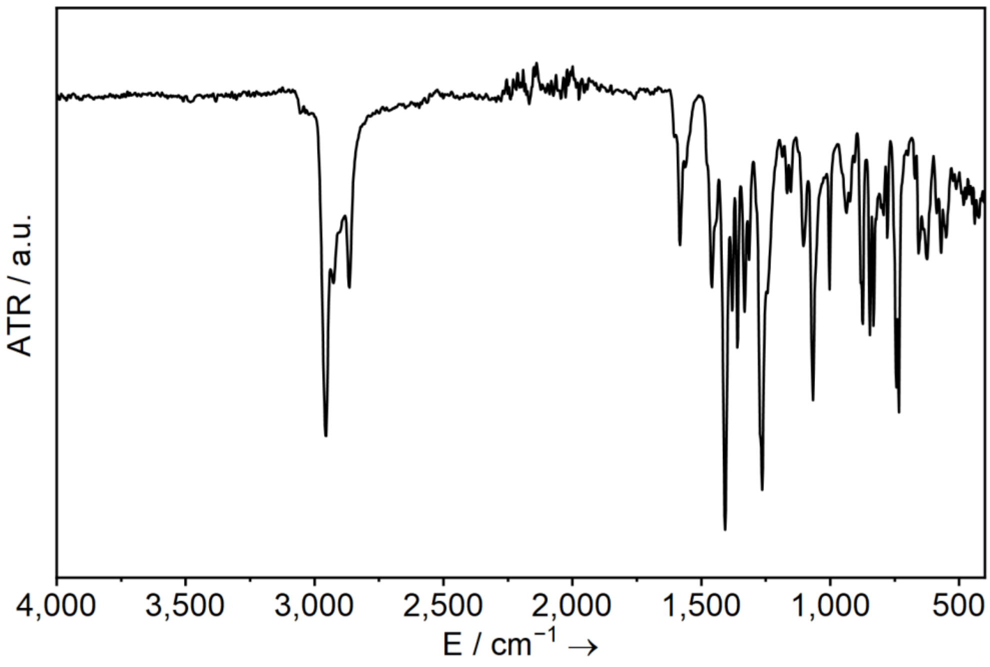

Figure S51. Crystalline ATR-IR spectrum of [Eu(NHAr$^{i\mathrm{Pr6}}$)$_2$] (**2Eu**).

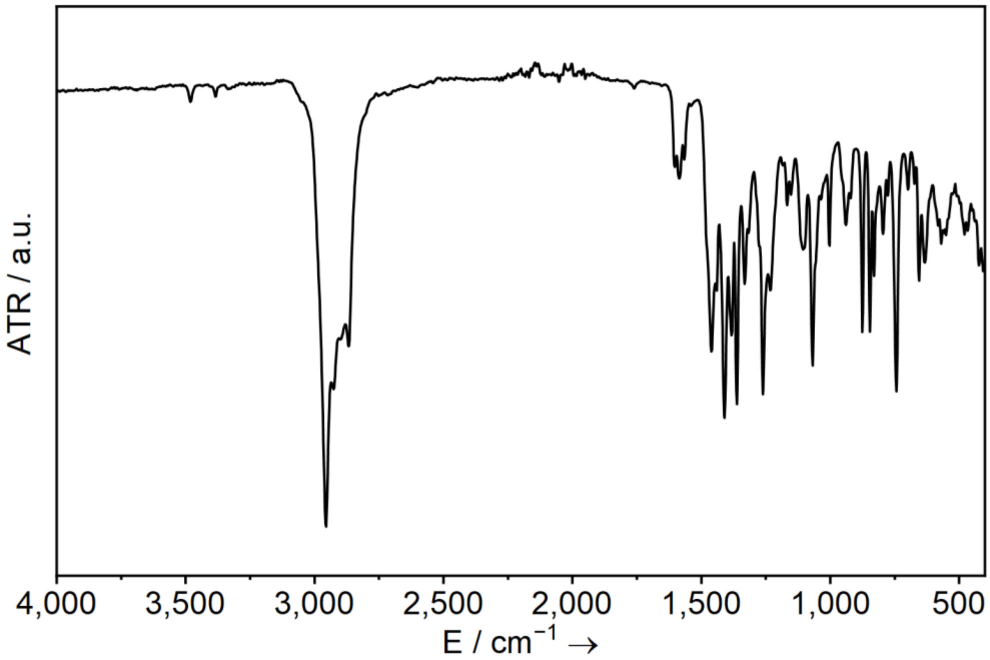

Figure S52. Crystalline ATR-IR spectrum of [Tm(NHAr^{iPr6})$_2$] (**2Tm**).

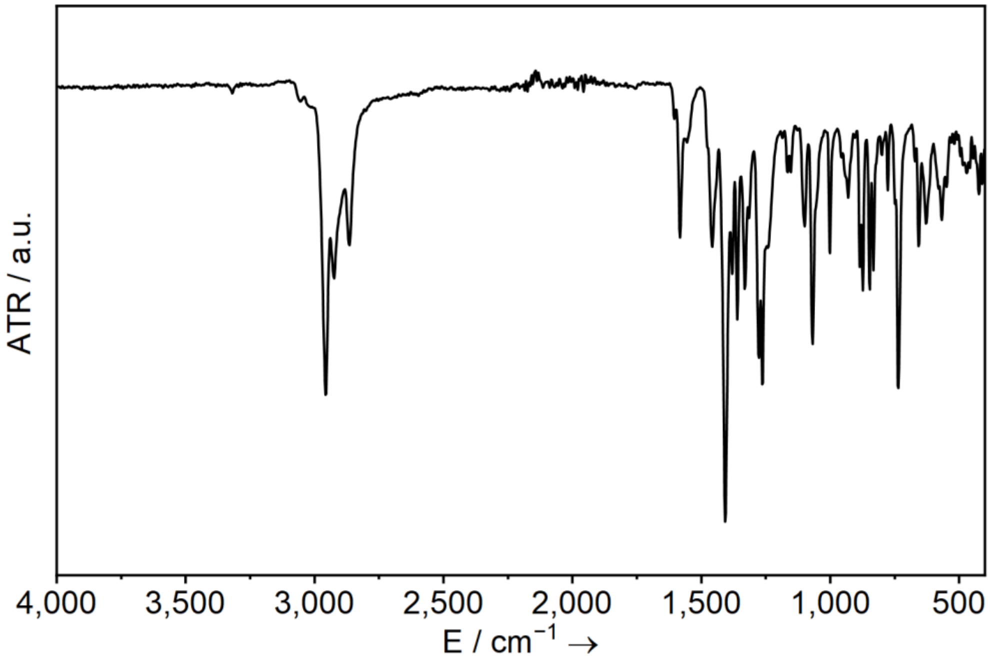

Figure S53. Crystalline ATR-IR spectrum of [Yb(NHAr^{iPr6})$_2$] (**2Yb**).

*Comparative ATR-IR spectra for **1M** (M = Sc, Y, La) and **2M** (M = Sc, Y, La, Sm, Eu, Tm, Yb)*

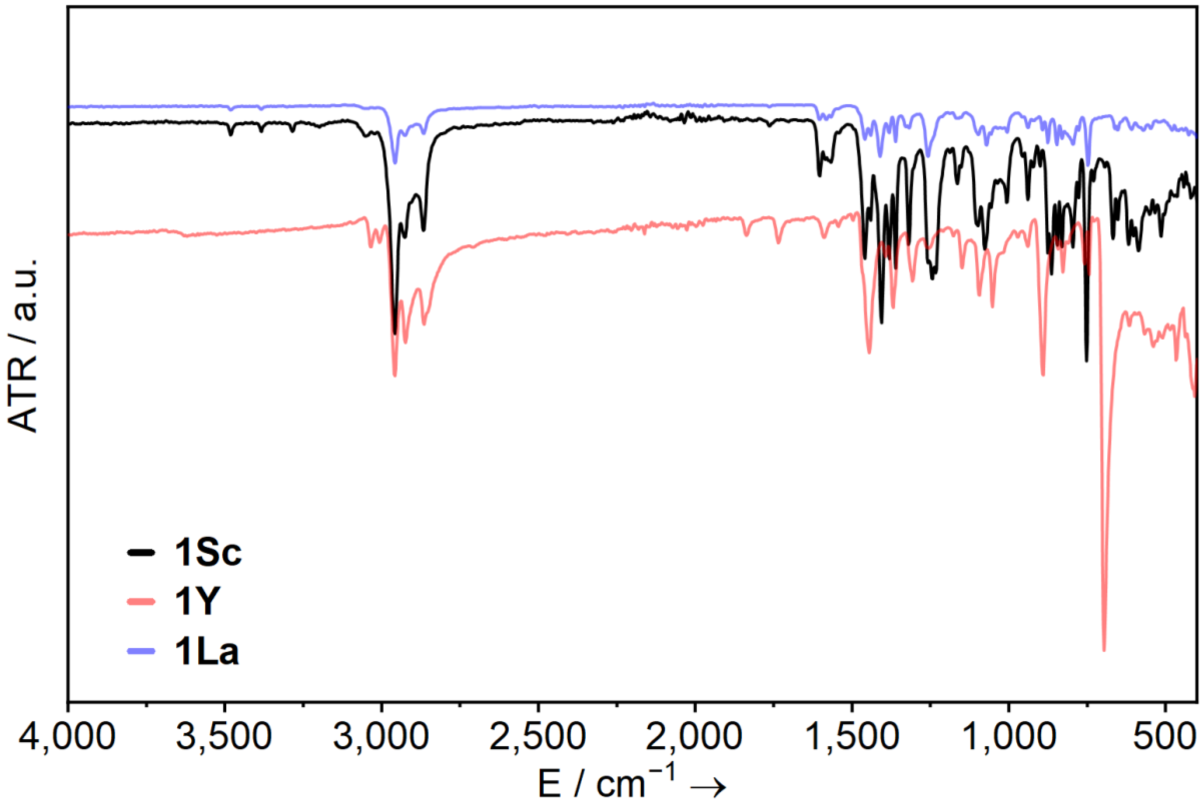

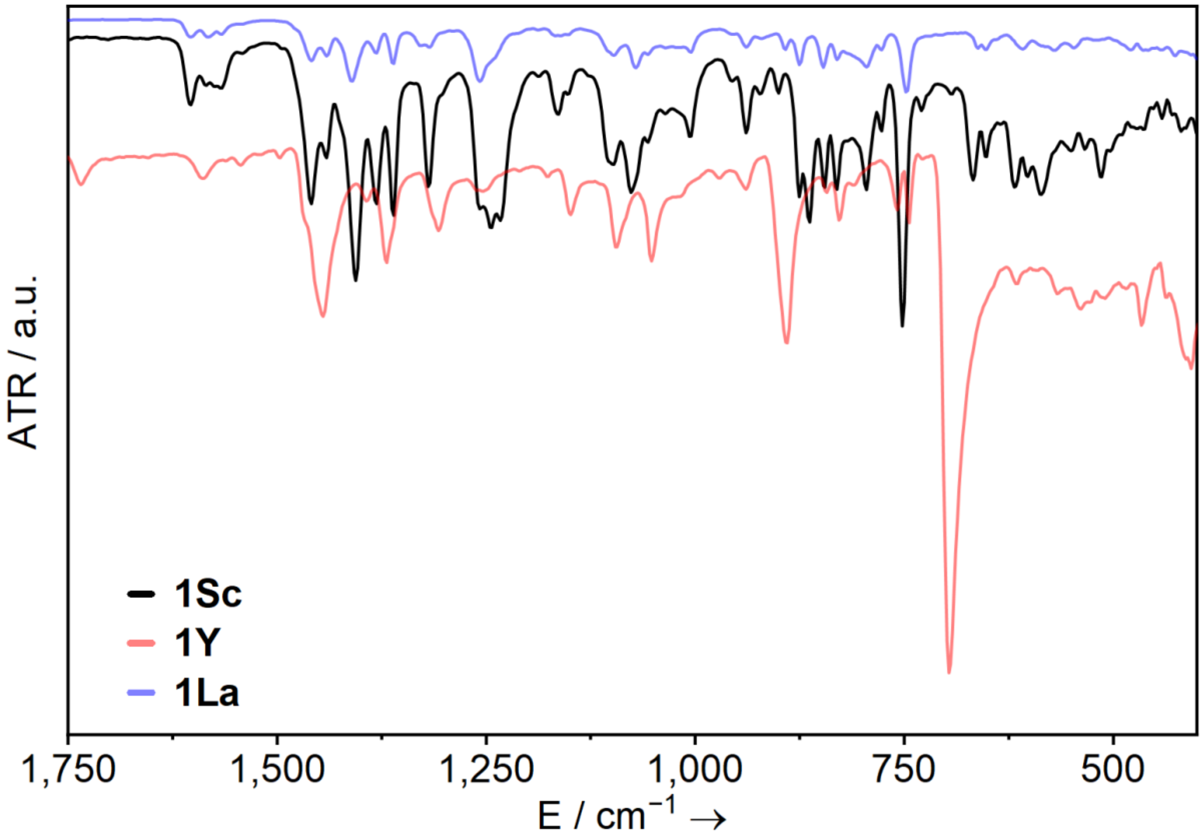

Figure S54. Combined ATR-IR spectra of **1M** (M = Sc, black; Y, red; La, blue). The top spectrum shows the full collected range (4,000–400 cm^{-1}), the bottom spectrum shows between 1,750–400 cm^{-1}.

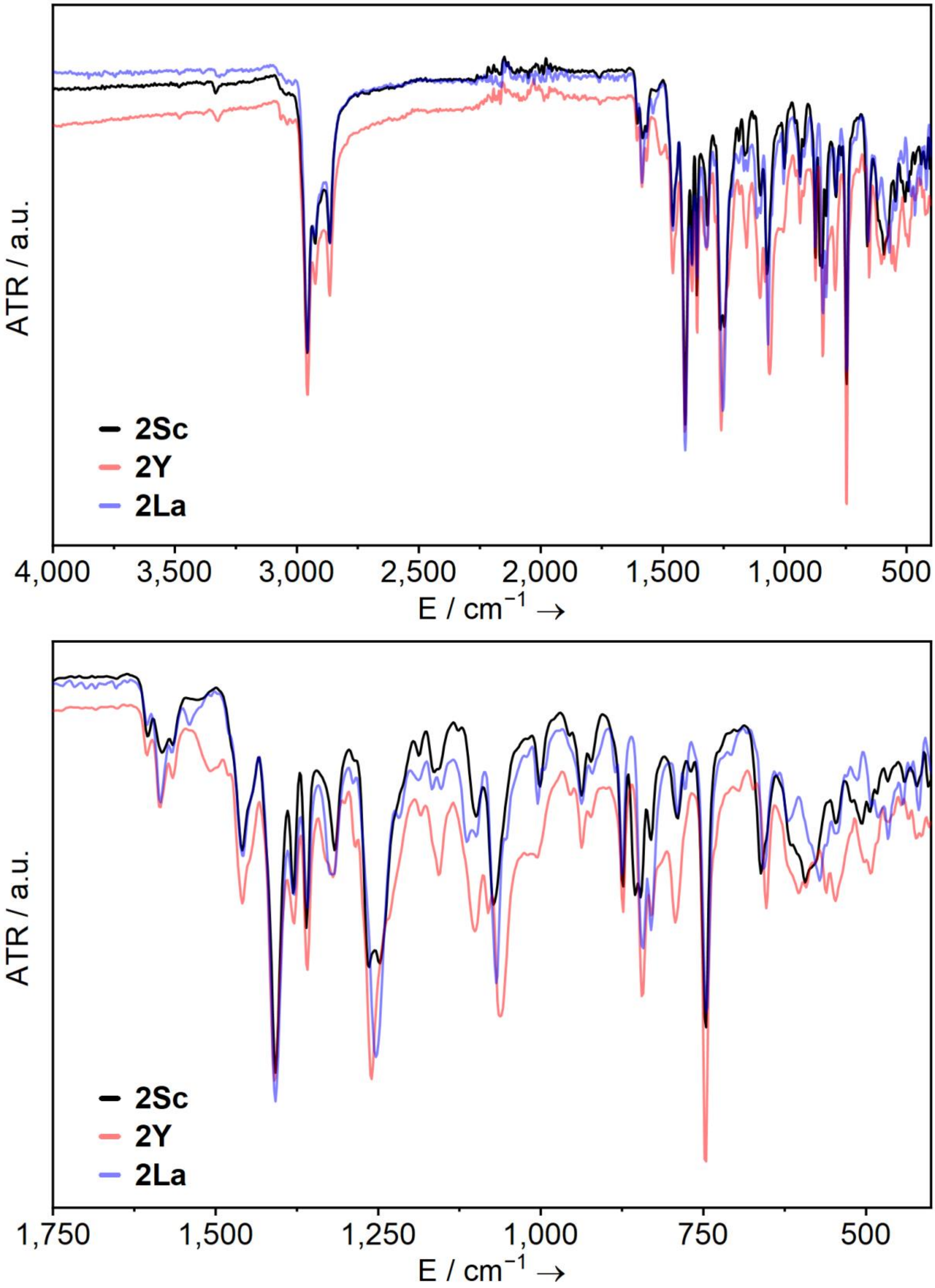

Figure S55. Combined ATR-IR spectra of **2M** (M = Sc, black; Y, red; La, blue). The top spectrum shows the full collected range (4,000–400 cm^{-1}), the bottom spectrum shows between 1,750–400 cm^{-1}.

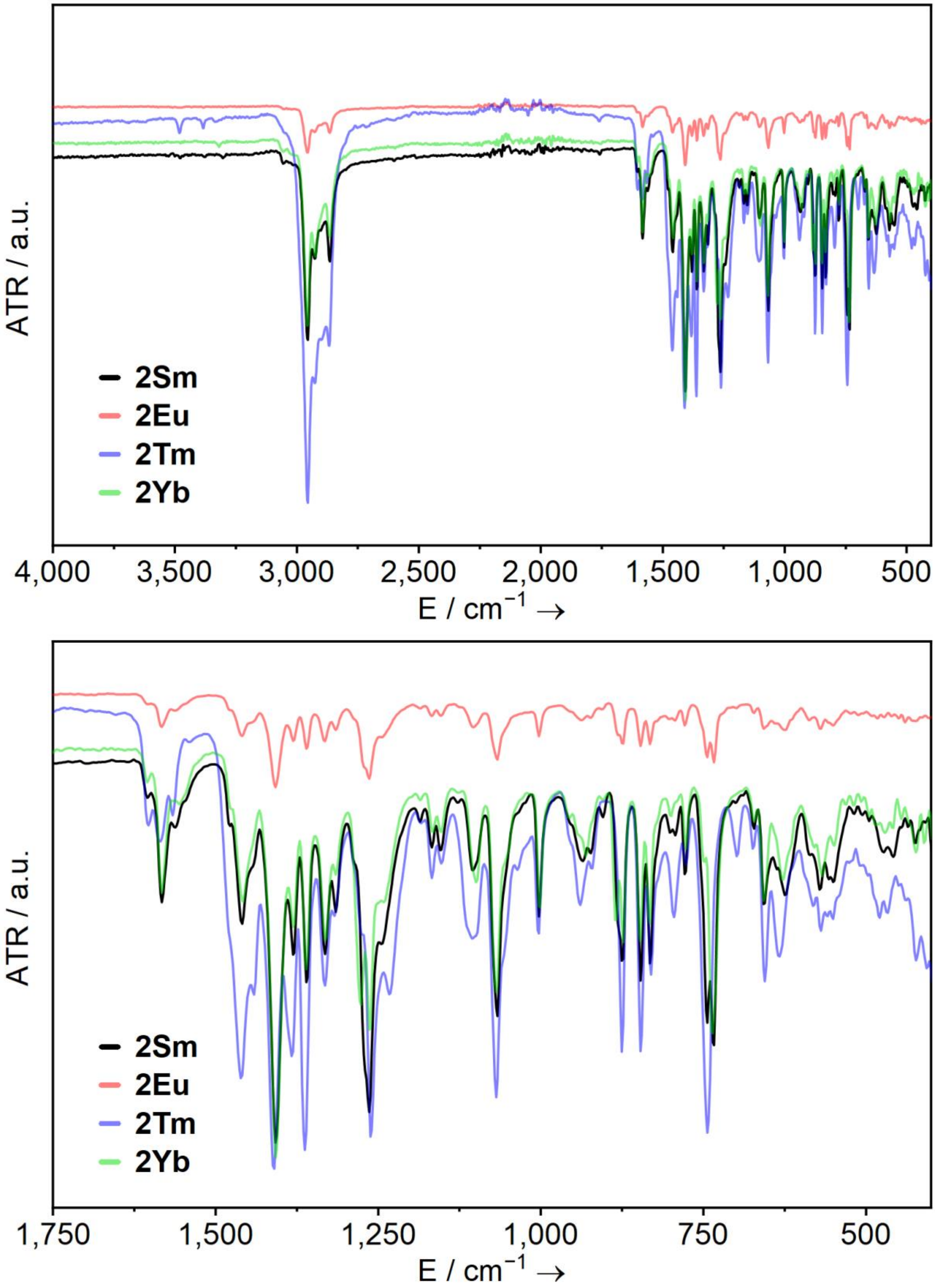

Figure S56. Combined ATR-IR spectra of **2M** (M = Sm, black; Eu, red; Tm, blue; Yb, green). The top spectrum shows the full collected range (4,000–400 cm^{-1}), the bottom spectrum shows between 1,750–400 cm^{-1}.

S6. UV-Vis-NIR spectra

*UV-Vis-NIR spectra of **1M** (M = Sc, Y, La, Tm, Yb)*

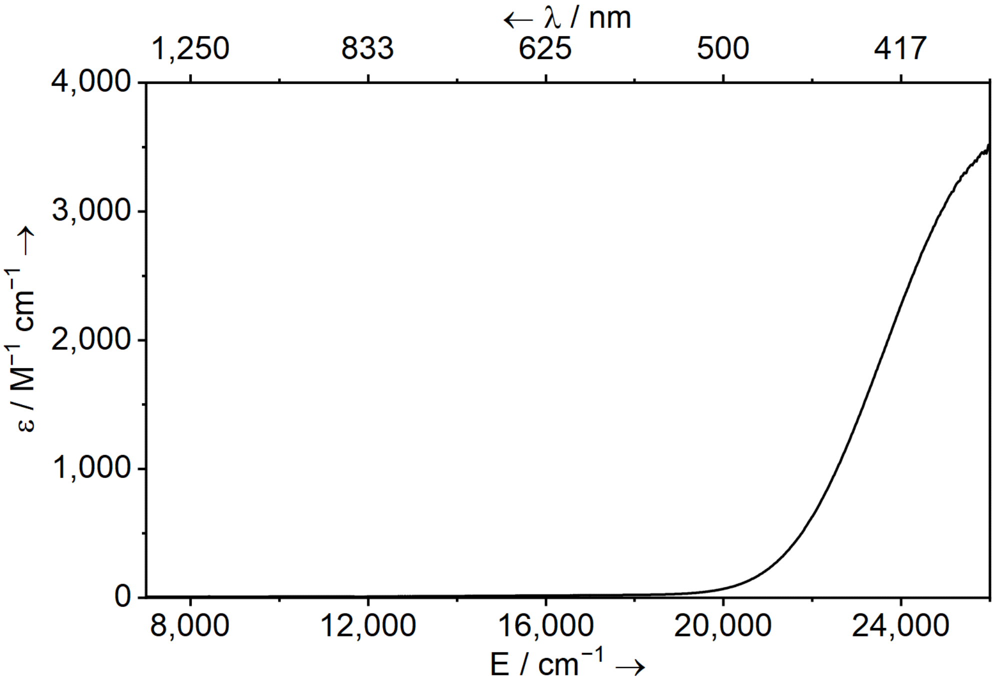

Figure S57. Solution UV-Vis-NIR spectrum of [Sc(NHAr^{iPr6})$_2$(I)] (**1Sc**) (1 mM) in Et_2O shown between 7,000–26,000 cm^{-1} (1,429–385 nm) at ambient temperature.

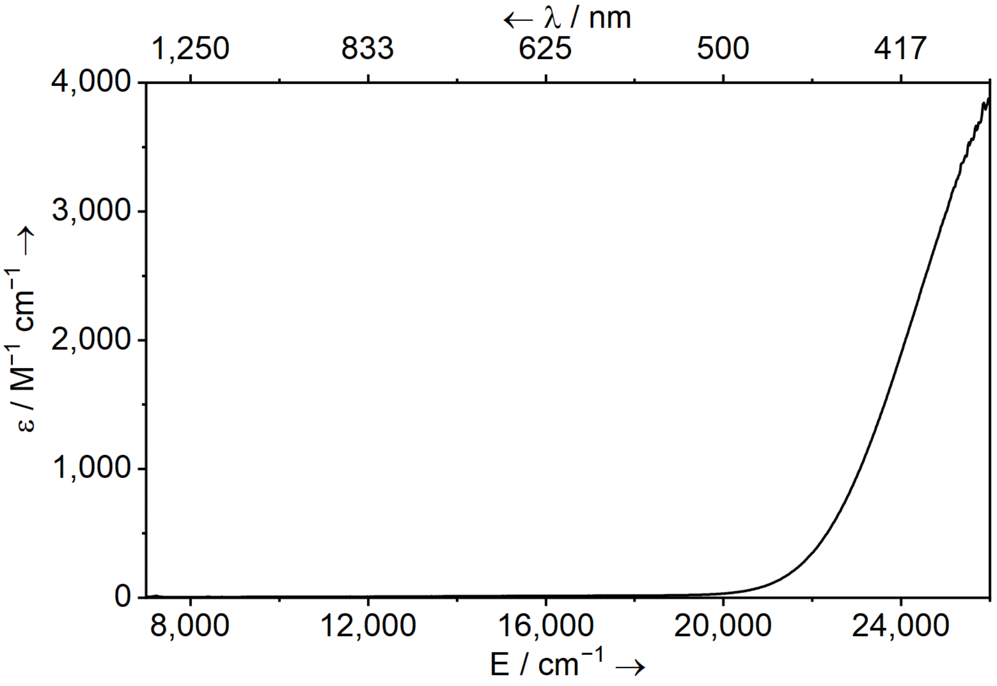

Figure S58. Solution UV-Vis-NIR spectrum of [Y(NHAr^{iPr6})$_2$(I)] (**1Y**) (1 mM) in Et_2O shown between 7,000–26,000 cm^{-1} (1,429–385 nm) at ambient temperature.

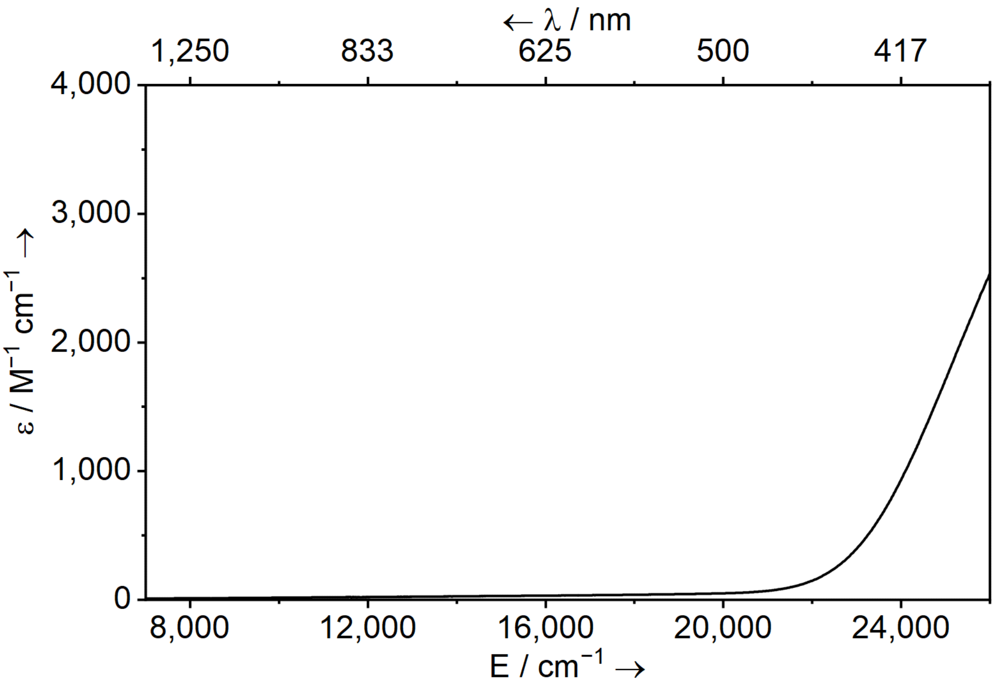

Figure S59. Solution UV-Vis-NIR spectrum of [La(NHAr^{iPr6})$_2$(I)] (**1La**) (1 mM) in Et_2O shown between 7,000–26,000 cm^{-1} (1,429–385 nm) at ambient temperature.

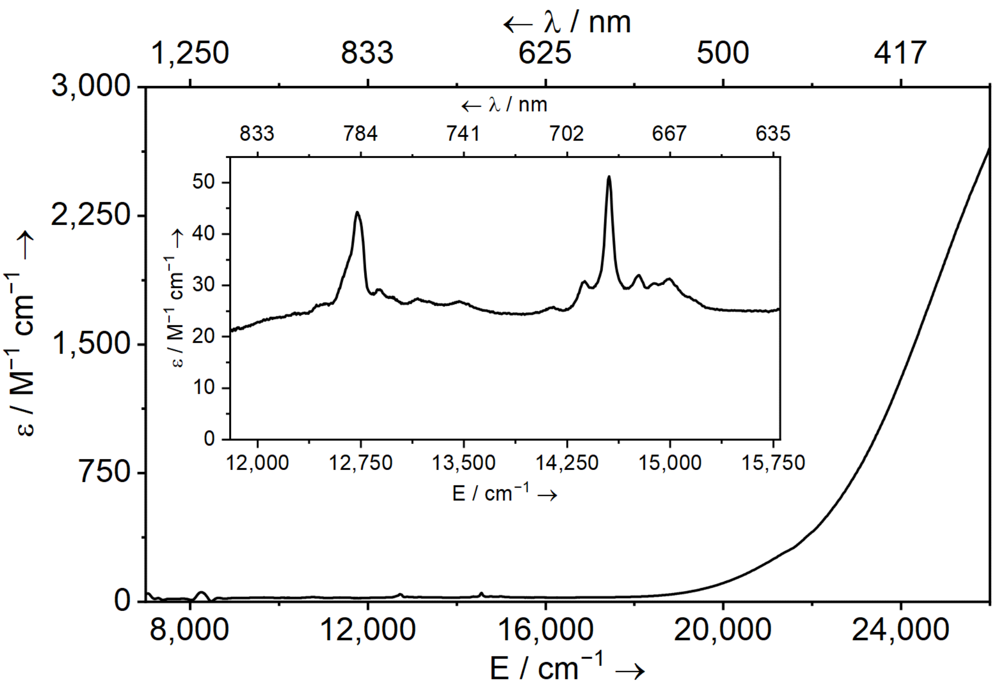

Figure S60. Solution UV-Vis-NIR spectrum of [Tm(NHAr^{iPr6})$_2$(I)] (**1Tm**) (1 mM) in Et_2O shown between 7,000–26,000 cm^{-1} (1,429–385 nm) at ambient temperature.

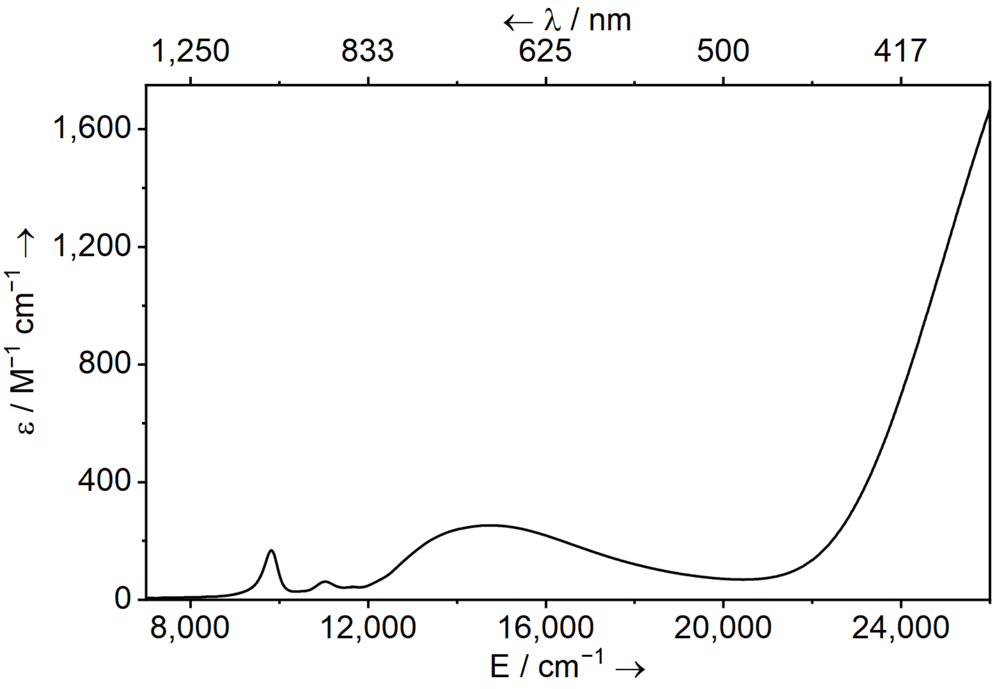

Figure S61. Solution UV-Vis-NIR spectrum of [Yb(NHAr^{iPr6})$_2$(I)] (**1Yb**) (*ca*. 1 mM) in Et_2O shown between 7,000–26,000 cm^{-1} (1,429–385 nm) at ambient temperature. Note that the concentration of this sample is only approximate, we could not isolate **1Yb** in pure form as it consistently co-crystallized with **3**.

UV-Vis-NIR spectra of ***2M*** *(M = Sc, Y, La, Sm, Eu, Tm, Yb)*

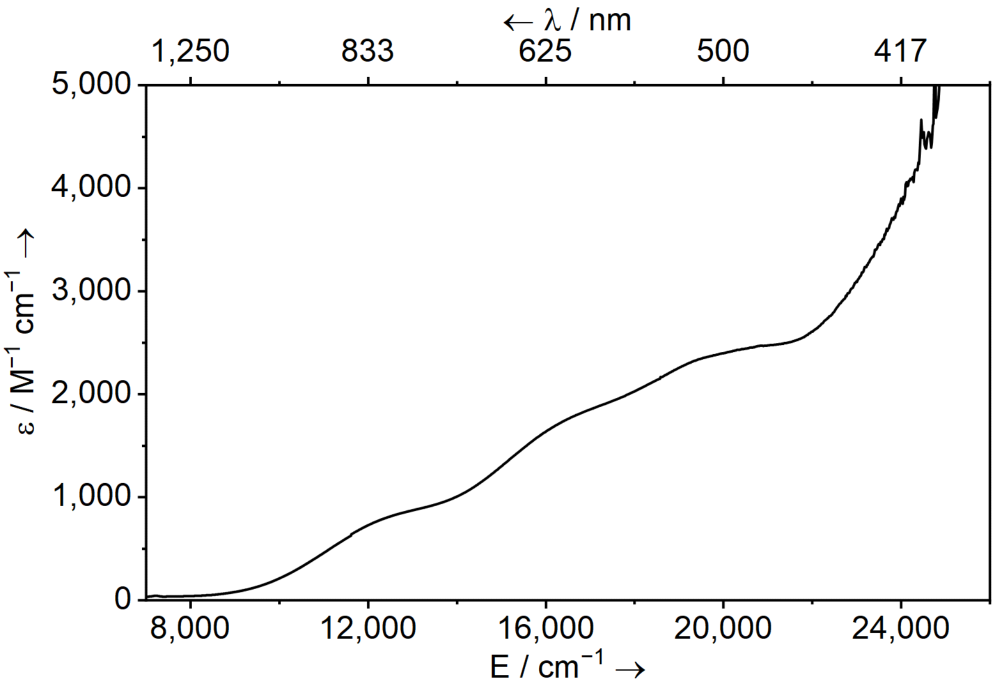

Figure S62. Solution UV-Vis-NIR spectrum of [Sc(NHAr^{iPr6})$_2$] (**2Sc**) (1 mM) in Et_2O shown between 7,000–26,000 cm^{-1} (1,429–385 nm) at ambient temperature.

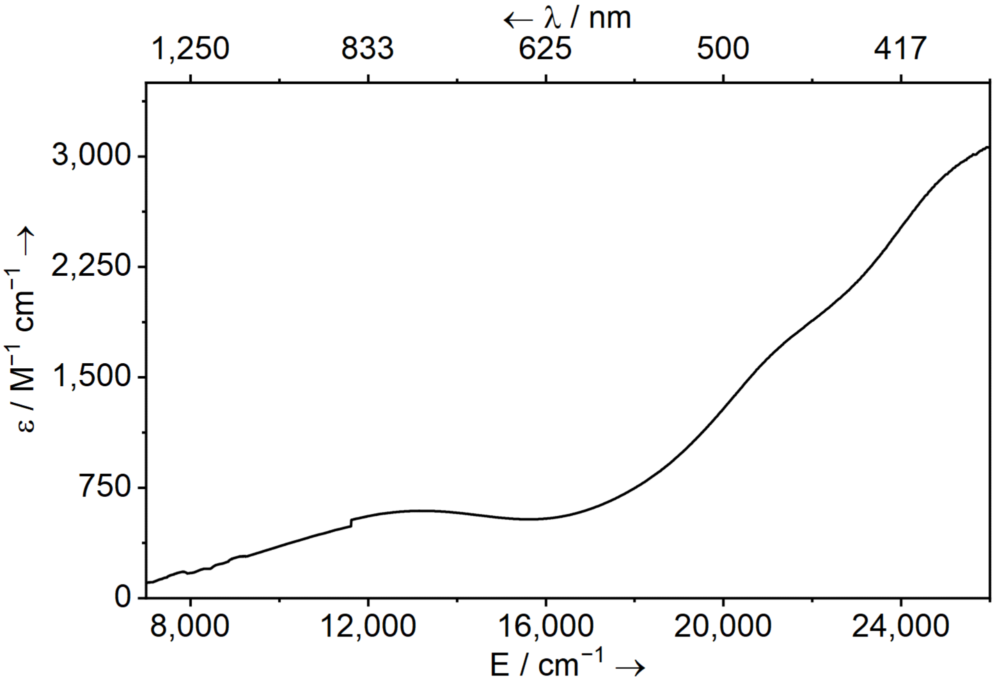

Figure S63. Solution UV-Vis-NIR spectrum of [Y(NHAr^{iPr6})$_2$] (**2Y**) (1 mM) in Et_2O shown between 7,000–26,000 cm^{-1} (1,429–385 nm) at ambient temperature.

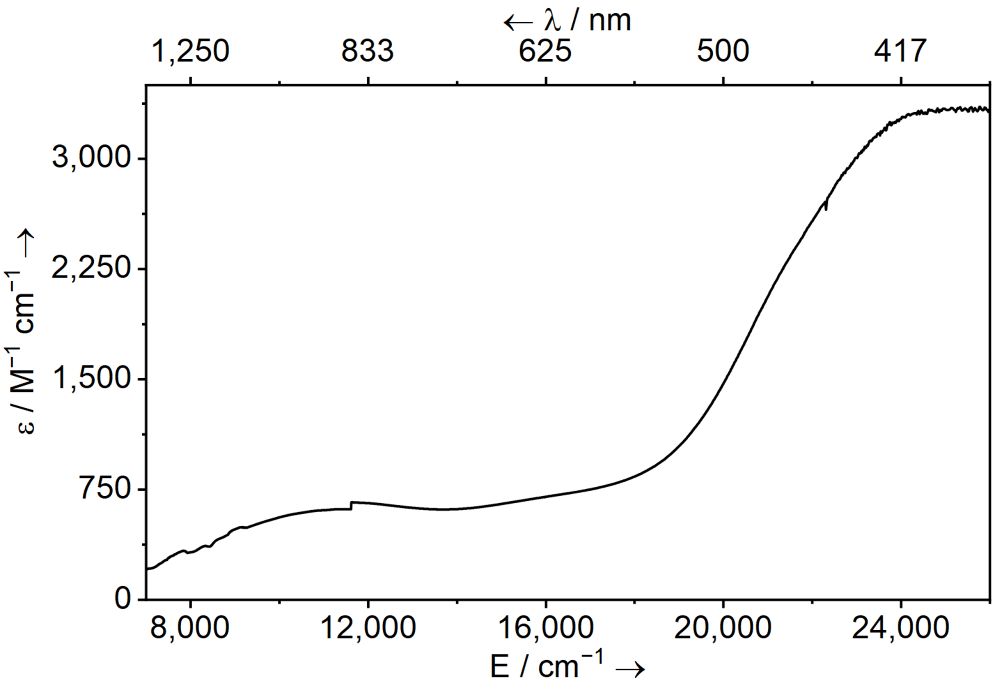

Figure S64. Solution UV-Vis-NIR spectrum of [La(NHAr^{iPr6})$_2$] (**2La**) (1 mM) in Et_2O shown between 7,000–26,000 cm^{-1} (1,429–385 nm) at ambient temperature.

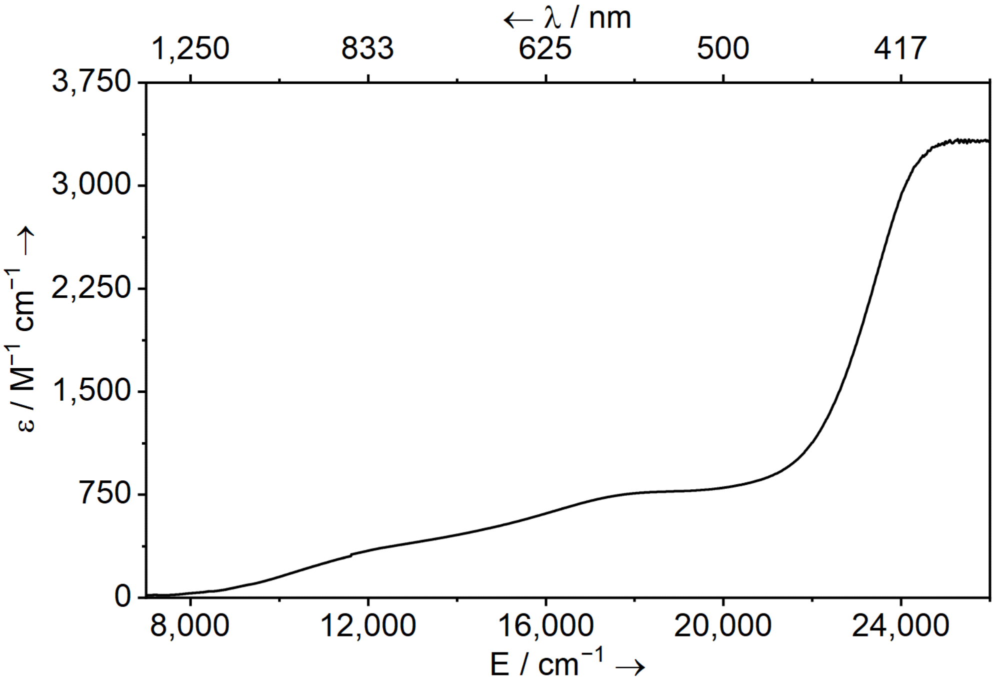

Figure S65. Solution UV-Vis-NIR spectrum of [Sm(NHAr^{iPr6})$_2$] (**2Sm**) (1 mM) in Et_2O shown between 7,000–26,000 cm^{-1} (1,429–385 nm) at ambient temperature.

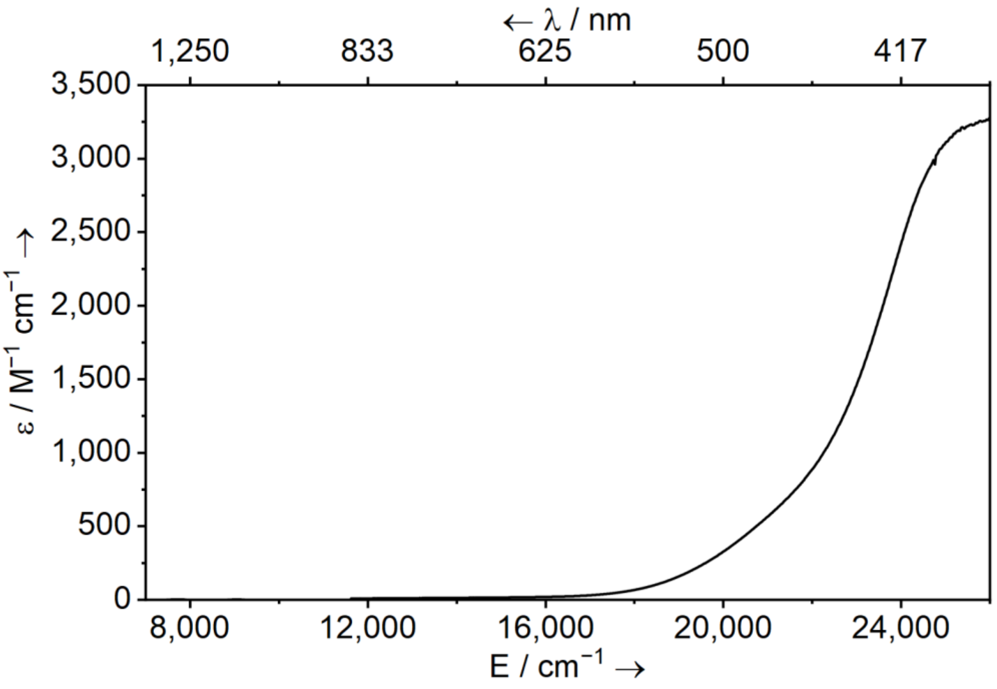

Figure S66. Solution UV-Vis-NIR spectrum of [Eu(NHAr^{iPr6})$_2$] (**2Eu**) (1 mM) in Et_2O shown between 7,000–26,000 cm^{-1} (1,429–385 nm) at ambient temperature.

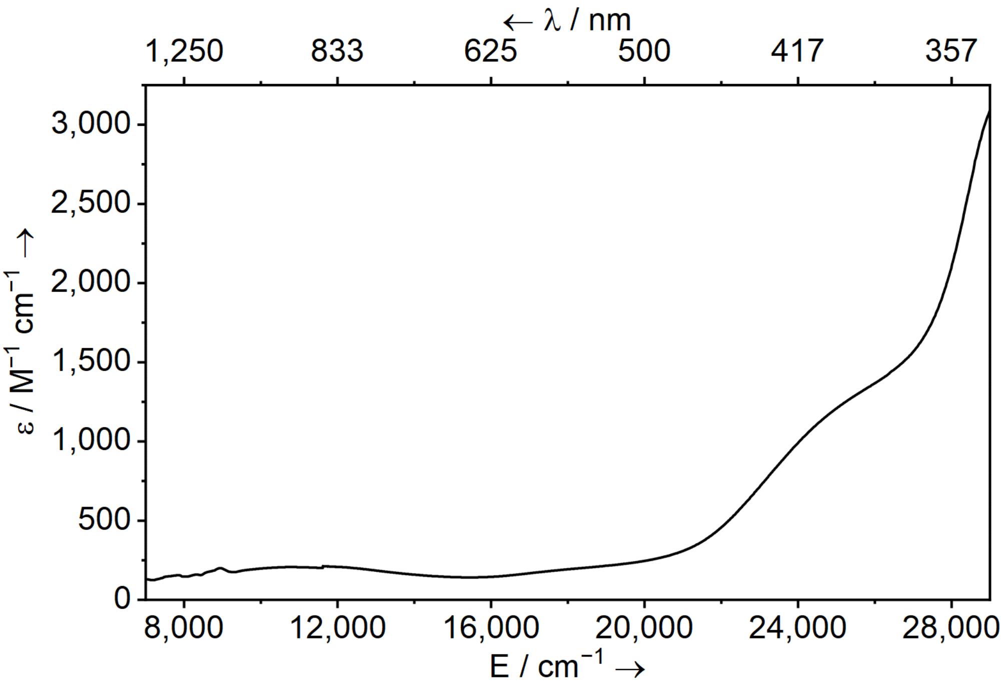

Figure S67. Solution UV-Vis-NIR spectrum of [Tm(NHAr^{iPr6})$_2$] (**2Tm**) (1 mM) in Et_2O shown between 7,000–26,000 cm^{-1} (1,429–385 nm) at ambient temperature.

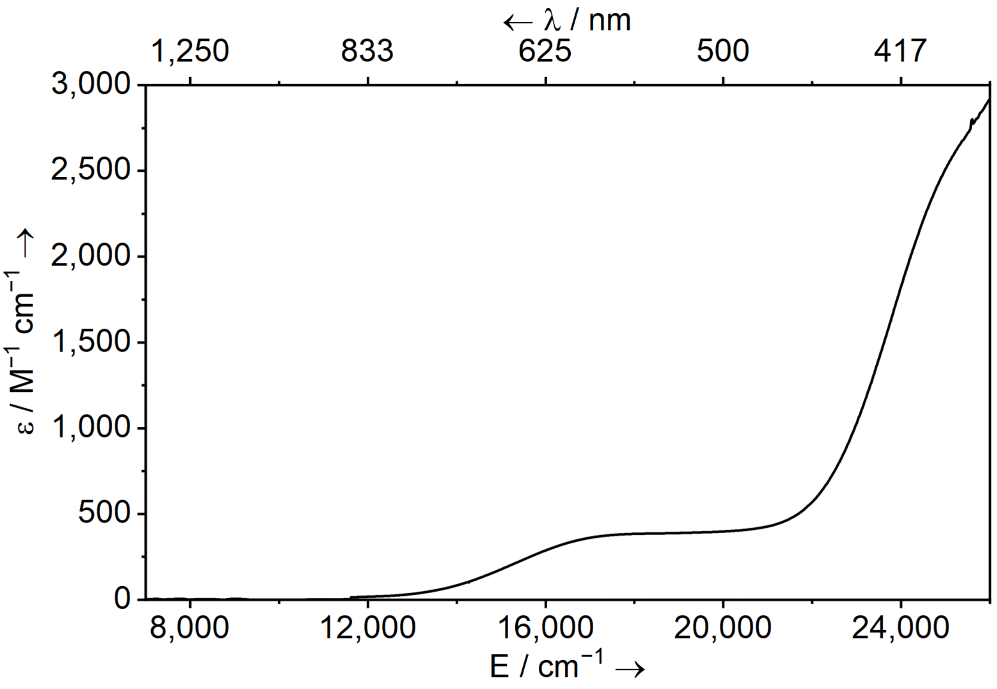

Figure S68. Solution UV-Vis-NIR spectrum of [Yb(NHAr^{iPr6})$_2$] (**2Yb**) (1 mM) in Et_2O shown between 7,000–26,000 cm^{-1} (1,429–385 nm) at ambient temperature.

*A discussion of the UV-Vis-NIR spectra of **1Tm**, **2Sm**, **2Eu**, **2Tm**, and **2Yb***

Complex **1Tm** shows a broad feature which tails from *ca*. 18,000 cm^{-1} (556 nm) into the UV region, and two distinctive sharp peaks at 12,723 cm^{-1} (786 nm, 44 M^{-1} cm^{-1}) and 14,556 cm^{-1} (687 nm, 51 M^{-1} cm^{-1}) for the $^3H_6 \rightarrow {}^3F_4$ and $^3H_6 \rightarrow {}^3F_{2/3}$ transitions, respectively. UV-Vis-NIR spectra of Sm(II), Tm(II), and Yb(II) often show moderately intense 4*f*→5*d* transitions, much like that of Ce(III), and indeed we see such features for **2Sm** at 12,337 cm^{-1} (811 nm, 364 M^{-1} cm^{-1}) and 18,784 cm^{-1} (532 nm, 774 M^{-1} cm^{-1}); for **2Tm** at 25,882 cm^{-1} (386 nm, 1,305 M^{-1} cm^{-1}) and 11,224 cm^{-1} (891 nm, 207 M^{-1} cm^{-1}); and for **2Yb** at 18,643 cm^{-1} (536 nm, 387 M^{-1} cm^{-1}). Complex **2Eu** displays no features within the UV-Vis-NIR spectral range except a broad LMCT which tails from 16,000 cm^{-1} (625 nm) into the UV region, as is typical for $^8S_{7/2}$ ions.[15]

*Comparison of UV-Vis-NIR spectra between **1M** and **2M** series.*

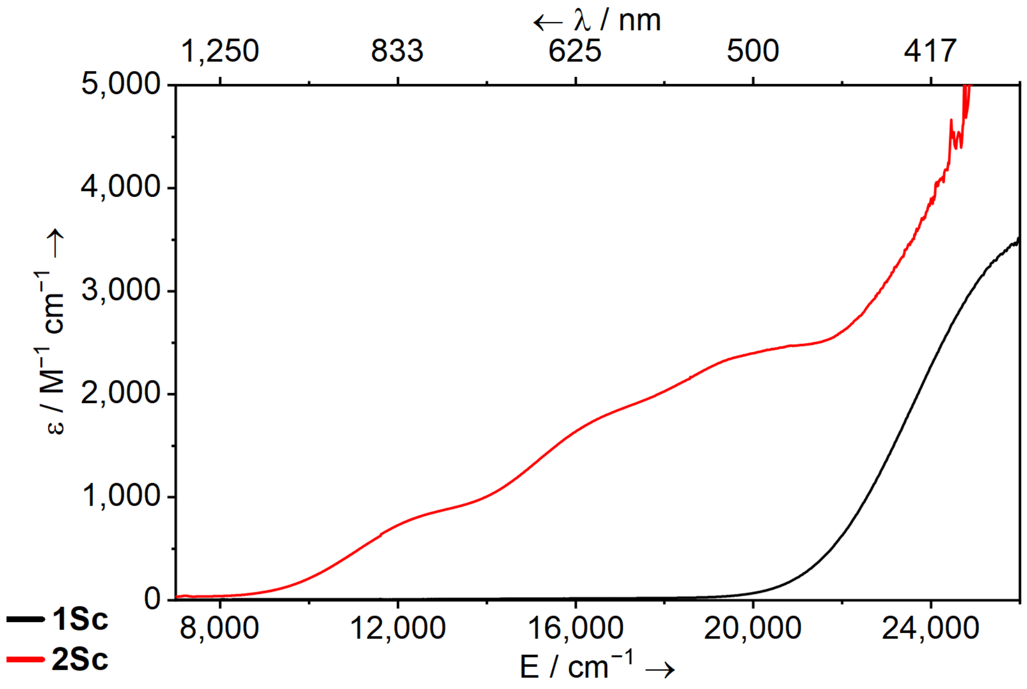

Figure S69. Solution UV-Vis-NIR spectra of [Sc(NHAr^{iPr6})$_2$(I)] (**1Sc**) (1 mM, Et_2O, black line) and [Sc(NHAr^{iPr6})$_2$] (**2Sc**) (1 mM, Et_2O, red line) shown between 7,000–26,000 cm^{-1} (1,429–385 nm) at ambient temperature.

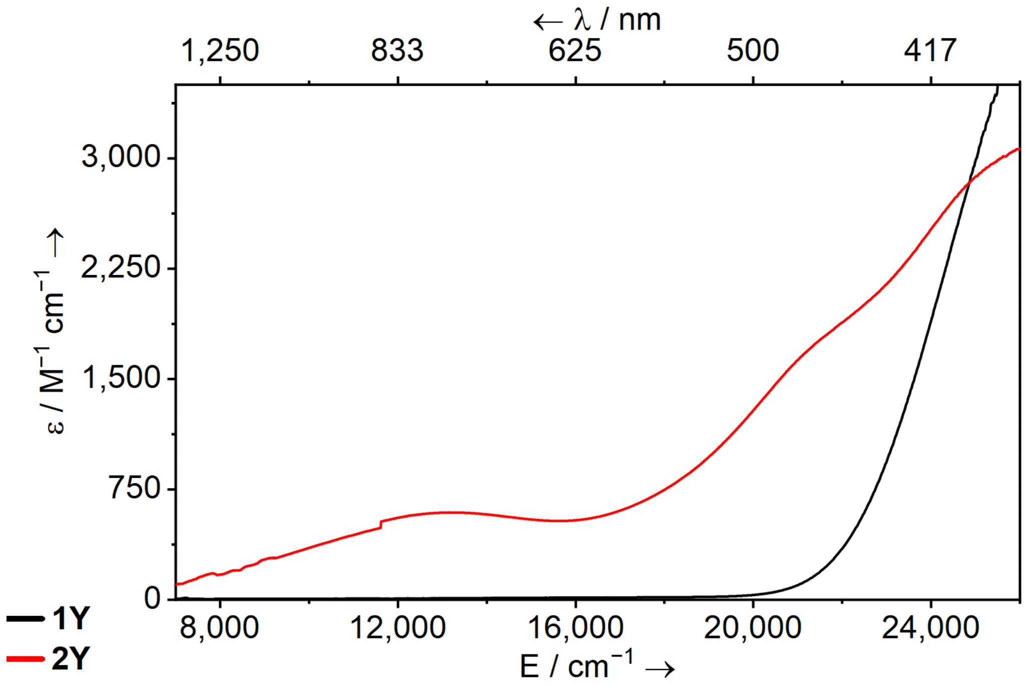

Figure S70. Solution UV-Vis-NIR spectra of [Sc(NHAr^{iPr6})$_2$(I)] (**1Y**) (1 mM, Et_2O, black line) and [Y(NHAr^{iPr6})$_2$] (**2Y**) (1 mM, Et_2O, red line) shown between 7,000–26,000 cm^{-1} (1,429–385 nm) at ambient temperature.

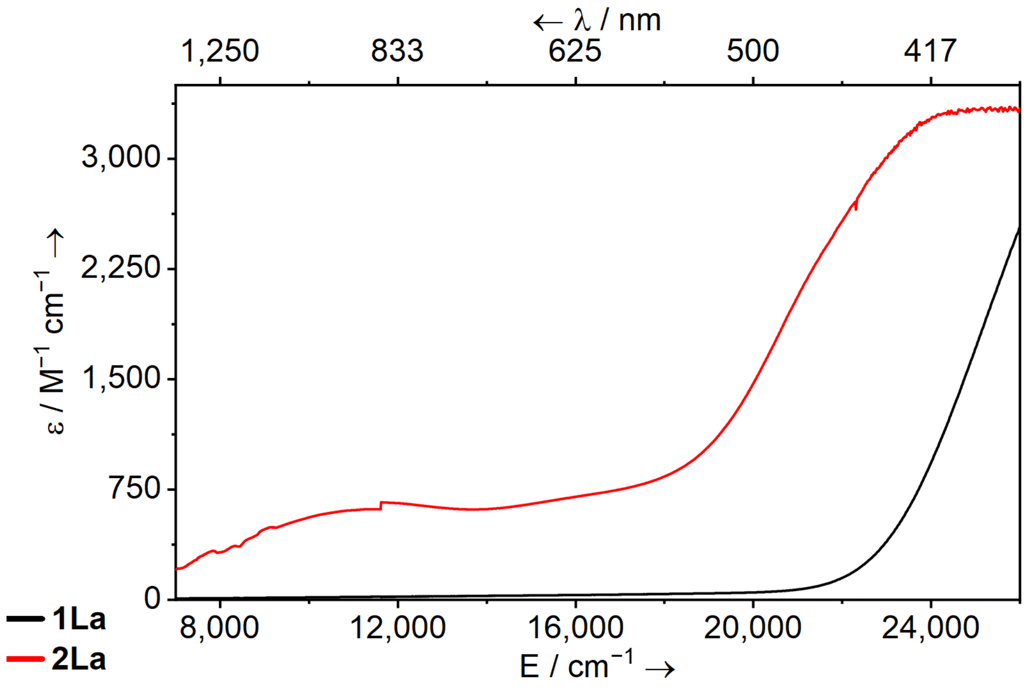

Figure S71. Solution UV-Vis-NIR spectra of [La(NHAr^{iPr6})$_2$(I)] (**1La**) (1 mM, Et_2O, black line) and [La(NHAr^{iPr6})$_2$] (**2La**) (1 mM, Et_2O, red line) shown between 7,000–26,000 cm^{-1} (1,429–385 nm) at ambient temperature.

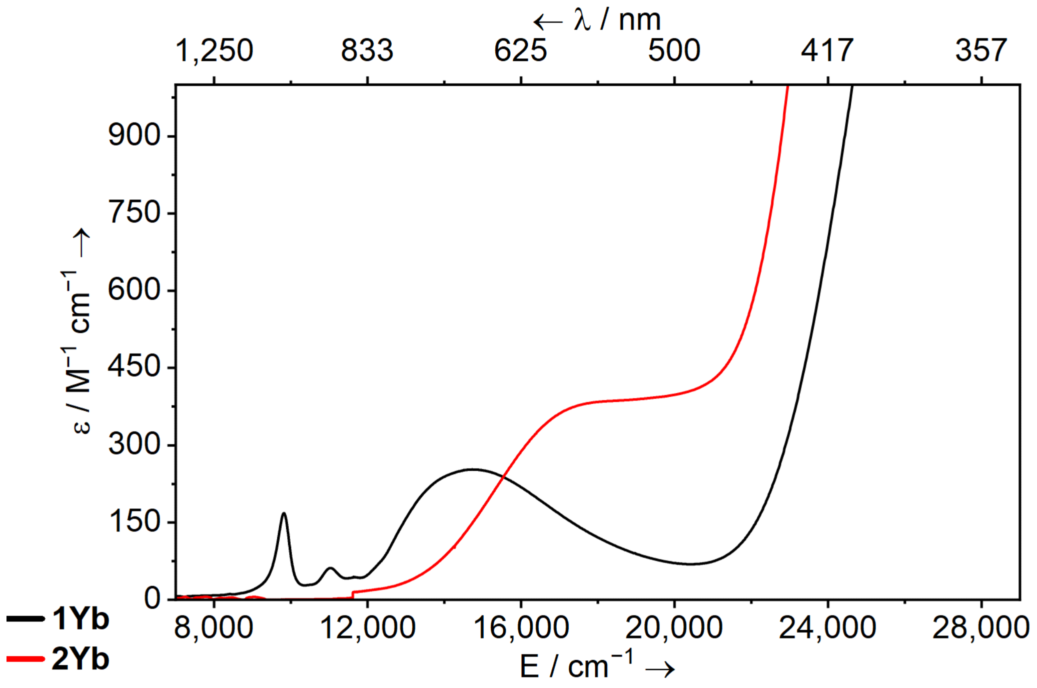

Figure S72. Solution UV-Vis-NIR spectra of [Yb(NHAr^{iPr6})$_2$(I)] (**1Yb**) (1 mM, Et_2O, black line) and [Yb(NHAr^{iPr6})$_2$] (**2Yb**) (1 mM, Et_2O, red line) shown between 7,000–26,000 cm^{-1} (1,429–385 nm) at ambient temperature.

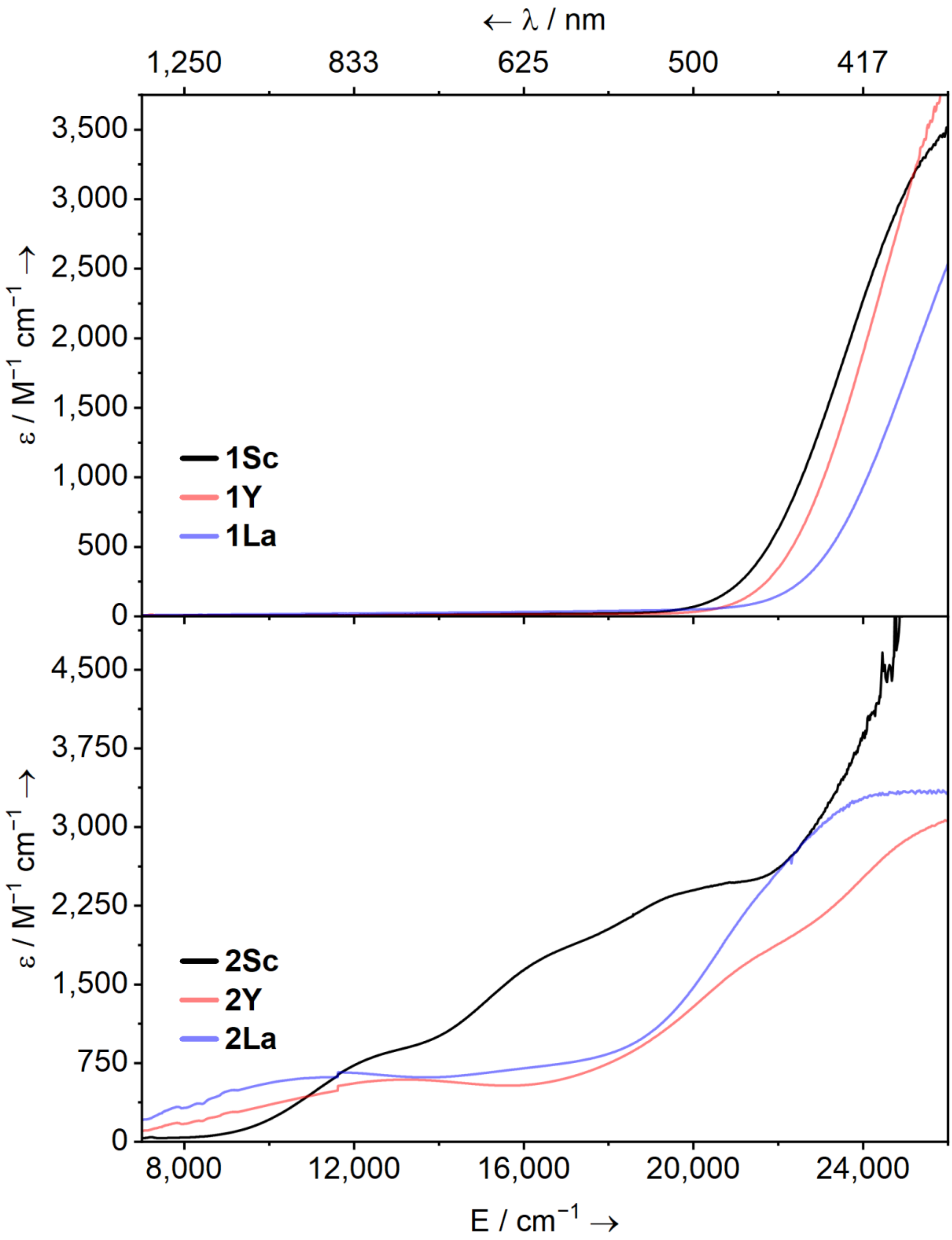

Figure S73. Solution UV-Vis-NIR spectra of **1M** (top): [Sc(NHAr^{iPr6})$_2$(I)] (**1Sc**, black line), [Y(NHAr^{iPr6})$_2$(I)] (**1Y**, red line), and [La(NHAr^{iPr6})$_2$(I)] (**1La**, blue line); and **2M** (bottom): [Sc(NHAr^{iPr6})$_2$] (**2Sc**, black line), [Y(NHAr^{iPr6})$_2$] (**2Y**, red line), and [La(NHAr^{iPr6})$_2$] (**2La**, blue line). All were collected at ambient temperature as 1 mM solutions in Et_2O, and are shown between 7,000–26,000 cm^{-1} (1,429–385 nm) at ambient temperature.

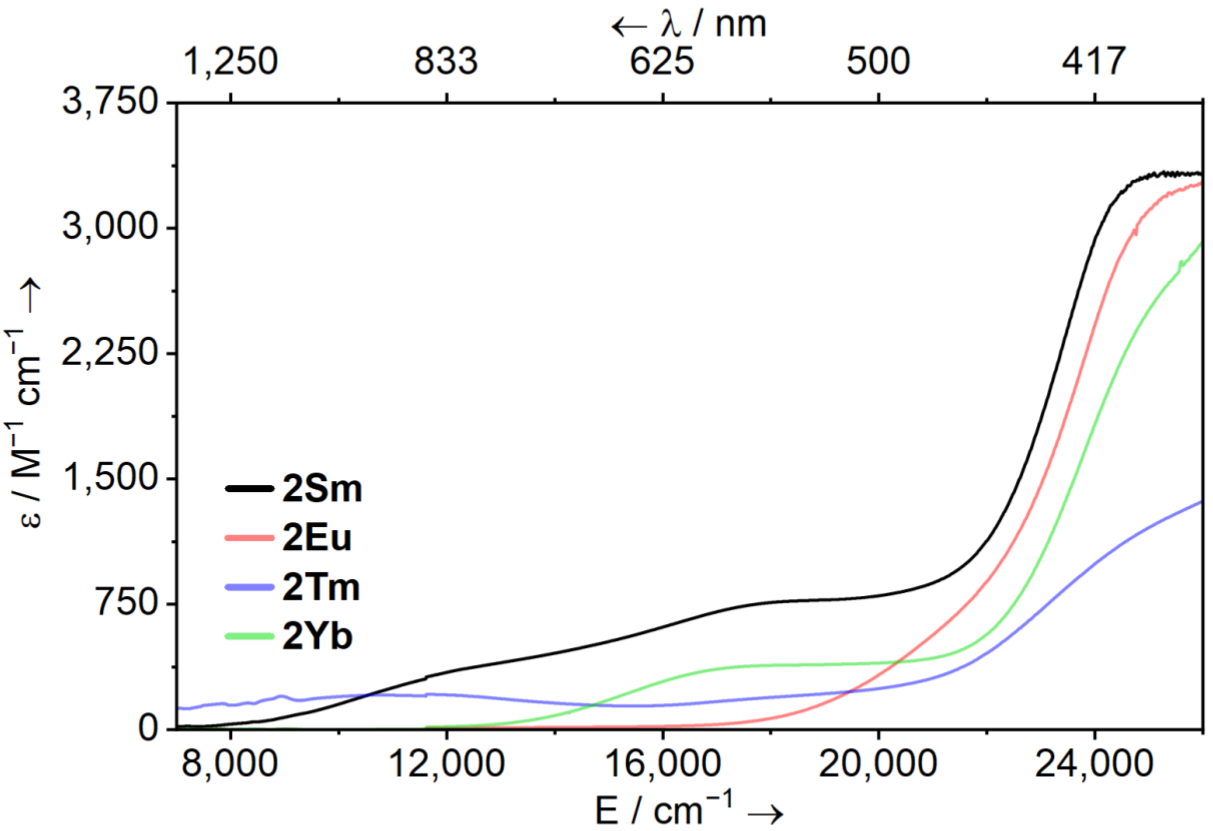

Figure S74. Solution UV-Vis-NIR spectra of [Sm(NHAr$^{i\text{Pr}6}$)$_2$] (**2Sm**, black line), [Eu(NHAr$^{i\text{Pr}6}$)$_2$] (**2Eu**, red line), [Tm(NHAr$^{i\text{Pr}6}$)$_2$] (**2Tm**, blue line), and [Yb(NHAr$^{i\text{Pr}6}$)$_2$] (**2Yb**, green line). All were collected at ambient temperature as 1 mM solutions in Et_2O, and are shown between 7,000–26,000 cm^{-1} (1,429–385 nm) at ambient temperature.

S7. EPR Spectroscopy

EPR spectroscopy data and discussion

While we find better resolved frozen solution c.w. EPR spectra in *n*Pr_2O than in Et_2O for **2Sc**, **2Y** and **2La**, in the case of **2Sc** and **2La** we were able to obtain reliable fluid solution spectra to higher temperatures in Et_2O. This is because the samples decomposed on thawing more rapidly in *n*Pr_2O, than in Et_2O, though this was not always consistent across samples and likely reflects difficulties in sample preparation, rather than intrinsic instabilities as all three complexes were found to be stable at room temperature in both solvents for multiple days inside an inert atmosphere glovebox. Nevertheless, this factor is important because the greater spectral spread and hyperfine anisotropy for **2Sc** and **2La** than for **2Y** means that higher temperatures are required for truly isotropic spectra to be obtained (*i.e.* for tumbling rates in solution being sufficient to average out anisotropy).

X-band spectra of powders are partially metal hyperfine-resolved for **2Sc** (^{45}Sc, I = 7/2, 100% abundant) and **2Y** (^{89}Y, I = 1/2, 100%) but unresolved for **2La** (^{139}La, I = 7/2, 100%), at room temperature and on cooling to 5 K. For **2Sc**, a hyperfine octet is observed consistent with the formal Sc(II) oxidation state. The spectra appear isotropic (even at K-band) with g = 2.000 and A = 145 MHz at 300 K, narrowing to 120 MHz at 5 K. For **2Y** we find axial spectra which narrow on cooling, with best estimates from simulation giving $g_{\perp}$ = 2.005, $g_{\parallel}$ = 1.995 and $g_{\perp}$ = 2.003, $g_{\parallel}$ = 1.996 at 300 and 5 K, respectively. We estimate a hyperfine interaction of A = 14 MHz from the distorted lineshapes although there is considerable uncertainty in this value. For **2La**, we observe a signal with $g_{\perp}$ = 2.019 and $g_{\parallel}$ = 1.958 (from K-band) with no hyperfine resolution. The limited resolution of the powder spectra of **2Sc**, **2Y** and **2La** are indicative of intermolecular interactions but also make it difficult to discuss changes in electronic structure between solid and solution phases.

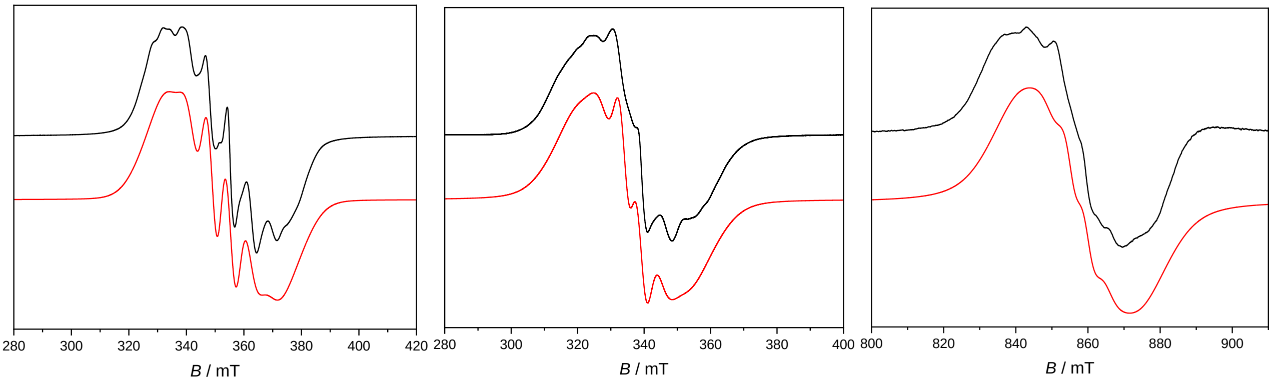

Figure S75. X-band polycrystalline **2Sc** at r.t. (left) and 5 K (middle), and K-band at 5 K (right). Black experimental, red simulation (g = 1.9995 and A = 120 MHz with significant A-strain, Sys.AStrain = 120 MHz for X-band and Sys.AStrain = 110 MHz for K-band, used in simulations).

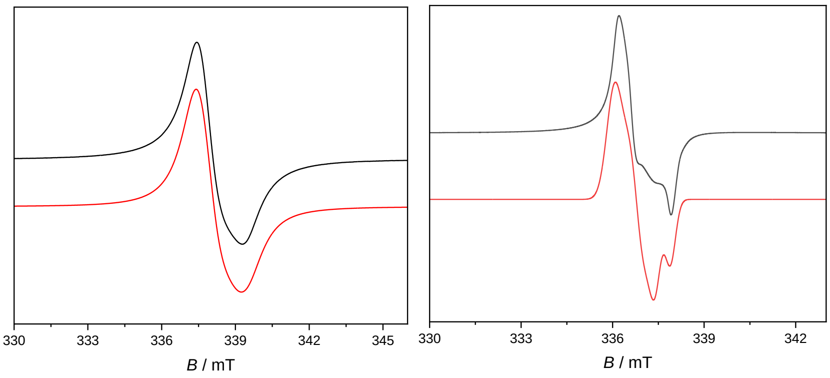

Figure S76. X-band c.w. EPR spectrum of polycrystalline **2Y** recorded at r.t. (left) and 5 K (right). Black experimental, red simulation ($g_{\perp}$ = 2.0045, $g_{\parallel}$ = 1.9945 at RT; $g_{\perp}$ = 2.003, $g_{\parallel}$ = 1.9958 at 5 K; rough estimate of an isotropic ^{89}Y hyperfine of $A \approx$ 14 MHz from the distorted lineshapes).

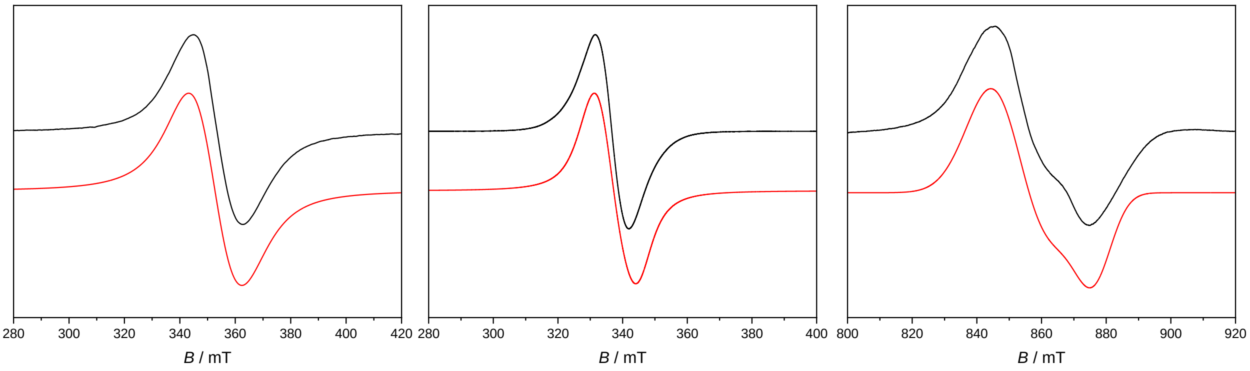

Figure S77. X-band polycrystalline **2La** at r.t. (left) and 5 K (middle), and K-band at 5 K (right). Black experimental, red simulation ($g_{\perp}$ = 2.019 and $g_{\parallel}$ = 1.958; obtained from K-band data).

We observed much improved resolution in frozen solution spectra in nPr$_2$O than in Et$_2$O for all three complexes, although we could more reliably measure fluid solution spectra to higher temperature in Et$_2$O.

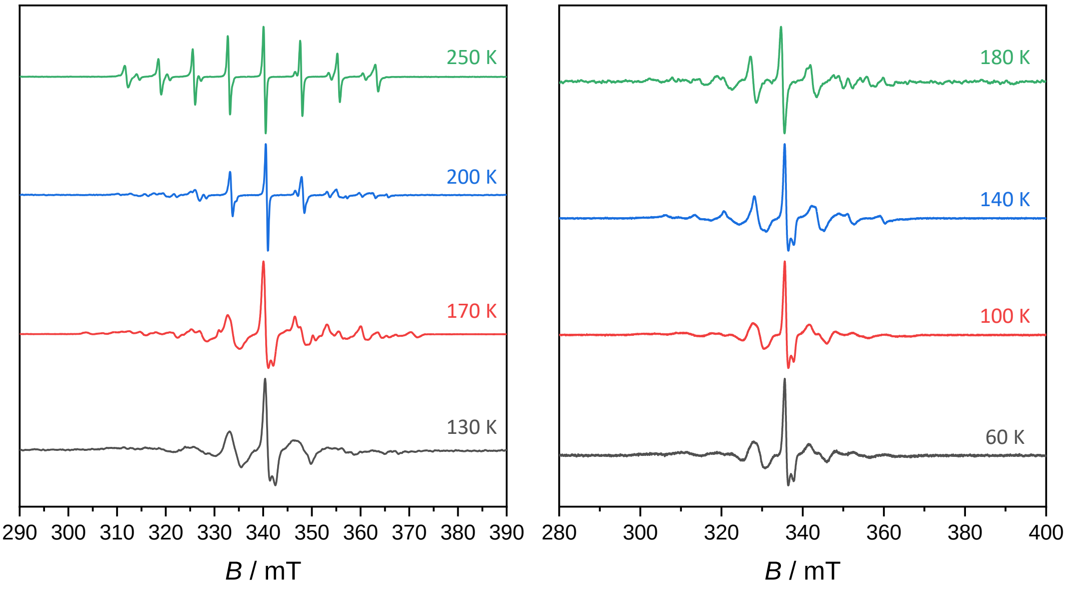

Figure S78. X-band c.w. EPR spectrum of 1 mM **2Sc** in Et$_2$O recorded at temperature range 130–250 K (left) and in nPr$_2$O recorded at temperature range 60–180 K (right). The sample degraded above 180 K in nPr$_2$O.

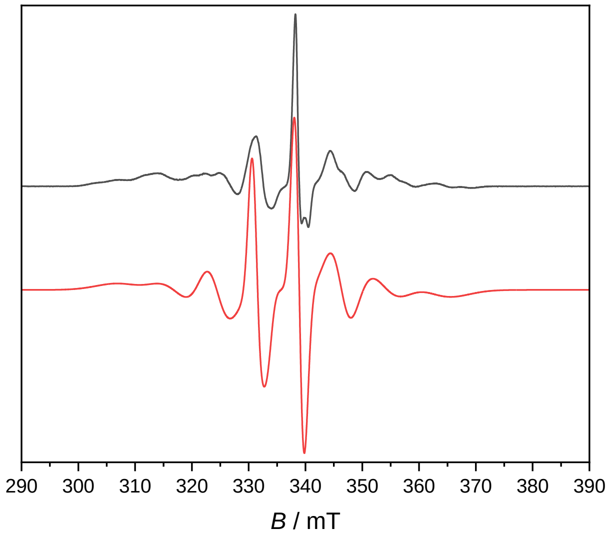

Figure S79. X-band c.w. EPR spectrum of 1mM **2Sc** in *n*Pr_2O recorded at 60 K. Black experimental, red simulation. The simulation parameters are $g_{\perp}$ = 2.002 and $g_{\parallel}$ = 1.990, $A_{\perp}$ = 210 MHz and $A_{\parallel}$ = 180 MHz.

The variable temperature measurement of **2Sc** in Et_2O across the temperature range of 130–250 K shows an 8-line signal that is being broadened and strained in the frozen solution. The sample thaws and motional effects are observed above ca. 170 K, but with incomplete averaging. An isotropic spectrum is observed above ca. 200 K. At the highest temperature of 250 K it is evident that two different species are present with subtly different hyperfine values.Upon recooling of the system back to 130 K an indistinguishable spectrum is obtained compared to the original spectrum recorded at 130 K.

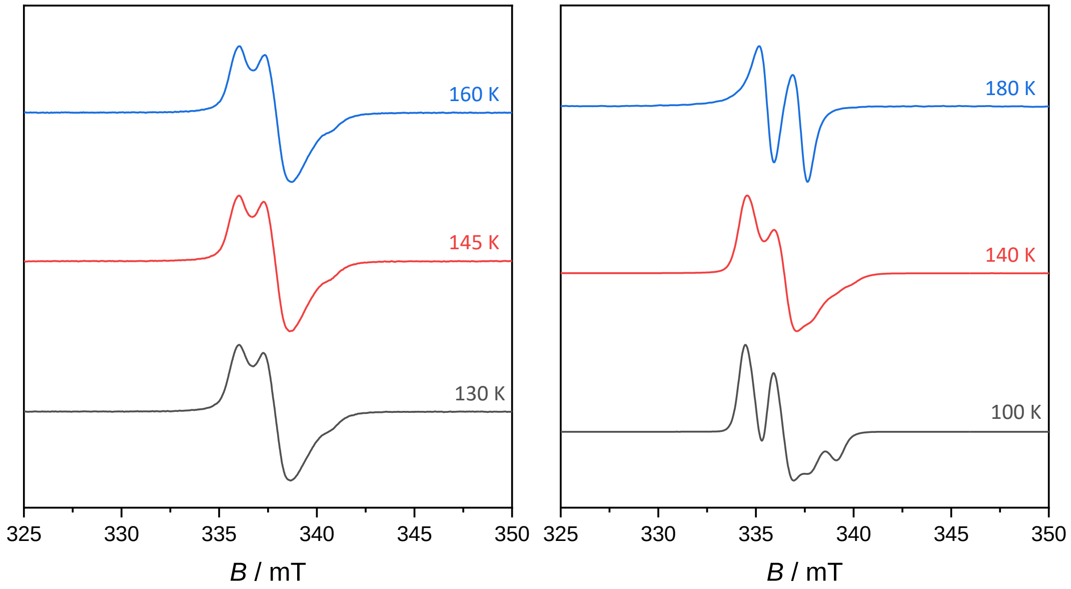

Figure S80. X-band c.w. EPR spectrum of 1 mM **2Y** in Et_2O recorded at temperature range 130–160 K (left) and in $n$$Pr_2O$ recorded at temperature range 100–180 K (right). The sample degraded above 165 K in Et_2O and above 180 K in $n$$Pr_2O$.

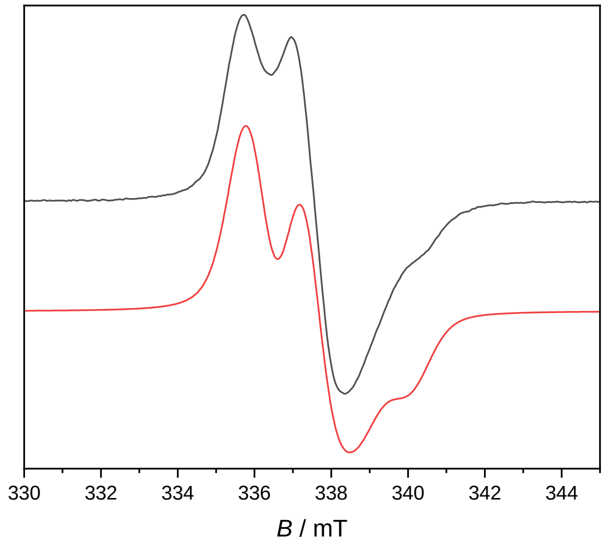

Figure S81. X-band c.w. EPR spectrum of 1 mM **2Y** in Et_2O recorded at 130 K. Black experimental, red simulation. The simulation parameters are $g_{\perp}$ = 2.008 and $g_{\parallel}$ = 1.992, $A_{\perp}$ = 39 MHz and $A_{\parallel}$ = 36 MHz.

The spectrum of **2Y** in Et_2O has been reported and we find comparable results.[16] The variable temperature measurement of **2Y** in Et_2O across the temperature range of 130–165 K shows an axial signal with an observable hyperfine splitting. The spectral features are more resolved in $n$$Pr_2$-O, at

180 K the spectrum appears to be in the isotropic limit; the sample did not survive heating to higher temperatures. The spectra in both solvent systems are consistent across the temperature range, exhibiting only slight changes upon heating. The *g*-values are slightly shifted downfield compared to the values recorded in *n*Pr_2O, reported in the main text. Both values have been corrected against a strong pitch. The hyperfine value is consistent throughout the two different solvents.

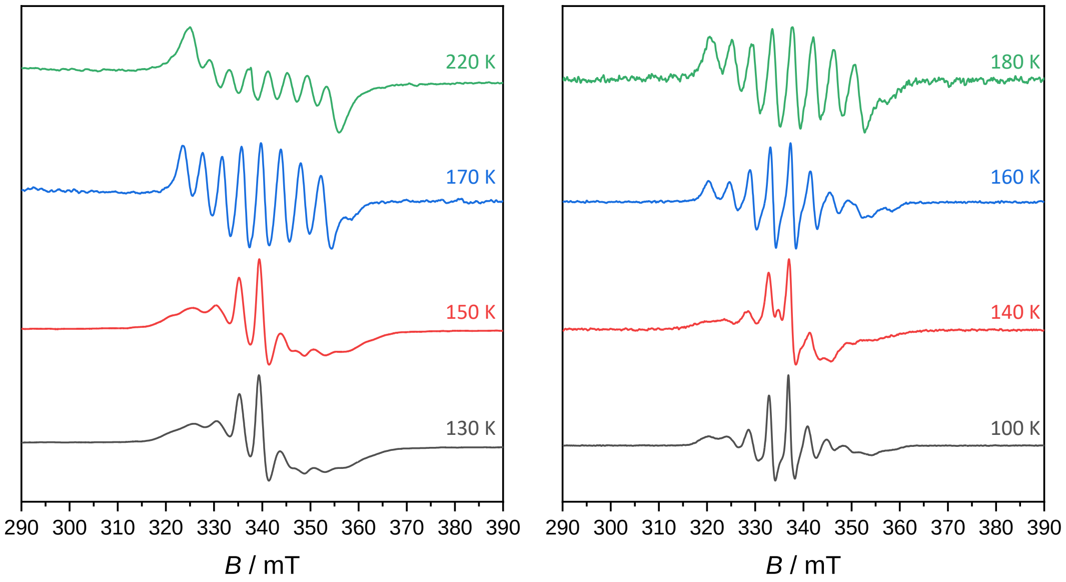

Figure S82. X-band c.w. EPR spectrum of 1 mM **2La** in Et_2O recorded at temperature range 130–220 K (left) and in *n*Pr_2O recorded at temperature range 100–180 K (right). The sample degraded above 180 K in *n*Pr_2O.

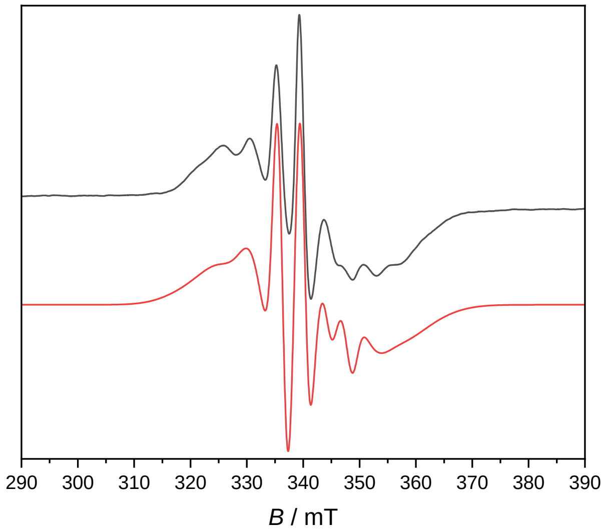

Figure S83. X-band c.w. EPR spectrum of 1 mM 2Y in Et_2O recorded at 130 K. Black experimental, red simulation. The simulation parameters are $g_{\perp}$ = 2.002 and $g_{\parallel}$ = 1.950, $A_{\perp}$ = 110 MHz and $A_{\parallel}$ = 100 MHz.

The variable temperature measurement of **2La** in Et_2O across the temperature range of 130–220 K shows an axial signal with an observable 8-line hyperfine splitting. As for **2Sc**, the sample starts to thaw and the spectrum shows motional effects above ca. 170 K, with distinctive 8-line isotropic spectra being observed above ca. 200 K. The simulation of the fluid solution data in Et_2O gives g_{iso} = 1.989 and A_{iso} = 112 MHz, consistent with the frozen solution data.

S8. SQUID Magnetometry

For the elements with f^n configurations, like Ln(III) ions, contributions resulting from interelectronic repulsion (spin-spin coupling) are greatest, spin-orbit coupling is large due to the population of the 4*f* orbitals, but crystal field effects are relatively small as the 4*f* orbitals are core-like and therefore interactions with ligand orbitals are weak and predominately electrostatic in nature (resultant ligand field effects are therefore small), therefore, in general, magnetic properties can be determined almost entirely by an *assumed* well-isolated ground state, and are generally well-described by the *L*-*S* coupling scheme.[17-19] This is important as it means that mostly the ground *J* state is populated, and the magnetic properties are somewhat or almost completely independent of environment.

In contrast, lanthanides with $4f^n5d^1$ configurations, such as which may be anticipated for some molecular complexes in the Ln(II) oxidation state, necessarily deviates from the values calculated using the *L*-*S* coupling scheme.[20-22] As such, magnetic properties may be better described by a *J*-*s* coupling scheme.[20, 21, 23] However, in this coupling scheme $\chi_M T$ (cm^3 mol^{-1} K) will depend on the relative magnitude of 4*f*-5*d* coupling with respect to $k_B T$. Recent work has shown exact alignment with any coupling scheme for $4f^n5d^1$ ions is over-reductive, as the impact of CF splitting on the ground state, particularly important where the occupation of *d* orbitals in concerned, needs to be considered.[24] Below, we present a brief account of the various coupling schemes which the **2M** (M = Sc, Y, La, Sm, Eu, Tm, and Yb) complexes may operate in to show how SQUID magnetometry may (or may not) provide clarity to their ground state electron configurations.

Coupling schemes

$4f^{n+1}$ L-S coupling scheme: Only 4*f* orbitals are populated, so we have a well-isolated ground state, and the magnetic moment of a *J* state is calculated using the Landé formula:

$$\mu_{eff} = g \times \sqrt{J(J+1)}$$

$$g = \frac{3}{2} \times \{S(S+1) - \frac{L(L+1)}{2J(J+1)}\}$$

Table S12. Effective magnetic moment (μ_B) and $\chi_M T$ (cm^3 mol^{-1} K) values calculated using the $4f^{n+1}$ *L*-*S* coupling scheme for divalent Sm, Eu, Tm, and Yb.

	μ_{eff}	$\chi_M T$
**Sm(II)**	0	0
**Eu(II)**	7.94	7.88
**Tm(II)**	4.54	2.57
**Yb(II)**	0	0

$4f^n 5d^1$ L-S coupling scheme: the *d*-orbitals are now populated, however 4*f*-5*d* spin-spin coupling is greater than 4*f* spin-orbit coupling, and we still have a well-isolated ground state. The magnetic moment of the systems is therefore calculated as follows (*g* is defined above):

$$S_{total} = S_{4f} + S_{5d}$$

$$L_{total} = L_{4f}$$

$$J_{total} = S_{total} + L_{total}$$

$$\mu_{eff} = g \times \sqrt{J_{total}(J_{total}+1)}$$

Table S13. Effective magnetic moment (μ_B) and $\chi_M T$ ($cm^3\ mol^{-1}\ K$) values calculated using the $4f^n5d^1$ *L-S* coupling scheme for divalent Sc, Y, La, Sm, Eu, Tm, and Yb.

	μ_{eff}	$\chi_M T$
**Sc(II)**	1.73	0.38
**Y(II)**	1.73	0.38
**La(II)**	1.73	0.38
**Sm(II)**	0	0
**Eu(II)**	3.46	1.50
**Tm(II)**	8.59	9.23
**Yb(II)**	5.59	3.91

$4f^n5d^1$ J-s coupling scheme: There are two possible scenarios. Firstly, 4*f* spin-orbit coupling is now much greater than 4*f*-5*d* spin-spin coupling. However, the 4*f*-5*d* spin-spin coupling is still greater than k_BT, therefore the ground state is still well-isolated and the magnetic moment values match those calculated using the $4f^n5d^1$ *L-S* coupling scheme.

Secondly, where the 4*f* spin-orbit coupling and k_BT is now much greater than 4*f*-5*d* spin-spin coupling. There is thermal population of both ground and the first excited state at 300 K and the magnetic moment is calculated *via* the incorporation of an $S = {}^1/_2$ unpaired spin into the Landé formula (where J_{4f} is the total angular momentum of the 4*f* electrons):

$$g = \sqrt{3 + \{(g_{4f}{}^2) \times J_{4f}(J_{4f} + 1)\}}$$

Table S14. Effective magnetic moment (μ_B) and $\chi_M T$ ($cm^3\ mol^{-1}\ K$) values calculated using the $4f^n5d^1$ *J-s* coupling scheme for divalent Sc, Y, La, Sm, Eu, Tm, and Yb.

	μ_{eff}	$\chi_M T$
**Sc(II)**	1.73	0.38
**Y(II)**	1.73	0.38
**La(II)**	1.73	0.38
**Sm(II)**	1.93	0.46
**Eu(II)**	1.73	0.38
**Tm(II)**	7.76	7.52
**Yb(II)**	4.86	2.95

In general, we would anticipate that Ln(II) systems with f^{n+1} configurations (*i.e.* Sm(II), Eu(II), Tm(II), and Yb(II) complexes) to exhibit magnetic properties closely aligned with those calculated by the *L-S* coupling scheme (**Table S12**). Conversely, those with f^nd^1 configurations (*i.e.* Sc(II), Y(II), and La(II) where $n = 0$), could deviate from those calculated *via* the *J-s* coupling scheme, or from the spin-only value, due to the ground state now **not** being well-isolated and the increased electronic effect of CF splitting on the ground state term.[7]

*Magnetometry data and discussion for **1Tm**, **2Sm**, **2Eu**, and **2Tm***

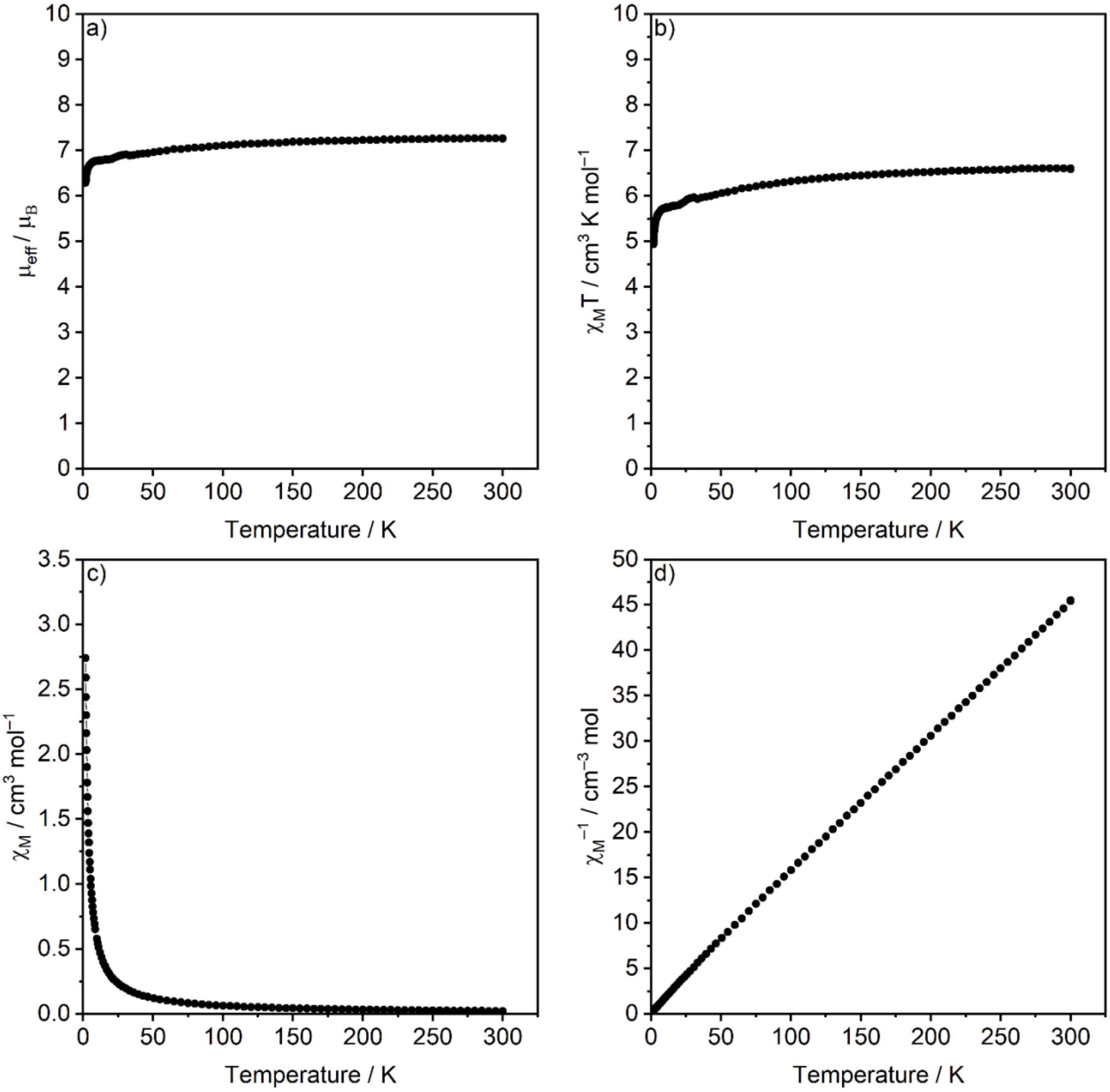

Figure S84. Variable-temperature SQUID magnetometry of **1Tm** over the temperature range 1.8-300 K: a) μ_{eff} vs T; b) χT vs T; c) χ vs T; d) χ^{-11} vs T.

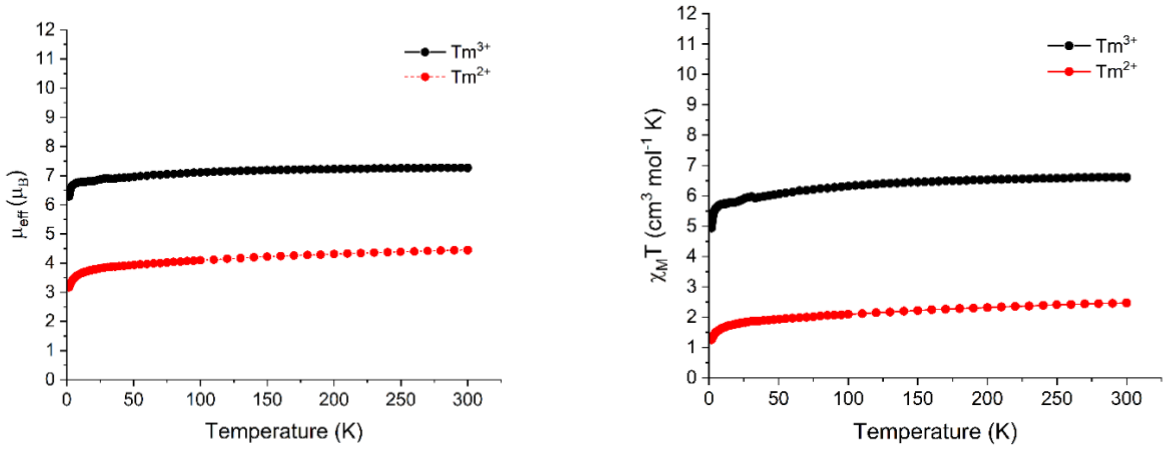

Figure S85. ***Left:*** Temperature-dependent SQUID effective magnetic moment (μ_B) vs Temperature (K) magnetometry data for **1Tm** (black circles) measured over the temperature range of 1.8 to 300 K. The lines are a guide to the eye only, and the red circles represent experimental data for **2Tm**. ***Right:*** Temperature-dependent SQUID $\chi_M T$ (cm^3 mol^{-1} K) vs Temperature (K) magnetometry data for **1Tm** (black circles) measured over the temperature range of 1.8 to 300 K. The lines are a guide to the eye only, and the red circles represent experimental data for **2Tm**.

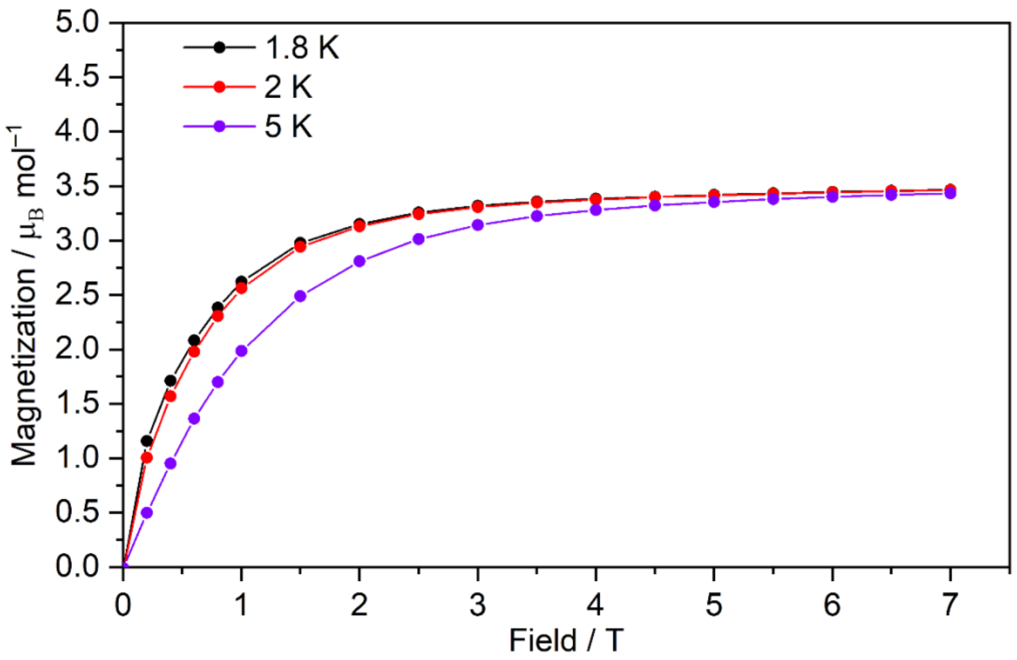

Figure S86. Magnetization vs Field data for **1Tm** at 1.8, 2, and 5 K. The lines are a guide to the eye only.

Trivalent thulium, Tm(III), as in **1Tm**, should possess an electronic configuration of $4f^{12}$ and follow the $4f^{n+1}$ L-S coupling scheme. As such, DC magnetic susceptibility measurements of a powdered sample of **1Tm** exhibit a magnetic moment at 300 K which is in good agreement with the theoretical calculated value using the $4f^{n+1}$ L-S coupling scheme. The magnetic moment at 300 K is 7.27 μ_B (6.61 cm^3 mol^{-1} K), in reasonable agreement with the expected value of 7.56 μ_B (7.15 cm^3 mol^{-1} K) based on a $4f^{12}$ $^3H_6$ Tm(III) ion. This value decreases gradually across the temperature range due to progressive thermal depopulation of crystal field states settling on a value of 6.29 μ_B (4.94 cm^3 mol^{-1} K) at 1.8 K (**Figure S85**). Many Tm(III) complexes possess a singlet non-magnetic ground state for the $4f^{12}$ non-Kramers ion with the gradual reduction in magnetic moment across the temperature range tending towards zero.[25] Additionally, the non-Kramers nature of a singlet non-magnetic ground state should preclude saturation of magnetization up to the highest field. However, in this system, the magnetization versus field data saturates above 4 T at 1.8, 2 and 4 K up to ~3.40 μ_B mol^{-1} (**Figure S86**). This suggests that in this system we have a pseudo-doublet, not singlet, ground state.[25, 26]

Comparison to the experimental data of **1Tm** to **2Tm** (*vide infra*) highlights significant differences in effective magnetic moment (μ_B) and $\chi_M T$ values at both 300 K (**1Tm**: 7.27 μ_B, 6.61 cm^3 mol^{-1} K; **2Tm**: 4.45 μ_B, 2.47 cm^3 mol^{-1} K) and 1.8 K (**1Tm**: 6.29 μ_B, 4.94 cm^3 mol^{-1} K; **2Tm**: 3.17 μ_B, 1.26 cm^3 mol^{-1} K) consistent with the change in oxidation state.

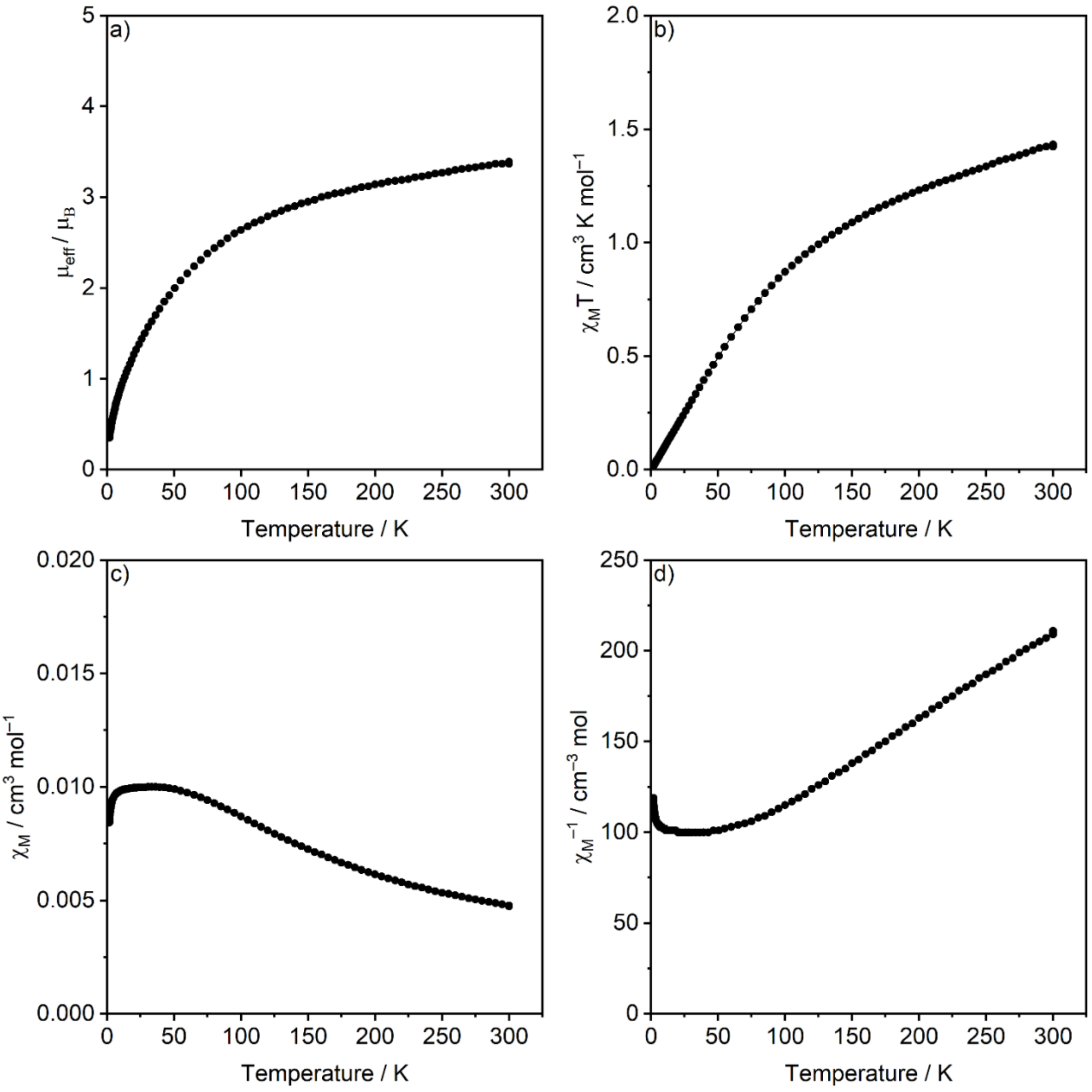

Figure S87. Variable-temperature SQUID magnetometry of **2Sm** over the temperature range 1.8-300 K: a) μ_{eff} vs T; b) χT vs T; c) χ vs T; d) χ^{-1} vs T.

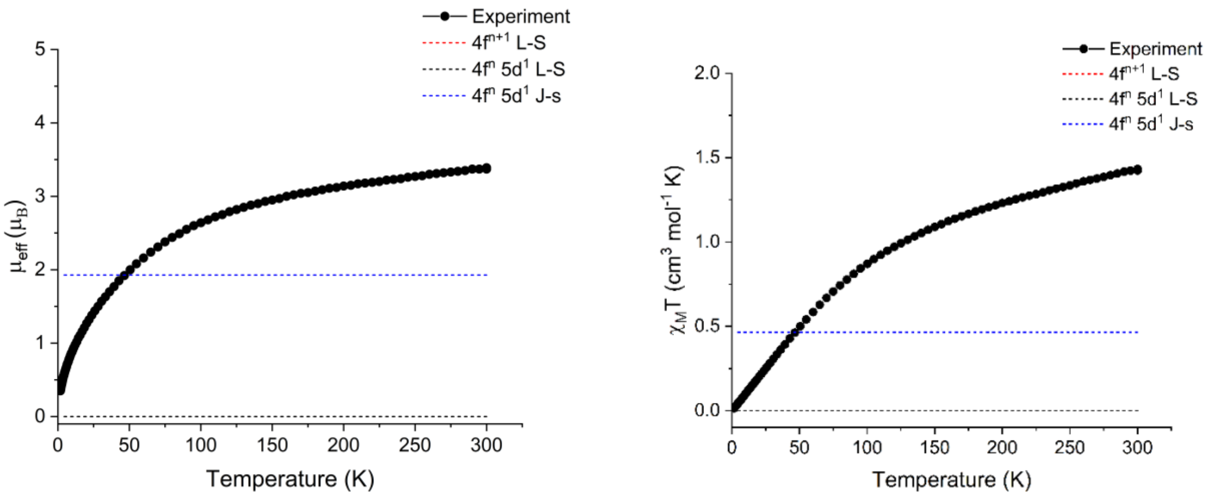

Figure S88. ***Left:*** Temperature-dependent SQUID effective magnetic moment (μ_B) vs Temperature (K) magnetometry data for **2Sm** (black circles) measured over the temperature range of 1.8 to 300 K. The black line is a guide to the eye only, and the dashed lines represent 4f^6 (red dashes), 4$f^5$5d^1 *L-S* (black dashes), and 4$f^5$5d^1 *J-s* (blue dashes) expected values – note that in this instance the blue and red dashed lines overlap. ***Right:*** Temperature-dependent SQUID χ_MT (cm^3 mol^{-1} K) vs Temperature (K) magnetometry data for **2Sm** (black circles) measured over the temperature range of 1.8 to 300 K. The black line behind the black circles is a guide to the eye only, and the dashed lines represent 4f^6 (red dashes), 4$f^5$5d^1 *L-S* (black dashes), and 4$f^5$5d^1 *J-s* (blue dashes) expected values – note that in this instance the blue and red dashed lines overlap.

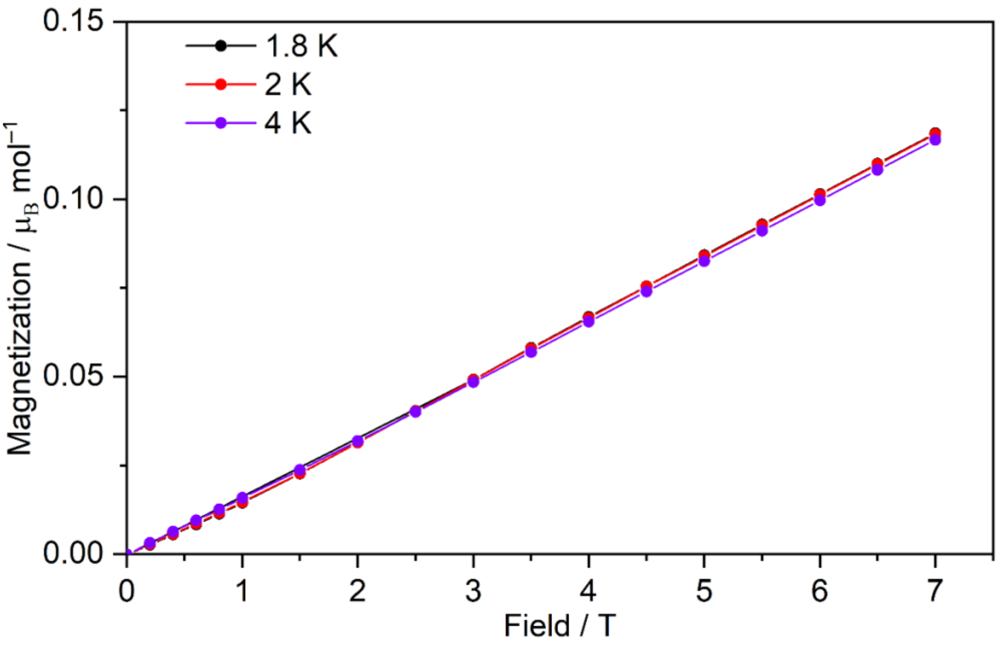

Figure S89. Magnetization vs Field data for **2Sm** at 1.8, 2, and 4 K. The lines are a guide to the eye only.

Divalent samarium, Sm(II), as in **2Sm**, could possess an electronic configuration of either $4f^6$ or $4f^5 5d^1$ – though the latter configuration is unlikely.[27] For the former, a $4f^6$ configuration would result in a formally diamagnetic $^7F_0$ ground state multiplet with population of close-lying excited states providing a non-zero magnetic moment at room temperature. For **2Sm**, the magnetic moment at 300 K is 3.38 μ_B (1.43 cm^3 mol^{-1} K), which decreases rapidly across the temperature range as excited states depopulate into a diamagnetic ground state, with values of 0.35 μ_B (0.015 cm^3 mol^{-1} K) at 1.8 K in agreement with previous examples of Sm(II) and formally a $4f^6$ electronic configuration (**Figure S88**).[28] Magnetization (M) *vs* Field (H) curves measured from 0–7 Tesla at 1.8, 2, and 4 K do not saturate and exhibit very low values of magnetization in line with a non-magnetic ground state (**Figure S89**).

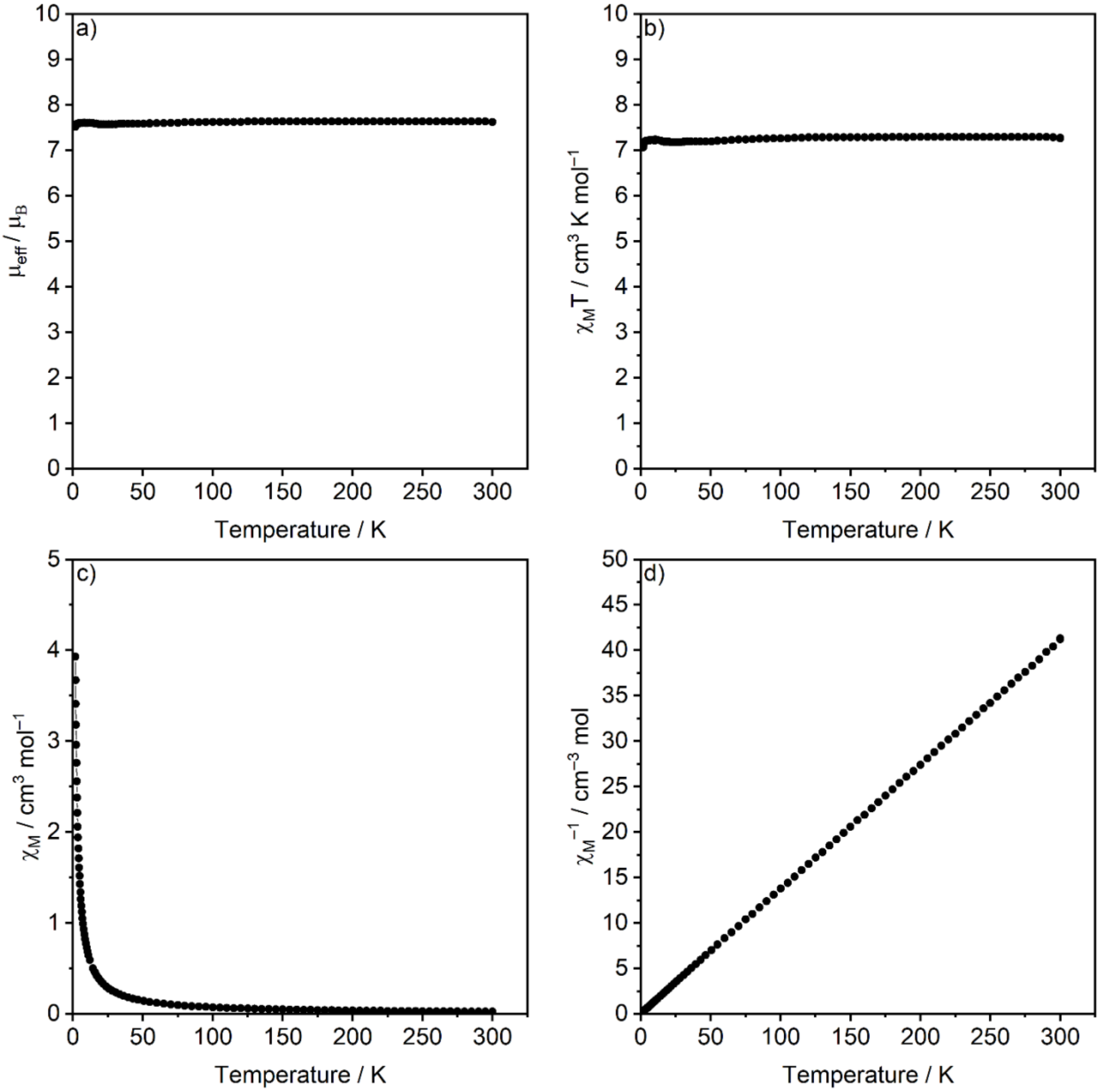

Figure S90. Variable-temperature SQUID magnetometry of **2Eu** over the temperature range 1.8-300 K: a) μ_{eff} vs T; b) χT vs T; c) χ vs T; d) χ^{-1} vs T.

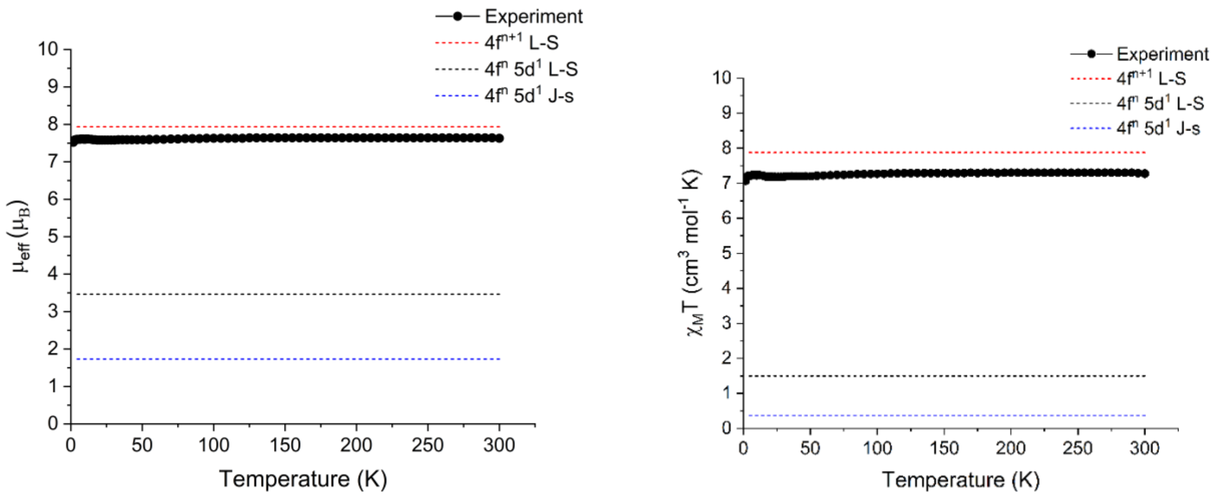

Figure S91. ***Left:*** Temperature-dependent SQUID effective magnetic moment (μ_B) vs Temperature (K) magnetometry data for **2Eu** (black circles) measured over the temperature range of 1.8 to 300 K. The black line is a guide to the eye only, and the dashed lines represent $4f^7$ (red dashes), $4f^6 5d^1$ *L-S* (black dashes), and $4f^6 5d^1$ *J-s* (blue dashes) expected values. ***Right:*** Temperature-dependent SQUID χ_MT (cm^3 mol^{-1} K) vs Temperature (K) magnetometry data for **2Eu** (black circles) measured over the temperature range of 1.8 to 300 K. The black line behind the black circles is a guide to the eye only, and the dashed lines represent $4f^7$ (red dashes), $4f^6 5d^1$ *L-S* (black dashes), and $4f^6 5d^1$ *J-s* (blue dashes) expected values.

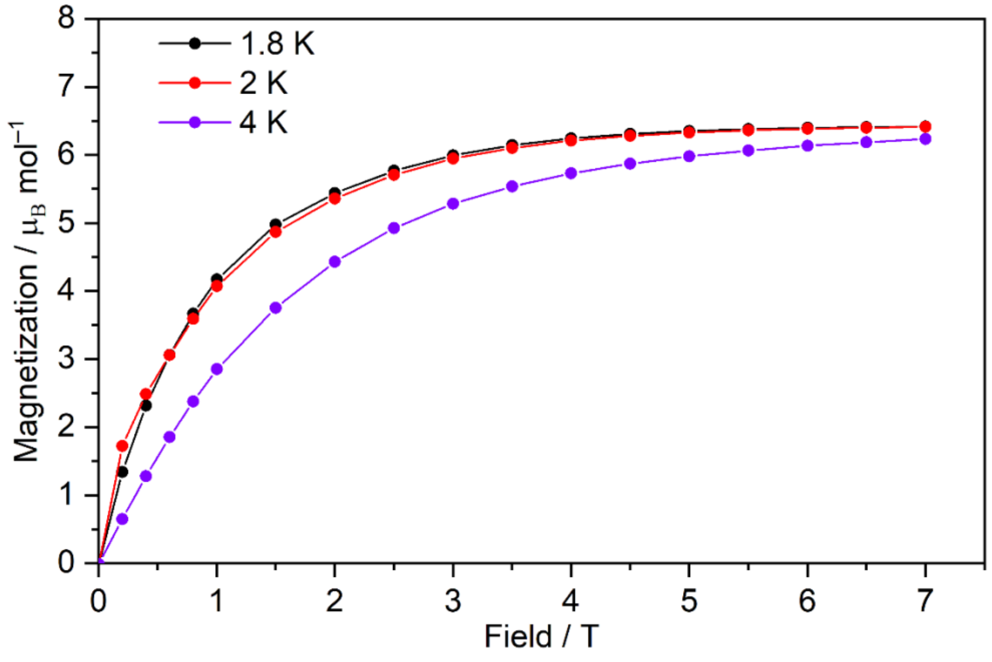

Figure S92. Magnetization vs Field data for **2Eu** at 1.8, 2, and 4 K. The lines are a guide to the eye only.

Divalent europium, Eu(II), as in **2Eu**, could possess an electronic configuration of either $4f^7$ or $4f^65d^1$ – though the latter configuration is unlikely.[27] DC magnetic susceptibility measurements of a powdered sample of **2Eu** exhibit a magnetic moment at 300 K which is in excellent agreement with the theoretical calculated value using the $4f^{n+1}$ *L-S* coupling scheme. The magnetic moment at 300 K is 7.63 μ_B (7.27 cm^3 mol^{-1} K), only slightly less than the expected value of 7.94 μ_B (7.88 cm^3 mol^{-1} K) based on a $4f^7$ $^8S_{7/2}$ Eu(II) ion. This value remains reasonably constant across the temperature range due to the half-filled occupation of the 4*f* valence sub-shell, settling on a value of 7.52 μ_B (7.07 cm^3 mol^{-1} K) at 1.8 K (**Figure S91**).[29-31] In the case of a Eu(II) $4f^65d^1$ electronic configuration, an L-S coupling scheme would give values of 3.46 μ_B (1.50 cm^3 mol^{-1} K) at 300 K, whereas a *J-s* coupling scheme would result in idealised values of 1.73 μ_B (0.375 cm^3 mol^{-1} K) at 300 K. In agreement with the Kramers ion nature of the $4f^7$ $^8S_{7/2}$ ground multiplet, magnetization (M) vs field (H) curves measured from 0–7 Tesla at 1.8, 2, and 4 K saturate up to ~6.0 μ_B mol^{-1} (**Figure S92**).

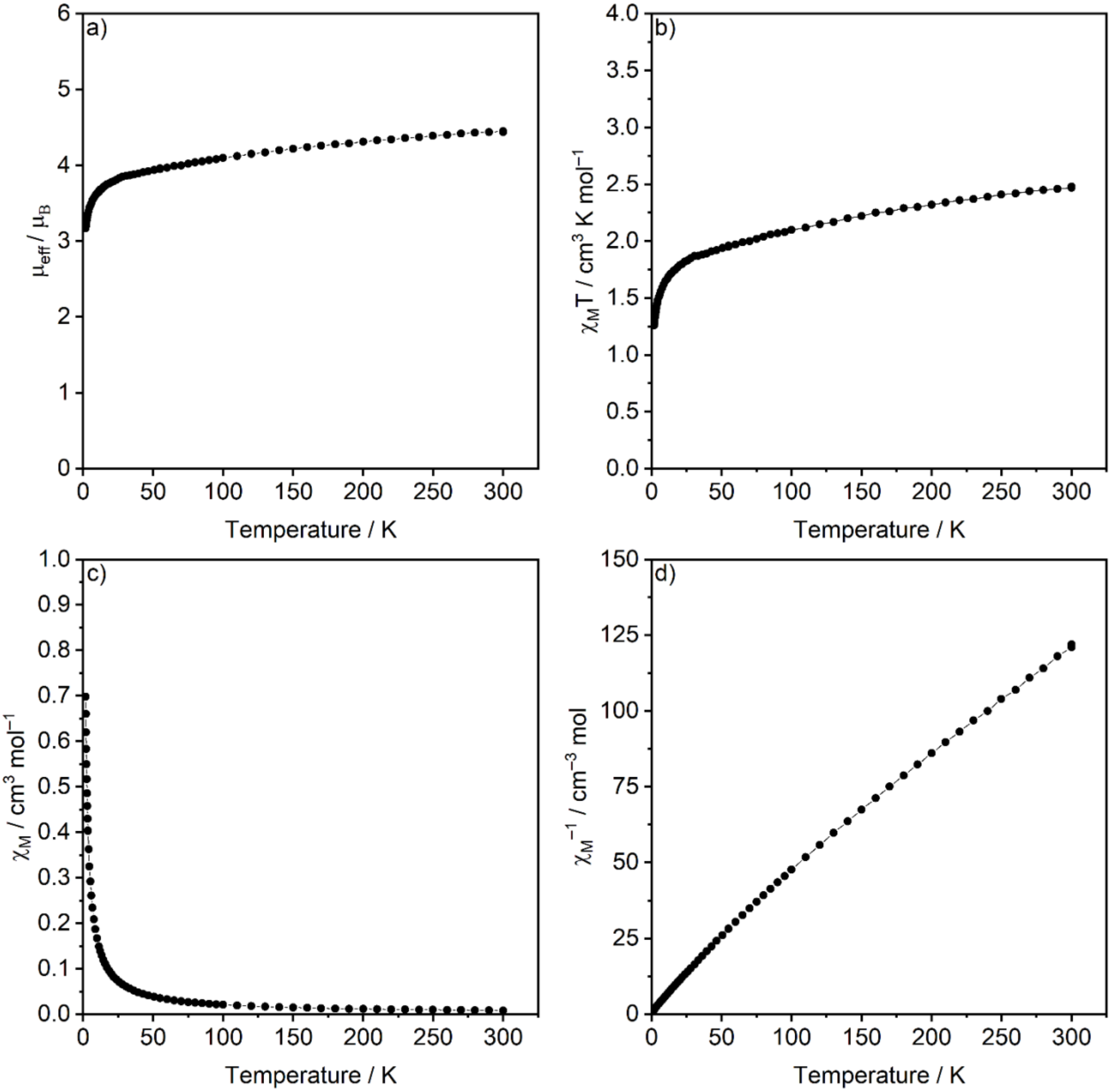

Figure S93. Variable-temperature SQUID magnetometry of **2Tm** over the temperature range 1.8-300 K: a) μ_{eff} vs T; b) χT vs T; c) χ vs T; d) χ^{-1} vs T.

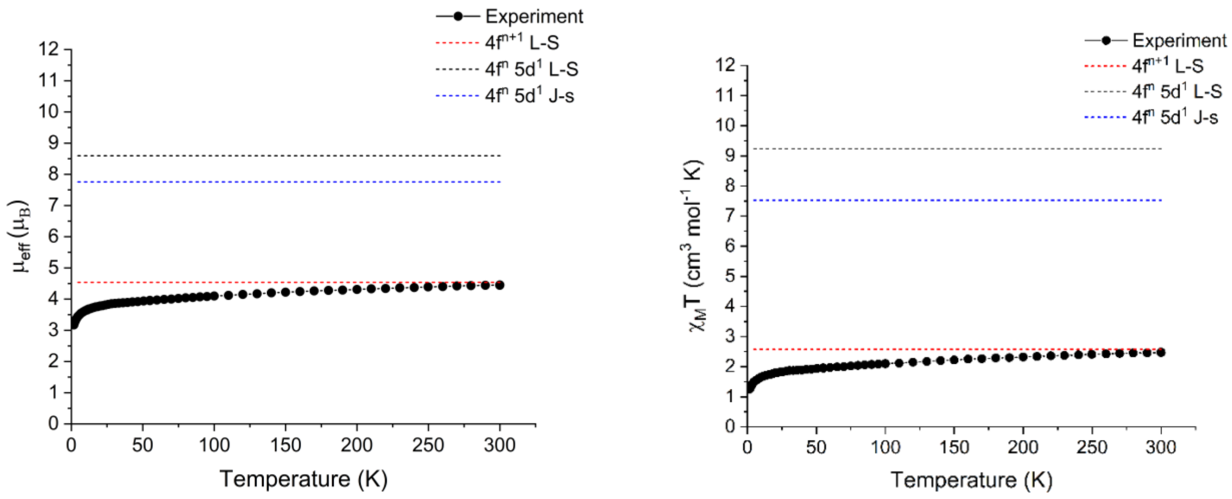

Figure S94. ***Left:*** Temperature-dependent SQUID effective magnetic moment (μ_B) vs Temperature (K) magnetometry data for **2Tm** (black circles) measured over the temperature range of 1.8 to 300 K. The black line is a guide to the eye only, and the dashed lines represent $4f^{13}$ (red dashes), $4f^{12}5d^1$ *L-S* (black dashes), and $4f^{12}5d^1$ *J-s* (blue dashes) expected values. ***Right:*** Temperature-dependent SQUID $\chi_M T$ (cm^3 mol^{-1} K) vs Temperature (K) magnetometry data for **2Tm** (black circles) measured over the temperature range of 1.8 to 300 K. The black line behind the black circles is a guide to the eye only, and the dashed lines represent $4f^{13}$ (red dashes), $4f^{12}5d^1$ *L-S* (black dashes), and $4f^{12}5d^1$ *J-s* (blue dashes) expected values.

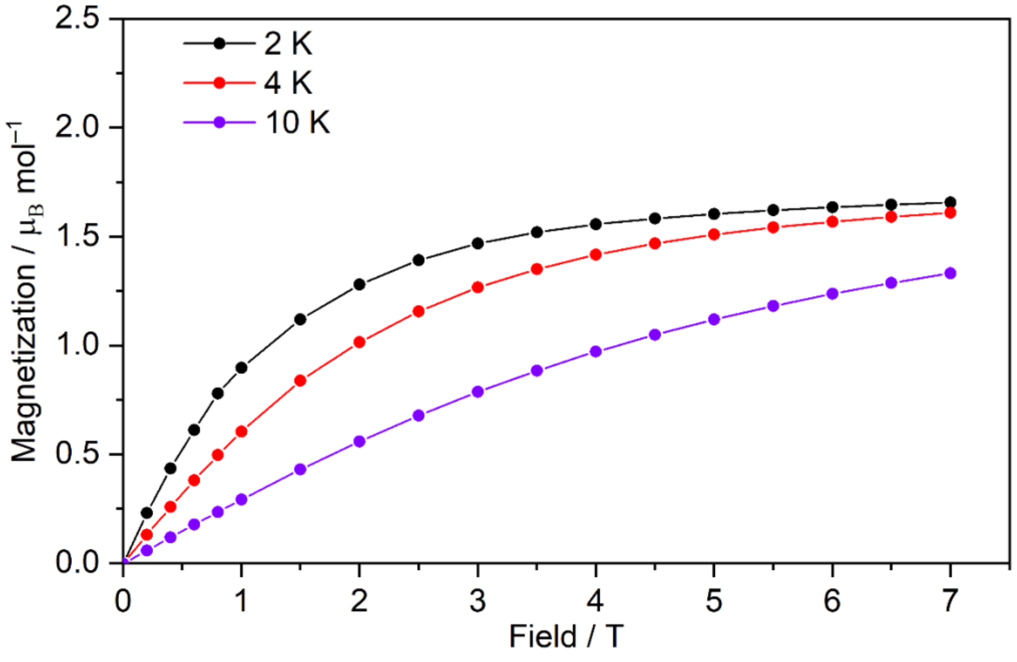

Figure S95. Magnetization vs Field data for **2Tm** at 2, 4, and 10 K. The lines are a guide to the eye only.

Divalent thulium, Tm(II), as in **2Tm**, could possess an electronic configuration of either $4f^{13}$ or $4f^{12}5d^{1}$ – though the latter configuration is unlikely.[27] DC magnetic susceptibility measurements of a powdered sample of **2Tm** exhibit a magnetic moment at 300 K which is in excellent agreement with the theoretical calculated value using the $4f^{n+1}$ *L*-*S* coupling scheme. The magnetic moment at 300 K is 4.45 μ_B (2.47 cm^3 mol^{-1} K), only slightly less than the expected value of 4.54 μ_B (2.57 cm^3 mol^{-1} K) based on a $4f^{13}$ $^2F_{7/2}$ Tm(II) ion. This value decreases across the temperature range due to progressive thermal depopulation of crystal field states settling on a value of 3.17 μ_B (1.26 cm^3 mol^{-1} K) at 1.8 K (**Figure S94**), in agreement with an isolated ground $^2F_{7/2}$ multiplet well separated from excited states and in accordance to that previously reported for Tm(II) and Yb(III) complexes.[30-32] In the case of a Tm(II) $4f^{12}5d^{1}$ electronic configuration, an *L*-*S* coupling scheme would give values of 8.59 μ_B (9.23 cm^3 mol^{-1} K) at 300 K, whereas a *J*-*s* coupling scheme would result in idealised values of 7.76 μ_B (7.52 cm^3 mol^{-1} K) at 300 K, approximately double of that observed experimentally. In accordance with the Kramers ion nature of the $^2F_{7/2}$ ground multiplet, the magnetization versus field data saturates at both 2 and 4 K up to ~1.60 μ_B mol^{-1} (**Figure S95**).

Magnetometry data and discussion for ***2Sc****,* ***2Y****, and* ***2La***

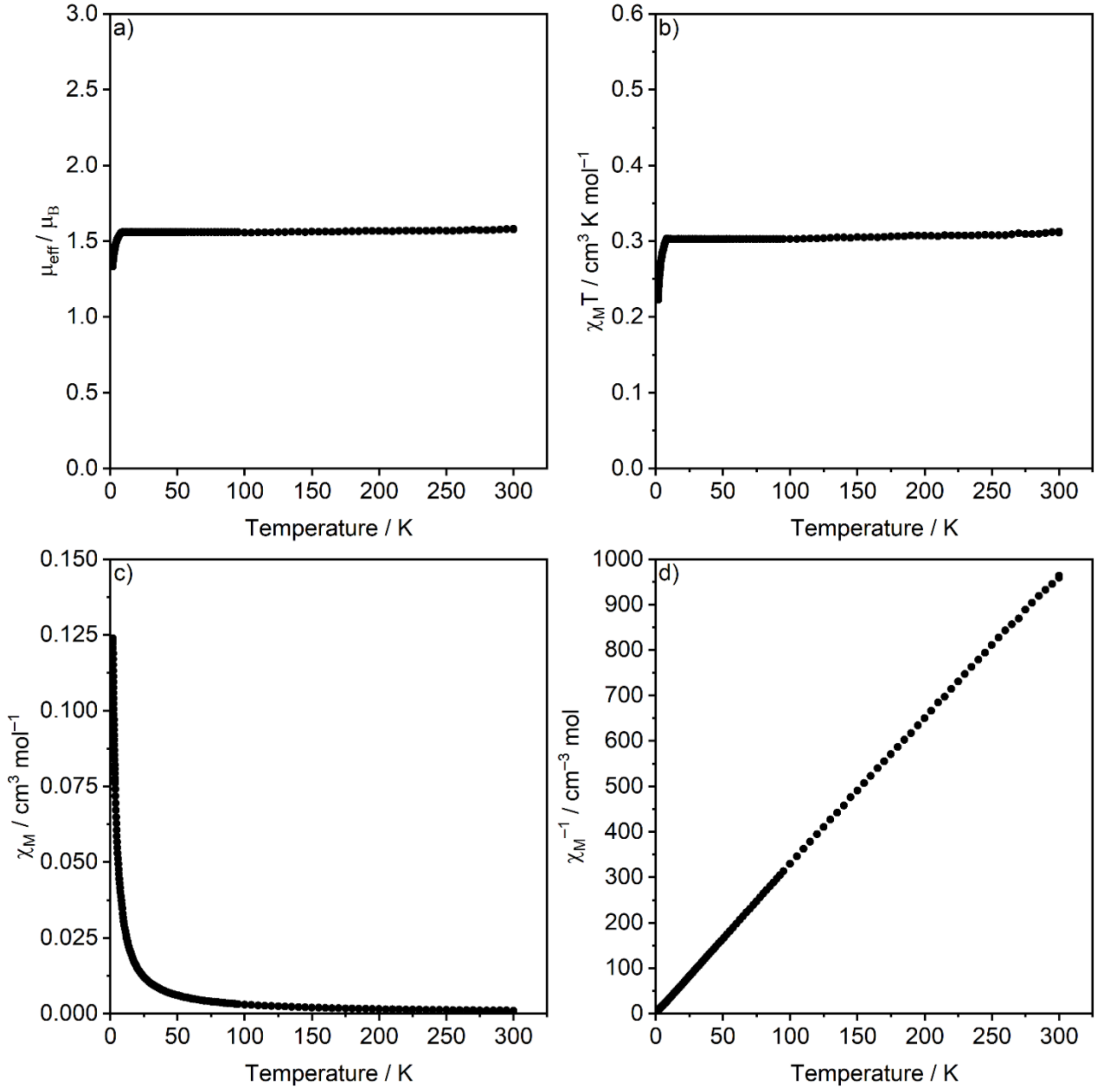

Figure S96. Variable-temperature SQUID magnetometry of **2Sc** over the temperature range 1.8-300 K: a) μ_{eff} vs T; b) χT vs T; c) χ vs T; d) χ^{-1} vs T.

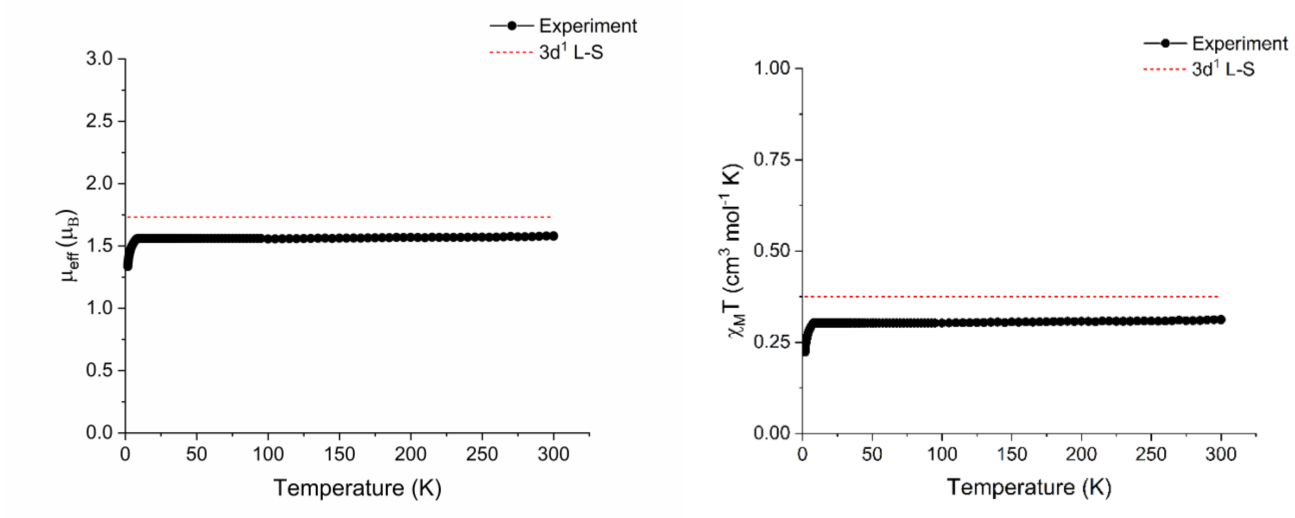

Figure S97. ***Left:*** Temperature-dependent SQUID effective magnetic moment (μ_B) vs Temperature (K) magnetometry data for **2Sc** (black circles) measured over the temperature range of 1.8 to 300 K. The black line is a guide to the eye only, and the dashed line represents d^1 (S = $^1/_2$, red dashes) expected value for a $g = 2$ system. ***Right:*** Temperature-dependent SQUID χ_MT (cm^3 mol^{-1} K) vs Temperature (K) magnetometry data for **2Sc** (black circles) measured over the temperature range of 1.8 to 300 K. The black line behind the black circles is a guide to the eye only, and the dashed line represents d^1 (S = $^1/_2$, red dashes) expected value for a $g = 2$ system.

DC magnetic susceptibility measurements of a powdered sample of **2Sc** exhibit a magnetic moment at 300 K which is in good agreement, although marginally supressed, with respect to the theoretical calculated value using the spin-only formula (S = $^1/_2$ = 1.73 μ_B; 0.375 cm^3 mol^{-1} K; g = 2). The magnetic moment at 300 K is 1.58 μ_B (0.31 cm^3 mol^{-1} K). This value remains constant across most of the temperature range before a rapid drop-off below 8 K settling to 1.34 μ_B at 1.8 K. Repeated measurements across multiple samples from independent syntheses show small variations in this low-temperature drop-off. This suggests that rather than this behaviour being a result of an electronic property inherent to the molecular system, it is in fact a measurement issue whereby complete thermal equilibration of the sample is not achieved. This effect appears to have a more noticeable impact in systems for which the paramagnetic component is weak in comparison to the overall diamagnetism of the sample: ligand, sample containment etc.

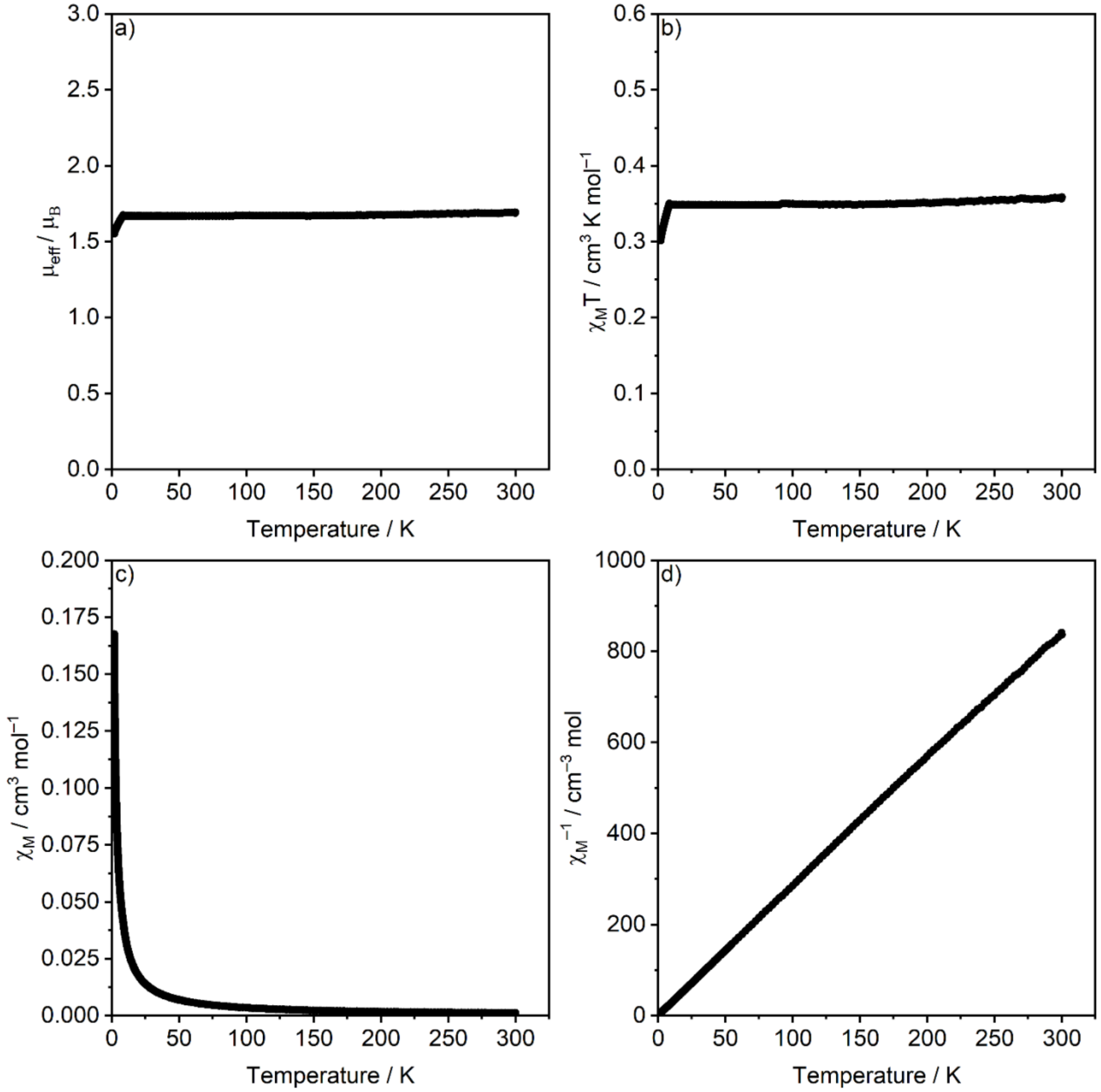

Figure S98. Variable-temperature SQUID magnetometry of **2Y** over the temperature range 1.8-300 K: a) μ_{eff} vs T; b) χT vs T; c) χ vs T; d) χ^{-1} vs T.

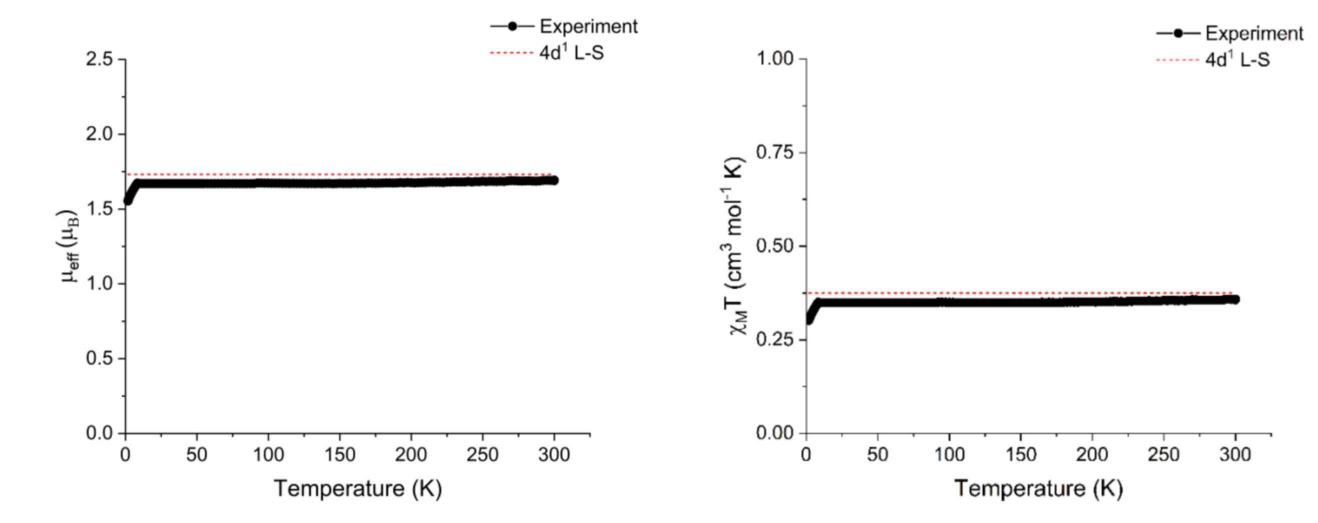

Figure S99. ***Left:*** Temperature-dependent SQUID effective magnetic moment (μ_B) vs Temperature (K) magnetometry data for **2Y** (black circles) measured over the temperature range of 1.8 to 300 K. The black line is a guide to the eye only, and the dashed line represents d^1 (S = $^1/_2$, red dashes) expected value for a $g = 2$ system. ***Right:*** Temperature-dependent SQUID $\chi_M T$ (cm^3 mol^{-1} K) vs Temperature (K) magnetometry data for **2Y** (black circles) measured over the temperature range of 1.8 to 300 K. The black line behind the black circles is a guide to the eye only, and the dashed line represents d^1 (S = $^1/_2$, red dashes) expected value for a $g = 2$ system.

Divalent yttrium, Y(II), as in **2Y**, should possess an electronic configuration of $4d^1$. DC magnetic susceptibility measurements of a powdered sample of **2Y** exhibit a magnetic moment at 300 K, which is in very good agreement with the theoretical value using the spin-only formula (S = $^1/_2$ = 1.73 μ_B; 0.375 cm^3 mol^{-1} K; g = 2). The magnetic moment at 300 K is 1.69 μ_B (0.36 cm^3 mol^{-1} K). This value remains constant across most of the temperature range before a rapid drop-off below 8 K, settling at 1.55 μ_B at 1.8 K. As for **2Sc**, repeated measurements across multiple samples from independent syntheses show variations in this low-temperature drop-off.

2Y has been previously reported by Odom and Demir.[16] The reported magnetic data do not agree with our own. The prior report shows much larger room temperature magnetic moments and temperature-dependent behaviour, which was attributed to temperature-independent paramagnetism.

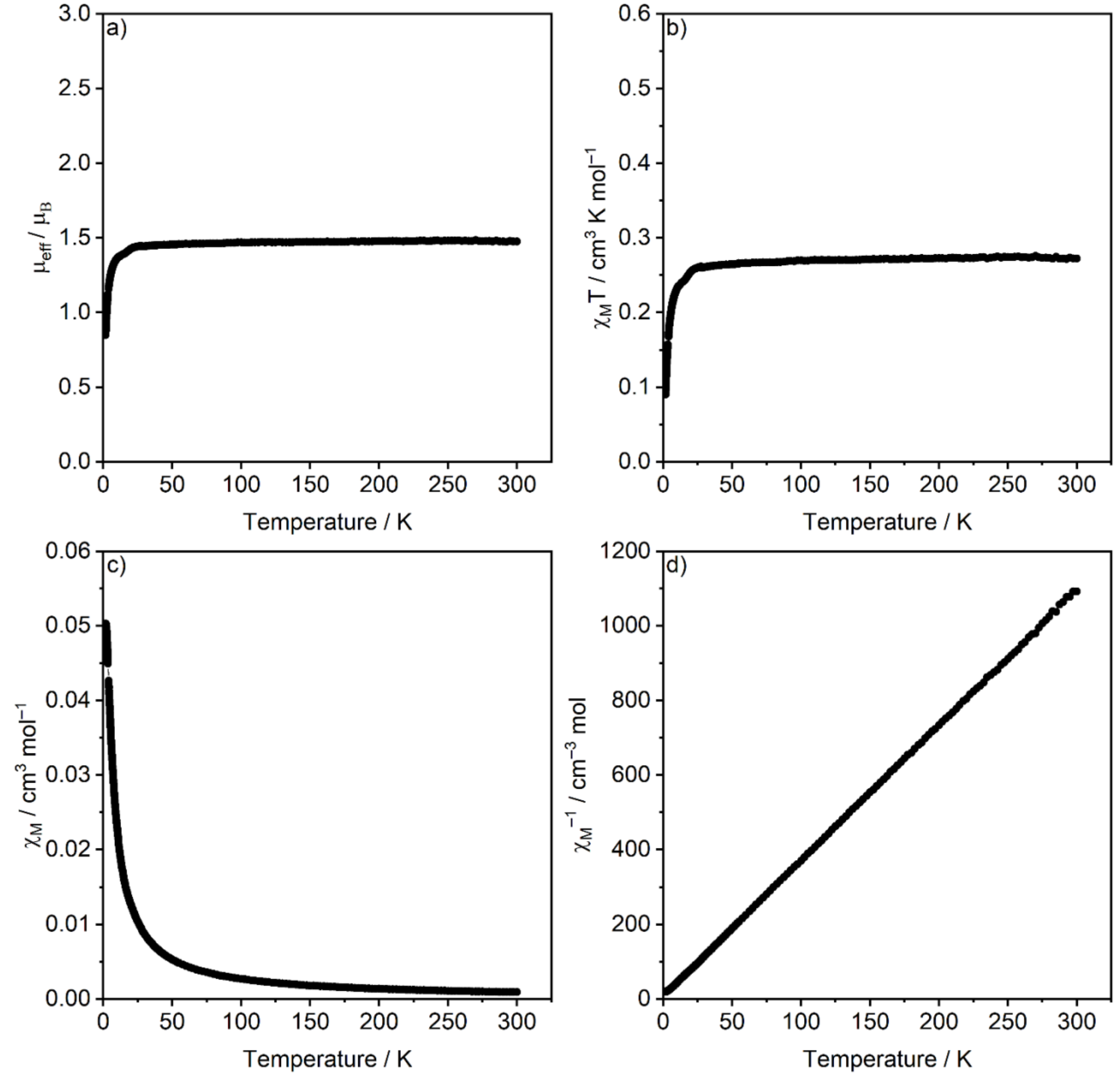

Figure S100. Variable-temperature SQUID magnetometry of **2La** over the temperature range 1.8-300 K: a) μ_{eff} vs T; b) χT vs T; c) χ vs T; d) χ^{-1} vs T.

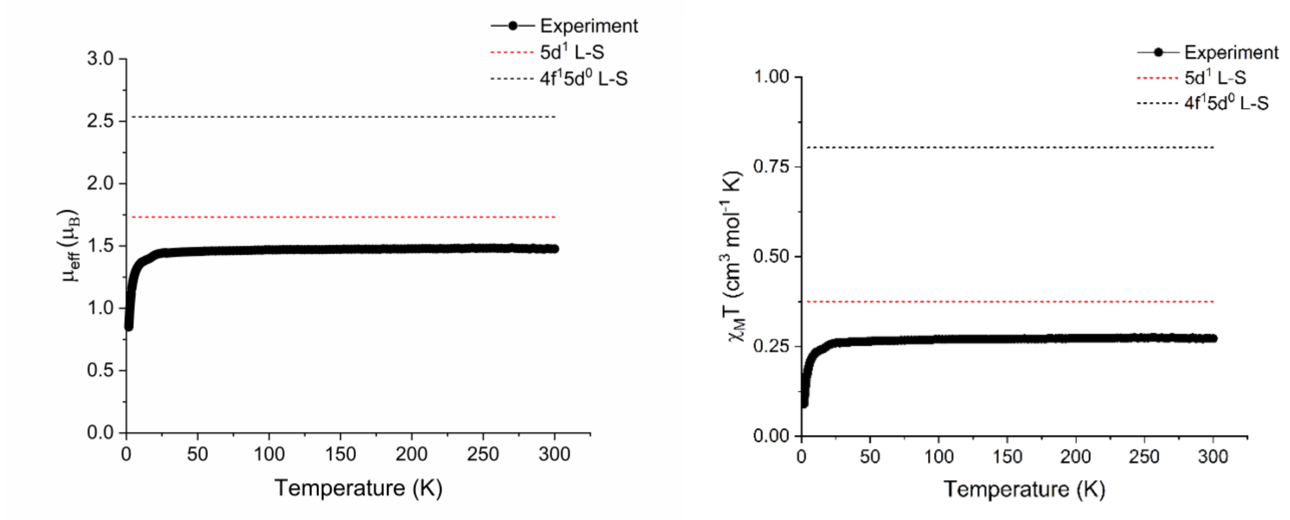

Figure S101. ***Left:*** Temperature-dependent SQUID effective magnetic moment (μ_B) vs Temperature (K) magnetometry data for **2La** (black circles) measured over the temperature range of 1.8 to 300 K. The black line is a guide to the eye only, and the dashed line represents d^1 (S = $^1/_2$, red dashes) expected value for a $g = 2$ system, and $4f^1$ (black dashes) expected values. ***Right:*** Temperature-dependent SQUID $\chi_M T$ ($cm^3\ mol^{-1}$ K) vs Temperature (K) magnetometry data for **2La** (black circles) measured over the temperature range of 1.8 to 300 K. The black line behind the black circles is a guide to the eye only, and the dashed line represents d^1 (S = $^1/_2$, red dashes) expected value for a $g = 2$ system, and $4f^1$ (black dashes) expected values.

Divalent lanthanum, La(II), could possess an electronic configuration of either $4f^1$ or $4f^0 5d^1$. Though the former is unlikely. DC magnetic susceptibility measurements of a powdered sample of **2La** exhibit a magnetic moment at 300 K which is in good agreement, although supressed, with respect to the theoretical value using the spin-only formula ($S = {}^1/_2 = 1.73\ \mu_B$; 0.375 $cm^3\ mol^{-1}$ K; $g = 2$). The magnetic moment at 300 K is 1.52 μ_B (0.29 $cm^3\ mol^{-1}$ K). This value remains fairly constant across most of the temperature range before a rapid drop-off below 10 K, settling at a low value of 0.85 μ_B (0.09 $cm^3\ mol^{-1}$ K) at 1.8 K. Repeated measurements across multiple samples from independent syntheses show consistency in the low-temperature drop-off and the magnetic moment value at 1.8 K, suggestive that this behaviour is due to the electronic properties inherent to the molecular system, rather than a measurement issue. This is in contrast to our observations with **2Sc** and **2Y** above.

However, given the proximity of metal centres in **2La** (10.1745(8) Å) and the extensive spin density on the ligands, it is likely that intramolecular interactions explain the low-temperature deviations.

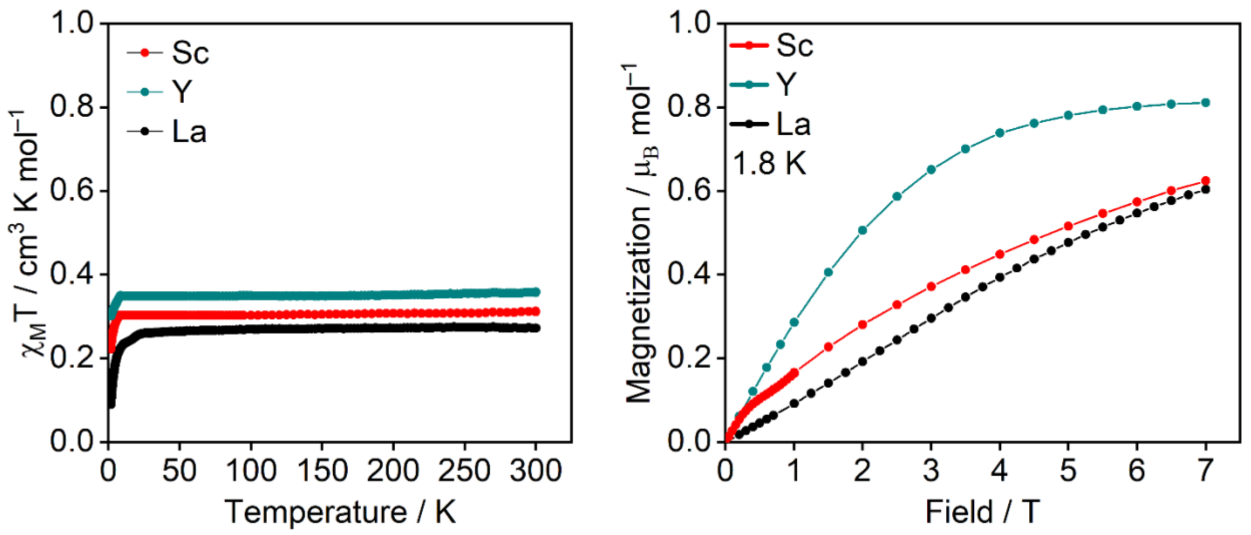

Figure S102. ***Left:*** Temperature-dependent SQUID $\chi_M T$ (cm^3 mol^{-1} K) vs Temperature (K) magnetometry data for **2Sc** (red circles), **2Y** (teal circles), and **2La** (black circles) measured over the temperature range of 1.8 to 300 K. ***Right:*** Magnetization vs Field data for **2Sc** (red circles), **2Y** (teal circles), and **2La** (black circles) at1.8 K. The solid lines are to guide the eye.

Given the proximity of metal centres in these complexes (**2Sc** (9.976(2) Å), **2La** (10.1745(8) Å), and **2Y** (12.3175(2) Å) and the extensive spin density on the ligands, it is likely that intermolecular interactions explain the low-temperature deviations.

S9. Density Functional Theory calculations

Density Functional Theory – General considerations

Unrestricted Kohn-Sham calculations were performed on *S* = ½ complexes [M(NHAr^{iPr6})$_2$] (**2M**, M = Sc, Y, La), *S* = 3 [Sm(NHAr^{iPr6})$_2$] (**2Sm**), *S* = $^7/_2$ [Eu(NHAr^{iPr6})$_2$] (**2Eu**), *S* = $^1/_2$ [Tm(NHAr^{iPr6})$_2$] (**2Tm**), and *S* = 0 [Yb(NHAr^{iPr6})$_2$] (**2Yb**) at the DFT level using the ORCA 5.0 quantum chemistry program suite.[33] Other multiplicities for **2Eu** (*S* = $^5/_2$) and **2Tm** (*S* = $^3/_2$) were considered, but were discounted due to experimental verification of their ground states by SQUID magnetometry. Geometry optimisations were performed using the TPSSh hybrid meta-generalised gradient density functional (meta-GGA)[34, 35] with Grimme's D3BJ dispersion correction,[36-38] and the resolution of the identity 'chain of spheres' (RIJCOSX) approximation.[39, 40] For most calculations, elements Y, La, Sm, Eu, Tm, and Yb were treated with a segmented all-electron relativistically contracted (SARC) Dougless-Kroll-Hess second order (DKH-def2) basis set at the triple-ζ level (SARC-DKH-TZVP),[41] Sc was treated with a DKH-def2 basis set at the triple-ζ level (DKH-def2-TZVP),[42] while a split-valence polarised basis set of double-ζ quality (DKH-def2-SVP) was used for all other atoms.[42] A SARC/J auxiliary basis set was used for each atom type as appropriate.[41, 43] No symmetry constraints were imposed. Occasionally the SlowConv switch was used to ensure SCF convergence, along with the DefGrid3 (tight integration grid) switch.

For property calculations, additional functionals (B3PW91,[44] CAM-B3LYP,[45] PBE,[46, 47] PBE0,[48, 49] TPSSh[34, 35], TPSS0[50]) and alternative relativistic treatments (DKH, and ZORA – Zeroth Order Regular Approximation) were investigated,[51-53] along with the use of larger basis sets on all atoms at either the triple-ζ (H, C, N) or quadruple-ζ (Sc, Y, La) level and with additional polarisation functions as appropriate. The basis sets explored were as follows: H, C, N (DKH/ZORA-def2-TZVP); Sc (ma-DKH/ZORA-def2-QZVPP); Y (SARC-DKH/ZORA-TZVPP); also Sc, Y (Sapporo-DKH3-QZP-2012);[54] La, Sm, Eu, Tm, Yb (SARC2-DKH/ZORA-QZVP).[55] All were used with their default settings within ORCA 5.0.4. We make no claims regarding the accuracy of the various basis sets

and functionals herein, but have chosen them due to their success in describing similar complexes in the literature.[56]

Notes on DFT geometry optimisations

Complexes **2Y**, **2Eu**, and **2Yb** have true C_2 symmetry by single-crystal X-ray diffraction (space group $C2/c$ or $C2$), with one (**2Y**) or two (**2Eu**, **2Yb** – on two unique metals) ligands in the asymmetric unit with little or no crystallographic disorder of the pendant *i*Pr groups. Meanwhile, complexes **2Sc**, **2Sm**, and **2Tm** have C_1 symmetry (spacegroup $P\bar{1}$) with minor crystallographic disorder on ligand substituents not within the primary coordination sphere. For these six structures, the largest disorder component was taken, and only the H-atom positions were optimised, while all remaining atom positions were fixed.

Complex **2La** has C_1 symmetry (spacegroup $P\bar{1}$), with one whole molecule in the asymmetric unit. One of the metal-bound C_6 arene rings is slightly buckled (fold angle *ca*. 10°) and is also slightly disordered in the arene *xy* plane which could not be easily resolved as two well-separated components by single-crystal X-ray diffraction – instead they have roughly equal occupancy, meaning there was no good reason to select one over the other for calculations. The *para-i*Pr group on this ring is also disordered over two positions in a roughly 50:50 ratio. As such, in addition to all H-atoms for **2La**, we have also optimised the cartesian coordinates of the 9 C-atoms that make up this C_6 ring and the *para-i*Pr group. A frequency analysis was not performed on any of these structures as the coordinates of most of the atoms were not optimised.

In addition to constrained geometry optimisations, full optimisations were performed on complexes **2Sc**, **2Y**, **2La**, **2Sm**, **2Eu**, **2Tm**, and **2Yb** in the gas phase starting from the single-crystal X-ray diffraction structures. This was intended to further validate the DFT methodology for investigating these complexes. Where disorder is present, the largest occupancy component was used. Where multiple molecules are present in the asymmetric unit, “M1” was chosen arbitrarily as the starting point. Due to the large size of the molecules and associated computational cost, we restricted this

work to using the PBE functional,[46, 47] and a split-valence all-electron polarised basis set of double-ζ quality (def2-SVP) for all other atoms (Sc, N C, H),[57] except for the metal atoms where the Stuttgart-Dresden effective core potentials were used (Y,[58] La,[59] Sm,[60] Eu,[60] Tm,[60] Yb,[60] as appropriate). Convergence tolerances were set to the default parameters within ORCA 5.0.4. Analytical frequency calculations show all are true local minima with the exception of **2Eu** where repeated attempts resulted in a structure with one or two very energy (ca. 4 to 15 cm^{-1}) imaginary vibrational modes. Very tight convergence parameters did not resolve this. However, we note that the solid-state structure presents with two half-molecules in the asymmetric unit with negligible metrical difference which are however significant enough to lower the symmetry. Clearly the potential energy surface is very shallow for this structure.

Comparisons of experimental and calculated molecular structures are shown in the Tables below.

Table S15. Selected experimental and calculated lengths (Å) and angles (°) for **2Sc**.

		**2Sc (SC XRD)**	**2Sc (Calculated)**	**Δ (Calc – exp)**
**M–N**	N(1)	2.0884(11)	2.0900	0.0016
	N(2)	2.0678(10)	2.0746	0.0068
**M–C$_{6\text{-range}}$**	Ring(1)	2.3913(12)–		
**M–C**	C(8)	2.6304(14)	2.4240–	
	C(11)	2.6067	0.0327	
**M–C$_{6\text{-centroid}}$**	Ring(1)	–0.0237		
	Ring(2)	2.3913(12)	2.4240	0.0327
**N–M–N**		2.5418(13)	2.5378	–0.0040
**C$_{6\text{-centroid}}$–M–C$_{6\text{-centroid}}$**		2.1006(7)	2.0883	–0.0123
**Arene fold angle**		11.43(11)	8.443	–2.987

Table S16. Selected experimental and calculated lengths (Å) and angles (°) for **2Y**.

		**2Y (SC XRD)**	**2Y (Calculated)**	**Δ (Calc – exp)**
**M–N**	N(1)	2.2600(12)	2.2812	0.0212
	N(2)	2.2600(12) [A]	2.2812	0.0212
**M–C$_{6\text{-range}}$**	Ring(1)	2.7276(14)–	2.7176–	–0.0099
		2.9273(15)	2.9613	0.0340
	Ring(2)	2.7276(14)–	2.7177–	–0.0099
		2.9273(15) [A]	2.9615	0.0342
**M–C**	C(8)	2.7684(14)	2.7522	–0.0162
			2.7522	–0.0162
	C(11)	2.7859(15)	2.7363	–0.0496
			2.7364	–0.0496
**M–C$_{6\text{-centroid}}$**	Ring(1)	2.4481(6)	2.4156	–0.0326
	Ring(2)	2.4481(6) [A]	2.4156	–0.0325
**N–M–N**		101.87(7)	104.18	2.3068
**C$_{6\text{-centroid}}$–M–C$_{6\text{-centroid}}$**		133.40(3)	136.98	3.5840
**Arene fold angle**		7.27(12)	8.76	1.49
		7.27(12)	8.79	1.52

[A] The solid-state structure is C_2 symmetric and so there is a single unique ligand. The geometry of the calculated structure was not constrained.

Table S17. Selected experimental and calculated lengths (Å) and angles (°) for **2La**.

		**2La (SC XRD)**	**2La (Calculated)**	**Δ (Calc – exp)**
**M–N**	N(1)	2.395(3)	2.449	0.054
	N(2)	2.434(3)	2.482	0.048
**M–$C_{6\text{-range}}$**	Ring(1)	2.778(16)–	2.876–	0.098
		2.971(9)	2.968	–0.003
	Ring(2)	3.047(3)–	2.979–	–0.068
		3.240(3)	3.210	–0.030
**M–C**	C(8)	2.843(13)	2.876	0.033
	C(11)	2.903(12)	2.867	–0.036
**M–$C_{6\text{-centroid}}$**	Ring(1)	2.479(5)	2.542	0.063
	Ring(2)	2.8348(12)	2.7394	–0.095
**N–M–N**		113.60(10)	112.19	–1.4110
**$C_{6\text{-centroid}}$–M–$C_{6\text{-centroid}}$**		151.47(12)	157.54	6.0725
**Arene fold angle**		12.9(9)	7.19	–5.71
		0.6(2)	2.67	2.07

Table S18. Selected experimental and calculated lengths (Å) and angles (°) for **2Sm**.

		**2Sm (SC XRD)**	**2Sm (Calculated)**	**Δ (Calc – exp)**
**M–N**	N(1)	2.412(2)	2.395	–0.017
	N(2)	2.425(2)	2.414	–0.011
**M–$C_{6\text{-range}}$**	Ring(1)	2.955(3)–	2.911–	–0.044
		3.160(3)	3.159	–0.001
	Ring(2)	2.953(3)–	2.981–	0.028
		3.201(3)	3.256	0.055
**M–$C_{6\text{-centroid}}$**	Ring(1)	2.7032(12)	2.6607	–0.0425
	Ring(2)	2.7265(12)	2.7543	0.02782
**N–M–N**		115.25(9)	113.45	–1.8038
**$C_{6\text{-centroid}}$–M–$C_{6\text{-centroid}}$**		153.70(4)	154.08	0.3825

Table S19. Selected experimental and calculated lengths (Å) and angles (°) for **2Eu**.

		**2Eu (SC XRD)**	**2Eu (Calculated)**	**Δ (Calc – exp)**
**M–N**	N(1)	2.411(4)	2.395	–0.016
	N(2)	2.411(4)	2.398	–0.013
**M–C$_{6\text{-range}}$**	Ring(1)	2.972(4)–	2.947–	–0.025
		3.176(5) [A]	3.176	0
	Ring(2)	2.972(4)–	2.933–	–0.039
		3.176(5) [A]	3.178	0.002
**M–C$_{6\text{-centroid}}$**	Ring(1)	2.7604(17)	2.7108	–0.0496
	Ring(2)	2.7604(17) [A]	2.7015	–0.0589
**N–M–N**		107.5(2)	106.9	–0.600
**C$_{6\text{-centroid}}$–M–C$_{6\text{-centroid}}$**		148.88(10)	148.04	–0.8355

[A] The solid-state structure is C_2 symmetric and so there is a single unique ligand. The geometry of the calculated structure was not constrained.

Table S20. Selected experimental and calculated lengths (Å) and angles (°) for **2Tm**.

		**2Tm (SC XRD)**	**2Tm (Calculated)**	**Δ (Calc – exp)**
**M–N**	N(1)	2.3060(17)	2.2959	-0.0101
	N(2)	2.3169(18)	2.3046	-0.0123
**M–C$_{6\text{-range}}$**	Ring(1)	2.8015(19)–	2.8242–	0.0227
		2.971(2)	3.1658	0.1948
	Ring(2)	2.819(2)–	2.8771–	0.0581
		3.118(2)	3.1899	0.0719
**M–C$_{6\text{-centroid}}$**	Ring(1)	2.5579(9)	2.6128	0.0549
	Ring(2)	2.5969(9)	2.6597	0.0628
**N–M–N**		116.45(7)	115.56	-0.8892
**C$_{6\text{-centroid}}$–M–C$_{6\text{-centroid}}$**		148.37(3)	151.82	3.4495

Table S21. Selected experimental and calculated lengths (Å) and angles (°) for **2Yb**.

		2Yb (SC XRD)	2Yb (Calculated)	Δ (Calc – exp)
**M–N**	N(1)	2.310(6)	2.294	–0.016
	N(2)	2.310(6) [A]	2.294	–0.016
**M–$C_{6\text{-range}}$**	Ring(1)	2.840(6)–	2.787–	–0.053
		3.047(6)	2.988	–0.059
	Ring(2)	2.840(6)–	2.789–	–0.051
		3.047(6) [A]	2.990	–0.057
**M–$C_{6\text{-centroid}}$**	Ring(1)	2.629(2)	2.512	–0.117
	Ring(2)	2.629(2) [A]	2.514	–0.145
**N–M–N**		108.5(3)	107.6	–0.918
**$C_{6\text{-centroid}}$–M–$C_{6\text{-centroid}}$**		145.63(14)	146.17	0.5395

TD-DFT methodology

Starting with the partially optimised structures obtained using the methods described above we have first screened the calculated UV-Vis-NIR spectroscopic properties of **2Sc**, **2Y**, and **2La** using both Time Dependent Density Functional Theory (TD-DFT) calculations and the Simplified-TD-DFT (sTD-DFT) methodology within the ORCA 5.0 quantum chemistry program suite.[33] For comparison to the experimental data the Conductor-like Continuum Polarization Model (C-PCM) was applied throughout, with values for the dielectric constant (ε) and refractive index (n_D) set to those of Et_2O (4.33 and 1.3524) as this was the solvent used in UV-Vis-NIR measurements.[61] By first examining two different popular functionals (PBE[46, 47] and TPSSh[34, 35]) with each complex with both TD-DFT and sTD-DFT methodologies, we found there to be minimal differences between the two approaches in most cases when comparing calculated and experimental UV-Vis-NIR spectra. **Figure S103** to **Figure S105** show the comparison between experimental spectra, and the (s)TD-DFT methodologies for **2Sc**, **2Y**, and **2La**. Simulated spectra were obtained by taking the TD-DFT (or sTD-DFT) output transitions and applying a Gaussian lineshape with a broadening coefficient of $25 \times \sqrt{E}$ (where E is energy in cm^{-1}) using the orca_mapspc module within the ORCA 5.0.4 software suite. TD-DFT calculations were performed with 120 roots.

*TD-DFT vs sTD-DFT with **2Sc**, **2Y**, and **2La***

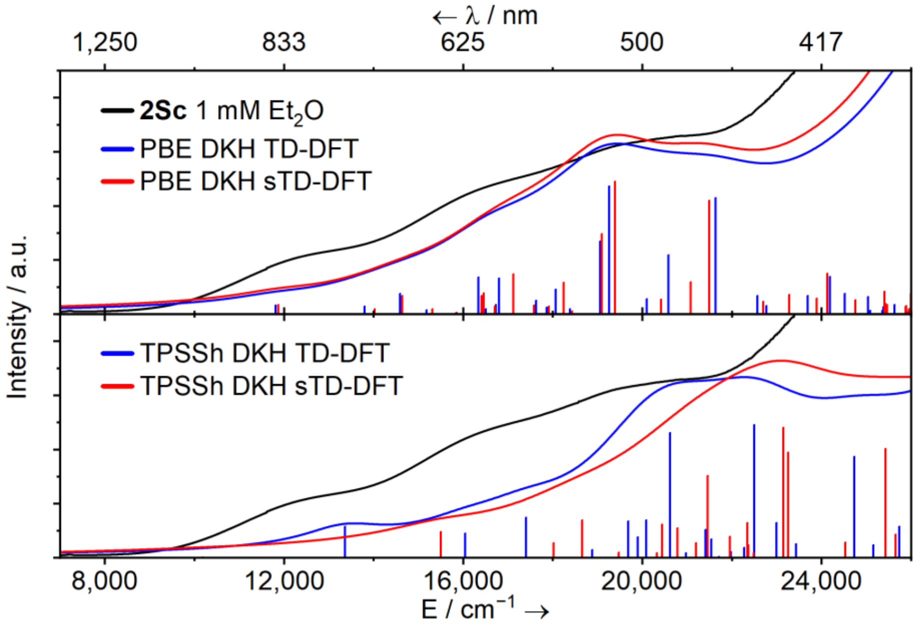

Figure S103. Experimental UV-Vis-NIR spectrum of [Sc(NHAr^{iPr6})$_2$] (**2Sc**, black line) compared to calculated transitions. Vertical lines correspond to transition electric dipole moments, red and blue curves correspond to the simulated spectra with Gaussian broadening and a linewidth factor of $25 \times \sqrt{E}$.

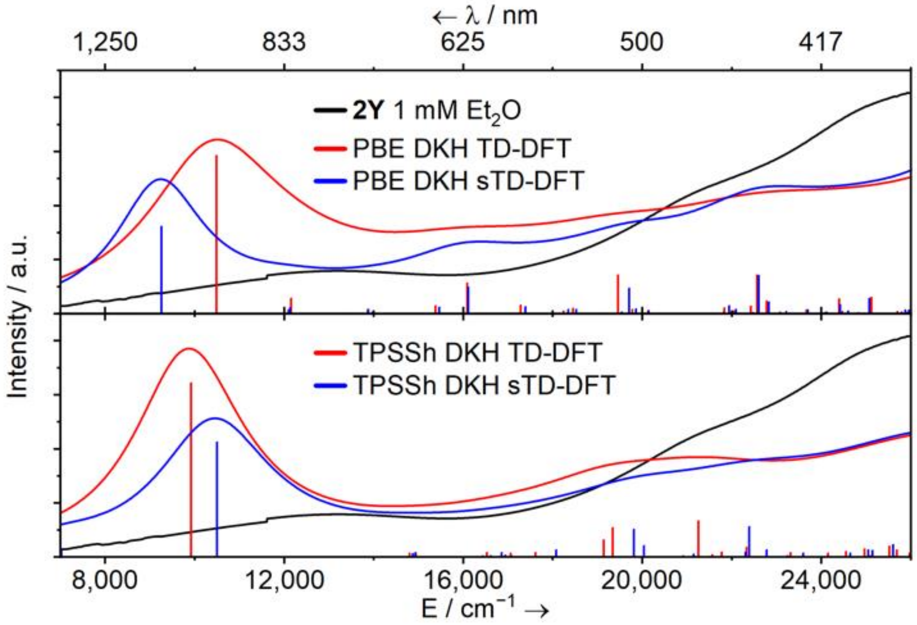

Figure S104. Experimental UV-Vis-NIR spectrum of [Y(NHAr^{iPr6})$_2$] (**2Y**, black line) compared to calculated transitions. Vertical lines correspond to transition electric dipole moments, red and blue curves correspond to the simulated spectra with Gaussian broadening and a linewidth factor of $25 \times \sqrt{E}$.

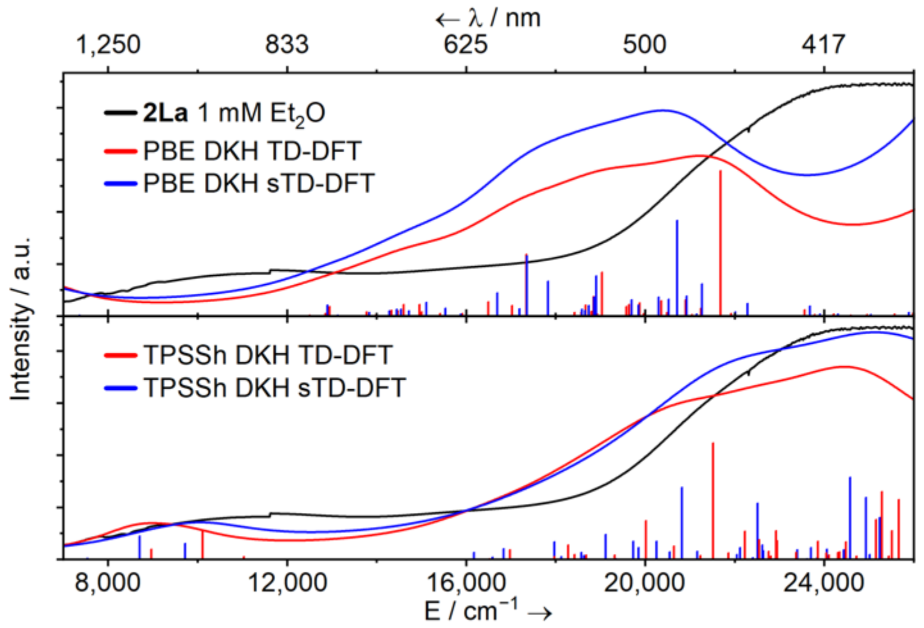

Figure S105. Experimental UV-Vis-NIR spectrum of [La(NHAr^{iPr6})$_2$] (**2La**, black line) compared to calculated transitions. Vertical lines correspond to transition electric dipole moments, red and blue curves correspond to the simulated spectra with Gaussian broadening and a linewidth factor of $25 \times \sqrt{E}$.

We can see that there is only a modest difference between the TD-DFT and sTD-DFT calculated spectra in most cases, with the exception of **2Sc** where functionals with Hartree-Fock exchange (TPSSh) underestimate the first excitation oscillator strength with the sTD-DFT formalism. Instead, a modest dependence upon functional can be seen, and so we have used the sTD-DFT method to screen a larger range of functionals due to its significantly lower computational cost.[62, 63] We also note that while some of the simulated spectra for **2Sc** and **2La** are qualitatively representative of the experimental data, **2Y** gave poor results in all cases – this will be more explicitly handled in a later section. We found no dependence upon relativistic treatment (DKH or ZORA formalisms) and so do not show these spectra below.

Next, we screened six functionals for all complexes (B3PW91,[44] CAM-B3LYP,[45] PBE,[46, 47] PBE0,[48, 49] TPSSh[34, 35], TPSS0[50]) and compared all results (**Figure S106**, **Figure S107**, and **Figure S108**).

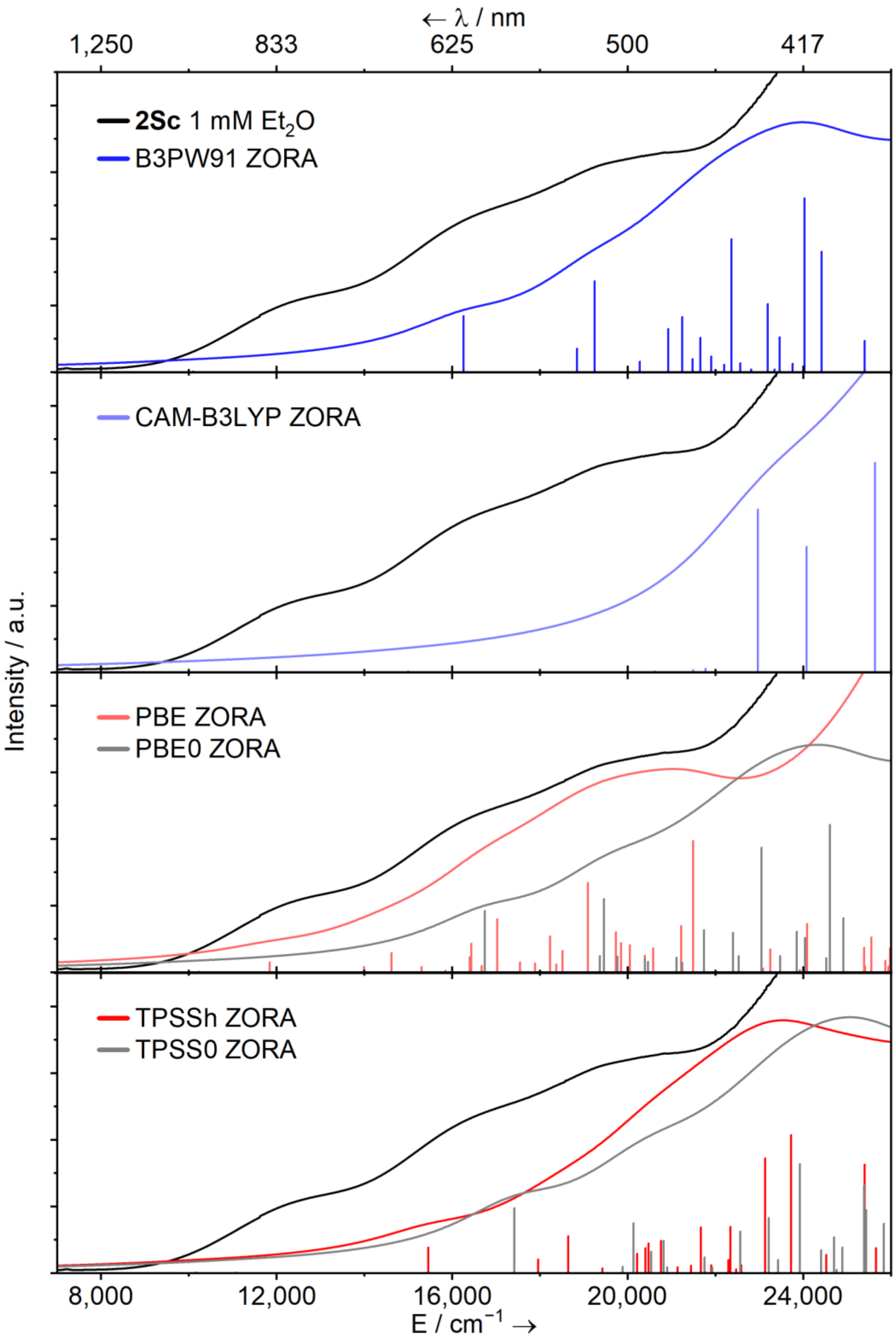

Figure S106. A comparison of the experimental UV-Vis-NIR spectrum of [Sc(NHAr^{iPr6})$_2$] (**2Sc**, black line) with sTD-DFT calculated transitions across all functionals studied herein, using the ZORA relativistic treatment (H, C, N – ZORA-def2-TZVP; Sc – ma-ZORA-def2-QZVPP) and the minimally optimised geometry as described above. Vertical lines correspond to transition electric dipole moments, curves correspond to the simulated spectra with Gaussian broadening and a linewidth factor of $25 \times \sqrt{E}$.

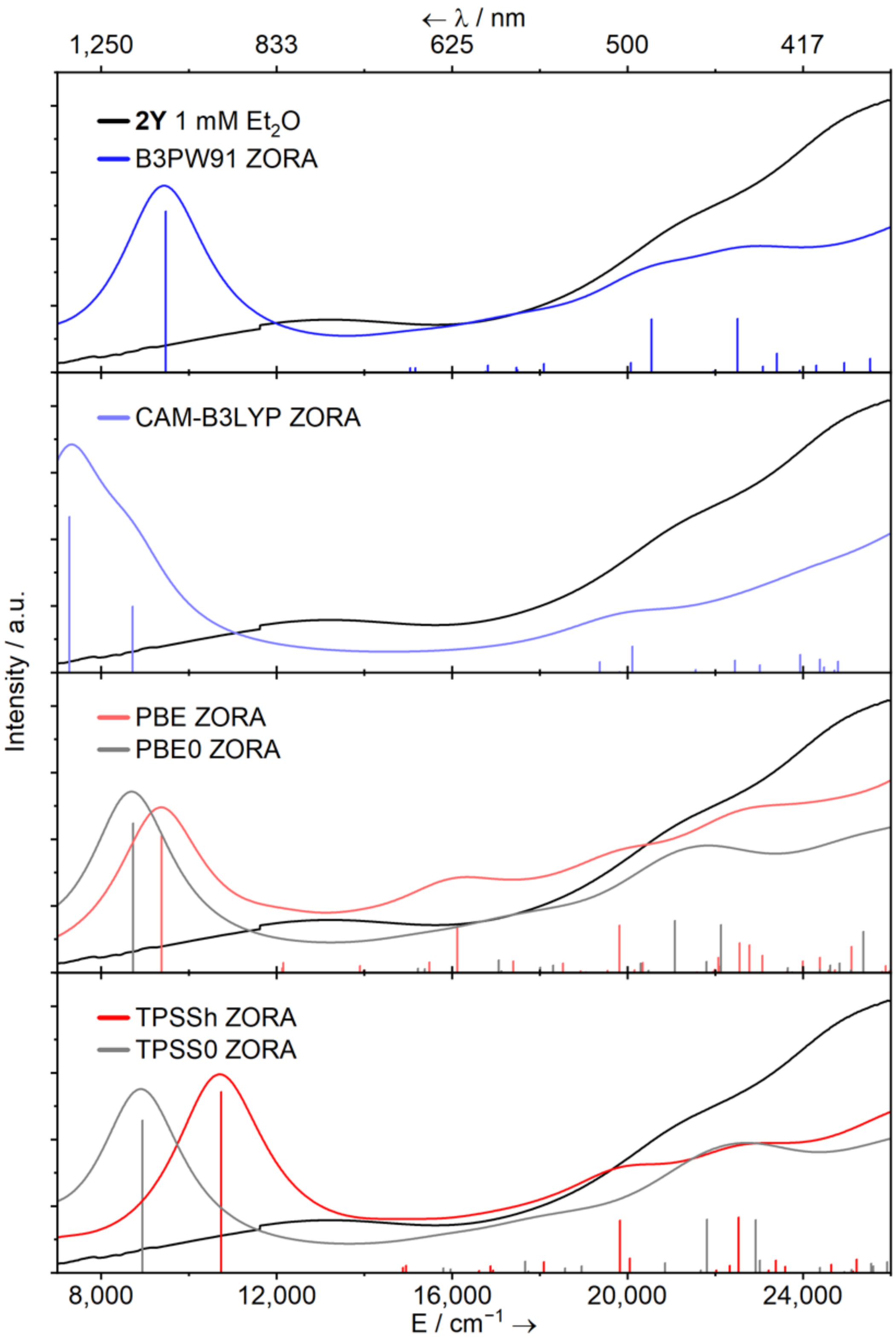

Figure S107. A comparison of the experimental UV-Vis-NIR spectrum of [Y(NHAr^{iPr6})$_2$] (**2Y**, black line) with sTD-DFT calculated transitions across all functionals studied herein, using the ZORA relativistic treatment (H, C, N – ZORA-def2-TZVP; Y – SARC-ZORA-TZVPP) and the minimally optimised geometry as described above. Vertical lines correspond to transition electric dipole moments, curves correspond to the simulated spectra with Gaussian broadening and a linewidth factor of $25 \times \sqrt{E}$.

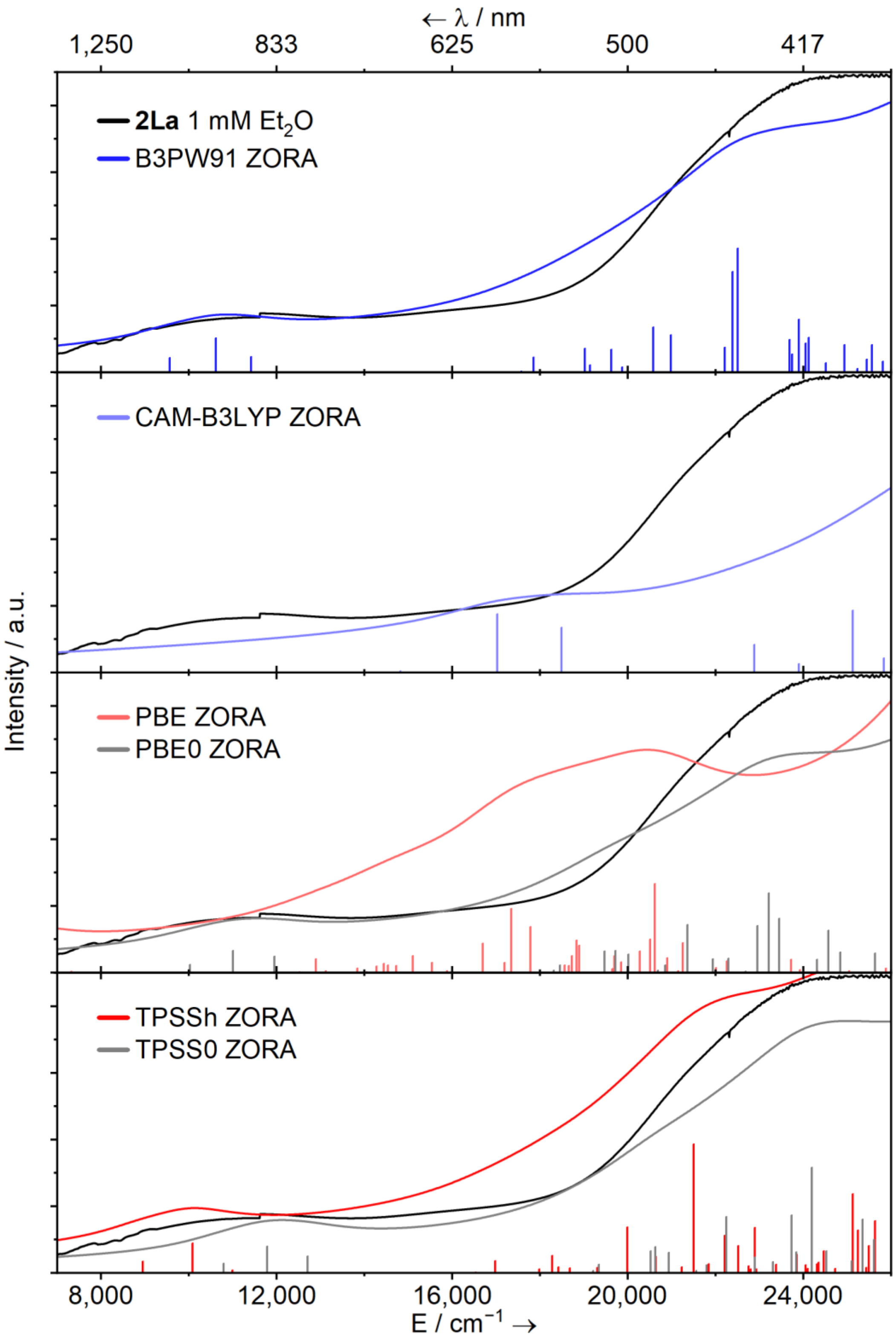

Figure S108. A comparison of the experimental UV-Vis-NIR spectrum of [La(NHAr^{iPr6})$_2$] (**2La**, black line) with sTD-DFT calculated transitions across all functionals studied herein, using the ZORA relativistic treatment (H, C, N – ZORA-def2-TZVP; La – SARC2-ZORA-QZVP) and the minimally optimised geometry as described above. Vertical lines correspond to transition electric dipole moments, curves correspond to the simulated spectra with Gaussian broadening and a linewidth factor of $25 \times \sqrt{E}$.

*Additional geometry optimisation of **2Y** to produce **2Y-Et$_2$O**, and TD-DFT screening*

The data above shows that **2Y** is an outlier, with all calculations severely overestimating the intensity and underestimating the energy of the lowest-lying transition. Inspection of the NTOs for this transition in **2Sc** and **2La** (*vide infra*) shows that it is a bound excitation of the SOMO {$d_{xy\ /\ x2-y2}$) + C_6 (E_g*)} to the LUMO which is also comprised of the same components – essentially it is a *d*→*d* transition with significant ligand character. With this in mind, the different interactions of the arene rings to the metal in **2Sc** and **2La** (one ring distorted) *vs* **2Y** (two rings distorted by half as much) shown by the X-ray diffraction data, contrasted with the significant similarities in their experimental UV-Vis-NIR spectra, suggested to us that the solid-state structure of **2Y** might not be representative of the structure in solution. Put simply, we suspected that the single-ring distortion of solid-state **2Sc** and **2La** may better represent the solution-state structure of **2Y**.

The full structure of complex **2Y** was optimised in both the gas phase and Et_2O solvent medium using the geometry of C_1-symmetric **2La** as a starting point. We assessed two functionals (PBE0 and TPSSh) for each phase. All four final geometries were verified to be true minima through harmonic vibrational analysis which showed no imaginary vibrational modes, and all retained a structure with only one Tripp group deformed and tightly bound to the metal. We then performed TD-DFT and sTD-DFT calculations on the Et_2O solution geometry (**2Y-Et$_2$O**) using the same methodology as above, and compared this to the experimental UV-Vis-NIR spectrum determined in this solvent. **Figure S109** shows the result of this.

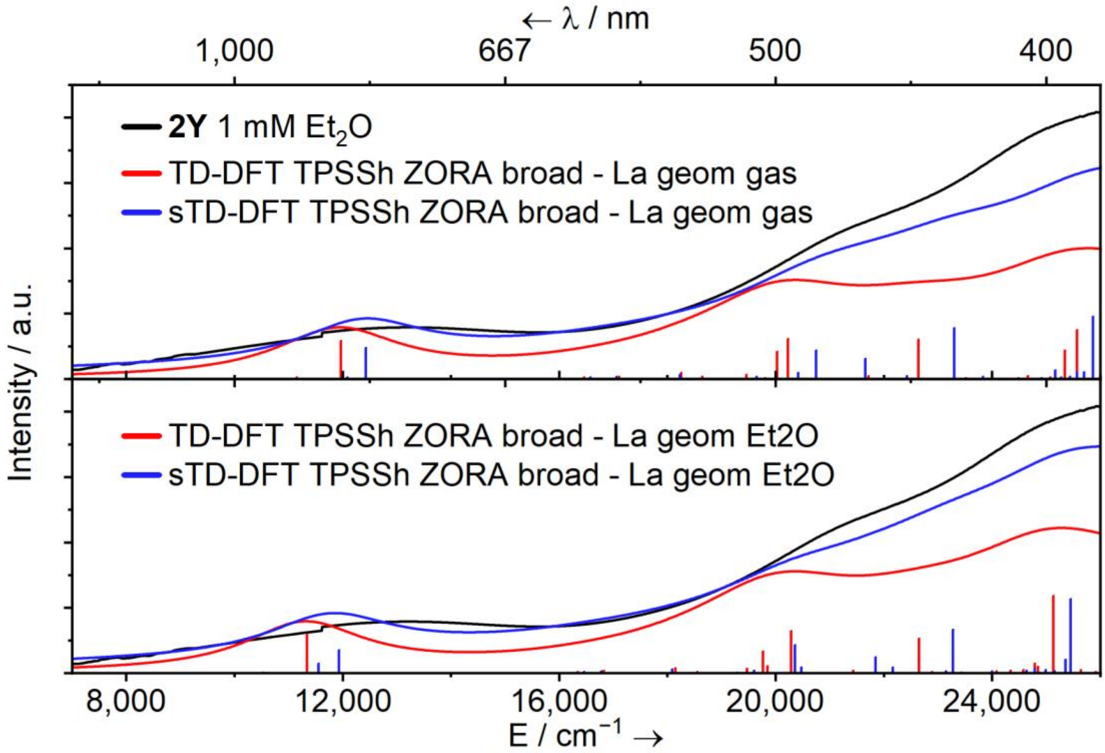

Figure S109. A comparison of the experimental UV-Vis-NIR spectrum of [Y(NHAr^{iPr6})$_2$] (**2Y**, black line) with TD-DFT and sTD-DFT calculated transitions using the TPSSh functional (H, C, N – ZORA-def2-TZVP; La – SARC2-ZORA-QZVP), and the fully optimised geometry of **2Y** in both the gas phase and solution phase (**2Y-Et$_2$O**) where **2La** was used as the starting point (*vide supra*). Vertical lines correspond to transition electric dipole moments, curves correspond to the simulated spectra with Gaussian broadening and a linewidth factor of $25 \times \sqrt{E}$.

*Natural transition orbitals for **2Sc**, **2Y-Et$_2$O**, and **2La***

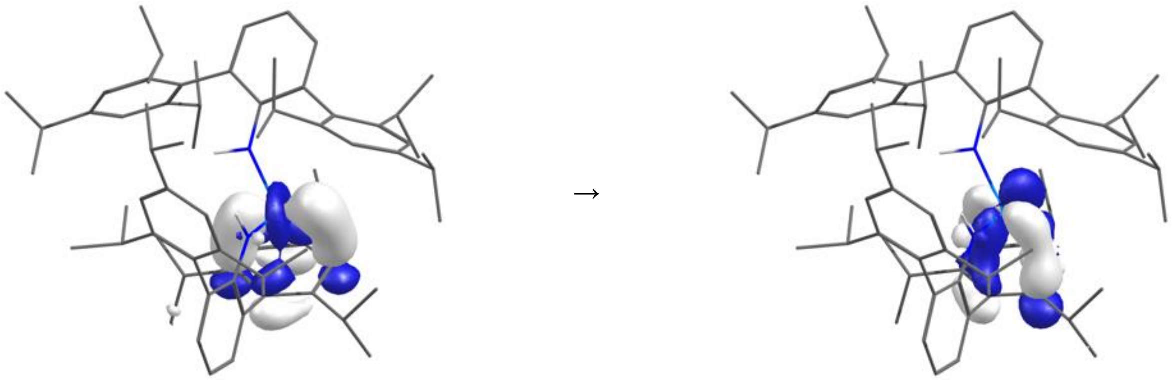

State 1 NTO 283 **State 1 NTO 284**

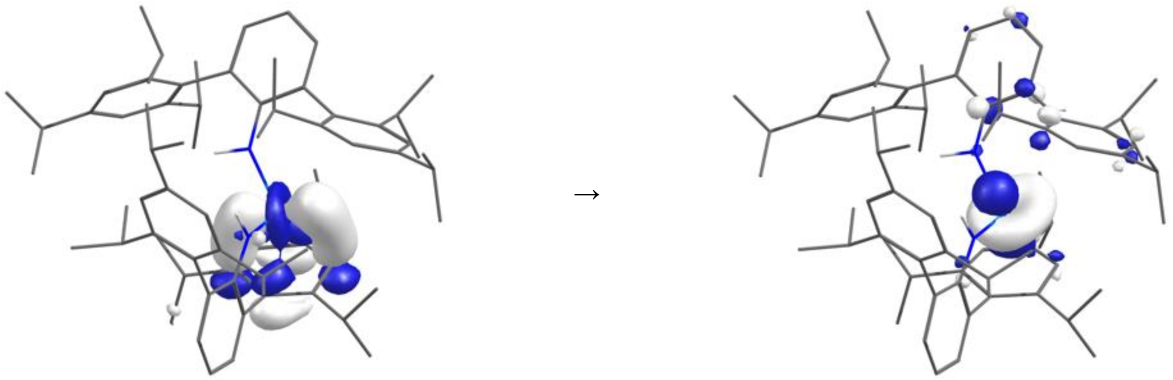

State 2 NTO 283 **State 2 NTO 284**

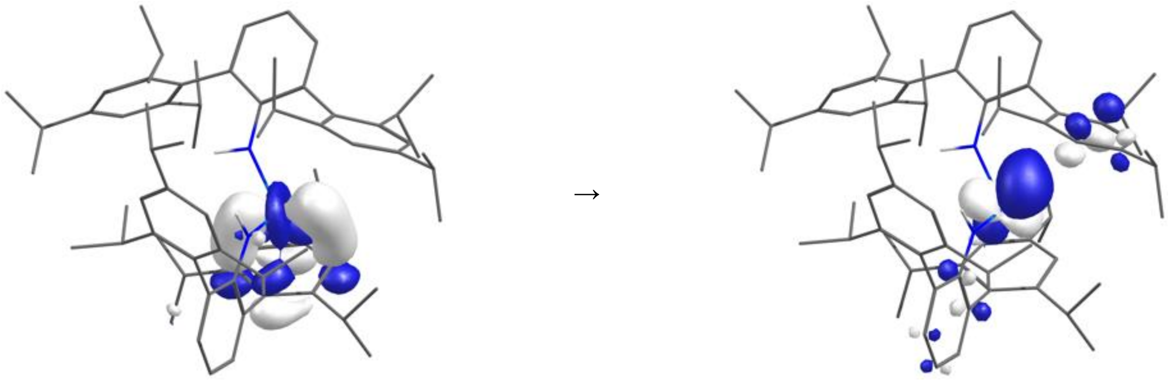

State 3 NTO 283 **State 3 NTO 284**

Figure S110. Natural transition orbitals (NTOs) for states 1, 2, and 3, for complex **2Sc** (isovalue = 0.05 a.u.).

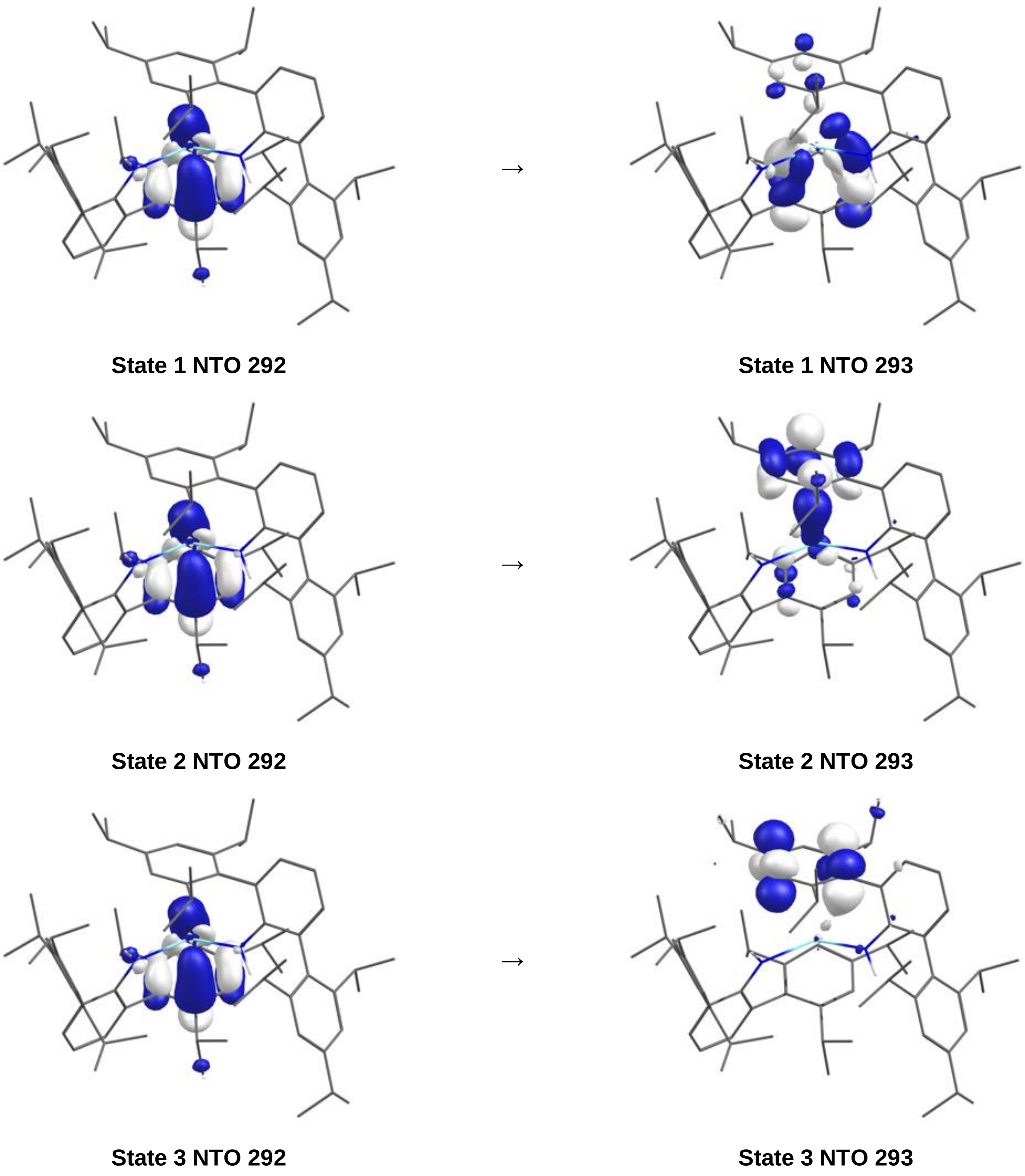

Figure S111. Natural transition orbitals (NTOs) for states 1, 2, and 3, for complex **2Y-Et$_2$O** (isovalue = 0.05 a.u.).

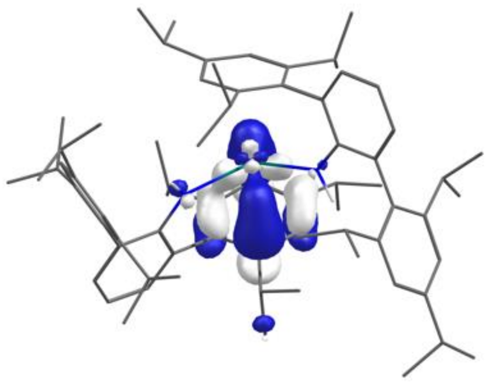

→

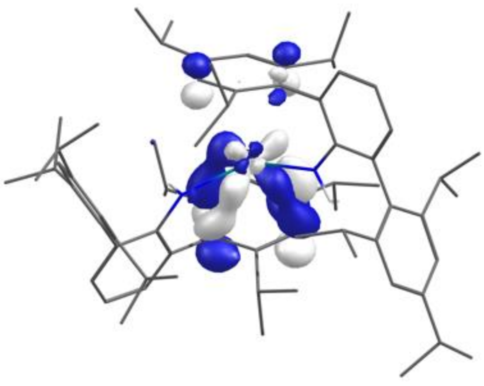

State 1 NTO 301 **State 1 NTO 302**

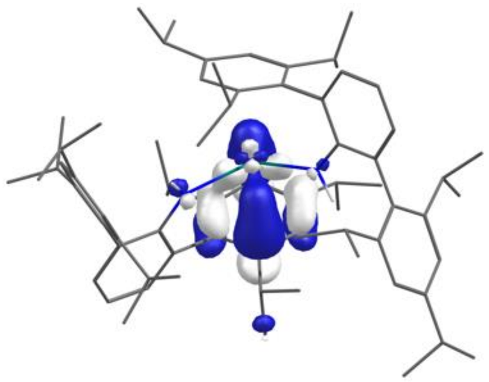

→

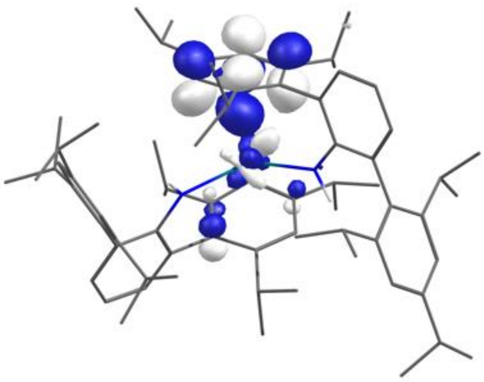

State 2 NTO 301 **State 2 NTO 302**

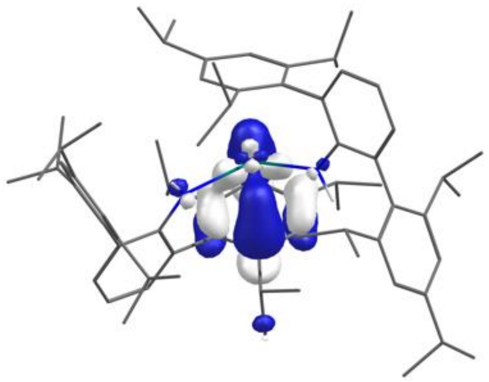

→

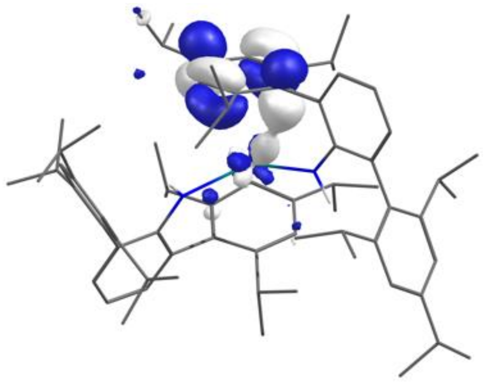

State 3 NTO 301 **State 3 NTO 302**

Figure S112. Natural transition orbitals (NTOs) for states 1, 2, and 3, for complex **2La** (isovalue = 0.05 a.u.).

Molecular orbital compositions and isosurfaces

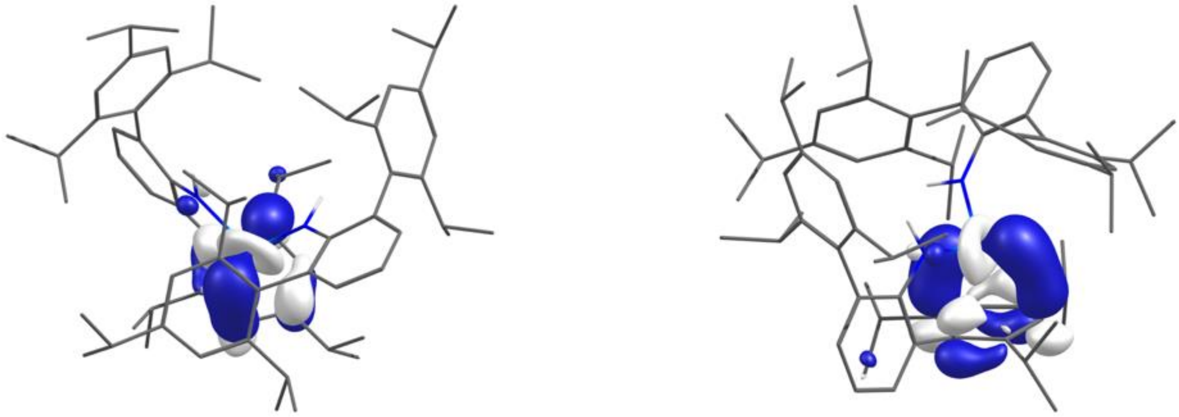

2Sc SOMO (α283)

Figure S113. Two views of the SOMO isosurface (0.05 a.u.) for **2Sc**.

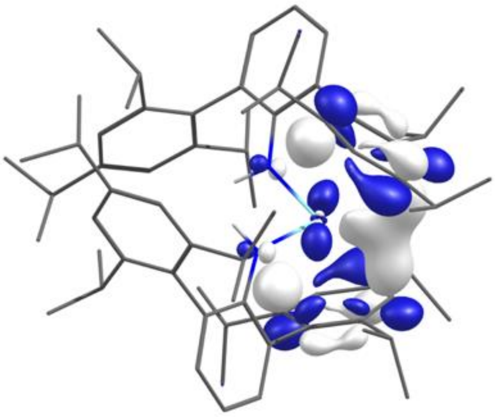

2Y SOMO (α292)

Figure S114. One view of the delocalised SOMO isosurface (0.05 a.u.) **2Y**.

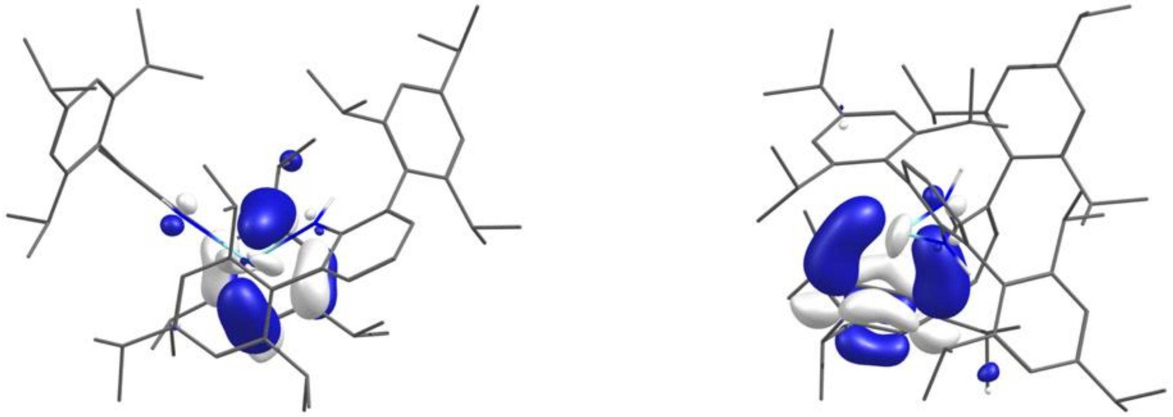

2Y-Et$_2$O SOMO (α292)

Figure S115. Two views of the SOMO isosurface (0.05 a.u.) for **2Y-Et$_2$O** – from the optimised (Et$_2$O solution) structure.

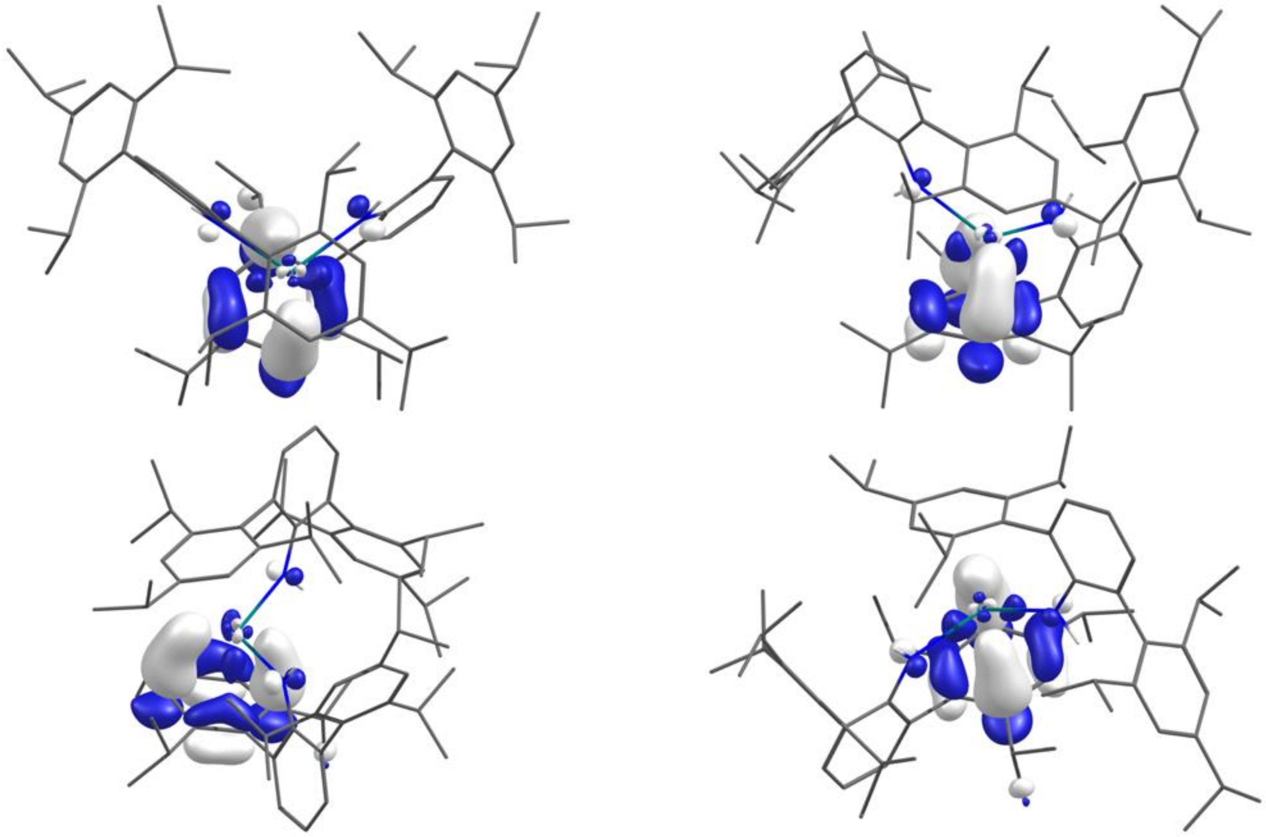

2La SOMO (α301)

Figure S116. Four views of the SOMO isosurface (0.05 a.u.) for **2La**.

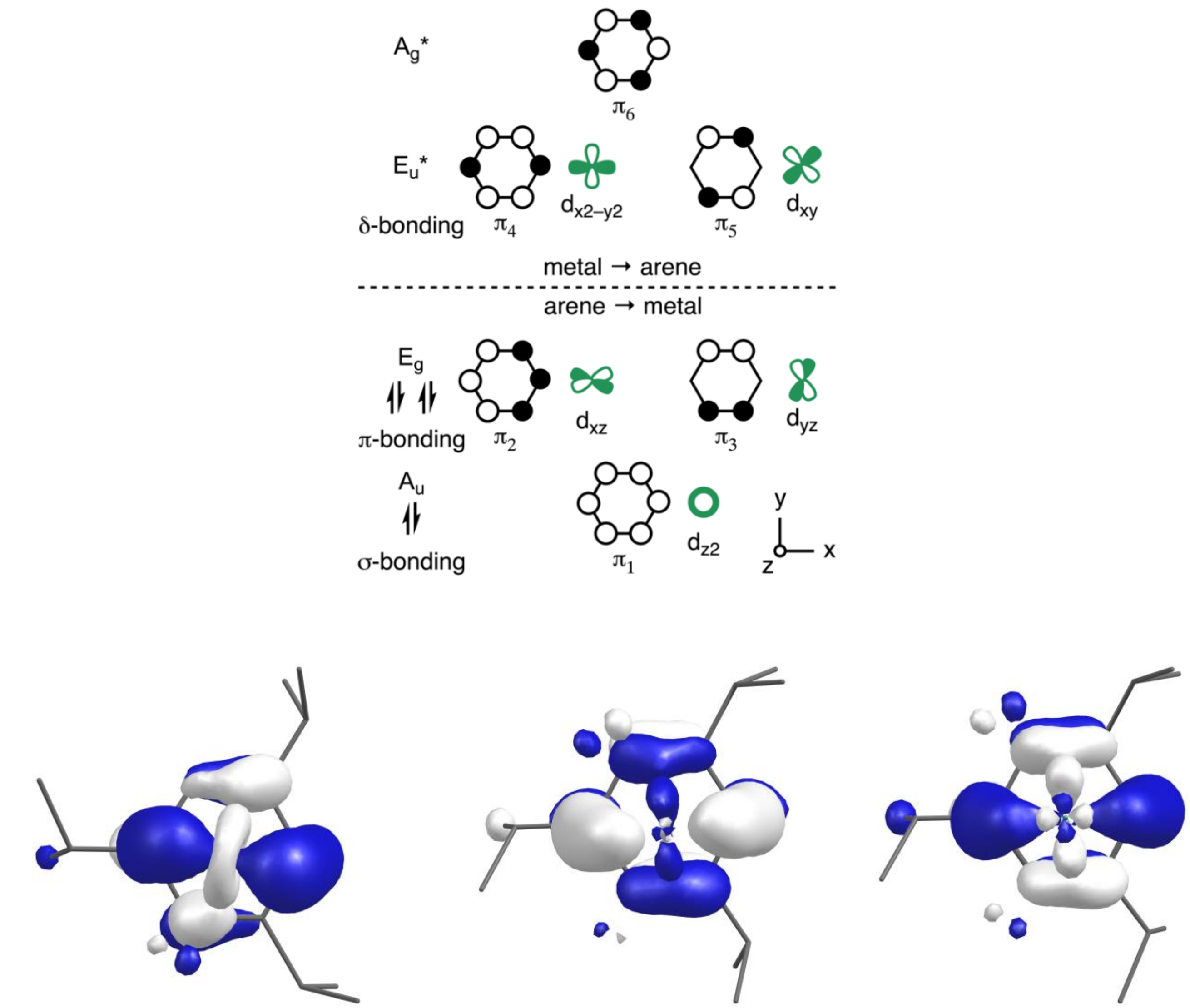

2Sc **2Y-Et$_2$O** **2La**

Figure 117. (Top) An illustration of metal-arene symmetry allowed orbital interactions for a d^0 metal, showing the divide between arene → metal, and metal→ arene donation; (Bottom) The SOMOs for **2Sc**, **2Y-Et$_2$O**, and **2La**, showing their similarity to a combination of the π_4 arene MO and a d_{x2-y2} atomic orbital (the majority of the molecular structures have been removed for clarity).

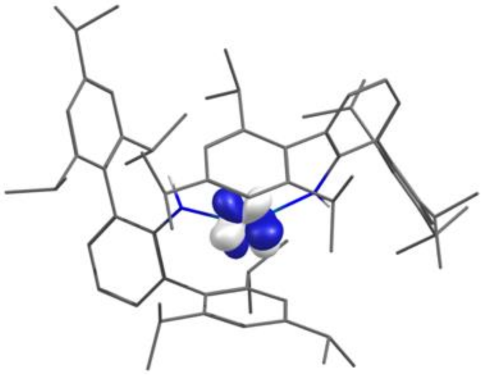

HOMO (α306)

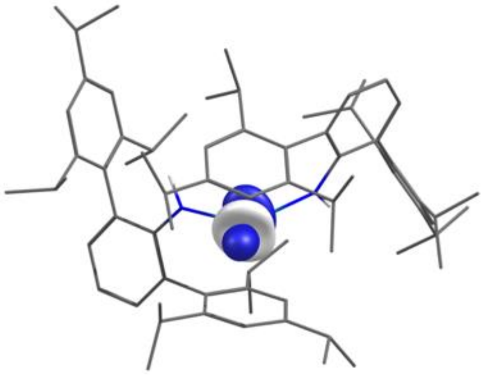

HOMO–1 (α305)

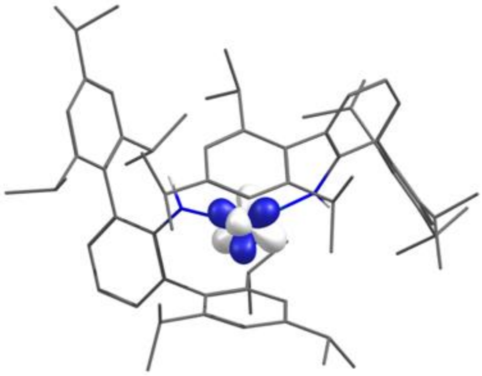

HOMO–2 (α304)

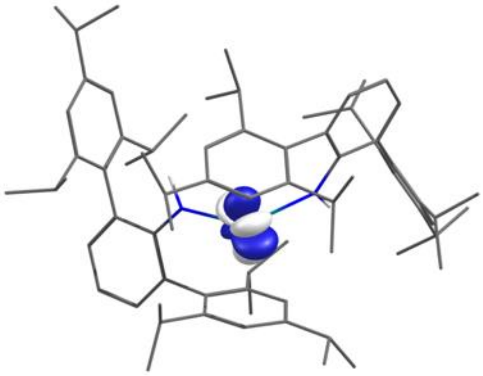

HOMO–3 (α303)

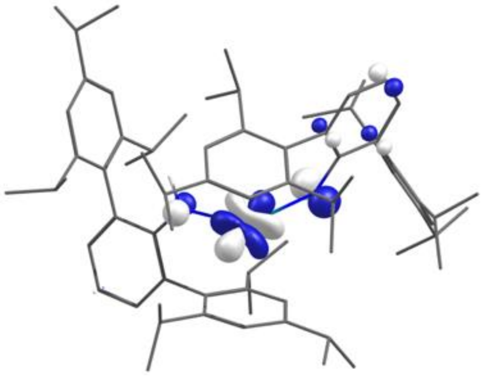

HOMO–4 (α302)

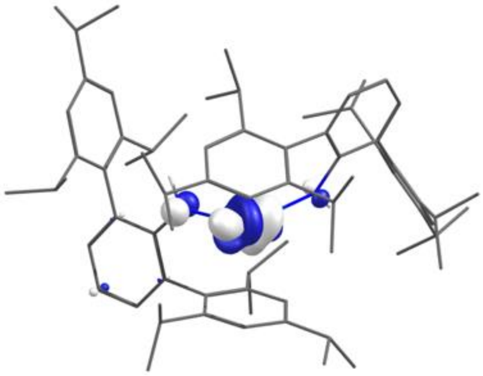

HOMO–5 (α301)

Figure S118. Views of the α-spin HOMO to HOMO–5 isosurfaces for **2Sm**.

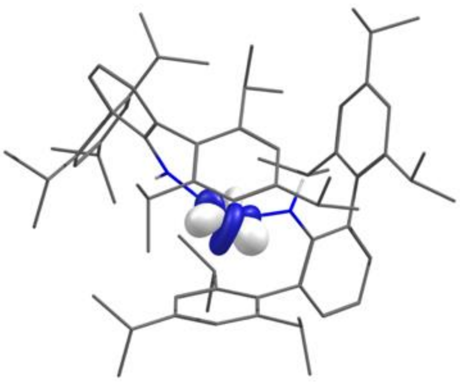

αHOMO (α307)

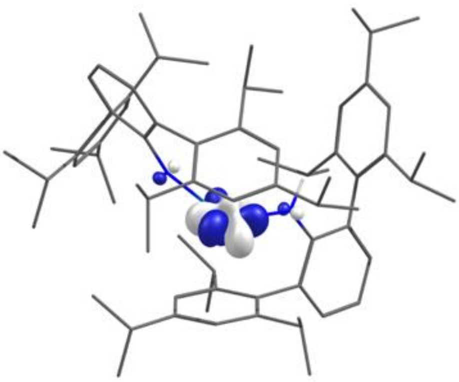

αHOMO–1 (α306)

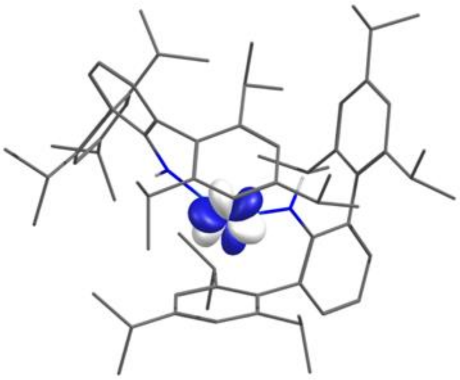

αHOMO–2 (α305)

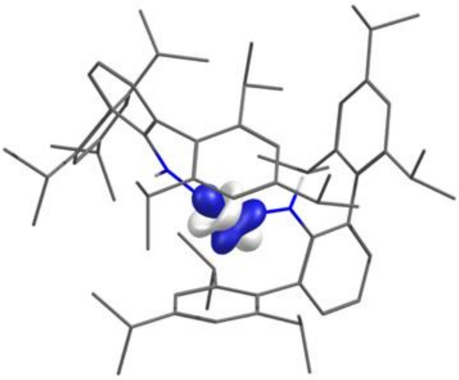

αHOMO–3 (α304)

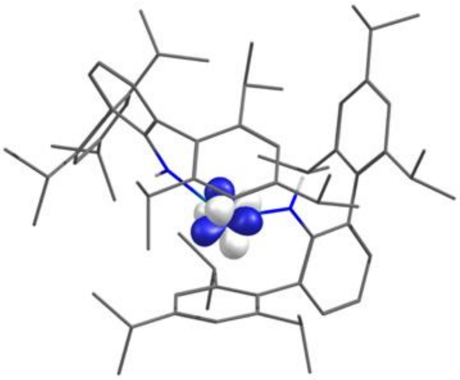

αHOMO–4 (α303)

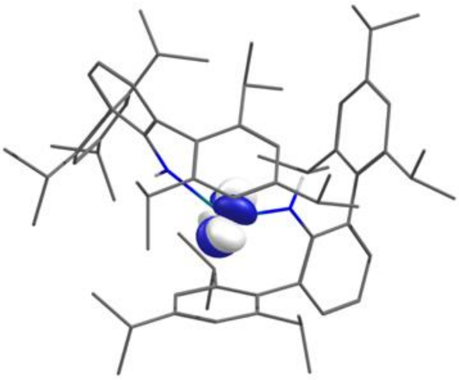

αHOMO–5 (α302)

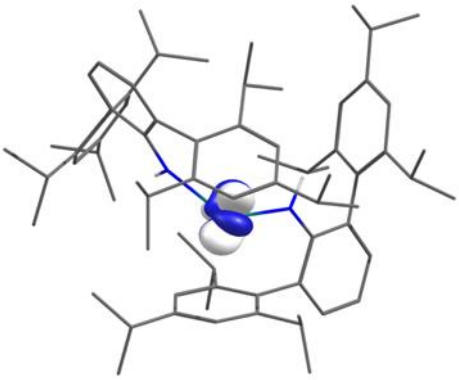

αHOMO–6 (α301)

Figure S119. Views of the α-spin HOMO to HOMO–6 isosurfaces (0.05 a.u.) for the Eu(1) molecule of **2Eu**.

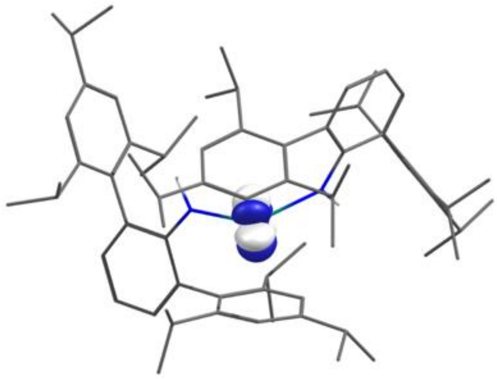

βHOMO (β306)

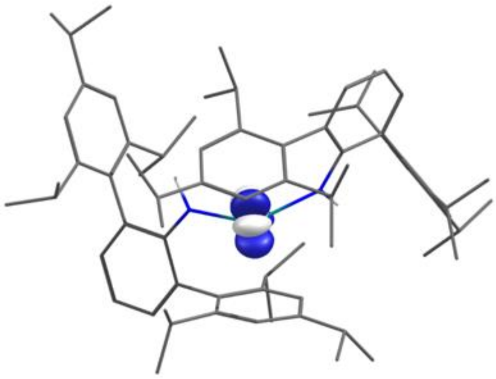

βHOMO–1 (β305)

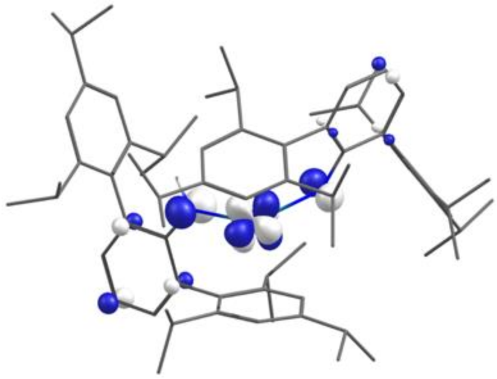

βHOMO–2 (β304)

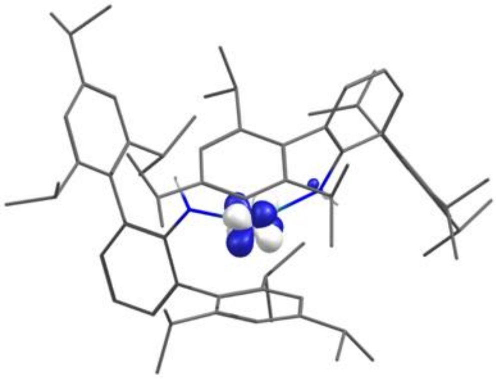

βHOMO–3 (β303)

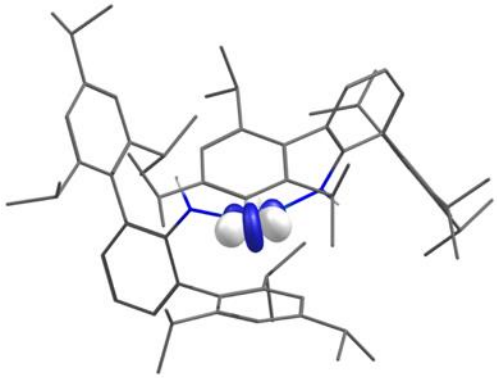

βHOMO–6 (α301)

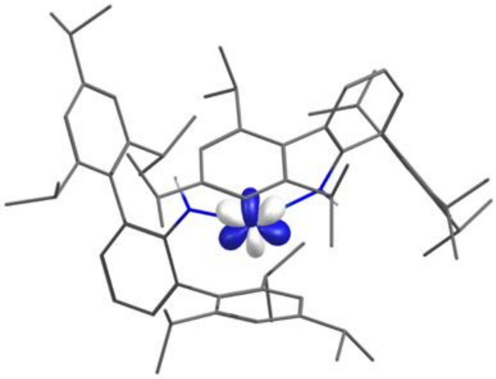

βHOMO–7 (α300)

Figure S120. Views of the β-spin HOMO to HOMO–3, and HOMO–6 to HOMO–7 isosurfaces (0.05 a.u.) of **2Tm**.

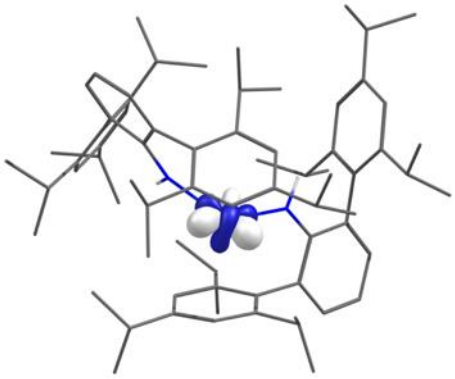

HOMO (MO307)

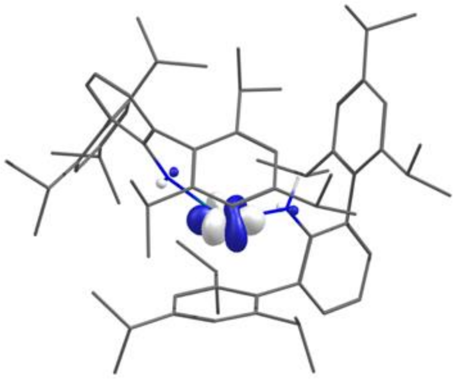

HOMO–1 (MO306)

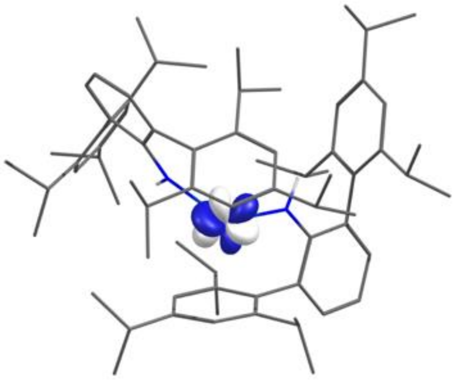

HOMO–2 (MO305)

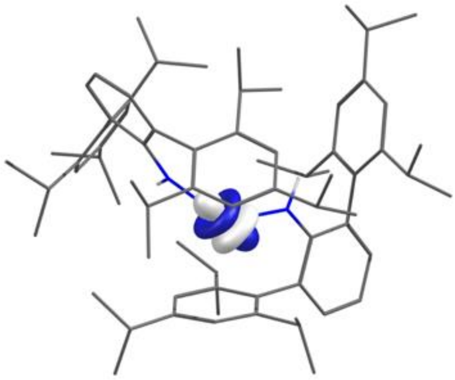

HOMO–3 (MO304)

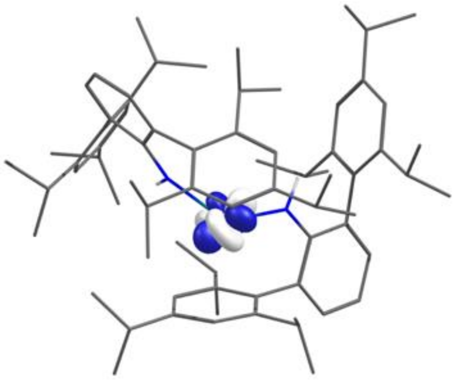

HOMO–4 (MO303)

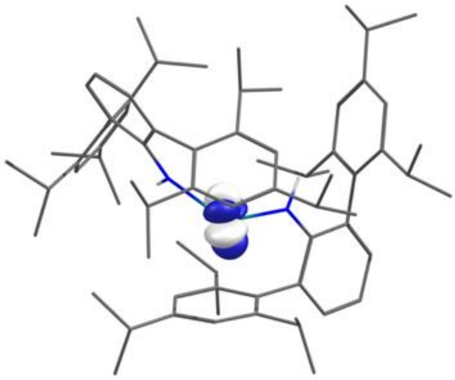

HOMO–5 (MO302)

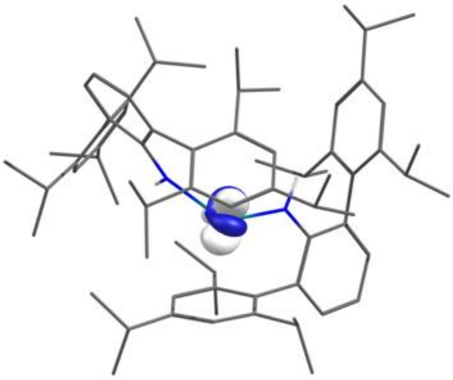

HOMO–6 (MO301)

Figure S121. Views of the HOMO to HOMO–6 isosurfaces (0.05 a.u.) of **2Yb**.

Table S22. Löwdin HOMO compositions and spin populations for **2Sc**, **2Y**, **2Y-Et$_2$O**, and **2La**, repeated for comparison to **2Sm**, **2Eu**, **2Tm**, and **2Yb**.

Complex	MO #	MO composition (%) [A]					Bound Tripp contribution (%) [B]	Metal spin population
		s	*p*	*d*	*f*	*g* [E]		
**2Sc** [C]	α283 – SOMO	3.6	0.9	35.6	1.9	0.4	41.0	0.459716
**2Y** [D]	α292 – SOMO	0.5	0.1	12.3	0.7	–	64.6	0.142622
**2Y-Et$_2$O** [D]	α292 – SOMO	1.8	0.3	22.4	0.8	–	55.7	0.245399
**2La** [E]	α301 – SOMO	1.0	0.2	14.2	9.6	0.0	56.0	0.265741
**2Sm** [E]	α301 – αHOMO–5	0.0	0.2	0.3	80.8	0.0	N/A	5.897171 Theoretical Sm(II) 4f^6 = 6.00
	α302 – αHOMO–4	0.0	0.2	0.3	62.9	0.0		
	α303 – αHOMO–3	0.0	0.0	0.0	97.8	0.0		
	α304 – αHOMO–2	0.0	0.0	0.3	94.5	0.0		
	α305 – αHOMO–1	0.0	0.3	1.4	92.9	0.0		
	α306 – αHOMO	0.0	0	2.4	90.7	0.0		
**2Eu** – Eu(1) [D, G]	α301 – αHOMO–6	0.0	0.0	0.1	98.7	0.0	N/A	6.975674 Theoretical Eu(II) 4f^7 = 7.00
	α302 – αHOMO–5	0.0	0.0	0.7	97.2	0.0		
	α303 – αHOMO–4	0.0	0.0	0.3	96.2	0.0		
	α304 – αHOMO–3	0.0	0.0	0.0	97.5	0.0		
	α305 – αHOMO–2	0.0	0.0	1.3	95.4	0.0		
	α306 – αHOMO–1	0.0	0.0	0.6	89.1	0.0		
	α307 – αHOMO	0.7	0.1	1.1	95.3	0.0		
**2Tm** [E]	β299 – βHOMO–7	0.0	0.0	0.0	98.6	0.0	N/A	1.146473 Theoretical (low-spin) Tm(II) 4f^{13} = 1.00
	β300 – βHOMO–6	0.3	0.0	0.9	94.0	0.0		
	β303 – βHOMO–3	0.0	0.0	0.0	95.9	0.0		
	β304 – βHOMO–2	0.0	0.0	0.0	98.2	0.0		
	β305 – βHOMO–1	0.2	0.1	1.1	92.7	0.0		
	β306 – βHOMO	0.0	0.1	1.8	89.1	0.0		
**2Yb** – Yb(1) [E, G]	β301 – βHOMO–6	0.0	0.0	0.0	99.0	0.0	N/A	N / A
	β302 – βHOMO–5	0.0	0.0	0.0	98.2	0.0		
	β303 – βHOMO–4	0.0	0.0	0.4	97.4	0.0		
	β304 – βHOMO–3	0.0	0.0	0.1	97.7	0.0		
	β305 – βHOMO–2	0.0	0.0	0.7	96.8	0.0		
	β306 – βHOMO–1	0.0	0.0	0.7	97.1	0.0		
	β307 – βHOMO	0.0	0.0	0.3	93.0	0.0		

[A] The basis set for each metal is listed separately (table footnotes B to D)[51-53, 55], H-, C-, and N-atoms treated with ZORA-def2-TZVP, and using the TPSSh functional; [B] The delocalised nature of Kohn-Sham orbitals means that the sum of the metal and bound-arene contributions does not sum to 100% and is not unexpected; [C] Sc basis set – ma-ZORA-def2-QZVPP; [D] Y-basis set – SARC-ZORA-TZVPP; [E] Ln-basis set (Ln = La, Sm, Eu, Tm, Yb) – SARC2-ZORA-QZVP; [F] Note that this is a polarization function in some of the basis sets used, but not all; [G] Only M(1) details are presented here as the crystallographically-distinct second molecule is essentially the same.

*Calculated EPR coupling constants and g-matrices for **2Sc**, **2Y**, **2Y-Et$_2$O**, and **2La***

Table S23. Calculated and experimental (frozen solution) EPR hyperfine coupling constants and *g*-matrices for [Sc(NHAr^{iPr6})$_2$] (**2Sc**).

ma-ZORA-def2-QZVPP	TPSSh	PBE	PBE0
A_{xx}	159.3730	175.2593	168.8108
A_{yy}	170.7893	186.0246	181.3446
A_{zz}	184.2000	203.7161	192.0219
A_{iso} / MHz	*171.4541*	*188.3333*	*180.7257*
**Experimental (MHz)**	A_1 = 195; A_2 = A_3 = 210; A_{iso} = 205		

	TPSSh	PBE	PBE0
g_{xx}	1.9904566	1.9889051	1.9894049
g_{yy}	2.0089290	2.0075239	2.0080231
g_{zz}	2.0146241	2.0137694	2.0137898
***g*-iso**	*2.0046699*	*2.0033995*	*2.0037393*
**Experimental (*g*)**	g_1 = 1.990; g_2 = g_3 = 2.002; g_{iso} = 1.998		

Table S24. Calculated EPR hyperfine coupling constants and *g*-matrices for [Y(NHAr^{iPr6})$_2$] (**2Y**).

SARC-ZORA-TZVPP	TPSSh	PBE	PBE0
A_{xx}	-10.5402	-13.2339	-11.6499
A_{yy}	-11.0378	-13.7995	-12.1158
A_{zz}	-14.7258	-17.1084	-15.8637
A_{iso} / MHz	*-12.1013*	*-14.7139*	*-13.2098*
**Experimental (MHz)**	40.2 [16] / A_1 = 36; A_2 = A_3 = 39; A_{iso} = 38		

	TPSSh	PBE	PBE0
g_{xx}	1.9832852	1.9840625	1.9836125
g_{yy}	1.9988012	1.9962388	1.9982958
g_{zz}	2.0049105	2.0035978	2.0055640
***g*-iso**	*1.9956656*	*1.9946331*	*1.9958241*
**Experimental (*g*)**	g_1 = 1.986; g_2 = g_3 = 2.004; g_{iso} = 1.998		

Table S25. Calculated EPR hyperfine coupling constants and *g*-matrices for the optimised structure of [Y(NHAr^{iPr6})$_2$] in Et_2o solution (**2Y-Et₂O**).

SARC-ZORA-TZVPP	TPSSh	PBE	PBE0
A_{xx}	-47.3321	-53.2726	-48.5672
A_{yy}	-48.9112	-55.6323	-49.4351
A_{zz}	-50.1662	-56.1595	-51.2763
A_{iso} / MHz	*-48.8032*	*-55.0214*	*-49.7595*
**Experimental (MHz)**	40.2 [16] / A_1 = 36; A_2 = A_3 = 39; A_{iso} = 38 *		

	TPSSh	PBE	PBE0
g_{xx}	1.9829600	1.9804402	1.9844721
g_{yy}	2.0037632	2.0041021	2.0033283
g_{zz}	2.0059047	2.0057958	2.0051026
***g*-iso**	*1.9975426*	*1.9967794*	*1.9976343*
**Experimental (*g*)**	g_1 = 1.986; g_2 = g_3 = 2.004; g_{iso} = 1.998 *		

* For comparison only.

Table S26. Calculated EPR hyperfine coupling constants and *g*-matrices for [La(NHAr^{iPr6})$_2$] (**2La**).

SARC2-ZORA-QZVP	TPSSh	PBE	PBE0
A_{xx}	101.3636	112.0555	111.0317
A_{yy}	106.0236	116.6250	113.5064
A_{zz}	108.3126	118.4198	119.1325
A_{iso} / MHz	*105.2333*	*115.7001*	*114.5569*
**Experimental (MHz)**	A_1 = 100; A_2 = A_3 = 110; A_{iso} = 107		

	TPSSh	PBE	PBE0
g_{xx}	1.9544639	1.9334955	1.9597755
g_{yy}	1.9714902	1.9660807	1.9754029
g_{zz}	1.9933645	1.9786102	1.9976386
***g*-iso**	*1.9731062*	*1.9593955*	*1.9776057*
**Experimental (*g*)**	g_1 = 1.952; g_2 = g_3 = 2.005; g_{iso} = 1.987		

S10. CASSCF calculations

Methodology for complete active space self-consistent field calculations

CASSCF calculations were performed with OpenMolcas v21.06.[64] (Partially) DFT-optimized structures were used in all cases: H-atoms optimized for **2Sc**, **2Y**, **2Sm**, **2Eu** (1st structure in asymmetric unit), **2Tm** and **2Yb** (1st structure in asymmetric unit); while for **2La** the six C-atoms of the distorted Tripp arene ring along with all atoms of the 4-*i*Pr group and all H-atoms in the structure were optimized; and **2Y-Et$_2$O** the whole structure was optimized as described. Basis sets from the ANO-RCC library were employed,[65-68] with VTZP quality for the metal atom, VDZP quality for the 12 arene ring carbon atoms and the nitrogen atoms, while VDZ quality was employed for all other atoms. Cholesky decomposition of the two-electron integrals to a threshold of 10^{-8} was performed, along with employing the 2nd order Douglass-Kroll-Hess transformation to account for scalar relativistic effects.[69] MC-PDFT corrections were made in some cases.[70, 71] For **2Sc**, **2Y**, **2Y-Et$_2$O** and **2La** the active space was one electron in the four lowest-lying metal-based orbitals (corresponding to the excitations discussed in the text), while for **2Sm**, **2Eu** and **2Tm** the active space was six, seven and thirteen electrons, respectively, in seven 4f orbitals. We performed state average CASSCF calculations for four doublet roots for **2Sc**, **2Y**, **2Y-Et$_2$O** and **2La**; the weight of the dominant configuration in the average orbital active space in each case was 66%, 63%, 73% and 71% respectively, and the natural occupation numbers for the four state-specific natural orbitals was 1.00 in all cases. In the case of state average CASSCF calculations for **2Sm** we considered seven septets, for **2Eu** we considered one octet and 48 sextets, and for **2Tm** we considered seven doublets. For **2Yb** we started with a restricted Hartree-Fock calculation and performed RAS-probing calculations for four doublet roots, including frontier orbitals (11 orbitals in Ras1 and 5 orbitals in Ras3) and single excitations, finding a $4f^{14}$ ground state. No indication of low-lying nd^1 or ligand radical configurations arose in any of **2Sm**, **2Eu**, **2Tm** or **2Yb**. Note that the NTOs for **2Sc**, **2Y-Et$_2$O**, and **2La** from TD-DFT calculations (**Figure S110** to **Figure S112**) show excellent qualitative agreement with the excited state natural orbitals from CASSCF calculations below.

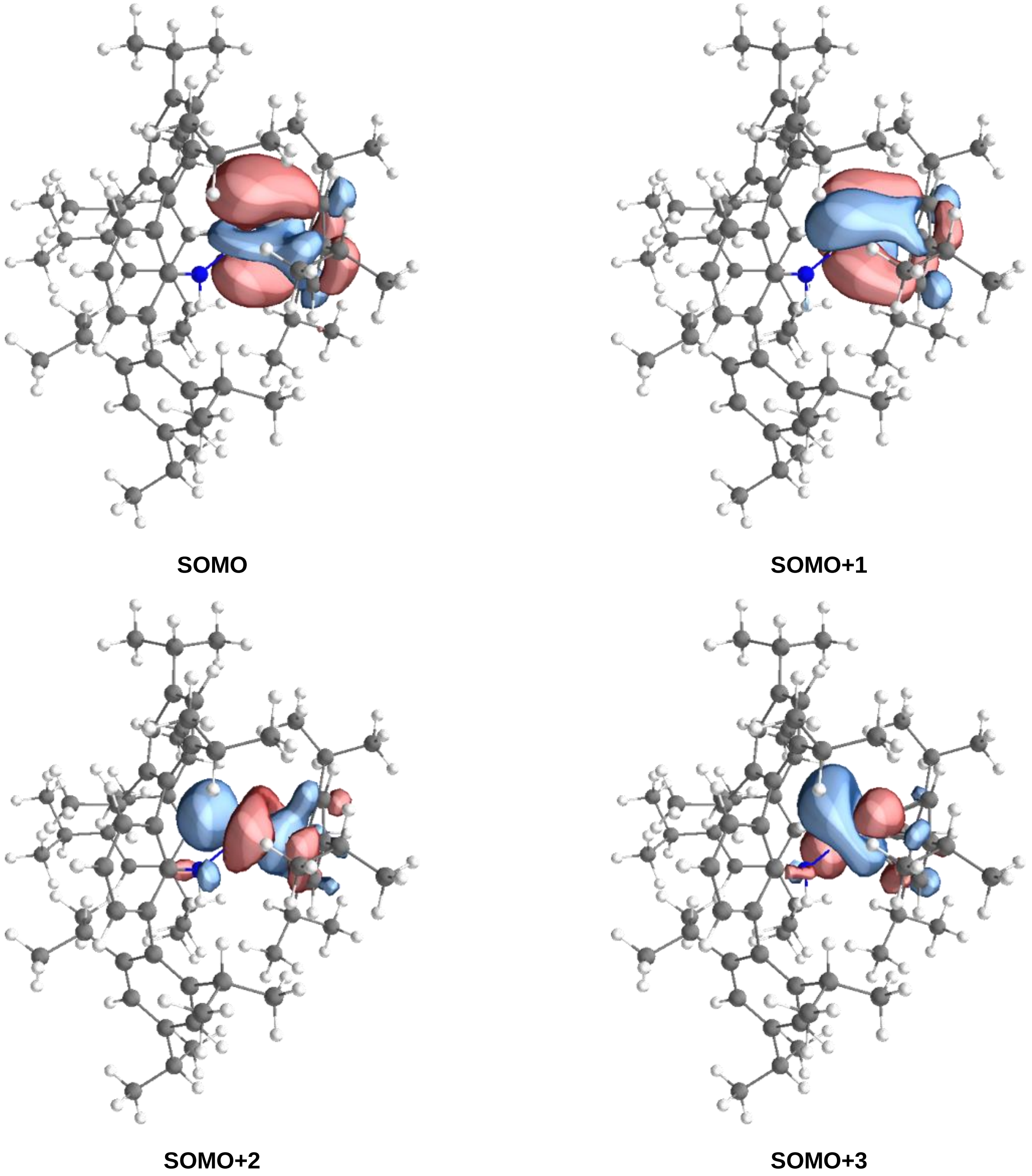

Figure S122. State-specific natural orbitals corresponding to SOMO, SOMO+1, SOMO+2, and SOMO+3 isosurfaces (0.03 a.u.) for **2Sc** from CASSCF calculations. All have unit occupation.

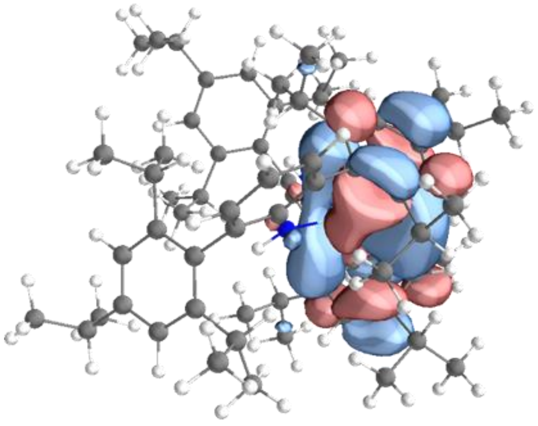

SOMO

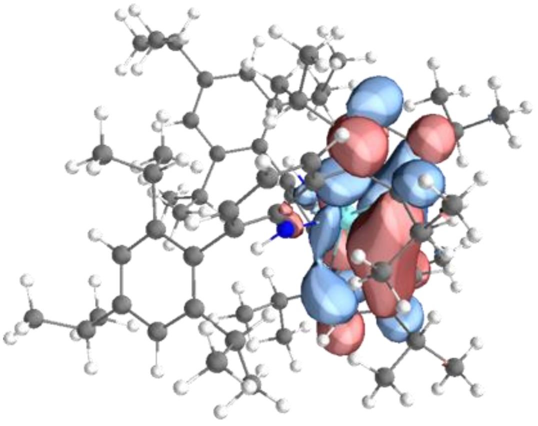

SOMO+1

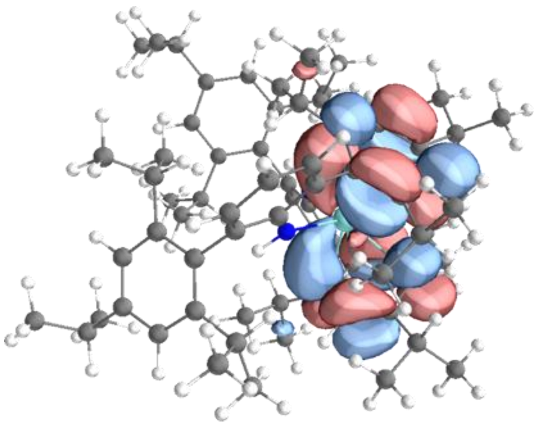

SOMO+2

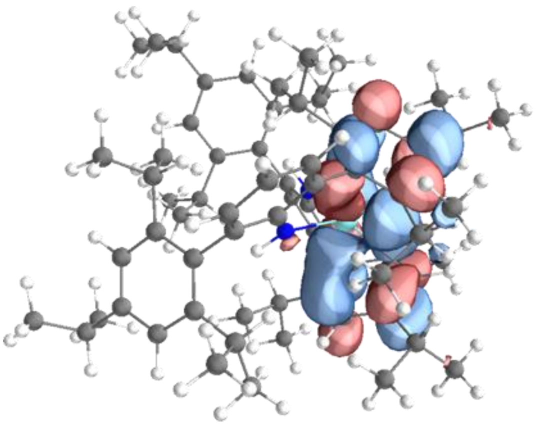

SOMO+3

Figure S123. State-specific natural orbitals corresponding to SOMO, SOMO+1, SOMO+2, and SOMO+3 isosurfaces (0.03 a.u.) for **2Y** from CASSCF calculations. All have unit occupation.

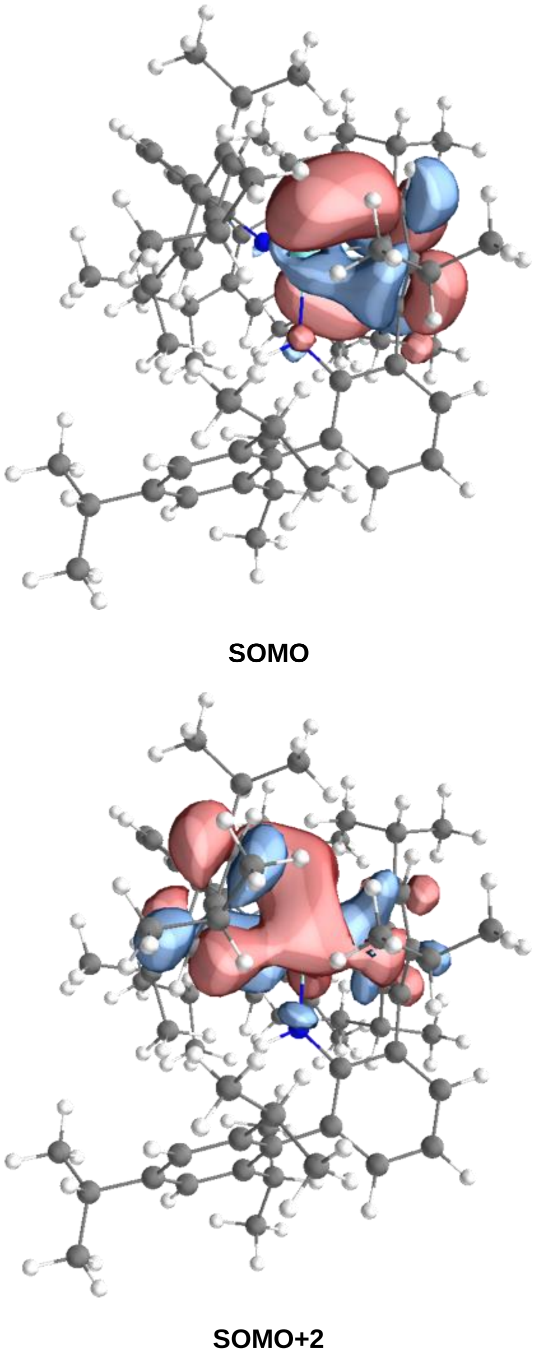

Figure S124. State-specific natural orbitals corresponding to SOMO, SOMO+1, SOMO+2, and SOMO+3 isosurfaces (0.03 a.u.) for **2Y-Et$_2$O** from CASSCF calculations. All have unit occupation.

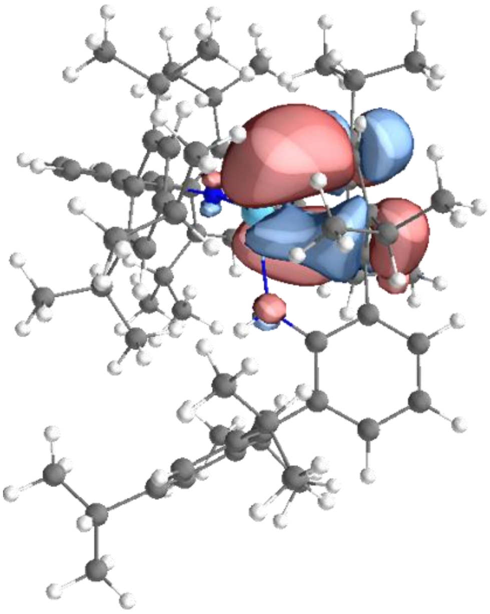

SOMO

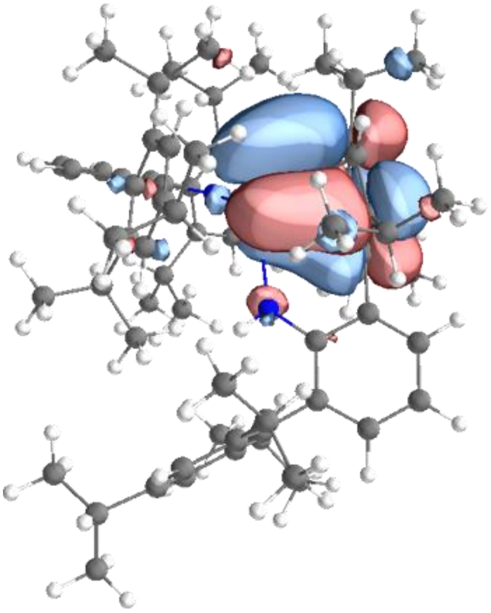

SOMO+1

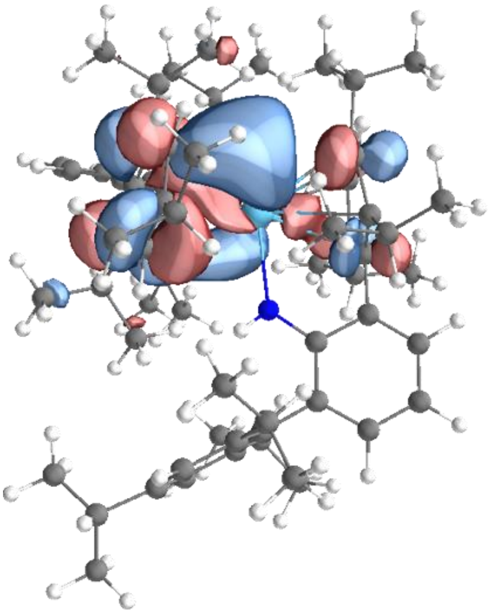

SOMO+2

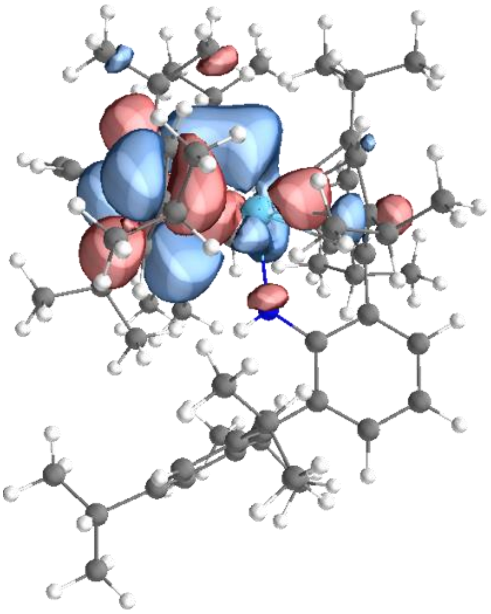

SOMO+3

Figure S125. State-specific natural orbitals corresponding to SOMO, SOMO+1, SOMO+2, and SOMO+3 isosurfaces (0.03 a.u.) for **2La** from CASSCF calculations. All have unit occupation.

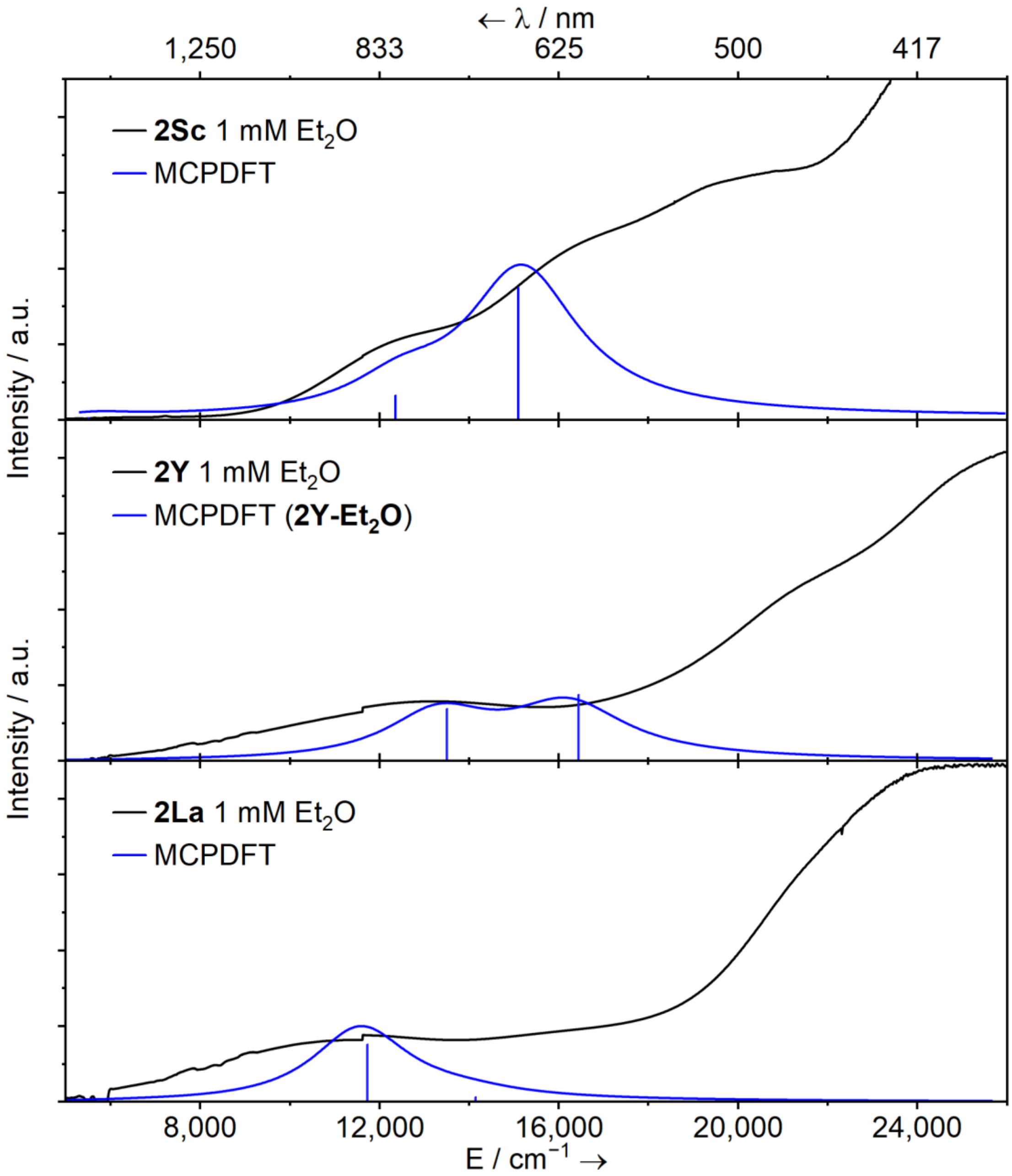

Figure S126. Experimental (1 mM, Et_2O, black), calculated multi-configurational pair-density functional theory (MC-PDFT) transitions (blue vertical lines), and simulated (blue solid lines, with Gaussian broadening and a linewidth factor of $25 \times \sqrt{E}$) UV-Vis-NIR spectra for complexes **2Sc**, **2Y-Et₂O** and **2La**.

S11. Calculated coordinates

Constrained optimisations

2Sc

Sc	0.002910138054	-0.000913512230	-0.002289259626
N	-0.378184841804	1.979922661985	0.531800461156
H	-0.598048242675	2.218910415287	1.498234069532
N	-1.062836053731	-1.180716557315	1.321572459814
H	-2.067412358583	-1.070433416807	1.160558333719
C	-0.630740944350	3.052922154807	-0.306486550829
C	-0.511189651378	2.820003620826	-1.700772721043
C	-0.665817839104	3.857331646262	-2.612183140220
H	-0.547029780469	3.653727697709	-3.676049577239
C	-0.982598967431	5.139121701127	-2.171897520798
H	-1.104568531152	5.953055379803	-2.883247137922
C	-1.168264302287	5.361361425486	-0.809491517678
H	-1.454117897132	6.351363830724	-0.455227702745
C	-1.014752020678	4.342994388126	0.131607682536
C	-0.242355710187	1.396481576835	-2.046945182699
C	-1.349194309660	0.448518827463	-1.922281269340
C	-1.045749406878	-0.920004145660	-2.132789779939
H	-1.864536434562	-1.636019082048	-2.156267184092
C	0.257633962336	-1.398287199961	-2.215329426068
C	1.332912567186	-0.465039830585	-2.119997351341
H	2.353269687817	-0.823204329972	-2.237591729992
C	1.070040089883	0.932397610053	-2.181551199211
C	-2.779546560735	0.959175248676	-1.893521533014
H	-2.793568330127	1.825058935922	-1.218435254901
C	-3.793106312051	-0.064082172318	-1.379801642109
H	-4.778337720648	0.404754664089	-1.285704712195
H	-3.521699283340	-0.464481617605	-0.396564549397
H	-3.904540867642	-0.908511096072	-2.071884600697
C	-3.189387541723	1.447095712103	-3.289499391860
H	-4.205604567443	1.861834931353	-3.271169149307
H	-3.172240215806	0.615551518972	-4.006724110615
H	-2.513067278770	2.227386674054	-3.651766711663
C	0.546225249213	-2.872142226634	-2.435050855285
H	1.637538052234	-2.972812194743	-2.509803989518
C	0.065123433634	-3.745454424207	-1.283400397877
H	-1.024202633669	-3.701103340078	-1.191461666393
H	0.482927406745	-3.420921726963	-0.324261557239
H	0.349002585238	-4.793335777073	-1.442577959190
C	-0.057146095179	-3.341061580390	-3.765012323805
H	-1.152751349648	-3.282894656124	-3.739887824435
H	0.217622818929	-4.384558771234	-3.964605354640
H	0.295039211843	-2.725346160310	-4.600984267605
C	2.235288232816	1.895876166594	-2.330708697145
H	1.824702479656	2.909425700352	-2.258443700976
C	3.278666066784	1.729267783035	-1.224150069135
H	3.743724706482	0.735657874299	-1.246972618367
H	2.823079603055	1.862087833881	-0.235283747652
H	4.073938803892	2.476144014265	-1.334212978416

C	2.864798254212	1.726116681984	-3.716211518272
H	2.120959809639	1.866539439408	-4.509626259133
H	3.299559153870	0.725139780160	-3.834577071585
H	3.665840067793	2.461154948232	-3.864303772889
C	-1.276653002680	4.579038910949	1.589307943490
C	-2.540138037628	4.238914068703	2.138946735181
C	-2.744439304126	4.404831843665	3.506576235304
H	-3.716860952253	4.144175031790	3.924042916265
C	-1.761629334894	4.897116915450	4.357822162889
C	-0.543890488359	5.266255913443	3.792701143370
H	0.240047185275	5.674400565163	4.425929623768
C	-0.285980801235	5.118704266850	2.423925731060
C	-3.685319793352	3.720819876011	1.276306722660
H	-3.407042658635	3.892164132266	0.228459585781
C	-3.915537951884	2.222830715289	1.472336444588
H	-4.689985343669	1.857151954744	0.788632515521
H	-4.249678225845	2.011812823741	2.494245550367
H	-3.001520248300	1.646321188998	1.288975680812
C	-4.993225311779	4.475769735336	1.554193939075
H	-5.772777541210	4.151051420075	0.853060682735
H	-4.863117572795	5.559254252414	1.445083216543
H	-5.362282324566	4.279299687198	2.568619541341
C	-2.063943697982	5.036467961607	5.846917368288
H	-2.915875463121	5.731288081516	5.929580022090
C	-2.485059554268	3.714452843976	6.470052297992
H	-3.330105629850	3.256717232554	5.944006993855
H	-2.781842928449	3.858389562406	7.516929279693
H	-1.654902393123	2.997139931553	6.451799521948
C	-0.911299471387	5.613880635474	6.646158604583
H	-0.607047915666	6.602892708216	6.281759119833
H	-0.034389364259	4.953484495278	6.606402929853
H	-1.195086214900	5.722137550889	7.700257622458
C	1.069904331044	5.548229716695	1.873456303330
H	0.983011649170	5.565808030843	0.780167380076
C	2.161705137093	4.540818814472	2.231108724627
H	1.923535973553	3.545003884905	1.838606822386
H	2.277112940618	4.456269275627	3.320299096710
H	3.128408381043	4.849818294648	1.811898149058
C	1.467229462217	6.942712864135	2.337197779627
H	0.690002915292	7.680327000029	2.102303707751
H	2.396191818037	7.258047765902	1.844786996849
H	1.644123830097	6.977100421162	3.420139030599
C	-0.871094105798	-2.180549342981	2.258808629741
C	0.414905883446	-2.402100561853	2.811951379646
C	0.619089364355	-3.474934605674	3.677506025602
H	1.610913430069	-3.617020255654	4.102192663938
C	-0.416461903922	-4.325230105148	4.043269280972
H	-0.233255900304	-5.160128562499	4.716387414792
C	-1.701927419146	-4.045934853094	3.595874621220
H	-2.543529239139	-4.644079964092	3.942956923475
C	-1.952475276033	-2.977575754702	2.740878714319
C	1.556014373774	-1.447167169490	2.599263017194
C	1.510719023378	-0.159679891001	3.198803931003
C	2.667774326398	0.623003694953	3.208999418365
H	2.630456896498	1.593317311098	3.695546931658

C	3.880568652462	0.169193677104	2.685546911346
C	3.896845661657	-1.070247523564	2.070440785206
H	4.836349091130	-1.437719198982	1.654965067632
C	2.764028645681	-1.889860270826	2.008247880340
C	0.291360160537	0.309129336374	3.987962866900
H	-0.599131664152	-0.034122123859	3.448341758955
C	0.193464731191	1.828033799104	4.140550758949
H	0.344187040527	2.358926167430	3.195646901564
H	0.932215215147	2.216170594698	4.854124753984
H	-0.796417194141	2.103065709039	4.516039755156
C	0.284206017291	-0.322429009021	5.387463358650
H	-0.595877015859	0.013105445850	5.950136924237
H	1.180386132096	-0.018368277230	5.944521821525
H	0.262872914574	-1.416322925332	5.339914722511
C	5.169931730270	0.976506505442	2.802632643507
H	5.979660258380	0.240085140591	2.921497424965
C	5.454025911587	1.773331683117	1.535033984337
H	5.493232441999	1.123184544381	0.653846172858
H	6.414020137236	2.300432099996	1.613181606373
H	4.669470536110	2.520958752264	1.366273124666
C	5.190511361602	1.888014755586	4.025968155664
H	4.952970806622	1.336792183331	4.943805057920
H	4.469421697674	2.709479367990	3.926108183354
H	6.182791796474	2.340457694343	4.145845144323
C	2.907187192709	-3.269257308897	1.381425639683
H	1.910692351529	-3.726129130031	1.351256825889
C	3.451009909979	-3.195208352185	-0.049085929293
H	2.860077002743	-2.501367266288	-0.656686760988
H	3.423019596833	-4.183400554737	-0.524934721972
H	4.491369633284	-2.845899813589	-0.063695359821
C	3.821872493707	-4.178210415873	2.224042539952
H	3.449880378108	-4.290882258986	3.247638993709
H	4.835816391911	-3.761627847881	2.279871390662
H	3.889605190870	-5.176781405482	1.773758313073
C	-3.359856288079	-2.581900319238	2.406163363669
C	-4.019482891009	-3.103568727923	1.273727894344
C	-5.310917241170	-2.672538382127	0.980734813962
H	-5.823270501582	-3.077438918366	0.108625513369
C	-5.976737913642	-1.750725449603	1.769272632708
C	-5.324758414265	-1.263787832436	2.904085867269
H	-5.840567132813	-0.554726505064	3.551663887925
C	-4.027540033336	-1.664245691837	3.245660481461
C	-3.376060028530	-4.196246063036	0.436456115096
H	-2.289942723852	-4.111413429796	0.574411728765
C	-3.684779717008	-4.094917751434	-1.060770532680
H	-3.434999325910	-3.103448020562	-1.454748818872
H	-4.745907851759	-4.279636697525	-1.270500311176
H	-3.109424646647	-4.844042889586	-1.618733236850
C	-3.808866211795	-5.570426068262	0.965117592881
H	-3.533834751958	-5.692852812398	2.018121741361
H	-3.332503994757	-6.376869950580	0.392037952410
H	-4.897489643022	-5.686942720597	0.883160886865
C	-3.403976978407	-1.171065200228	4.544300364102
H	-2.355689830467	-1.491041965186	4.546888782991
C	-3.428338671212	0.349307414106	4.688492180811

H	-2.890766720313	0.837250923106	3.869903543504
H	-2.953579864253	0.646569850741	5.631021096574
H	-4.454105091039	0.740713684959	4.701162433902
C	-4.096595279094	-1.825890014942	5.742094886171
H	-5.156576069434	-1.542219189324	5.785233046937
H	-3.621589726705	-1.513689467576	6.681209123988
H	-4.044405307254	-2.919465531339	5.679822000908
C	-7.376405646566	-1.325242106407	1.355139722237
H	-7.710644419486	-2.022986611390	0.574939740605
C	-7.357390190474	0.083608373409	0.776035683247
H	-6.684397733966	0.152195631955	-0.086093386533
H	-7.009614707755	0.804498734340	1.527026656202
H	-8.360263052145	0.391408564140	0.451331397976
C	-8.358187237591	-1.388811410466	2.520848438959
H	-9.374043262146	-1.138503687100	2.188764251525
H	-8.084303176877	-0.673337287408	3.306627348272
H	-8.382160814723	-2.388233588267	2.972018874363

2Y

Y	0.000003486290	0.003263919371	0.000001521672
N	1.717755555866	-1.424637051757	0.373320292707
H	1.938039791638	-2.148798109636	-0.309160439103
N	-1.717709544198	-1.424635106544	-0.373230221150
H	-1.937982954509	-2.148799125953	0.309250590241
C	-2.586132458541	-1.521133706273	-1.438624616609
C	-2.398508243498	-0.648432622769	-2.540810068653
C	-3.267252387807	-0.651631201808	-3.620724342158
H	-3.086928821491	0.029361036635	-4.451528786159
C	-4.362404902051	-1.511930804241	-3.646169764808
H	-5.051054579258	-1.508449356224	-4.487980648195
C	-4.540783068876	-2.402531846198	-2.589255140581
H	-5.365717089366	-3.114041011257	-2.613714412513
C	-3.670168271637	-2.440333274366	-1.501691070757
C	-1.196859532373	0.229066867387	-2.444333911756
C	-1.265423444851	1.532766346790	-1.920726578737
C	-0.063081256182	2.236965679149	-1.669010662457
H	-0.131059167496	3.249119358846	-1.275910720082
C	1.197640145557	1.744965712601	-2.109901084802
C	1.241275411647	0.448866221499	-2.618036233548
H	2.196121586108	0.035629907672	-2.931321309864
C	0.085413746442	-0.353333252421	-2.748656303405
C	-2.558399929489	2.300566509954	-1.653913064761
H	-2.272632857533	3.093585888347	-0.946595406375
C	-3.724374895968	1.528566262838	-1.019472213133
H	-4.266184700054	0.923825873632	-1.751075102639
H	-4.429840075233	2.243335249134	-0.577528685209
H	-3.371521214740	0.857850074375	-0.227921226532
C	-3.007452470050	2.992868021240	-2.947453193156
H	-2.198342556405	3.591941012615	-3.382245727636
H	-3.860180995356	3.655285878159	-2.750599996965
H	-3.320700681083	2.249253832442	-3.688949743506
C	2.393471750165	2.682165280770	-2.095314327515
H	2.494851406065	3.069241573484	-1.069519489407

C	3.710696951799	2.028365255649	-2.492139809365
H	3.938905462190	1.158293571090	-1.870615491660
H	4.534224102063	2.743952342823	-2.383932684727
H	3.690691709268	1.702364477737	-3.540353290851
C	2.112152151003	3.883266365067	-3.013608531307
H	1.997515535047	3.548425871474	-4.052480817799
H	2.942634829261	4.599341856232	-2.974459059057
H	1.195544620885	4.411557841692	-2.728805385265
C	0.154442152378	-1.702832546546	-3.449933731387
H	-0.704864046701	-2.288749034228	-3.100154832500
C	1.416005158209	-2.513533258161	-3.182914560616
H	2.315666344789	-2.020443519171	-3.569995531307
H	1.343573267932	-3.484822834903	-3.684918455645
H	1.570645518559	-2.711330982385	-2.118141357412
C	-0.009019496653	-1.484230927933	-4.960869258445
H	-0.944851561006	-0.962315368027	-5.186998463003
H	-0.018080108439	-2.444586875893	-5.492440123196
H	0.822086132359	-0.883495876970	-5.354040712779
C	-3.807895680211	-3.484034337603	-0.435510147771
C	-4.514772215979	-3.219735333445	0.750381754676
C	-4.606777528971	-4.215036318655	1.725688741461
H	-5.161751304051	-4.019733623596	2.642508047289
C	-4.001071656005	-5.456336360352	1.568414643619
C	-3.296814301182	-5.697335372463	0.391862994492
H	-2.808917663953	-6.662148041616	0.250731740620
C	-3.192511859508	-4.740434357033	-0.616255104640
C	-5.221254416102	-1.885535300567	0.949464731223
H	-4.709582647310	-1.148381206108	0.316641221286
C	-5.184958044318	-1.390936805432	2.397202083331
H	-5.647432569507	-0.399344618611	2.470007728813
H	-4.158195078549	-1.319580031049	2.770608556320
H	-5.742595243708	-2.056160509594	3.068126156154
C	-6.671925573384	-1.994634298014	0.477841071618
H	-7.210825314601	-2.749834545377	1.065002920999
H	-6.723289403719	-2.286898623665	-0.577083768316
H	-7.195088978379	-1.035651568059	0.590684268025
C	-4.073415873398	-6.501337485309	2.671229861847
H	-4.768722239488	-6.116514553020	3.431871659722
C	-2.703798395427	-6.673238635628	3.331230302646
H	-1.967127385597	-7.046459729862	2.608070856983
H	-2.752758148809	-7.388309080852	4.162962564431
H	-2.331514679293	-5.718392702177	3.719712766076
C	-4.614319718608	-7.837236787236	2.173505635075
H	-5.600248190343	-7.718377720922	1.708494883458
H	-4.709135095293	-8.551490642355	3.001567485273
H	-3.945249402056	-8.284369238750	1.426914098423
C	-2.422832837747	-5.081133297489	-1.888002563288
H	-2.519106467784	-4.221327217131	-2.562115419156
C	-0.943337518304	-5.275634104937	-1.596881746240
H	-0.776107826080	-6.127439811496	-0.925560822634
H	-0.515592738676	-4.386705786856	-1.120912155438
H	-0.383496260207	-5.466749138006	-2.521402281549
C	-3.019844272065	-6.289532368757	-2.587961777553
H	-2.493772064888	-6.490536739869	-3.530600642222
H	-4.080583563391	-6.131731366585	-2.817808375882

H	-2.944406534327	-7.193453330161	-1.968832266670
C	2.586178404413	-1.521138452968	1.438714487998
C	2.398554781830	-0.648439533474	2.540901753930
C	3.267298923307	-0.651640945342	3.620816021929
H	3.087009474629	0.029337317942	4.451636781592
C	4.362410845813	-1.511941339997	3.646169658797
H	5.051068858414	-1.508455664850	4.487980111378
C	4.540828417371	-2.402540306885	2.589343178959
H	5.365770020689	-3.114037064208	2.613820111646
C	3.670213593560	-2.440338887014	1.501779032008
C	1.196906666220	0.229060972551	2.444427422225
C	1.265471463602	1.532761492690	1.920822798513
C	0.063129751976	2.236962165938	1.669108346290
H	0.131104070703	3.249116068751	1.276006538878
C	-1.197591982393	1.744962138673	2.109997748456
C	-1.241228127943	0.448861622090	2.618130203250
H	-2.196074781546	0.035624807452	2.931417118889
C	-0.085367008564	-0.353338909044	2.748748603746
C	2.558448469113	2.300561333481	1.654010881009
H	2.272664720675	3.093594307644	0.946713811275
C	3.724392903361	1.528561615958	1.019478429439
H	4.266226546231	0.923781150852	1.751031815703
H	4.429846587681	2.243335679446	0.577525759068
H	3.371477857419	0.857885355176	0.227920898746
C	3.007501480851	2.992859851163	2.947552448005
H	2.198380223771	3.591917271033	3.382345946395
H	3.860218301902	3.655292939278	2.750699891310
H	3.320762051542	2.249249761861	3.689049062101
C	-2.393422950741	2.682162549035	2.095412941153
H	-2.494734476051	3.069266325070	1.069618507108
C	-3.710598611351	2.028362596026	2.492097053864
H	-3.938745658030	1.158301070650	1.870533846932
H	-4.534148093931	2.743919926282	2.383848479882
H	-3.690689538306	1.702322830665	3.540302607629
C	-2.112102533225	3.883261533985	3.013709640118
H	-1.997497375827	3.548427071492	4.052588653153
H	-2.942573200016	4.599350307955	2.974538363559
H	-1.195478903167	4.411531481477	2.728921539935
C	-0.154396332059	-1.702839614281	3.450023225938
H	0.704910686695	-2.288754897052	3.100241585850
C	-1.415959888454	-2.513538913588	3.183002368453
H	-2.315621774231	-2.020448962886	3.570082613273
H	-1.343528529919	-3.484829257663	3.685005427592
H	-1.570598565353	-2.711336463638	2.118228637030
C	0.009065464049	-1.484241248066	4.960959207637
H	0.944897937068	-0.962325496639	5.187090365306
H	0.018125883666	-2.444599624005	5.492525980554
H	-0.822040778639	-0.883507338082	5.354132888436
C	3.807940294988	-3.484037828097	0.435595940270
C	4.514907004064	-3.219736836131	-0.750335421590
C	4.606821646116	-4.215035856427	-1.725604467812
H	5.161781674546	-4.019762586521	-2.642441692253
C	4.001114935154	-5.456335815943	-1.568332952159
C	3.296857419023	-5.697336798388	-0.391781803337
H	2.808961803675	-6.662150513132	-0.250652666231

C	3.192555625236	-4.740437807856	0.616338285180
C	5.221300106139	-1.885536870861	-0.949375622440
H	4.709608160347	-1.148409256271	-0.316539981011
C	5.185004062304	-1.390935344432	-2.397111947168
H	5.647480225137	-0.399344685638	-2.469922500153
H	4.158240793856	-1.319580543776	-2.770516833132
H	5.742639639936	-2.056163677596	-3.068032010726
C	6.671971191758	-1.994637824664	-0.477752194970
H	7.210872803139	-2.749818228600	-1.064936832839
H	6.723332597758	-2.286936218364	0.577163179270
H	7.195135332734	-1.035651000325	-0.590561727909
C	4.073458444151	-6.501334695511	-2.671150344529
H	4.768765447894	-6.116510902561	-3.431791090656
C	2.703840848876	-6.673233546654	-3.331151140733
H	1.967170287419	-7.046454563414	-2.607991559754
H	2.752799096206	-7.388301858534	-4.162885182713
H	2.331557591238	-5.718386430792	-3.719630807165
C	4.614361386859	-7.837235398652	-2.173428897850
H	5.600289631928	-7.718377906129	-1.708417263942
H	4.709177130748	-8.551487170506	-3.001492548668
H	3.945290448417	-8.284368999985	-1.426838719178
C	2.422876374540	-5.081138872165	1.888085036295
H	2.519151622177	-4.221335187833	2.562201019928
C	0.943380923319	-5.275638074798	1.596963816759
H	0.776151198357	-6.127439603392	0.925637538618
H	0.515636846410	-4.386706354861	1.120999578751
H	0.383539602539	-5.466759330044	2.521483344737
C	3.019886995652	-6.289539799145	2.588031739647
H	2.493817273989	-6.490551055971	3.530671239409
H	4.080626717470	-6.131742760552	2.817878764251
H	2.944444725642	-7.193457826811	1.968898412239

2La

La	-0.007917724825	0.021553431285	0.033576907125
N	0.825699945896	-2.241100002631	0.185399989305
N	1.735999997039	1.644200033108	-0.451800010729
C	1.296099981588	-5.241100000848	2.322999998031
C	1.630499946411	1.996100042260	-2.828799981579
C	5.913299971453	2.953799972339	1.232200009892
C	3.375800057445	3.126399986207	-1.587700006944
C	0.290599965752	-3.483100023710	3.601400000778
C	1.930800012698	-4.940100026822	-0.091399989330
C	2.089099961828	-0.851699894351	-3.530100050028
C	0.871200020289	-3.097700015517	1.262300003964
C	1.374000015454	-4.423999993376	1.200600001481
C	0.357800035077	-2.640399970702	2.504799987919
C	5.446600066789	3.922200051134	2.111799962266
C	5.269300026294	2.690300020309	0.021900021309
C	1.076399991781	-5.546700011959	-1.018000018291
C	3.639900175514	4.419800223191	0.561399873298
C	0.754800016680	-4.791299997025	3.522900005546
C	0.491100055578	1.031199989129	-2.826399963099
C	-1.658899967552	-0.821599805413	-2.737200109070

C	-2.807499964143	-1.832200100524	-2.839500042175
C	-1.870200012422	0.545799947746	-2.497699995883
C	3.300599974218	-4.779599950767	-0.396999991541
C	-0.817300012466	1.476299977304	-2.540099955208
C	-2.481299977322	-2.112599987136	2.137000034795
C	2.635100049098	-2.018000056976	-2.696400011542
C	1.983499997270	-1.240700014157	-5.011899960769
C	2.197799998430	-0.408699997915	4.696399999142
C	6.141499987052	4.197500019070	3.444300002626
C	-0.403699982826	-5.773599994546	-0.717199954273
C	2.208000007155	-0.433799999209	3.157100006588
C	2.913699942783	-5.846099937152	-2.559500023859
C	5.818900022033	1.621700039387	-0.926100060777
C	4.766700012528	-5.059000016321	1.621700016053
C	-1.316000003983	-5.310399957038	-1.846600045110
C	7.507600002430	4.845299995181	3.238999998912
C	-2.946500015703	-2.278000004337	0.700199963849
C	4.585399981902	-7.209699994961	-3.850200003920
C	-4.181900021605	-1.234699981658	-2.593499972584
C	6.251200003878	2.941399994215	4.284099997889
C	-2.208599988083	3.194799994109	-1.285600009607
C	3.406900054171	-6.272900029690	-3.954499988032
C	5.423900018497	-3.406500014147	-0.096899981107
C	-0.649300000332	-7.238300002680	-0.384500006557
C	3.285599997478	0.525299995474	2.634100003223
C	3.739399986942	-5.085299990967	-4.816499999494
C	1.694200008751	5.854600004277	1.397899992035
C	7.414399993055	1.650099988190	-1.024099981470
C	-0.346100063052	-1.238600194514	-2.970999754443
C	5.349699990326	0.283699975728	-0.667799977736
C	2.096200029046	2.562999960986	-3.995900006913
C	3.813499983226	3.678500013619	-2.775900001499
C	3.767600036262	-5.252900052143	-1.627499987133
C	4.321199884114	4.644599873716	1.759300071168
C	-2.773500005752	-2.510699980430	-4.210399985560
C	4.237399976248	-4.101299980432	0.595599961010
C	2.433699946839	5.279199937817	0.184500064058
C	2.252499998519	2.254399975326	-1.575299992928
C	1.589600016022	-5.993000011400	-2.239999988678
C	-3.638800009457	-1.977800003275	3.107599995385
C	-1.420200001636	3.632200000402	-3.622200005510
C	3.200599989575	3.416400015498	-3.991100001145
C	4.108699883689	3.417799878033	-0.305999946115
C	0.730900024380	-0.343899927173	-3.062900214956
C	-1.094900009152	2.956000012721	-2.309899994221
C	2.848900007813	6.422500006653	-0.729400019440
H	1.125999997366	-2.631600015485	-0.479699958382
H	2.272448138014	1.940435220896	0.365504057213
H	1.664161872696	-6.264070442666	2.244585101806
H	6.805356613330	2.382911016413	1.487886034834
H	-0.131842635659	-3.105583169868	4.532547960212
H	2.792202713533	-0.015527724143	-3.442908050387
H	0.700971296146	-5.451693570726	4.385816812049
H	-2.628139299482	-2.605587211947	-2.080609850552
H	-2.879168025721	0.903041378916	-2.311731271948

H	-1.942805321365	-3.030413464209	2.399855069819
H	2.764137851982	-1.728886190032	-1.648242324319
H	3.613477302746	-2.325499386406	-3.081344202021
H	1.984449712552	-2.901357465955	-2.730296065740
H	1.285535971954	-2.077048568033	-5.149221056845
H	2.963278926380	-1.551672784402	-5.394845818900
H	1.628874196448	-0.399728358929	-5.619711435978
H	1.927085731372	0.591735863035	5.060164252035
H	3.186812859478	-0.663961968866	5.099702762063
H	1.470034181496	-1.123768441673	5.097442293389
H	5.507229497551	4.915331183576	3.985582059188
H	-0.645941101672	-5.178347638159	0.172841733318
H	2.469226977201	-1.449525902293	2.833311290692
H	0.918991266010	-6.460987968877	-2.961685558067
H	0.922226973963	2.018805599560	2.872075475147
H	5.473829370154	1.913631342195	-1.927146175944
H	-3.277853686931	-1.888916369971	4.139379061029
H	-4.290673813434	-2.858934907769	3.049871630569
H	-4.255430390985	-1.094179845353	2.893160959843
H	-3.005087279840	0.546870282198	1.870420881613
H	3.961102093959	-5.522133449420	2.202042378810
H	5.431944267038	-4.544841969939	2.328463040574
H	5.342111753220	-5.863560236221	1.143152791337
H	-2.369225797954	-5.387968160590	-1.547000142337
H	-1.187352733313	-5.911852421231	-2.755929117787
H	-1.111975790459	-4.264636902984	-2.103268088204
H	7.977595856482	5.082943185852	4.202511420619
H	7.424214552396	5.772541712834	2.659407411478
H	8.181926967348	4.171357275210	2.693736422208
H	-3.465210546016	-1.380531934220	0.338879035581
H	-3.634348581538	-3.127367885559	0.597656761573
H	-2.083216138504	-2.472433355384	0.049011852862
H	4.896120789606	-7.567065447019	-4.841367212353
H	4.350387298819	-8.085089151500	-3.232554743673
H	5.452525402060	-6.709717597256	-3.398422860846
H	-4.438576830463	-0.496036839988	-3.364764261075
H	-4.254429180525	-0.744803447701	-1.615741625245
H	-4.942794249275	-2.023220212101	-2.624403395122
H	-0.595185427808	3.531968979944	-4.336166821390
H	-1.605072601710	4.703715455656	-3.471547404652
H	-2.319758964967	3.194142108526	-4.075760189666
H	6.885326272373	2.189517945905	3.795909729289
H	5.268154291913	2.485343271575	4.451477771719
H	6.696917013572	3.161125720256	5.263362829881
H	-3.195021281556	2.915209204528	-1.677164249593
H	-2.250867455877	4.256554391732	-1.015966763481
H	-2.040842582044	2.623963118329	-0.363450625587
H	2.573136191917	-6.817479919077	-4.422521646605
H	6.127289364423	-4.138288319626	-0.514974773436
H	5.977273795255	-2.800317739437	0.630424135096
H	5.092427720981	-2.746399969207	-0.905265138494
H	-1.704695902395	-7.414397069786	-0.136063867422
H	-0.042090229950	-7.554026062737	0.471951585984
H	-0.389445610959	-7.882834053778	-1.235205511592
H	3.358915256638	0.482118758382	1.542500802583

H	4.266767119299	0.259321263486	3.045382549455
H	3.088046272159	1.567237770782	2.918904822553
H	2.881169698879	-4.411514039735	-4.926628012294
H	4.052181538032	-5.402099147588	-5.820722479235
H	4.562073335436	-4.500626017602	-4.382512150930
H	2.259820630124	6.684130958587	1.843652699801
H	1.537904972714	5.103427324761	2.174771313775
H	0.719131318576	6.253227135237	1.095092793102
H	7.775800477887	2.667759885120	-1.208256887949
H	7.730680976426	1.002913634570	-1.850452167927
H	7.875701304902	1.273859533075	-0.102234974675
H	-0.153457893419	-2.296769575050	-3.145112566896
H	5.663651543748	-0.087174046384	0.319143259801
H	5.733147140241	-0.425918184306	-1.413548190720
H	4.253364680913	0.218923216116	-0.695557824269
H	3.567769629326	3.860346997187	-4.913234694560
H	-2.545093085943	2.841699039253	1.743794902249
H	1.597335117962	2.323477519783	-4.935548043877
H	-1.340340744124	4.924868120584	2.225316308844
H	-0.282196584507	3.863194069834	1.265778734077
H	-0.030904652397	4.038105799579	3.011833091822
H	4.686515118112	4.331367384789	-2.744210829316
H	-1.624766834769	2.894137398293	4.663555845193
H	-3.082175724633	2.084405301534	4.056689358523
H	-2.950947425215	3.857535649023	3.969496754867
H	-0.165795392613	3.390426663001	-1.916425485354
H	4.818674425161	-5.124558806804	-1.879467937813
H	3.962994163167	5.414749058081	2.441440501382
H	1.981021866481	7.040088133895	-0.997810775229
H	3.299099101998	6.053915687903	-1.657178023739
H	3.581857754996	7.067030654944	-0.226203404026
H	-3.572648703559	-3.258640949450	-4.284472126777
H	-1.822841438403	-3.022383154912	-4.394671904322
H	-2.920200931991	-1.772989484794	-5.009962012328
H	3.645523344319	-3.329983050937	1.109686800860
H	1.739517750877	4.630293879019	-0.369352011818
C	-0.141548483805	-1.236250733769	2.519073436451
C	0.259927519897	1.185400721671	2.642347440613
C	-1.943230258807	0.352384480925	2.010524206032
C	-1.078823526259	1.445289265908	2.357042010665
C	-1.734802586299	2.806291326253	2.488417585282
C	-0.794227500077	3.974009048693	2.227944784309
C	-2.391902429237	2.917183311279	3.879228579970
C	-1.491311391918	-0.981366525556	2.261811242093
C	0.810677564329	-0.143407960281	2.632269714436

2Sm

Sm	-0.000617723342	0.000913621903	0.000169642298
N	2.373598984915	0.260219968743	0.355370697922
N	-0.755978525374	-2.281252442207	-0.335660491220
C	3.130942155528	0.634569705519	1.433786568392
C	2.479441660089	0.904237120709	2.663761388048
C	3.188519833745	1.342729874082	3.771718878375

C	4.566542705507	1.507429027169	3.715844709295
C	5.230097580911	1.212539890902	2.534287683186
C	4.548765022025	0.773756964303	1.401416447557
C	1.000579719720	0.705602555901	2.688255945562
C	0.469824495753	-0.594991499452	2.896939741516
C	-0.903503038744	-0.787545231061	2.819029498130
C	-1.795419709970	0.259442706060	2.590462246719
C	-1.248560016161	1.530233749723	2.382136259510
C	0.133784910058	1.770123381703	2.421627848547
C	1.385900595983	-1.733541719612	3.318234512039
C	1.025600707869	-3.088196934932	2.712606424322
C	1.358726743047	-1.821687135207	4.847091082756
C	-3.293483073407	-0.018574325098	2.645346841430
C	-3.646077591530	-0.776755514368	3.946207743030
C	-4.148014779626	1.215381558919	2.545124805679
C	0.687705688264	3.173070480216	2.226809432009
C	0.147499496747	3.835733421224	0.983906651194
C	0.414650341819	4.049870986280	3.394529599761
C	5.273060638401	0.476664991636	0.126912801628
C	5.687190766708	-0.832130834061	-0.177990083850
C	6.312975741203	-1.069074853952	-1.401354322270
C	6.516713832974	-0.063286999806	-2.343220447703
C	6.101144126990	1.214545701348	-2.021083970809
C	5.483800442530	1.507049159010	-0.802706899018
C	5.449237282353	-1.963723142015	0.818353550139
C	5.295134378269	-3.326380117879	0.131660800611
C	6.545438907385	-2.036966495634	1.851648590420
C	7.137000957714	-0.347613395339	-3.702055249886
C	6.212250943660	-1.196525815738	-4.560252410740
C	8.512314668546	-0.990789613481	-3.575538715333
C	5.078595315044	2.941855054745	-0.506437230514
C	4.198243605004	3.525673292194	-1.612632875112
C	6.305790479282	3.808304810260	-0.272247847689
C	-1.094755743003	-2.997651375030	-1.451268330031
C	-1.134956699798	-2.325316829513	-2.709335465076
C	-1.462864799521	-2.994698658395	-3.871201598454
C	-1.766645019673	-4.351045164217	-3.861177135380
C	-1.729093971292	-5.022520651577	-2.646760435657
C	-1.412307583700	-4.389441176848	-1.462573962940
C	-0.760822069021	-0.880414716051	-2.711546534509
C	-1.721599842823	0.106542037558	-2.411971220452
C	-1.337360750424	1.454505549068	-2.379799030514
C	-0.018794543539	1.851460895994	-2.615421109405
C	0.922340260702	0.853578250117	-2.866753037337
C	0.580164081112	-0.496561452423	-2.957436089177
C	-3.178216128701	-0.284359095127	-2.171179357188
C	-3.847853466754	0.570972842587	-1.099963633185
C	-3.959732572960	-0.235481752046	-3.466637602074
C	0.403301165523	3.318348977560	-2.692606954166
C	-0.716894362901	4.302935826680	-2.448808373705
C	1.074842329620	3.615971218603	-4.041175784367
C	1.629929904826	-1.507806410013	-3.399835937351
C	2.892708747266	-1.489015306842	-2.544064939501
C	1.972269512303	-1.259980198685	-4.869589448073
C	-1.375531799794	-5.154042283841	-0.167945731340

C	-2.484329651839	-5.117297690770	0.687289293420
C	-2.437991873811	-5.812613842630	1.896923308342
C	-1.327809664560	-6.535770733116	2.273513822702
C	-0.234480726224	-6.570071946708	1.415465763678
C	-0.236137334546	-5.892200197717	0.186678485863
C	-3.745491720729	-4.359659889644	0.293811201003
C	-4.499825823486	-3.768017159367	1.490625871417
C	-4.669276684492	-5.251528628416	-0.533164177161
C	-1.330018012110	-7.275712173314	3.606118033637
C	-0.235010574936	-6.796213876537	4.530521364717
C	-1.288959105105	-8.784341152188	3.409751033275
C	0.972268675636	-5.962170710227	-0.731090405249
C	1.638823484914	-7.364920183435	-0.692314326357
C	1.921159065333	-4.881854774230	-0.523932341025
H	2.988862077730	0.103960603808	-0.442291638851
H	-0.795066424726	-2.896010609337	0.478281536194
H	2.649169487995	1.554083774510	4.695259029463
H	5.117188131624	1.853504919485	4.588024435050
H	6.311870025577	1.331712802724	2.473670269678
H	-1.297320522484	-1.790283272462	2.972153275757
H	-1.916811179895	2.373824451318	2.217848570390
H	2.401825880887	-1.467361370886	3.002395697748
H	0.035939918282	-3.441763579136	3.029020099869
H	1.751572756553	-3.845540121451	3.031200628050
H	1.039798492284	-3.054488950863	1.618580778737
H	1.665496291423	-0.875267063318	5.307587496279
H	2.038612934443	-2.607619932074	5.199904507692
H	0.347097433832	-2.061584431830	5.200856386145
H	-3.531353463740	-0.686403765546	1.802364334620
H	-3.105896193640	-1.723988330435	4.036764688732
H	-4.719232646023	-1.001237158516	3.970544732629
H	-3.401806555135	-0.161637459174	4.821637050654
H	-3.965214917494	1.893525791872	3.389703779614
H	-5.210504784522	0.944767666713	2.563559818759
H	-3.973401093665	1.778300477947	1.620287452276
H	1.772748505382	3.060733563080	2.111150230696
H	-0.931455069957	4.028661539390	1.057300621771
H	0.318507285189	3.216503661775	0.093867215479
H	0.641309651634	4.799645318940	0.809078397966
H	-0.665309729033	4.178563326618	3.555916504169
H	0.846199005113	5.048092453131	3.243972248124
H	0.837675686562	3.644381012418	4.322419064946
H	6.629541315184	-2.083116699360	-1.640547350602
H	6.261311666902	2.016499098078	-2.743390070207
H	4.504298565389	-1.733803673964	1.331379858392
H	4.552296156667	-3.292261547552	-0.671862627407
H	4.970063819463	-4.079018727013	0.860444353708
H	6.246152183531	-3.674359795967	-0.292188362015
H	7.515731211768	-2.233533364756	1.375328151532
H	6.351889462138	-2.846043730008	2.568884355310
H	6.630054522487	-1.102557381487	2.417097660719
H	7.263789547081	0.624689449711	-4.201140062064
H	6.042463057208	-2.179084665825	-4.100177432295
H	6.642530243665	-1.363973075252	-5.556613170640
H	5.234454162143	-0.717415595894	-4.686681039203

H	9.185859181348	-0.372150334297	-2.970511215896
H	8.970421344757	-1.132386666265	-4.563174436386
H	8.446104309456	-1.976926462113	-3.097488278129
H	4.487836742670	2.928167686915	0.417949142702
H	4.740166893399	3.616625849190	-2.562895368070
H	3.842990077244	4.527377346287	-1.336924838948
H	3.323075482559	2.888128080618	-1.780940410470
H	6.909623828806	3.420324029049	0.556853965363
H	6.018511487263	4.840221286898	-0.029580160887
H	6.944717029069	3.836940626690	-1.165227677094
H	-1.471361999258	-2.442171622848	-4.811506854981
H	-2.020880714344	-4.873082599607	-4.780667432854
H	-1.953904223812	-6.089390784860	-2.614660878145
H	-2.095455343234	2.211198761554	-2.193914050031
H	1.958938845664	1.139053961176	-3.041817558723
H	-3.162714175697	-1.324105524111	-1.817933550500
H	-3.247899838928	0.598264954319	-0.181611235172
H	-4.830870422952	0.158969206027	-0.842003199127
H	-4.003415863301	1.605871431697	-1.431712010524
H	-3.964515530627	0.780498073742	-3.884770874330
H	-5.002108532159	-0.539440263474	-3.304428451088
H	-3.526626782574	-0.908789982669	-4.214825864149
H	1.166819446034	3.471269832358	-1.914611654176
H	-1.483150480675	4.236607470962	-3.233234809585
H	-0.325883209444	5.327230325251	-2.456793038059
H	-1.210147962897	4.147460243111	-1.482659518270
H	1.948828398469	2.979797224899	-4.214940549618
H	1.410391960474	4.659729999307	-4.074521959840
H	0.369267261966	3.455209873188	-4.866573500338
H	1.174712184116	-2.501227140728	-3.313840210880
H	3.411791890024	-0.522084018568	-2.581073284834
H	3.601674833745	-2.243875725819	-2.902656128469
H	2.667777069357	-1.716982845329	-1.496694449639
H	1.077370237178	-1.306608716390	-5.501760853671
H	2.684836927440	-2.012405596895	-5.230136760981
H	2.429949306676	-0.270913947103	-5.004970635341
H	-3.300385966142	-5.791201806483	2.562166393235
H	0.647035954425	-7.141973624008	1.701676614181
H	-3.427876473755	-3.524129141872	-0.345248393761
H	-4.966285843597	-4.546190099103	2.108236912111
H	-5.302715600523	-3.107381329222	1.139101973268
H	-3.830931343794	-3.182096725168	2.131357513936
H	-4.164531002955	-5.606810940012	-1.437936143532
H	-5.570359012377	-4.703477953432	-0.839956868358
H	-4.984145188187	-6.127648725656	0.049452727677
H	-2.292605804878	-7.036190629219	4.083811982518
H	0.760077395466	-7.002695255084	4.113253764347
H	-0.294090239608	-7.298213359491	5.505345764042
H	-0.301522036862	-5.714570621074	4.699999089231
H	-2.116680992883	-9.122411998712	2.775028667313
H	-1.357813062266	-9.308040659192	4.372445980223
H	-0.352349548697	-9.094844253372	2.927112554865
H	0.570430735061	-5.855533624963	-1.749027992496
H	2.163272413635	-7.541467543735	0.256033764169
H	2.382165794525	-7.443988651270	-1.495585580020

H	0.894495573338	-8.157796335220	-0.827314220490
H	1.443696449765	-3.898156932485	-0.617092707899
H	2.737711905907	-4.919954685610	-1.258137295827
H	2.382994367385	-4.923404269162	0.473037550230

2Eu (using Eu1)

Eu	0.000003213907	-0.001617816055	0.000001148997
N	1.893170708009	1.423781230600	0.454057828770
H	2.174830029878	2.083897768913	-0.271327261282
N	-1.893262741749	1.423783138188	-0.453945846001
H	-2.175079039431	2.084040909077	0.271243665170
C	-2.695824718040	1.597283433196	-1.543604982095
C	-2.460232448760	0.801683167655	-2.704935083653
C	-3.286158707025	0.894983474300	-3.814499230808
H	-3.078464592904	0.269470298115	-4.683011447264
C	-4.341074588750	1.803084033123	-3.844274687753
H	-4.983294373798	1.870459777559	-4.719937373134
C	-4.534984515333	2.638884265223	-2.765963472193
H	-5.314264102061	3.400826947963	-2.810003470789
C	-3.741132157305	2.582583982143	-1.627842494991
C	-1.213593969583	-0.027617496618	-2.710140464963
C	-1.221687066299	-1.406717446803	-2.345568568031
C	-0.004410069989	-2.102618091443	-2.311097711342
H	-0.007311406679	-3.162768178342	-2.055537099946
C	1.214376913380	-1.503118774698	-2.608647341520
C	1.204797245749	-0.153018810153	-2.939175469790
H	2.143778796185	0.345687558377	-3.170577153424
C	0.016851780733	0.586381812453	-3.013512578829
C	-2.522421857553	-2.112316715603	-2.037461475602
H	-3.283147640501	-1.376471322325	-1.754992232418
C	-2.361097258852	-3.221316676594	-0.932655666655
H	-1.772328695609	-4.060660394648	-1.314610052888
H	-3.349466663393	-3.597877007671	-0.648614262384
H	-1.869799981793	-2.852371203107	-0.021668196157
C	-2.935185317129	-2.972216620781	-3.131473494758
H	-3.184322295287	-2.420843432284	-4.052069763794
H	-3.824872912414	-3.561044786296	-2.873608045939
H	-2.139963507849	-3.678803982912	-3.402127980466
C	2.488592752399	-2.346919456931	-2.673178599675
H	2.313430315041	-3.225753112922	-2.035576554523
C	3.715115633365	-1.615620044083	-2.145079874710
H	4.566283415945	-2.303326193866	-2.068745942714
H	3.535958154908	-1.175770007532	-1.156567446067
H	4.012858010884	-0.804658305772	-2.818451031644
C	2.698513320379	-2.830719729976	-4.078023279853
H	2.856166043066	-1.987530257378	-4.764254064208
H	1.833605203801	-3.396838854795	-4.445502328460
H	3.580433846141	-3.481880622785	-4.142955903163
C	0.065219397743	2.045781733671	-3.450983063724
H	-0.871459318273	2.509947928185	-3.121959663263
C	1.215832285753	2.826881185800	-2.876018176368
H	2.195127828080	2.445508099129	-3.191073442356
H	1.192409313556	2.840913525584	-1.781336668157

H	1.159940861370	3.866006214529	-3.207383710010
C	0.104803959070	2.085981520912	-4.979281534282
H	0.097497945816	3.123621251891	-5.336923892210
H	-0.759909940599	1.571001739782	-5.413779191170
H	1.016467394852	1.603207557855	-5.356935893805
C	-3.855264109699	3.593584174665	-0.512538285964
C	-4.573363885255	3.283384698640	0.663324698922
C	-4.539945677884	4.226884809547	1.732587496484
H	-5.076836927463	3.992825084814	2.650650214325
C	-3.834433737389	5.411384424626	1.644737109883
C	-3.185195063658	5.723183934922	0.463688787641
H	-2.646338162674	6.666223719032	0.376501355865
C	-3.187873839017	4.821483807022	-0.608340558883
C	-5.376190248190	2.023285132381	0.730605813226
H	-4.818376545795	1.252436915957	0.179634443723
C	-5.614897069165	1.515385432604	2.155606740486
H	-4.674696846569	1.435375692322	2.715060709288
H	-6.087376719180	0.526254594208	2.132330679553
H	-6.283012881115	2.183933076418	2.714210830343
C	-6.742941996954	2.201685785388	0.017706830468
H	-7.317088667395	1.266805118342	0.052192910955
H	-6.603967486542	2.481173103391	-1.030913113720
H	-7.327261503172	2.987832122182	0.512989572378
C	-3.792651105749	6.377384547485	2.828648123534
H	-3.171806913043	7.230338015685	2.515056124643
C	-3.140900657840	5.756984347472	4.053159433247
H	-3.664088632775	4.843192156137	4.363869243727
H	-3.161152497822	6.457027818904	4.899024272908
H	-2.096000824633	5.494513982570	3.860568400882
C	-5.158168950441	6.891685317316	3.166348855236
H	-5.640461191016	7.359819490668	2.299007228836
H	-5.113784495004	7.637537771444	3.971654485086
H	-5.816842447426	6.081155622481	3.507825310688
C	-2.461683523384	5.244283264220	-1.875865284402
H	-2.549162869825	4.413329484798	-2.587139084319
C	-0.962836618164	5.491782491816	-1.656384497999
H	-0.790853157733	6.344170154144	-0.986675003536
H	-0.470858997277	5.715342406597	-2.611472642013
H	-0.477081378811	4.615935884069	-1.214621544772
C	-3.103735134128	6.456083533869	-2.496848717350
H	-4.166205755137	6.283399586758	-2.710890169252
H	-2.608428051624	6.720198974044	-3.441001966366
H	-3.039654628150	7.330667632729	-1.834765186021
C	2.695722845959	1.597280935133	1.543616923309
C	2.460129733132	0.801681200579	2.704947216879
C	3.286156084672	0.894980896445	3.814501324721
H	3.078452916524	0.269427572069	4.682987337679
C	4.340982949630	1.803080341024	3.844376579584
H	4.983215745017	1.870455327207	4.720026444463
C	4.534893744048	2.638880112475	2.765975161072
H	5.314152560161	3.400844660429	2.810009574968
C	3.741041347985	2.582580394388	1.627954199041
C	1.213510393543	-0.027618135733	2.710252789414
C	1.221602019379	-1.406718182043	2.345671223318
C	0.004304261130	-2.102617538631	2.311110536830

H	0.007208873876	-3.162828044912	2.055797349867
C	-1.214462065336	-1.503116853189	2.608750014299
C	-1.204890978615	-0.153016818013	2.939177822155
H	-2.143873968321	0.345705101977	3.170539147280
C	-0.016944723506	0.586382557296	3.013524754319
C	2.522316039866	-2.112318911955	2.037464307473
H	3.283035377005	-1.376451584293	1.755028006955
C	2.361100270234	-3.221318962201	0.932748742671
H	1.772300615945	-4.060656345273	1.314669425189
H	3.349494163721	-3.597878798788	0.648796030870
H	1.869872685661	-2.852435608579	0.021694686057
C	2.935088577247	-2.972218989391	3.131476536285
H	3.184211531818	-2.420851742007	4.052081402338
H	3.824783281699	-3.561041892831	2.873626193872
H	2.139874298183	-3.678817111264	3.402126658521
C	-2.488698824735	-2.346916164956	2.673191474699
H	-2.313514886186	-3.225756324053	2.035611397599
C	-3.715220924565	-1.615615566853	2.145082568847
H	-4.566378045449	-2.303331535606	2.068721492513
H	-3.536041655854	-1.175749314745	1.156581390797
H	-4.012982250523	-0.804666681661	2.818459878890
C	-2.698599897920	-2.830715882658	4.078126265272
H	-2.856236560081	-1.987500215533	4.764324999910
H	-1.833670928424	-3.396816608601	4.445580434411
H	-3.580513827740	-3.481881821227	4.143089882326
C	-0.065300763686	2.045782636586	3.451094886399
H	0.871373105733	2.509948222951	3.122051500840
C	-1.215932840987	2.826883184175	2.876019815681
H	-2.195234046908	2.445513764627	3.191059075476
H	-1.192448437470	2.840841029321	1.781341844909
H	-1.160053665375	3.866022788495	3.207339097589
C	-0.104895305180	2.085982836037	4.979283346177
H	-0.097592884861	3.123619175559	5.336941045798
H	0.759814522672	1.571011818246	5.413805187220
H	-1.016552998712	1.603213181174	5.356964633168
C	3.855174371467	3.593580200211	0.512639749795
C	4.573363794484	3.283379675904	-0.663333180549
C	4.539846589549	4.226879565135	-1.732576182587
H	5.076700638536	3.992822020128	-2.650664139830
C	3.834335905345	5.411379949436	-1.644726081296
C	3.185117586922	5.723180436521	-0.463577837323
H	2.646274513789	6.666226659543	-0.376376967155
C	3.187775385476	4.821480562179	0.608341729963
C	5.376088826173	2.023279242922	-0.730593974594
H	4.818255629300	1.252450347237	-0.179616133185
C	5.614805099251	1.515378950417	-2.155594784978
H	4.674609456520	1.435381520685	-2.715058800593
H	6.087275781950	0.526243865913	-2.132320950572
H	6.282931331848	2.183926378936	-2.714185773519
C	6.742840769170	2.201678621885	-0.017695045391
H	7.316984787442	1.266794689084	-0.052153609924
H	6.603863631834	2.481195063511	1.030916546794
H	7.327163903702	2.987809565637	-0.512996892216
C	3.792554292179	6.377379831740	-2.828637327169
H	3.171685956464	7.230322586151	-2.515063502158

C	3.140903178793	5.756980024853	-4.053158506608
H	3.664140736367	4.843213102697	-4.363861045956
H	3.161164621163	6.457029738109	-4.899018660426
H	2.096003795868	5.494459550202	-3.860629123309
C	5.158092701056	6.891679067770	-3.166238192928
H	5.640324146395	7.359809704450	-2.298861379669
H	5.113773491655	7.637533321381	-3.971547207312
H	5.816786702312	6.081146411829	-3.507666882363
C	2.461585523923	5.244281093649	1.875866357296
H	2.549048237961	4.413315047668	2.587127711676
C	0.962738884659	5.491781861092	1.656395520338
H	0.790744287558	6.344185986115	0.986708323185
H	0.470759342738	5.715313101433	2.611489214507
H	0.476988747066	4.615942916492	1.214611303166
C	3.103648446214	6.456080827205	2.496959491625
H	4.166109939786	6.283364828239	2.711015918078
H	2.608307464571	6.720127247554	3.441112256100
H	3.039588750152	7.330703013375	1.834924359703

2Tm

Tm	-0.001875104090	0.000388525378	-0.000133335371
N	2.282943577963	0.266248806692	0.196626874595
N	-0.774448567549	-2.175324347320	-0.226760154778
C	-1.957364475637	-4.229047760659	-3.706358696640
C	-1.630127057738	-2.878844205199	-3.729430506917
C	-1.897756975599	-4.900218149846	-2.492975187199
C	-3.984332043645	-0.181829596899	-3.298151726763
C	-4.763493574274	-5.160710365324	-0.339843484129
C	1.731103332135	-1.246064104138	-4.850966041553
C	-1.250570427344	-2.221893629339	-2.574929592913
C	-1.518937139795	-4.270137324834	-1.316603860908
C	-3.227816358826	-0.139278907884	-1.974004904174
C	-3.829940020189	-4.264384543727	0.469026105559
C	1.439162078749	-7.270678713057	-0.837074617706
C	-0.837559378145	-0.787327498714	-2.570018616895
C	-1.171311576010	-2.884338212137	-1.323542942143
C	1.500411832108	-1.480823383930	-3.359050248332
C	-1.459186598928	-5.037466294271	-0.034248398478
C	-1.767796605435	0.215240937927	-2.215005643821
C	0.889562374103	-5.817425322539	-0.619276483452
C	-2.558951509807	-5.010520919394	0.842555504839
C	0.505828842098	-0.443038279854	-2.845658833520
C	-3.915702766215	0.800464241905	-0.994839773430
C	-0.310756757481	-5.769494706941	0.309880037906
C	-4.569682755369	-3.680800233324	1.671819625474
C	-1.336419509756	1.553832800632	-2.159209646218
C	6.109489036034	-1.289238654883	-4.758656385952
C	-2.491384479045	-5.710369520063	2.043090932462
C	0.892039320464	0.898272694545	-2.754790511783
C	2.830768918294	-1.493386983037	-2.611115505832
C	1.981804985194	-4.897621274404	-0.433170564699
C	-0.295614952105	-6.457476502483	1.523326383611
C	1.221912538617	3.609903741475	-3.934649151593

C	-0.015494215419	1.923565826043	-2.456204161077
C	-1.378219268548	-6.432876722980	2.400183228471
C	-1.343949822448	-8.683315637237	3.514834071433
C	-0.656801755686	4.421829733484	-2.536732376144
C	0.427453975489	3.375627068596	-2.579468481127
C	7.060511724091	-0.457287471203	-3.906062558062
C	-1.379643465663	-7.176430354815	3.733727892517
C	8.419944739517	-1.137876056591	-3.777439549299
C	6.445763378542	-0.147498019803	-2.549932466985
C	5.227790024198	-3.379700431088	-0.044373278093
C	6.219459092144	-1.142717154073	-1.603610723968
C	-0.332282785198	-6.674372473750	4.685174574601
C	6.061788840782	1.145849740983	-2.238103135057
C	0.991087138494	-3.011341996496	2.730104539803
C	4.278424198616	3.546810298091	-1.832622100275
C	5.599545328924	-0.878171757074	-0.383640601481
C	5.355014429469	-2.000194478551	0.616920604582
C	5.450492345379	1.459087373045	-1.016732533884
C	0.050035146833	4.008079958112	0.896445228199
C	5.204606797253	0.441642858087	-0.088393649488
C	3.072060035402	0.643832323668	1.243752485229
C	1.341611999143	-1.626661323970	3.262425975807
C	5.109768465745	2.912626030718	-0.715359113063
C	0.619805719970	3.255167867626	1.979106452152
C	4.497379125465	0.759387065072	1.192320676307
C	6.446897887881	-2.046693061038	1.675005785449
C	2.446968787948	0.964866748649	2.481440602930
C	6.374927557082	3.714466091500	-0.447491678242
C	1.303481514966	-1.617070430962	4.791559372924
C	0.669580499064	4.010416968725	3.291179762780
C	5.197217394699	1.206468904045	2.300799830237
C	3.173494718774	1.394785047718	3.570594606547
C	4.561593029147	1.527119814785	3.498266177893
C	-3.332514233147	0.075306093064	2.606003167013
C	-4.152924310791	1.361205604560	2.606248976175
C	-1.833615958094	0.345967468305	2.442090710047
C	-3.616496457486	-0.662010493769	3.918100015633
C	-0.944691778763	-0.701749137334	2.707201669583
C	-1.289118280850	1.600111350720	2.148797473906
C	0.436548205095	-0.508103445502	2.768770859050
C	0.100905084100	1.840648654414	2.178277205956
C	0.965550630507	0.784257111613	2.507724249069
H	-2.252047262904	-4.747390901530	-4.616043166481
H	-1.654096374414	-2.325850696717	-4.668812709599
H	-2.141165456046	-5.962320701115	-2.450382872801
H	-3.545613596448	-0.916595977642	-3.980780872065
H	-4.276093908125	-5.515373124451	-1.254158912078
H	-5.033745110705	-0.458135130592	-3.133227963264
H	-5.672695214338	-4.616731723485	-0.629332514175
H	-3.963260686595	0.800979578569	-3.788030749810
H	0.788952349523	-1.274325787561	-5.411584475791
H	-5.063421484846	-6.037501402034	0.249800631790
H	2.396383727870	-2.015841474217	-5.261899704098
H	0.625512235590	-7.972546769133	-1.053163109897
H	2.198811284755	-0.267865146249	-5.025908460420

H	2.137721566904	-7.269968209848	-1.682151629519
H	0.456855848646	-5.564275537805	-1.599658762182
H	-3.233832452073	-1.151691789252	-1.546404751106
H	1.030023636162	-2.462832363241	-3.232268775242
H	-3.529347242850	-3.427760012165	-0.177459814995
H	-4.898449370821	0.404954750298	-0.711911218260
H	6.533184096760	-1.474686021758	-5.754617576096
H	-5.380790204876	-3.023603644206	1.332857378776
H	0.557961614306	3.427525758693	-4.788414262297
H	-4.077130278956	1.798341265425	-1.423745662667
H	-5.024989807258	-4.462490580883	2.293619467219
H	1.981943748413	-7.636820734937	0.045564032756
H	5.144604896445	-0.784244261946	-4.885620103952
H	-2.165756006147	-9.000417860625	2.862482762705
H	3.487165847502	-2.263709849234	-3.031623818693
H	5.915087582578	-2.263354308814	-4.290476757173
H	2.714694511990	-4.999368324662	-1.243244471935
H	-1.351424083408	4.304582839189	-3.379599960955
H	-2.062813043387	2.331162957838	-1.935345555450
H	-3.344041789403	-5.693648392406	2.721197664088
H	-3.321879262063	0.917798127033	-0.081093869027
H	1.646403866693	-3.851801885144	-0.429788721682
H	1.927672716948	1.154083491991	-2.968408475288
H	2.092733856682	2.955325966038	-4.027222595174
H	-0.403263468834	-8.995506580498	3.040877407416
H	-3.897888355968	-3.092996401554	2.307090124557
H	3.369339462298	-0.539740745651	-2.691527915013
H	-0.805981961075	-2.781043387940	0.592542391326
H	0.595670371524	-7.025321809037	1.792873076325
H	1.570883597212	4.647740816278	-3.972969621170
H	2.688296058018	-1.719595921945	-1.549660387776
H	2.523380173343	-5.061156338346	0.511342882162
H	-1.431911427381	-9.222938251544	4.467397018318
H	8.871309368432	-1.297974090054	-4.765289424718
H	7.212772863622	0.507021038285	-4.413325154354
H	-0.213322029578	5.421449869385	-2.605871794962
H	-3.657778979947	-0.562999490134	1.770380070259
H	-2.357370240261	-6.951409422425	4.188569951005
H	-1.248802873726	4.393125510825	-1.613519156791
H	8.327752091997	-2.118572417218	-3.292586079787
H	4.500586368974	-3.371355296360	-0.862465710608
H	1.146347990680	3.574149216726	-1.770615930668
H	-4.047133664199	1.935075825866	1.678887511931
H	-5.218273256454	1.130177237809	2.725110059987
H	6.515896199169	-2.164545419899	-1.833223771708
H	1.029584891733	-3.040561939533	1.637096054752
H	2.871775128632	0.098399706945	-0.617116271189
H	6.190845565077	-3.725142298906	-0.442501280653
H	3.372290787988	2.958644993432	-2.011155855292
H	-4.693276647435	-0.843338199349	4.020037397796
H	6.247529465987	1.939821788631	-2.962525241506
H	9.110888766331	-0.533178034786	-3.177925252793
H	4.898442192335	-4.118473503851	0.695627266316
H	-1.340887691793	-1.691959681148	2.916699538759
H	0.682785120929	-6.849253103790	4.301490382583

H	-3.114074716474	-1.632450934698	3.980551946301
H	-0.005827572989	-3.346828181693	3.044718097918
H	4.835625623741	3.609073856893	-2.776150963037
H	0.041571352992	3.425026648727	-0.030391746682
H	-0.436134681211	-5.595475897422	4.853575994974
H	-1.952877855444	2.438670254016	1.949484792537
H	-0.406688987183	-7.178858593544	5.657890031647
H	-3.855096561714	2.008364564699	3.441591850435
H	1.708865460724	-3.748985943469	3.108154883586
H	-0.989358647223	4.325262559631	1.081303074389
H	3.974956508723	4.566115317856	-1.559524471673
H	4.402514984496	-1.774502413281	1.118307309749
H	-3.287557909245	-0.058116504376	4.773596104946
H	0.622134861674	4.924303896362	0.702330166770
H	2.361701248477	-1.381083140759	2.942016683008
H	4.501265492502	2.920232128517	0.197674700872
H	1.679965225401	3.090950268114	1.717747640169
H	7.424429484197	-2.234542446281	1.210691289998
H	7.032443998657	3.718792357786	-1.327352616891
H	6.253023366440	-2.851749954114	2.396375005018
H	0.289704443459	-1.835031679014	5.153644334207
H	6.508287231074	-1.105017321601	2.230291506508
H	1.981682074951	-2.377859162147	5.198629985435
H	6.133909847123	4.757027575040	-0.200052768975
H	-0.339935774168	4.196782688882	3.686349674187
H	6.941126099378	3.289018681604	0.389644823913
H	1.605986009289	-0.642859734386	5.192538900686
H	1.160324868545	4.982565800948	3.153576988216
H	6.279891000277	1.307835742485	2.221831569262
H	1.229994828036	3.453391866562	4.048316864731
H	2.654506163382	1.623492994890	4.501444126929
H	5.133142226979	1.870781040400	4.357331853899

2Yb (using Yb1)

Yb	-0.000000455918	-0.003804854980	0.000000582130
N	1.820780789905	1.353792144225	0.324477533217
H	2.069884646297	2.015999468894	-0.409717869817
N	-1.820777864510	1.353798144406	-0.324472803667
H	-2.070767967058	2.016347180283	0.409213727917
C	-2.670456337761	1.522500734851	-1.383295026956
C	-2.473240663239	0.703001976813	-2.542354320329
C	-3.339804703620	0.792904620391	-3.623512522096
H	-3.165538610325	0.163343618047	-4.496096809376
C	-4.388223192288	1.688006099652	-3.622365204050
H	-5.060012372734	1.745018692369	-4.476815452932
C	-4.552178773180	2.559004919458	-2.545113974217
H	-5.329794655727	3.321399626797	-2.571915508109
C	-3.714802492516	2.494702305806	-1.454918608195
C	-1.228489442629	-0.114999740290	-2.573709505056
C	-1.198978358021	-1.492200281962	-2.192928510384
C	0.021168402414	-2.162302048004	-2.161527487450
H	0.047046057211	-3.218849721898	-1.897873783330
C	1.223761521956	-1.538803276029	-2.520900491665

C	1.194818456246	-0.188902778725	-2.868691658426
H	2.119585965364	0.314092584808	-3.139407346452
C	-0.016029562331	0.535798980651	-2.905564880934
C	-2.483331978207	-2.231398882605	-1.875865686004
H	-3.183490427875	-1.483741663856	-1.475156686236
C	-2.302799765186	-3.343400497921	-0.837141219428
H	-3.280156466751	-3.707485729707	-0.500261914027
H	-1.749776031601	-2.988557091778	0.039468049911
H	-1.759048907017	-4.201329303356	-1.253502345413
C	-3.088161911099	-2.809896369315	-3.141008709633
H	-3.305904868377	-2.028776638317	-3.876219404148
H	-4.027239641973	-3.332440118757	-2.917724366993
H	-2.400892843119	-3.530772208501	-3.604266338336
C	2.497861972957	-2.347104919975	-2.637342832182
H	2.325865135562	-3.296509894364	-2.110368001986
C	3.705220731676	-1.657707477879	-1.991656881392
H	4.567779690280	-2.334809255441	-1.972673297886
H	3.488462351418	-1.338135659062	-0.966319738882
H	3.997399496750	-0.767426220038	-2.557218496295
C	2.781895119053	-2.678103410013	-4.096122700676
H	1.941306690755	-3.210465400712	-4.557493141957
H	3.676178060004	-3.308174168505	-4.183336859728
H	2.956483022510	-1.762580111874	-4.676464474814
C	-0.021248737988	1.978899638449	-3.401711726146
H	-0.890308439390	2.464935871345	-2.942267139398
C	1.207302582883	2.766297408228	-3.032650830502
H	1.352529015969	2.818156669474	-1.949648550328
H	1.105786746918	3.794059193298	-3.388722468327
H	2.125764390053	2.367148412712	-3.481014204840
C	-0.251371870083	1.978901968602	-4.928965552520
H	0.577052765865	1.475161779554	-5.444742667619
H	-0.312103450985	3.009169682136	-5.301306399987
H	-1.183875823381	1.466472747269	-5.185288355473
C	-3.792252973649	3.520000978107	-0.357698306365
C	-3.134800087270	4.755800255274	-0.516857318891
C	-3.132754117195	5.658898833363	0.566523398815
H	-2.600261080485	6.604562444792	0.447037116396
C	-3.771652519610	5.414598168926	1.761539209796
C	-4.446354622221	4.210998966817	1.882792477411
H	-4.971353958976	3.992184890928	2.811895023072
C	-4.481196426732	3.256500362430	0.857689703889
C	-2.454824217158	5.122200973958	-1.804793008062
H	-2.566795827186	4.278747520036	-2.495057334291
C	-0.964961867605	5.398598636049	-1.633246638011
H	-0.446739681360	4.537518040300	-1.197943920892
H	-0.790090079523	6.260012262034	-0.976520602801
H	-0.501156439270	5.622382181953	-2.602384842735
C	-3.138364898580	6.343502757353	-2.428544432290
H	-4.207175623469	6.159650054686	-2.592895290323
H	-2.681696441375	6.593043816759	-3.395440024517
H	-3.046381781416	7.221656556224	-1.776337459576
C	-3.706413372610	6.414196582104	2.898284620691
H	-3.141950345407	7.285137265351	2.535525595974
C	-2.964599947954	5.832493943918	4.102542649211
H	-3.443567373269	4.909599436955	4.452982279484

H	-2.955931810467	6.546020872643	4.937108609523
H	-1.925985118729	5.597679738819	3.848153749493
C	-5.098783061153	6.883398019960	3.306315433099
H	-5.696236522463	6.049676412772	3.697880045794
H	-5.640962428747	7.309019754511	2.453305320486
H	-5.040286156317	7.647872008542	4.092062453560
C	-5.283215132103	1.962801323070	0.993383953174
H	-4.713052801332	1.173685907747	0.483657674700
C	-5.514411110176	1.541799732658	2.444899923087
H	-4.573056696704	1.485123997812	3.005841952757
H	-5.997790295570	0.558086891748	2.482623481966
H	-6.174457506463	2.246297838744	2.967381722397
C	-6.638589279907	2.113904179587	0.279865267299
H	-7.223186199642	1.187512276659	0.358650940082
H	-6.503795028237	2.340512914694	-0.781656841854
H	-7.218371926255	2.926567026912	0.736426517621
C	2.670459736705	1.522489555104	1.383290197864
C	2.473241750558	0.702988312769	2.542357339343
C	3.339806048085	0.792885669262	3.623515774871
H	3.165525366263	0.163325658254	4.496095378198
C	4.388227066362	1.687984188971	3.622370805234
H	5.060014138193	1.744991304734	4.476823331157
C	4.552185106925	2.558985372120	2.545121860521
H	5.329806761462	3.321374918805	2.571923715473
C	3.714808640232	2.494687986950	1.454926329242
C	1.228488212634	-0.115009969463	2.573700378056
C	1.198973237398	-1.492209426324	2.192925769091
C	-0.021175417289	-2.162307661673	2.161522990511
H	-0.047052292868	-3.218857025552	1.897874388628
C	-1.223766774655	-1.538806434142	2.520897632852
C	-1.194819895692	-0.188906928750	2.868682340348
H	-2.119589518124	0.314083302245	3.139388268361
C	0.016030175086	0.535791310887	2.905567463346
C	2.483324768177	-2.231410825235	1.875861004866
H	3.183466479451	-1.483749171755	1.475128661186
C	2.302789414720	-3.343409206435	0.837133621849
H	3.280144235832	-3.707481010134	0.500234526130
H	1.749744606244	-2.988569064253	-0.039464013756
H	1.759055190717	-4.201347302118	1.253498659456
C	3.088253054351	-2.809913339692	3.140982490117
H	3.306021809547	-2.028791395326	3.876182710781
H	4.027329396777	-3.332426137658	2.917624184569
H	2.401031204120	-3.530812093684	3.604273963985
C	-2.497869509128	-2.347104783956	2.637337854475
H	-2.325877311768	-3.296509983592	2.110361518295
C	-3.705226321588	-1.657702238543	1.991653713232
H	-4.567785942377	-2.334803115088	1.972661556554
H	-3.488463942959	-1.338121208692	0.966320239010
H	-3.997405185530	-0.767425790336	2.557222772158
C	-2.781903587563	-2.678106302798	4.096116854196
H	-1.941317584562	-3.210474860752	4.557483833936
H	-3.676190470436	-3.308171368769	4.183332013397
H	-2.956482893538	-1.762583409900	4.676461582472
C	0.021253430330	1.978890652204	3.401718094746
H	0.890291053961	2.464908428661	2.942210035056

C	-1.207295663430	2.766292865250	3.032659265191
H	-1.352421103642	2.818239320973	1.949642266905
H	-1.105854698913	3.793996537300	3.388927130988
H	-2.125784245378	2.367049115119	3.480883734446
C	0.251356536993	1.978888325677	4.928871921998
H	-0.577133917712	1.475276725288	5.444673604643
H	0.312230050450	3.009149418753	5.301212008253
H	1.183781147377	1.466336244331	5.185248485429
C	3.792262021564	3.519989318521	0.357708717100
C	3.134812634616	4.755790039842	0.516870970527
C	3.132769223493	5.658891464032	-0.566517375212
H	2.600277858031	6.604556609457	-0.447029250867
C	3.771666938237	5.414592127998	-1.761523825424
C	4.446365628547	4.210991333494	-1.882790248872
H	4.971367652184	3.992184119837	-2.811893540760
C	4.481204727660	3.256489937980	-0.857689981692
C	2.454837796535	5.122189309083	1.804797620748
H	2.566802464430	4.278737136020	2.495065150674
C	0.964976232164	5.398591644354	1.633261981145
H	0.446753132337	4.537522082906	1.197938559875
H	0.790106278400	6.260026594530	0.976562042624
H	0.501167289184	5.622339128401	2.602406323070
C	3.138381947821	6.343487517783	2.428562238218
H	4.207189513627	6.159624622848	2.592922640892
H	2.681706144629	6.593031615008	3.395453888708
H	3.046413285928	7.221642229706	1.776354196553
C	3.706430624248	6.414193714732	-2.898266608226
H	3.141968477926	7.285135923460	-2.535509263129
C	2.964615552817	5.832496348658	-4.102536168717
H	3.443605820626	4.909621570623	-4.452997907553
H	2.955924148167	6.546038710253	-4.937088953520
H	1.926008017243	5.597650235880	-3.848147765513
C	5.098801641474	6.883392283087	-3.306296186296
H	5.696254471801	6.049667452051	-3.697854886457
H	5.640977334569	7.309013557286	-2.453283617075
H	5.040312750043	7.647865024687	-4.092045615935
C	5.283219772906	1.962788980747	-0.993377626590
H	4.713073322721	1.173678564778	-0.483631186427
C	5.514414558349	1.541790550221	-2.444904702632
H	4.573058565834	1.485132120703	-3.005847994737
H	5.997783267222	0.558072843472	-2.482639579820
H	6.174465752038	2.246289359081	-2.967378643070
C	6.638694343172	2.113886115317	-0.279878566927
H	7.223262099870	1.187479879565	-0.358692174255
H	6.503930128328	2.340502274446	0.781645655200
H	7.218453998891	2.926541930267	-0.736476811035

Full optimisations

2Sc

Sc	0.057028509968	-0.041604336779	0.020704901634
N	-0.356052605679	1.936565430051	0.553891561379
H	-0.600345750514	2.160943354432	1.527618776164
N	-1.147425567571	-1.138852888880	1.304986658668
H	-2.160276772097	-1.022177982674	1.159585525935
C	-0.574137089904	3.014174362364	-0.278695494258
C	-0.456501767185	2.784434763972	-1.686444842291
C	-0.586308624399	3.836136303633	-2.601673330230
H	-0.467988856793	3.631110989787	-3.677540904309
C	-0.878878644406	5.135305417694	-2.151242401437
H	-0.986177241981	5.962604778897	-2.867651089843
C	-1.067861016084	5.357275086459	-0.776871357708
H	-1.343414096381	6.360648519181	-0.414957150022
C	-0.930728795218	4.324308759096	0.168673526745
C	-0.215099410890	1.353547342032	-2.020049696245
C	-1.343691324212	0.418497803327	-1.903326148462
C	-1.057796689555	-0.964202287199	-2.067917213187
H	-1.893677468520	-1.678267806203	-2.062777247929
C	0.250276567710	-1.468516448435	-2.152160297769
C	1.348172872730	-0.537509429857	-2.107111023485
H	2.372139099549	-0.913544179983	-2.238571844214
C	1.103964726074	0.865667383290	-2.151823513553
C	-2.770227188001	0.948205600369	-1.898808195535
H	-2.772228707375	1.849820241843	-1.250345383948
C	-3.800015290683	-0.037461855521	-1.338746864127
H	-4.790532702010	0.451548239740	-1.251491633419
H	-3.528794420290	-0.415307758212	-0.333902898441
H	-3.939188504891	-0.915549192713	-2.002809220791
C	-3.176482227707	1.398222339987	-3.316208694652
H	-4.195523085104	1.837092618362	-3.312353079828
H	-3.177083798417	0.534730763491	-4.014021498177
H	-2.479868927213	2.160977463002	-3.713484127360
C	0.512674035166	-2.960405987942	-2.304610250489
H	1.613022172597	-3.081173613569	-2.387228259714
C	0.040540395047	-3.765289669068	-1.085816664594
H	-1.061132913183	-3.730790069868	-0.993704544371
H	0.454999196438	-3.370431425339	-0.138044490700
H	0.337956876944	-4.830520939445	-1.171781654511
C	-0.109793458651	-3.500857100635	-3.603647497189
H	-1.216996355514	-3.430912439119	-3.576016011297
H	0.151925401520	-4.569174932514	-3.748120039243
H	0.243024818353	-2.935066701401	-4.488946690171
C	2.284709321970	1.819858021597	-2.234025224576
H	1.882653876764	2.839229572554	-2.066273937325
C	3.327080484991	1.548368515600	-1.142830834023
H	3.816510645534	0.559400990389	-1.261144613703
H	2.871290851300	1.576318661288	-0.131540230845
H	4.123282770271	2.318621380275	-1.173004542038
C	2.903010020253	1.785366370971	-3.641854916254
H	2.149162655185	2.028447268069	-4.417294276243
H	3.318577681442	0.783047775502	-3.876546037588

H	3.728820127058	2.521248175166	-3.722652029930
C	-1.216943196145	4.551637271889	1.618376239128
C	-2.484531020449	4.169034256063	2.138383610157
C	-2.753093660107	4.376164580324	3.502263841154
H	-3.740894020650	4.092725620112	3.903363123562
C	-1.813573649361	4.959725437305	4.370389609673
C	-0.568269128585	5.332962104238	3.834929264432
H	0.180014755303	5.797328700807	4.491821495832
C	-0.249799198319	5.140640417931	2.477009915497
C	-3.564586596349	3.584148516395	1.232890756389
H	-3.131788576918	3.520434464141	0.214115635504
C	-3.968910790510	2.163535476720	1.650828793896
H	-4.693161803412	1.731248571311	0.933030933765
H	-4.452454922866	2.152410771196	2.648763037306
H	-3.091605532650	1.487278460210	1.689301827103
C	-4.785964255337	4.513782291351	1.144582690479
H	-5.538483137585	4.105837906649	0.438229396703
H	-4.495750248895	5.524192228590	0.793061347596
H	-5.281564510814	4.629724777929	2.131227469156
C	-2.170516043899	5.149765170681	5.841676442150
H	-3.241957674321	5.451626587576	5.863619059437
C	-2.059307828591	3.820989548817	6.613477176825
H	-2.674309500712	3.026522684721	6.145550331361
H	-2.396395175995	3.942251254239	7.663749367858
H	-1.008849226850	3.464671835669	6.629707149317
C	-1.366654091700	6.252123623539	6.540286602607
H	-1.416260073736	7.210787182699	5.986221566129
H	-0.296792335026	5.974383781602	6.641601542540
H	-1.754364246201	6.425437586171	7.564342360296
C	1.127334897649	5.519647650467	1.936763241995
H	0.989285486222	5.779766227170	0.867021504253
C	2.080508613815	4.310397807355	1.983638105328
H	1.660383719182	3.435341567794	1.449527585679
H	2.266972553835	4.005633852608	3.034792385668
H	3.059805503854	4.560157884114	1.524252860083
C	1.753316833992	6.735208869691	2.632106072804
H	1.062305973330	7.601991478921	2.643768007899
H	2.681024906488	7.041786466539	2.107659142101
H	2.036129442749	6.513813163516	3.682529125648
C	-0.914964730711	-2.181945500632	2.171627043985
C	0.406704049686	-2.422712703080	2.676609051952
C	0.635010216177	-3.549356245772	3.492339060445
H	1.648605191991	-3.710133888057	3.888449203529
C	-0.396167779369	-4.423876544290	3.858827914260
H	-0.188813505344	-5.294933675797	4.496996887916
C	-1.707839359430	-4.128731585355	3.453675921930
H	-2.545142739246	-4.757846951130	3.794255752960
C	-1.987114928686	-3.018572537981	2.643131194546
C	1.521076990121	-1.430008441853	2.495301488351
C	1.436071532356	-0.151357923079	3.144870159059
C	2.570158899795	0.681887607263	3.180097448967
H	2.492743812511	1.637592727210	3.712628691109
C	3.811624534151	0.298433822051	2.645266759448
C	3.870402834058	-0.936673417751	1.982351427151
H	4.834957931732	-1.261939923897	1.558570066441

C	2.765674332711	-1.806053848457	1.895977618848
C	0.212460878318	0.247450405094	3.969975976502
H	-0.682660150490	-0.089876328519	3.410736557047
C	0.065891280708	1.756616784264	4.196444113095
H	0.176005370936	2.347771885716	3.267698096182
H	0.812215331251	2.148135227226	4.918860198620
H	-0.932684880656	1.982049609621	4.613973452231
C	0.235240845269	-0.480666143301	5.329777706169
H	-0.658077351205	-0.208571805307	5.928250566044
H	1.134040820770	-0.185163249892	5.910348554378
H	0.245181798419	-1.580621410064	5.211362340261
C	5.078494520162	1.131508894190	2.821580704362
H	5.892548493879	0.398251769316	3.020435285846
C	5.459040280006	1.889147386711	1.538155932530
H	5.552119726278	1.205019706889	0.672473743973
H	6.427625785592	2.414847254436	1.666163118421
H	4.688976733636	2.646821714263	1.289449060922
C	5.022943367921	2.091215277193	4.016511963495
H	4.739992925508	1.569272766773	4.952378812126
H	4.291918647364	2.908613755076	3.847453105469
H	6.011538000861	2.567373283396	4.173794497504
C	2.985684190457	-3.171915651297	1.249303445003
H	1.998886249126	-3.673769573925	1.194470345205
C	3.539977766805	-3.047859366815	-0.178170954203
H	2.925112762549	-2.355865576050	-0.782815546354
H	3.554509889366	-4.035335879427	-0.683241649938
H	4.579398777428	-2.659094851114	-0.181441718426
C	3.911902104496	-4.061253246342	2.098974988883
H	3.525443054779	-4.200601485131	3.126949991000
H	4.924820934241	-3.615397131108	2.181840489309
H	4.021952544451	-5.064086453663	1.637230466925
C	-3.397189311210	-2.607296946590	2.341364658528
C	-4.099424273325	-3.119206289003	1.218795278290
C	-5.400314998283	-2.650685221802	0.952815070703
H	-5.950851548254	-3.045781258777	0.084262421060
C	-6.026768163187	-1.689138808931	1.761214090256
C	-5.324530052612	-1.211336184572	2.882840901452
H	-5.806233347003	-0.474015635089	3.543563208075
C	-4.028848403112	-1.655811058235	3.195658829840
C	-3.482586023847	-4.216390496008	0.360459915011
H	-2.384506561226	-4.150872280152	0.509472188880
C	-3.771451268427	-4.068814353717	-1.139138064020
H	-3.498847899515	-3.060454233095	-1.508204473089
H	-4.843542323078	-4.230100970478	-1.374593986339
H	-3.196133750759	-4.816655842420	-1.722060815250
C	-3.925450547379	-5.602702175031	0.863916711471
H	-3.650735579331	-5.751353018642	1.926089905555
H	-3.449051894932	-6.410462736324	0.270323621165
H	-5.026303098987	-5.717833257739	0.779816976689
C	-3.353216088718	-1.212205901399	4.490812367111
H	-2.266449668026	-1.389559965599	4.368031405631
C	-3.541380161219	0.269711077402	4.835952564900
H	-3.158207271628	0.927934117814	4.033952732505
H	-2.993208126290	0.515158887706	5.768355730836
H	-4.605869709818	0.530498377385	5.012115764428

C	-3.824481924142	-2.109491667581	5.650770524519
H	-4.916063438783	-1.996255515287	5.818781333071
H	-3.301050984584	-1.842839868390	6.592385956550
H	-3.624047439259	-3.177933258718	5.438981917809
C	-7.427176030469	-1.188584969665	1.433906694156
H	-7.777961918726	-1.772555013882	0.554709208050
C	-7.418132450299	0.295838769866	1.030433040416
H	-6.748144997956	0.472098358900	0.165169805368
H	-7.060798156326	0.935803900510	1.863475492507
H	-8.436277927482	0.639126595383	0.753588518365
C	-8.412938140242	-1.446961126624	2.585372687293
H	-9.441080468060	-1.140943525347	2.302172669981
H	-8.133317569404	-0.871127041079	3.491934256261
H	-8.435182511702	-2.519563326507	2.863489886671

2Y

Y	-0.000178329919	0.085086715049	-0.000021429840
N	1.773403880375	-1.316670971398	0.305462892140
H	2.019044967564	-2.006452938515	-0.415023995780
N	-1.773937773484	-1.316499531689	-0.305390673543
H	-2.019629399329	-2.006221810159	0.415128693694
C	-2.619622862883	-1.444770447555	-1.380070220094
C	-2.423292634053	-0.590839808785	-2.514964961138
C	-3.279904577892	-0.643751608613	-3.620972118627
H	-3.091388319865	0.022166226358	-4.477955189780
C	-4.360752490759	-1.541839884971	-3.644601103823
H	-5.039252546945	-1.578669352401	-4.508879936591
C	-4.533850463966	-2.423701243747	-2.563576199273
H	-5.340992244550	-3.172970107113	-2.593430791854
C	-3.680928976514	-2.406970521338	-1.446457033213
C	-1.205782761808	0.259593562700	-2.429354229572
C	-1.233792537834	1.585396836157	-1.932381259576
C	0.006977326490	2.248538505605	-1.675407546925
H	-0.025621720467	3.281610549608	-1.299063132091
C	1.259776053852	1.712854134638	-2.128857768480
C	1.257379337677	0.394358193792	-2.605630406754
H	2.205329117764	-0.064991594053	-2.917373335032
C	0.067996854625	-0.377663119762	-2.712154321314
C	-2.502331159876	2.400519252086	-1.688243359481
H	-2.194072394335	3.192522277102	-0.968128544170
C	-3.704254753729	1.674236762862	-1.064800245949
H	-4.264458416533	1.077335369424	-1.806872576581
H	-4.401421935707	2.419877285405	-0.631398727153
H	-3.388707270664	0.985845300492	-0.257485746190
C	-2.903219071053	3.118073219469	-2.991965659819
H	-2.059097660184	3.699743541168	-3.413810213199
H	-3.747680200469	3.813964222429	-2.809274397661
H	-3.228019783159	2.381987786937	-3.754576448314
C	2.487967677100	2.608915410850	-2.143816796632
H	2.589325940662	3.039835757298	-1.121146687283
C	3.789518519833	1.879748540809	-2.482703976687
H	3.967949014348	1.015617237829	-1.816049201440
H	4.653573387574	2.566309902178	-2.383353464085

H	3.782722045535	1.506512977402	-3.527661399545
C	2.269125675264	3.790777390211	-3.111443627803
H	2.164243477889	3.424155999248	-4.153570246310
H	3.131220740567	4.487783446297	-3.078103294408
H	1.355830620073	4.367280590962	-2.865094942798
C	0.100375642687	-1.756419531565	-3.364061896686
H	-0.853103521115	-2.257524384719	-3.102115016189
C	1.238770772951	-2.668817989028	-2.896315124392
H	2.243512948942	-2.224698708401	-3.048127511711
H	1.222944261209	-3.620687500430	-3.463224457497
H	1.137488126503	-2.934756299486	-1.826648065692
C	0.117957955542	-1.587146061608	-4.896439649368
H	-0.744291922697	-0.983259920943	-5.242028374200
H	0.070363112433	-2.573469209127	-5.402352368804
H	1.046625217317	-1.076996297076	-5.228867511550
C	-3.764468442702	-3.444346066624	-0.371480420623
C	-4.469235566087	-3.200431004089	0.837883215548
C	-4.485563585930	-4.199299404565	1.829122386134
H	-5.034635548138	-4.025219916457	2.767315831307
C	-3.815023061689	-5.423356425978	1.667534109789
C	-3.109649682753	-5.637836482918	0.470865258014
H	-2.573123143640	-6.588982961985	0.325331003258
C	-3.068813599982	-4.673040461553	-0.550992606350
C	-5.230987987040	-1.891339186461	1.028102687324
H	-4.655243324834	-1.109121880937	0.486188324348
C	-5.357817958353	-1.455609829567	2.493329340104
H	-5.812742720106	-0.447194648152	2.559113362780
H	-4.372237362082	-1.420676068132	3.000369993553
H	-6.009416901277	-2.141145920112	3.073395164382
C	-6.621927404119	-1.970853099930	0.371696148782
H	-7.233339129715	-2.764409534592	0.849700878181
H	-6.544370713899	-2.201130439685	-0.707787223942
H	-7.164223281341	-1.008256757177	0.477872435965
C	-3.856494443910	-6.484186295565	2.759101103750
H	-4.473589004339	-6.069228555987	3.586222223006
C	-2.457341449945	-6.777282653950	3.325646833949
H	-1.786654541892	-7.193993250445	2.546001163924
H	-2.509368694918	-7.514689379476	4.153045529499
H	-1.980759479773	-5.855197038430	3.714563002586
C	-4.541006578483	-7.771825099995	2.269934793716
H	-5.560993398432	-7.565077923546	1.888880584793
H	-4.621070438571	-8.515623834010	3.089485380472
H	-3.965271805590	-8.242857452939	1.446210471146
C	-2.332685900777	-4.972930228692	-1.854035912403
H	-2.233822337889	-4.010960214845	-2.397013113212
C	-0.916871880108	-5.525621901086	-1.647133232273
H	-0.917803512655	-6.509116429478	-1.132403242296
H	-0.294661358439	-4.833809792708	-1.047619799863
H	-0.412570748352	-5.669633355642	-2.623733184515
C	-3.174865753883	-5.909133243189	-2.738622735905
H	-2.670127771876	-6.097203738149	-3.708982091432
H	-4.171354196609	-5.472563892218	-2.948783186749
H	-3.334408910006	-6.888903892815	-2.241131694365
C	2.619255390767	-1.444757083855	1.380021501431
C	2.423056157402	-0.590692177103	2.514836446764

C	3.279963486949	-0.643301268939	3.620633180372
H	3.091612444203	0.022774931032	4.477529089319
C	4.360927180333	-1.541257994357	3.644146665938
H	5.039659813377	-1.577838946764	4.508253940422
C	4.533917564161	-2.423232792288	2.563193726190
H	5.341190271408	-3.172364773819	2.592948635425
C	3.680740100719	-2.406763455439	1.446268647178
C	1.205582905219	0.259807836947	2.429234578391
C	1.233789296003	1.585500928468	1.932044839286
C	-0.006867972319	2.248842635416	1.675014529519
H	0.025916740191	3.281852997706	1.298511183909
C	-1.259681897187	1.713524169474	2.128878566731
C	-1.257512374074	0.395062399356	2.605742753995
H	-2.205469240413	-0.063917412645	2.918017090517
C	-0.068285414388	-0.377197104173	2.712224218663
C	2.502615402193	2.400062750349	1.687721019724
H	2.195012300286	3.191359161198	0.966554242496
C	3.704615272911	1.672540688741	1.065850039639
H	4.263800156600	1.075840514119	1.808879160457
H	4.402638409958	2.417394366165	0.632481108432
H	3.389314260753	0.983733458989	0.258782919848
C	2.902983228675	3.118940395538	2.990873286245
H	2.058758505613	3.701222964002	3.411664811472
H	3.747663506189	3.814479724511	2.807854933429
H	3.227270065569	2.383619238466	3.754434970220
C	-2.487365530957	2.610241834469	2.145156942620
H	-2.590519792537	3.039943193223	1.122150150159
C	-3.788621566997	1.882262051091	2.487733774167
H	-3.968612687623	1.016946346825	1.823054891836
H	-4.652556908149	2.569007745968	2.388620660013
H	-3.780140906458	1.511036605033	3.533392928950
C	-2.265820691819	3.793221385173	3.110793267600
H	-2.159165438004	3.427897980481	4.153195206976
H	-3.127488611300	4.490792068659	3.078210657638
H	-1.352590575137	4.368747278372	2.861906429661
C	-0.100879853457	-1.755781911097	3.364496857350
H	0.852392001992	-2.257218960027	3.102445433427
C	-1.239657873628	-2.667997382075	2.897329596375
H	-2.244231060830	-2.223484215460	3.049124954805
H	-1.224058020521	-3.619633065030	3.464638660943
H	-1.138636191254	-2.934433800220	1.827766930000
C	-0.117988340385	-1.586099182760	4.896832694820
H	0.744536886779	-0.982373977425	5.242019544089
H	-0.070538074037	-2.572302131000	5.402992677772
H	-1.046410578367	-1.075586645747	5.229387713556
C	3.764318343795	-3.444091769123	0.371249273420
C	4.468341307345	-3.199786610759	-0.838470731177
C	4.484505815073	-4.198536192686	-1.829832033656
H	5.032807433179	-4.024048834392	-2.768404841367
C	3.814510388305	-5.422862523203	-1.668010452278
C	3.110039244223	-5.637817428707	-0.470895141700
H	2.573944929351	-6.589180141827	-0.325171448095
C	3.069354662785	-4.673129309093	0.551068204548
C	5.229082939169	-1.890182496229	-1.029169356459
H	4.653133699109	-1.108344172087	-0.486935832907

C	5.354798669654	-1.454411138852	-2.494481977583
H	5.809058788868	-0.445712710628	-2.560553329192
H	4.368918511042	-1.420107101650	-3.000970437299
H	6.006502169964	-2.139552062971	-3.074901109147
C	6.620445019322	-1.968762610651	-0.373549164123
H	7.232118466220	-2.761917264453	-0.851886754830
H	6.543648963045	-2.199080345567	0.705981898909
H	7.162038195861	-1.005806148376	-0.480040069555
C	3.855561121682	-6.483473209717	-2.759804180345
H	4.471972395157	-6.068180526533	-3.587266917754
C	2.456105161894	-6.776871053818	-3.325454568465
H	1.786035940887	-7.193829084558	-2.545406959609
H	2.507777764495	-7.514181384451	-4.152961787179
H	1.979031622176	-5.854874217598	-3.713972639185
C	4.540783445195	-7.771003931014	-2.271350875397
H	5.560963976727	-7.564030015222	-1.890938291229
H	4.620520883438	-8.514612688940	-3.091105518850
H	3.965743691958	-8.242380661226	-1.447338204464
C	2.334362450398	-4.973596111274	1.854610952491
H	2.235301541227	-4.011744911898	2.397760398181
C	0.918748200096	-5.527138465193	1.648675412755
H	0.919900336711	-6.510573583582	1.133832601912
H	0.295679716011	-4.835634829614	1.049697461166
H	0.415253792328	-5.671554664557	2.625625405883
C	3.177766277891	-5.909363440836	2.738494486142
H	2.673859876338	-6.097827723422	3.709209767758
H	4.174140759019	-5.472197536463	2.947957398709
H	3.337546426799	-6.888994358185	2.240804117889

2La

La	-0.124479077946	0.035075156006	-0.046683727331
N	0.846055893103	-2.199641586410	0.199845674854
N	1.607180031714	1.772465830393	-0.426907637605
C	1.450979185104	-5.177392668042	2.373279035978
C	1.518551977742	2.076647390986	-2.827496613981
C	5.884237784147	2.813341105142	1.171599132253
C	3.264296898583	3.239391243211	-1.557913397854
C	0.427185792032	-3.418153373865	3.678652900201
C	1.990188686085	-4.865415147342	-0.080914978607
C	2.125423535325	-0.756717062906	-3.431554111853
C	0.917899507747	-3.035709341673	1.288689783138
C	1.455423202065	-4.367121306003	1.225524082865
C	0.410324784958	-2.583305224239	2.552278564940
C	5.485997648945	3.752165747730	2.138224438006
C	5.191714320045	2.652189748737	-0.040933965174
C	1.103980564847	-5.420817353631	-1.043622761740
C	3.642713876660	4.434138930336	0.655636005845
C	0.944600296867	-4.721641991797	3.602342736053
C	0.421589627424	1.068238090043	-2.818960833597
C	-1.669771350485	-0.882342997775	-2.706483973136
C	-2.783395954302	-1.923937680746	-2.797153379772
C	-1.934662328724	0.487623020245	-2.445351986236
C	3.375172122270	-4.747450882092	-0.382376742532

C	-0.911088905101	1.471810461090	-2.534613094164
C	-2.477961342749	-2.177423270915	2.199564198121
C	2.664865688860	-1.988232212662	-2.696696875580
C	2.142223409575	-0.972143753172	-4.958000523698
C	2.270050678505	-0.269911137251	4.677774970126
C	6.238970780554	3.898723245105	3.453616576502
C	-0.377038703203	-5.585919311620	-0.719419554805
C	2.197298438480	-0.296889045714	3.137716785219
C	2.986664357510	-5.762302484785	-2.589223638786
C	5.657157310015	1.616279331943	-1.061851633993
C	4.936210497624	-5.263314156882	1.530455848665
C	-1.296284891409	-5.381209779293	-1.930039480244
C	7.709822040297	4.287921208757	3.230574422558
C	-2.664388420046	-2.561795035258	0.722793006534
C	4.596763636620	-7.299457173077	-3.807010380353
C	-4.175804286357	-1.387983989925	-2.449592487725
C	6.122121941384	2.631219524660	4.317772753711
C	-2.380592365049	3.212673008487	-1.373510205572
C	3.511220016341	-6.219491495154	-3.943785885293
C	5.458530561283	-3.316337554921	0.007705950837
C	-0.637105208831	-6.952547256879	-0.059737645292
C	3.213451016402	0.669355268241	2.521638509156
C	4.009587429325	-5.030015340734	-4.783674044393
C	1.871428552527	6.029809374655	1.600034641052
C	7.175799427298	1.397810240673	-1.070996152335
C	-0.331540411034	-1.260966218307	-2.935539889511
C	4.917323244341	0.280979628160	-0.876709119657
C	1.977778494790	2.674064326259	-4.009155672668
C	3.702831625613	3.812778123396	-2.764706550111
C	3.846009525342	-5.206140769785	-1.625485115122
C	4.373049616250	4.559086197904	1.853331140072
C	-2.799307075661	-2.567832294817	-4.197618218969
C	4.341570470681	-4.156892132779	0.639878627253
C	2.480920520127	5.378532328549	0.352382907954
C	2.121715585559	2.363456917998	-1.555313563828
C	1.620455397234	-5.858752754097	-2.277825034259
C	-3.826198841656	-1.930400158340	2.890107035098
C	-1.543506205454	3.587784781096	-3.731492273240
C	3.067472538950	3.559020399766	-3.990950583796
C	4.039442181818	3.452236412133	-0.293641657377
C	0.729235137894	-0.319131276364	-3.004650731025
C	-1.239633227371	2.950171456704	-2.362903752018
C	2.907205661149	6.477776205332	-0.640626942899
H	1.265503615228	-2.660667684673	-0.617047121065
H	2.149339055579	2.076912987468	0.392781526370
H	1.854596888981	-6.199313837109	2.293485434312
H	6.766219605949	2.185208354155	1.367524683330
H	0.021971618216	-3.035834182271	4.629296615673
H	2.804433644342	0.091401427721	-3.209552778684
H	0.954962040641	-5.373502230363	4.487590802821
H	-2.534651435451	-2.727156515542	-2.070362198106
H	-2.966950415008	0.812195773016	-2.265582719493
H	-1.987576697610	-3.034694540255	2.701704878367
H	2.762995722153	-1.801042692201	-1.610581849304
H	3.671962020215	-2.249593335941	-3.076995148706

H	2.034990761307	-2.891742699931	-2.836229587615
H	1.464077873783	-1.801865911051	-5.249895201444
H	3.163757335147	-1.231442322098	-5.304002724252
H	1.816884713691	-0.061576267838	-5.499027086330
H	1.982016562809	0.729428133666	5.067421723466
H	3.299543367229	-0.489234957419	5.029697863439
H	1.587876481775	-1.019169867303	5.125998419787
H	5.752331775157	4.729696874527	4.010358797995
H	-0.627412546891	-4.808850977476	0.032313266032
H	2.471835835228	-1.322687226180	2.813003647225
H	0.940760536232	-6.293995076738	-3.027566373283
H	0.856444574698	2.103067664740	2.793064028093
H	5.371675689377	2.005708080237	-2.060244481923
H	-3.691192612784	-1.609888867495	3.942686921128
H	-4.431787682473	-2.859300095637	2.889823340580
H	-4.425136641466	-1.152204168479	2.372500404520
H	-3.083490374733	0.505147204864	1.934177814166
H	4.145433242660	-5.822927350861	2.065388141601
H	5.622622139736	-4.832595093878	2.288713030141
H	5.512990552147	-5.988325998717	0.918621749556
H	-2.357354419719	-5.336996675570	-1.609472104447
H	-1.214095522678	-6.210304755031	-2.663113383137
H	-1.059969335890	-4.439366973308	-2.463300200116
H	8.227776179563	4.452647357460	4.197950104579
H	7.793985113949	5.215811903995	2.630325689484
H	8.259250092834	3.488985727456	2.690572080593
H	-3.128233944407	-1.724445414066	0.160246980900
H	-3.330308302539	-3.442185230098	0.612967905184
H	-1.690503264518	-2.808166657222	0.252352576292
H	4.919993815894	-7.665188676002	-4.803404819411
H	4.230319651947	-8.166503983958	-3.222008720358
H	5.495821249816	-6.903206785815	-3.290945663383
H	-4.516843424917	-0.636732715634	-3.191851388180
H	-4.210220732854	-0.914466288512	-1.448279622887
H	-4.913594925719	-2.214440778273	-2.452315072315
H	-0.684716870612	3.483471083125	-4.421698985864
H	-1.760590347836	4.669671760870	-3.617586193882
H	-2.427288829219	3.107753265666	-4.201364427280
H	6.589710830731	1.759938039028	3.813250594059
H	5.062792632337	2.372177156245	4.514535791500
H	6.632834329739	2.766819094926	5.293537970022
H	-3.366086293578	2.897526137752	-1.775195521270
H	-2.452112534127	4.294730088589	-1.145519468498
H	-2.227337610636	2.680243201374	-0.413059029078
H	2.651340357801	-6.673648382256	-4.483840936442
H	6.185010938424	-3.943218627648	-0.550422743702
H	6.031147907894	-2.782168082867	0.792968145894
H	5.053967296984	-2.560259118560	-0.691160016457
H	-1.708587547233	-7.066291337876	0.206975139347
H	-0.040772527043	-7.068079497691	0.865789871786
H	-0.364521402406	-7.779219707705	-0.748645817261
H	3.214434135017	0.604682647589	1.417770154420
H	4.238019906845	0.428826075180	2.868313811803
H	3.029383157032	1.728246236611	2.801482045893
H	3.215170313961	-4.268978063348	-4.919014949174

H	4.343938836363	-5.363656622597	-5.787790957508
H	4.869957088584	-4.529171806072	-4.292520239048
H	2.550650174165	6.794940156254	2.030014145926
H	1.653868196512	5.290661453370	2.394097909205
H	0.924032858494	6.544464893697	1.343251379987
H	7.728690471766	2.354350678847	-1.162965437009
H	7.462971350423	0.750832344134	-1.924639280902
H	7.529423211213	0.888408599775	-0.150219246253
H	-0.101310801635	-2.324998086826	-3.101668712927
H	5.183415497534	-0.184892515998	0.094075037596
H	5.191305670418	-0.431460381029	-1.681489649464
H	3.818783583749	0.416404734809	-0.895199880085
H	3.430500549083	4.023726141793	-4.918623168989
H	-2.613511551350	2.831781743371	1.609494112842
H	1.483447736979	2.422029979030	-4.961088171800
H	-1.381423004990	4.942809644478	1.915550190482
H	-0.295053756646	3.788848995988	1.064968066471
H	-0.078448546278	4.122370676872	2.807661491354
H	4.590664547700	4.464831412596	-2.734940321482
H	-1.694965321170	3.108573809006	4.538994068373
H	-3.179519531746	2.275240960906	3.983610741261
H	-3.007002133928	4.047976485975	3.750364485526
H	-0.320470226668	3.432083851683	-1.967502622118
H	4.918683755758	-5.113910150001	-1.854557417741
H	4.074971976947	5.314679429538	2.595650386677
H	2.056810357453	7.156494333444	-0.859966754304
H	3.257365087213	6.051827993181	-1.598626953736
H	3.728912415752	7.087672906483	-0.210843279127
H	-3.565761937110	-3.367641142064	-4.247845860504
H	-1.823515333697	-3.021244108415	-4.459168002965
H	-3.038519290749	-1.811552159092	-4.973708240954
H	3.741803009669	-3.488911188877	1.294664660114
H	1.693643020003	4.768252397679	-0.143418323342
C	-0.145807442564	-1.199484761796	2.565427218365
C	0.196672889183	1.239619686597	2.621644293287
C	-2.009339360432	0.333695012563	2.093305870466
C	-1.158373400239	1.466292130632	2.338875128495
C	-1.796111305918	2.845578571474	2.365738261134
C	-0.837523164614	3.982186127972	2.012760291550
C	-2.461132849003	3.082123169986	3.736737148651
C	-1.524769264089	-0.993137990196	2.326567125015
C	0.760335637466	-0.071702055520	2.687998286867

2Sm

Sm	-0.008568466426	-0.022551450469	-0.092648161639
N	2.351326795335	0.203379082428	0.360293362385
N	-0.705354347776	-2.291729019619	-0.407284702596
C	3.079377804709	0.523327244152	1.476968159391
C	2.395046587812	0.726024884273	2.722576299089
C	3.089659427952	1.068683795755	3.891081688548
C	4.484404457951	1.234941017113	3.875812564553
C	5.173857750671	1.038850126830	2.666632655248
C	4.509559563123	0.681180842462	1.481779060731

C	0.916080080094	0.556040144241	2.681553983520
C	0.332341811530	-0.735266633039	2.865249860336
C	-1.068782288395	-0.871669441103	2.759456211245
C	-1.922978144813	0.235565523890	2.528214585127
C	-1.318516283890	1.494230331283	2.313887811082
C	0.085107770180	1.669861346260	2.369846410821
C	1.203686212175	-1.918222877253	3.272631795016
C	0.963720805796	-3.189057342911	2.451213934164
C	1.037095704338	-2.178777360467	4.781284935502
C	-3.438466535524	0.053339649193	2.597867495803
C	-3.849366052971	-0.521779972069	3.968006043477
C	-4.239868399775	1.327361101438	2.310506862574
C	0.702808089653	3.045594753469	2.152337780629
C	0.056847705018	3.807921734581	0.990707572493
C	0.672399775033	3.865675435726	3.453454328901
C	5.250781303129	0.452978536894	0.202173907754
C	5.643068186203	-0.867328867355	-0.160766448475
C	6.282640142944	-1.075371323193	-1.394223277468
C	6.534004112256	-0.020317756901	-2.290529587554
C	6.147337212982	1.274662806724	-1.910764172229
C	5.516643734331	1.535589707019	-0.678500213334
C	5.405820196298	-2.027850528555	0.802339053901
C	5.163817676930	-3.374525470809	0.109827079637
C	6.569389410605	-2.139624390932	1.805321014570
C	7.197121788403	-0.273748430828	-3.637774188170
C	6.321499120427	-1.157108660798	-4.543496932193
C	8.607760309581	-0.864816437747	-3.478424424255
C	5.086168471094	2.954433466490	-0.316268163085
C	3.696939139949	3.265441279355	-0.903518726322
C	6.110183479544	4.025003793104	-0.716756916166
C	-1.087525484646	-3.000983665284	-1.515892559717
C	-1.125251441656	-2.337204073865	-2.788460560841
C	-1.521320198616	-3.010936325232	-3.951789315796
C	-1.889147407129	-4.366364214320	-3.906871013901
C	-1.847505476370	-5.036938012620	-2.672447260382
C	-1.457085948028	-4.390667543339	-1.487309373998
C	-0.722279176184	-0.903068879443	-2.773604257152
C	-1.693136136558	0.101298992971	-2.491117308154
C	-1.293281257070	1.458831521067	-2.442042812755
C	0.050747837747	1.851710849876	-2.634713590433
C	1.008905370445	0.825938003553	-2.841177396538
C	0.647267553388	-0.535727571596	-2.944057872374
C	-3.156722307808	-0.282142151768	-2.315346774621
C	-3.819992561359	0.438253580062	-1.137484081542
C	-3.930145724598	-0.067083950999	-3.627454625179
C	0.478464412568	3.318219473969	-2.696963723564
C	-0.672613858405	4.313438868991	-2.516427178031
C	1.227955381841	3.615832811263	-4.010434756663
C	1.676268394706	-1.588429790171	-3.337650353224
C	2.983902162472	-1.522319952166	-2.544476715676
C	1.933076424265	-1.503550206753	-4.854018561838
C	-1.375610226304	-5.117675711609	-0.182551259837
C	-2.400355828073	-4.952128138191	0.789731051035
C	-2.266460244062	-5.586791354123	2.039152887007
C	-1.151917378645	-6.381343627276	2.354799990126

C	-0.156985075858	-6.544355162129	1.374953589998
C	-0.243919724700	-5.929423491926	0.113385652988
C	-3.645186757083	-4.134620115806	0.453297288364
C	-4.340312886325	-3.532343675215	1.679589429077
C	-4.640478574993	-4.975136860844	-0.368418160718
C	-1.022528572013	-7.030845828553	3.726130292383
C	0.138981252190	-6.420612425219	4.530367872368
C	-0.891942958490	-8.559682876002	3.630043822148
C	0.880963238238	-6.084694233799	-0.907863670951
C	1.622390183557	-7.424197476210	-0.816112228912
C	1.867437339062	-4.905969043480	-0.815817425850
H	2.997591700088	0.089014605637	-0.431734705874
H	-0.733011243214	-2.913008822358	0.412024540290
H	2.523881140698	1.205890668377	4.826825927381
H	5.026773601607	1.509517372682	4.791814461085
H	6.267371095411	1.170441587882	2.632273498281
H	-1.514191615173	-1.869055294445	2.901088505660
H	-1.952887175794	2.372132582746	2.129151231860
H	2.253778655387	-1.602430384584	3.104914118482
H	-0.067427661409	-3.587330790010	2.556181003127
H	1.646616785530	-3.995745025835	2.784791225546
H	1.155996581509	-3.011988906734	1.376518133667
H	1.273386798626	-1.273949688625	5.376335727390
H	1.711892560939	-2.994313784565	5.112652708544
H	-0.004218020173	-2.481617797110	5.021290204258
H	-3.708767951251	-0.704506284741	1.828770712206
H	-3.328646007036	-1.471295600442	4.197964603801
H	-4.939573423288	-0.722133510929	3.994332887603
H	-3.610614078960	0.197056890084	4.779027798960
H	-4.059754285391	2.098877935229	3.087652915452
H	-5.324878355312	1.103015345284	2.313411930913
H	-3.995805304877	1.772435029395	1.326670123153
H	1.767822837273	2.871383572243	1.893368244313
H	-0.984397691269	4.121842963225	1.213847312639
H	0.034129050741	3.187367751709	0.071796137461
H	0.631923857111	4.725897870769	0.754947228710
H	-0.371231439529	4.035391291953	3.792390288759
H	1.149874572398	4.855943367454	3.305051438210
H	1.215728775399	3.343252430813	4.264613538232
H	6.584322570390	-2.096546767197	-1.672374710157
H	6.349816107484	2.107980672129	-2.602700425636
H	4.491224908693	-1.777604390297	1.381687087955
H	4.360921213910	-3.308561730092	-0.648550751113
H	4.864256093036	-4.139475691913	0.854545273570
H	6.078316565500	-3.755181789166	-0.391355167125
H	7.521177260913	-2.352794547439	1.275107220578
H	6.388150378518	-2.961600287933	2.528755822265
H	6.699839596987	-1.202632858249	2.379523867760
H	7.301790710769	0.715835238947	-4.134836674168
H	6.179845355880	-2.165569449052	-4.101813802141
H	6.789564631962	-1.291468206029	-5.540581994192
H	5.315456389260	-0.715690180780	-4.690768490764
H	9.247249163654	-0.214460201016	-2.848851286163
H	9.101427481137	-0.986550638547	-4.464697753484
H	8.571599969558	-1.864650322846	-2.998012424881

H	4.984036385437	2.982391237412	0.787857660368
H	3.733912839024	3.267850782661	-2.014094482703
H	3.339579684330	4.264707222848	-0.575745832102
H	2.954986466962	2.505642997604	-0.585508274883
H	7.118916407338	3.790991452731	-0.321166124085
H	5.807239837786	5.016000396140	-0.321067585083
H	6.195666940074	4.129350654937	-1.818627011537
H	-1.536165291145	-2.462176831643	-4.907354023554
H	-2.199175580504	-4.893745659273	-4.820295203240
H	-2.120838327049	-6.103367460825	-2.619373711356
H	-2.061651304021	2.227786890128	-2.281400710201
H	2.066486654794	1.102288595079	-2.969335276673
H	-3.170473716316	-1.369932939670	-2.096857852058
H	-3.235072546485	0.289677299686	-0.207087753831
H	-4.838760513727	0.040376005550	-0.955012969085
H	-3.921755789837	1.530632732314	-1.308416970523
H	-3.919192726550	1.001977303068	-3.927365920393
H	-4.988462185420	-0.380742601514	-3.516860467467
H	-3.483171211673	-0.657494384032	-4.451269244005
H	1.202057332032	3.478779282706	-1.867895032664
H	-1.410070952047	4.229830465005	-3.341595218801
H	-0.282894925462	5.350555026630	-2.523319272177
H	-1.214224523562	4.171142680118	-1.560930751636
H	2.114960125499	2.966865930845	-4.143355420452
H	1.578660655358	4.667539404610	-4.025403326314
H	0.562751044786	3.462269422569	-4.885425282235
H	1.209245973830	-2.574004129904	-3.135472154212
H	3.523849935339	-0.560859375631	-2.671408192091
H	3.676759908605	-2.317413286334	-2.883818776043
H	2.805855413174	-1.675331884064	-1.462683358781
H	0.992955180879	-1.617286129387	-5.429806008516
H	2.632985783067	-2.301493341181	-5.176061618528
H	2.383406778366	-0.525778777069	-5.127252204002
H	-3.054809149996	-5.467402188603	2.798401977677
H	0.721237808830	-7.166523328367	1.604070738929
H	-3.307871710717	-3.293410037470	-0.189709563152
H	-4.833945411534	-4.308080665285	2.301107500677
H	-5.130562153628	-2.820221968738	1.365417553193
H	-3.627329307220	-2.987778169619	2.329004616014
H	-4.177643112100	-5.345780817211	-1.302711182269
H	-5.531786446306	-4.373483637493	-0.643910150660
H	-4.986391880220	-5.852084609497	0.217580728100
H	-1.963428114418	-6.808060187228	4.276135693488
H	1.110947305666	-6.601993026804	4.025712128646
H	0.198781868572	-6.866843509613	5.544634523865
H	0.022491275665	-5.324109253596	4.641566934165
H	-1.740313707351	-9.001572037507	3.070291034149
H	-0.865496632134	-9.019461079469	4.639502762631
H	0.042461624332	-8.851907166257	3.107425763295
H	0.409381404015	-6.031648654752	-1.910261187726
H	2.226314922458	-7.503389849672	0.112061595689
H	2.326674575299	-7.530187519653	-1.666012864604
H	0.923156006016	-8.284309107855	-0.839960289548
H	1.347201100002	-3.931537262493	-0.895828291191
H	2.625930747041	-4.962119990088	-1.623967036563

H	2.404884625163	-4.921027522910	0.154538821325

2Eu (using Eu1)

Eu	0.022983536393	-0.068041356416	-0.014018001249
N	1.881366304695	1.391043594927	0.395403292281
H	2.161783292252	2.066167670962	-0.329709924894
N	-1.878842897444	1.331843718205	-0.407013091490
H	-2.178045484996	1.981004995553	0.333171815933
C	-2.698194420194	1.483110016015	-1.488118527721
C	-2.470193963192	0.692255421300	-2.671067300372
C	-3.334935619607	0.781909191228	-3.772628351462
H	-3.128082670839	0.168781619436	-4.664536372671
C	-4.428958655132	1.662713220949	-3.769737335848
H	-5.101590427712	1.723250957681	-4.637077090512
C	-4.610866336791	2.506219150725	-2.659404580772
H	-5.411458020563	3.263519619354	-2.673140248579
C	-3.768266923294	2.447589228120	-1.538618780187
C	-1.214577260187	-0.118670162707	-2.688885514944
C	-1.210516462143	-1.500071821244	-2.330948679901
C	0.023583917670	-2.194723480353	-2.263819969742
H	0.030179414441	-3.264969891410	-2.004575021709
C	1.255107371536	-1.562478192537	-2.533355799831
C	1.231482645267	-0.194670160724	-2.888022294230
H	2.180250568131	0.314381229546	-3.107348433592
C	0.027842414603	0.535464397446	-2.982423327009
C	-2.520049152090	-2.229035320857	-2.055085394224
H	-3.224363023740	-1.464183548832	-1.661078212943
C	-2.383016662269	-3.354811349494	-1.021390648461
H	-1.841429385214	-4.231371692332	-1.433806727206
H	-3.382442995780	-3.711151626453	-0.702124282145
H	-1.838266993923	-3.020933811183	-0.115748829136
C	-3.121840421895	-2.777644463609	-3.362686214973
H	-3.314053955080	-1.967887157727	-4.090619360104
H	-4.083863101592	-3.292455570193	-3.162916245400
H	-2.432302346851	-3.509279350595	-3.833400074488
C	2.557708047751	-2.349832098098	-2.521069402703
H	2.352548927344	-3.302018728119	-1.985253155534
C	3.689445514701	-1.619577239011	-1.784026406417
H	4.571232964544	-2.281407559733	-1.667810471864
H	3.377507963020	-1.273848206015	-0.778406641895
H	4.022625591342	-0.725021969849	-2.344245802348
C	2.972405952520	-2.711974255055	-3.959804520199
H	3.176575410157	-1.795935734070	-4.551944540696
H	2.175291029959	-3.279162120651	-4.480509476367
H	3.894244099895	-3.328883145997	-3.960249462293
C	0.042150468723	1.983919157146	-3.458427512756
H	-0.809708413676	2.490185910422	-2.960016815897
C	1.320611851088	2.754430442907	-3.115531861286
H	2.215301603271	2.343038369958	-3.627090818972
H	1.527353669992	2.772999019315	-2.028295749002
H	1.228223940342	3.806685750250	-3.443066549831
C	-0.220194614400	2.027523868824	-4.976996707857
H	-0.264650477318	3.078113414010	-5.329853306449

H	-1.179075786659	1.540133626573	-5.236242956008
H	0.593381205759	1.513898560428	-5.530916501357
C	-3.845198746452	3.479265040024	-0.454237440275
C	-4.584686434244	3.262224526996	0.741731493791
C	-4.615803166540	4.269695051669	1.722931991383
H	-5.195799480859	4.101831595662	2.641984508870
C	-3.931650043645	5.488696444536	1.564096392486
C	-3.187971189149	5.673644669933	0.389096207913
H	-2.639048406736	6.620783461661	0.255227410444
C	-3.125031109433	4.694205812444	-0.619503772788
C	-5.369908918127	1.967292741073	0.931325181496
H	-4.789106280918	1.167320672157	0.422308371684
C	-5.542961119765	1.565183614654	2.401888532834
H	-4.573680384970	1.545282640497	2.941295143060
H	-6.002321751296	0.559250923993	2.476923049382
H	-6.212096467230	2.263671192708	2.945585279745
C	-6.742479659450	2.052779955473	0.238040947738
H	-7.303648123163	1.101976233140	0.353029722658
H	-6.633462732049	2.255381044493	-0.844103718910
H	-7.352284190332	2.867424613464	0.681530893424
C	-3.951618751924	6.565499619527	2.641790675138
H	-3.599269162707	7.503467392134	2.158587929948
C	-2.961306117958	6.232851113003	3.772590411343
H	-3.235484939088	5.278665374190	4.269084911377
H	-2.953350513908	7.029713219544	4.545065013976
H	-1.930121504383	6.122908519137	3.381273045864
C	-5.358554999337	6.831470555268	3.197295061078
H	-6.077747421038	7.059939471782	2.385551766530
H	-5.346831164659	7.688556878249	3.901282906736
H	-5.749010908045	5.955848543023	3.756303212784
C	-2.298301337257	4.966384408048	-1.872164794572
H	-2.326154730693	4.043072402959	-2.484888567486
C	-0.826910784524	5.255489697981	-1.540439718854
H	-0.709703682370	6.170455127314	-0.924250839646
H	-0.237850607976	5.406639660891	-2.468146319587
H	-0.370559258235	4.412592812500	-0.984349765266
C	-2.923000561038	6.088075927244	-2.717800199099
H	-3.965063724586	5.838941641612	-3.002000089944
H	-2.341956586923	6.249690046433	-3.649511357576
H	-2.945604004800	7.049622394468	-2.163132382531
C	2.698766750027	1.543447880842	1.478233021805
C	2.476895766305	0.738244547051	2.653342646473
C	3.327010825253	0.832973010987	3.765052163748
H	3.110108477971	0.221708311787	4.655827097927
C	4.416368629474	1.719777508642	3.772020659824
H	5.085161796312	1.779950505944	4.642351904607
C	4.597852083814	2.570083362083	2.667217441362
H	5.401619750310	3.323770900413	2.685389680438
C	3.756048075106	2.521265024381	1.544642745074
C	1.222392686192	-0.071850073565	2.663394859453
C	1.216546667053	-1.460308997592	2.334695475530
C	-0.020183091276	-2.150480631357	2.272993416380
H	-0.029584065074	-3.226686945918	2.039613614652
C	-1.251737208509	-1.507588433313	2.519144834993
C	-1.225949178779	-0.131042370701	2.836485332956

H	-2.174007196084	0.390158190554	3.034028019393
C	-0.019351902185	0.595272216987	2.922556592208
C	2.525026154059	-2.199473241029	2.081793852327
H	3.237569551232	-1.442426200028	1.687005291635
C	2.391786108792	-3.335182074708	1.058138091636
H	1.840889215340	-4.204302133823	1.473902374568
H	3.392095934076	-3.700534573017	0.751974975602
H	1.857473920583	-3.007803240051	0.143800433663
C	3.110846825279	-2.737714484971	3.400788406389
H	3.301091798942	-1.921370031897	4.121872481538
H	4.071490142505	-3.260664015401	3.216003102796
H	2.412178941641	-3.459916362850	3.872635806328
C	-2.554652102428	-2.294168928108	2.521668904330
H	-2.348585788129	-3.257950097132	2.007520243250
C	-3.685082385688	-1.580197033906	1.767430806637
H	-4.561185188246	-2.249465648391	1.650842316732
H	-3.365858525438	-1.240657857452	0.762189560735
H	-4.029770681528	-0.683616958599	2.316920348722
C	-2.971167015859	-2.624470960701	3.967581232543
H	-3.174023122156	-1.695218954400	4.539287414874
H	-2.175781460820	-3.181802183450	4.501448637121
H	-3.894333294098	-3.239283567921	3.980424432965
C	-0.039055632313	2.051773320909	3.370613467470
H	0.930419222121	2.492590276924	3.065082809499
C	-1.152052768115	2.893523053586	2.738053007149
H	-2.170413790362	2.517952733446	2.968548737878
H	-1.044210048732	2.946208448759	1.637157325067
H	-1.105978458155	3.931385866942	3.117656054426
C	-0.096695443258	2.101868152213	4.909224661398
H	-0.048717305090	3.151324391425	5.265113208843
H	0.750669140542	1.547743292591	5.359664035644
H	-1.039932529136	1.654885434077	5.288259569777
C	3.824773880540	3.564734661225	0.471418985469
C	4.506021301391	3.332614494999	-0.756412518087
C	4.514667255527	4.339709118109	-1.738001186185
H	5.046474932331	4.160041709386	-2.683862100058
C	3.868196838820	5.574338902597	-1.545790941924
C	3.188520037531	5.778195015189	-0.335671420776
H	2.673757285131	6.739641480769	-0.176526427507
C	3.145834572251	4.798700113412	0.674116823002
C	5.262050770572	2.024084198446	-0.972983111095
H	4.674307569625	1.229549314127	-0.463775513953
C	5.406537162342	1.631503757873	-2.448645699624
H	4.427197950896	1.616705645961	-2.968897436239
H	5.862696059573	0.625238585664	-2.537460250317
H	6.067895415614	2.331078394606	-3.000401565002
C	6.646316438718	2.079384982379	-0.299729653595
H	7.186216882068	1.117801864181	-0.426364608662
H	6.557605391303	2.280110271495	0.784630122902
H	7.265782943561	2.883026913814	-0.749814977167
C	3.874497415525	6.652121960019	-2.622196809844
H	3.471557725714	7.575063993471	-2.150027630037
C	2.936729578490	6.284571367600	-3.785715369772
H	3.268174996543	5.348836758752	-4.282302821097
H	2.916594302818	7.086975280463	-4.552136367166

H	1.899915067806	6.125198962929	-3.427462881284
C	5.290044838675	6.970643881756	-3.128119327746
H	5.965980221941	7.240576966704	-2.292318265064
H	5.271220211358	7.816462941470	-3.845731438194
H	5.737811800397	6.102633760856	-3.655125723111
C	2.417924424845	5.095220783821	1.982621539732
H	2.274479774449	4.123184466579	2.497292363097
C	1.030541407639	5.720202768188	1.780106269248
H	1.087358444627	6.725246943169	1.312510940873
H	0.520605534304	5.847404619367	2.757349480298
H	0.387783136799	5.085965921119	1.140815006512
C	3.291303654772	5.970740137847	2.898540411243
H	4.263790650286	5.484707605215	3.110003172559
H	2.783821903375	6.158891761771	3.867576116660
H	3.499999673365	6.953422086511	2.425598805712

2Tm

Tm	-0.041802993801	-0.004615043978	-0.067336397755
N	2.232007921738	0.259998415232	0.198875989810
N	-0.766929624606	-2.170937411357	-0.296048353523
C	-2.007708827535	-4.275171943278	-3.760186485793
C	-1.623973697921	-2.925197373979	-3.823293010084
C	-1.962468080681	-4.931658638762	-2.518789185585
C	-3.946006905229	-0.039079648419	-3.488653429335
C	-4.736040801472	-4.808851786744	-0.211466409228
C	1.767378543176	-1.467111883183	-4.855810392491
C	-1.210210881447	-2.244728180796	-2.670433923829
C	-1.553136885722	-4.277694571035	-1.343786305611
C	-3.190553185619	-0.160840297122	-2.153329655121
C	-3.725757019198	-3.982189294841	0.606096929030
C	1.476688068057	-7.372022634171	-0.675838412768
C	-0.780504456220	-0.819745889313	-2.668503102364
C	-1.167746086875	-2.893545685570	-1.391610234848
C	1.577659793294	-1.554197035838	-3.329451960822
C	-1.473285673821	-4.999853416759	-0.036602941691
C	-1.722950794315	0.199422988498	-2.342920956995
C	0.752922675538	-6.023862799553	-0.781147842945
C	-2.492152436924	-4.818293133915	0.938840859789
C	0.588965879550	-0.480920321964	-2.887885176660
C	-3.867795532642	0.651915495028	-1.048190038472
C	-0.357869721763	-5.837040319434	0.251206570359
C	-4.409181295100	-3.369697340917	1.833765162312
C	-1.297183356924	1.551249379205	-2.309437522785
C	6.198964332792	-1.281534726577	-4.787873121645
C	-2.367184372140	-5.460132588962	2.185561579550
C	0.973310337928	0.877873407928	-2.821020435914
C	2.923269641486	-1.525033889570	-2.600390261687
C	1.759926367855	-4.859852175742	-0.729909367964
C	-0.279884846407	-6.458108304219	1.510394779421
C	1.126790232268	3.617394213694	-4.096477936947
C	0.041521983191	1.917721012064	-2.581232451552
C	-1.267942518865	-6.278189192623	2.494365320616
C	-1.016229467514	-8.460394592382	3.768069971482

C	-0.642721371413	4.394984915896	-2.497822886949
C	0.480979370643	3.375692045474	-2.717195794407
C	7.046496815587	-0.318038068352	-3.937848047038
C	-1.145933931665	-6.931709070967	3.864507410740
C	8.496558385105	-0.812245792743	-3.812829115834
C	6.404329943054	-0.070328320661	-2.579541033723
C	5.178811530982	-3.376905947984	-0.010963352707
C	6.304421094260	-1.094566233060	-1.622882575265
C	0.012659791061	-6.323251668393	4.674604772531
C	5.857671770710	1.185410841705	-2.262919745578
C	0.851828616355	-3.171307017935	2.519872236025
C	4.221445896991	3.616626150324	-1.920743766397
C	5.673610848217	-0.895419961695	-0.381728351069
C	5.581759204578	-2.038823246759	0.624666796392
C	5.228882522327	1.436357959226	-1.029081155715
C	0.129624309683	3.904897124043	0.936808053328
C	5.142815227808	0.386800020494	-0.073041806860
C	3.021717838964	0.512698906233	1.290262909869
C	1.135022742628	-1.865958100321	3.267851021307
C	4.681834777024	2.821514237109	-0.693355349484
C	0.787489270277	3.078708642601	2.043979502410
C	4.455559420899	0.615262756148	1.235759815445
C	6.898037062757	-2.176271045586	1.410220821591
C	2.403308347664	0.704426039026	2.571382532006
C	5.710057620918	3.632207237291	0.117229388819
C	0.972430147382	-2.041992110815	4.789529421740
C	0.838447187182	3.853393228853	3.372843013808
C	5.184807536621	0.898838076109	2.402711070219
C	3.159834596548	0.982738659806	3.717710742418
C	4.558827520742	1.085566318842	3.646967675713
C	-3.446318138964	0.242095028016	2.609008523617
C	-4.204284014356	1.552584035117	2.368570685406
C	-1.927790466328	0.362162728128	2.479043077760
C	-3.824654979875	-0.319889038913	3.994272285993
C	-1.108728455308	-0.767106840534	2.729729287857
C	-1.285008011067	1.591837371209	2.211372087960
C	0.297905855913	-0.676313406698	2.810768640098
C	0.124740741733	1.722791698028	2.249396207944
C	0.920436580517	0.587540671059	2.578578005325
H	-2.331779032289	-4.809299014914	-4.664756851210
H	-1.638175642399	-2.387575032021	-4.785015226872
H	-2.248156024205	-5.993973889815	-2.450805670724
H	-3.496944863587	-0.693116088702	-4.261056457331
H	-4.282422489786	-5.187203872318	-1.147114448423
H	-5.008522198267	-0.333972108319	-3.366881715543
H	-5.619444623894	-4.194415620840	-0.484109451815
H	-3.920381952228	1.005223679274	-3.864810159194
H	0.800349143958	-1.553898559134	-5.390167005281
H	-5.092483278247	-5.679973083327	0.376894133476
H	2.432525101017	-2.279804595932	-5.212813040868
H	0.765539046297	-8.222627142570	-0.674217631105
H	2.229195279740	-0.498987312560	-5.144024931878
H	2.166994646244	-7.502831360961	-1.533664055794
H	0.267848107939	-5.982878839290	-1.777786708833
H	-3.215225403469	-1.229464213727	-1.854346140079

H	1.097808054606	-2.529806646334	-3.110342593660
H	-3.378478126930	-3.146395363749	-0.038567177570
H	-4.883016013763	0.260254958831	-0.836254829905
H	6.640643488800	-1.417310055227	-5.796826896399
H	-5.192310490652	-2.649071242635	1.521245629677
H	0.382583903043	3.465341366881	-4.905473076522
H	-3.981432556724	1.723299831662	-1.316533090990
H	-4.909654828768	-4.138648349770	2.458197499502
H	2.092923397581	-7.442958134301	0.244893477149
H	5.162715623634	-0.907725762139	-4.911127253032
H	-1.860943870499	-8.901213305731	3.201983809687
H	3.576367384258	-2.339244376893	-2.971945889898
H	6.136166063139	-2.281907011316	-4.310739230266
H	2.503923781720	-4.948001299300	-1.548525841111
H	-1.426057883204	4.298524836597	-3.277965017781
H	-2.043611907564	2.334919402043	-2.122110475581
H	-3.151345388990	-5.328897958985	2.947191273715
H	-3.284399974211	0.596667681735	-0.108469675347
H	1.256251338861	-3.878211185718	-0.826371020254
H	2.027320190790	1.136771600461	-3.004653950001
H	1.976490136937	2.935547471957	-4.291130450105
H	-0.078207522879	-8.753964682208	3.252766413303
H	-3.688336895534	-2.832078531942	2.479937897087
H	3.482188516126	-0.578567786735	-2.755596312398
H	-0.805253330505	-2.776461443780	0.533398432441
H	0.585381508863	-7.099792083404	1.734273309523
H	1.507992847188	4.655990184801	-4.169046398615
H	2.793381864060	-1.670418754895	-1.511311368313
H	2.313562180095	-4.861992909966	0.231491833587
H	-0.997670884312	-8.920570461229	4.777490324476
H	8.964808146213	-0.923623105725	-4.812522994490
H	7.069373557781	0.661290024252	-4.464880413083
H	-0.238802048023	5.424857808380	-2.559414642784
H	-3.781363451563	-0.498761294935	1.848473943090
H	-2.088936524104	-6.708467823158	4.410714713519
H	-1.132351605723	4.290553413816	-1.510831138093
H	8.543409140818	-1.801884673569	-3.312756555424
H	4.243845548938	-3.283542675212	-0.595515775284
H	1.266717208770	3.552375613715	-1.948133775267
H	-3.991186329751	1.997680819687	1.377983036716
H	-5.296536428420	1.375144452255	2.423686859560
H	6.715412990528	-2.089306730049	-1.856163061004
H	1.049563957893	-3.062742781340	1.437806806628
H	2.833824042077	0.171299481674	-0.629079902273
H	5.965214718927	-3.772308235522	-0.687429884442
H	3.540577282154	3.023295383000	-2.561494543488
H	-4.920753971110	-0.470948610382	4.064756306567
H	5.931338024960	1.990721832991	-3.010348127222
H	9.112171200873	-0.107738082949	-3.218679691463
H	5.013058700853	-4.142613863558	0.774291801717
H	-1.585498696024	-1.742165256961	2.914795193256
H	0.986238632553	-6.504118962990	4.172832891178
H	-3.339508002921	-1.292447115279	4.203264151382
H	-0.191790824132	-3.528330555313	2.645632049276
H	5.075611269644	3.941134972428	-2.550661483479

H	0.050427146647	3.315491101523	0.002630180618
H	-0.103876506376	-5.226858404574	4.787164133495
H	-1.892762243370	2.485922463473	2.016739322894
H	0.068381630622	-6.771264131838	5.688337813638
H	-3.951538257183	2.307142913356	3.142059242957
H	1.507246375493	-3.979720362896	2.900444726844
H	-0.889815167805	4.249314423518	1.210654229678
H	3.684521951892	4.535538758331	-1.608257440735
H	4.789245418166	-1.760072043269	1.350540882854
H	-3.521260722295	0.385419550247	4.795453855674
H	0.728933803041	4.810122689051	0.712464719872
H	2.192731863805	-1.592269788149	3.076936662198
H	3.795505401477	2.661479876654	-0.042382291905
H	1.834711156958	2.872709465508	1.739419514131
H	7.740349959434	-2.417845266583	0.728227398479
H	6.635610263877	3.790025092514	-0.474865651943
H	6.826268785692	-2.986751623123	2.165017925545
H	-0.075211019425	-2.302406154440	5.050911513064
H	7.151130148148	-1.237101855435	1.939638126042
H	1.626551156041	-2.858004426666	5.158997677766
H	5.302186839999	4.628427764779	0.388270303303
H	-0.184725418367	4.053225399795	3.754912996479
H	5.984746560500	3.110166898461	1.053386337375
H	1.237534342341	-1.114588663733	5.335592610567
H	1.350906843587	4.828145947123	3.238990340319
H	6.281656123141	0.971096449405	2.327145760299
H	1.388369288042	3.282468142853	4.145861080298
H	2.640651163201	1.115413582223	4.680677831434
H	5.152430629230	1.304191623113	4.546025589492

2Yb (using Yb1)

Yb	0.000996742509	-0.097376861655	-0.000728532540
N	1.836849516467	1.258276734672	0.235801064501
H	2.101354705680	1.899548682461	-0.522576587860
N	-1.835551848138	1.257689967410	-0.235174777315
H	-2.099920977281	1.899079407428	0.523132118353
C	-2.696010217959	1.424558826616	-1.282399914682
C	-2.508090484669	0.631109784843	-2.471028549514
C	-3.399141360263	0.726306146177	-3.549043551932
H	-3.215575168522	0.124072360517	-4.452886013516
C	-4.494536694912	1.605342710304	-3.507030285086
H	-5.196255409759	1.666091922177	-4.350991134355
C	-4.639137374910	2.449762475097	-2.392361494209
H	-5.443687424706	3.202759475126	-2.380108507613
C	-3.756303655903	2.400483520245	-1.301076748502
C	-1.237848484795	-0.154166215825	-2.496818606883
C	-1.191785246142	-1.538331747684	-2.154838519535
C	0.067773718256	-2.182443729491	-2.053411521732
H	0.107595332157	-3.256034043702	-1.814775549262
C	1.279585112247	-1.506849055549	-2.304674294985
C	1.212150999417	-0.138156916309	-2.649123124711
H	2.144990184618	0.402997046210	-2.858581428169
C	-0.014044773744	0.547354868604	-2.762087246727

C	-2.477259080589	-2.339531103241	-1.999680543413
H	-3.238644692934	-1.638744136386	-1.594258875295
C	-2.346463058157	-3.540704411684	-1.055448445916
H	-3.346405432566	-3.949469151959	-0.808824498311
H	-1.845539574676	-3.268568775496	-0.107100191905
H	-1.764161256507	-4.364619309226	-1.517842863271
C	-2.976797191545	-2.812757123779	-3.379005420296
H	-3.160708087265	-1.962489811178	-4.060713677793
H	-3.925008342529	-3.379049718737	-3.275689558033
H	-2.228794140454	-3.480487412432	-3.855293786761
C	2.601424399495	-2.258301076617	-2.336245033117
H	2.419133403508	-3.250307908948	-1.870756410812
C	3.713908707219	-1.553472264765	-1.550132357713
H	4.611754034242	-2.199864446085	-1.477559236507
H	3.391165593962	-1.279255638726	-0.527789747819
H	4.022108599385	-0.617423788611	-2.051661142631
C	3.025245584674	-2.509635925161	-3.796021158182
H	2.241083652175	-3.054722965741	-4.358531895086
H	3.959226163103	-3.106714722921	-3.835387549636
H	3.214186224229	-1.550909965796	-4.321953615910
C	-0.050444945977	1.978702380708	-3.284080513953
H	-0.972605485662	2.442623547679	-2.880856874706
C	1.135385723334	2.854622217611	-2.872762032966
H	1.189931191654	2.996670841989	-1.776362573825
H	1.033173748939	3.860181740786	-3.321544380898
H	2.111009856081	2.456042142058	-3.218402131516
C	-0.193080408670	1.936733060063	-4.818767957045
H	0.698350326076	1.466845798217	-5.284992823015
H	-0.292099262387	2.963561309334	-5.226254916155
H	-1.086928323870	1.359463213378	-5.125136284397
C	-3.783474792628	3.453650302978	-0.233907295013
C	-3.087442859390	4.673733414794	-0.463786521793
C	-3.099744903608	5.668768822130	0.531387351930
H	-2.572197718183	6.619281437910	0.349523733797
C	-3.771316172581	5.498037407395	1.751341611147
C	-4.440975432079	4.280356161726	1.967579928467
H	-4.971180494531	4.126459243492	2.919006900296
C	-4.459087784679	3.257444567966	1.002461210038
C	-2.390514441963	4.949737582838	-1.793303400407
H	-2.291329079972	3.975320963308	-2.313203670348
C	-0.980779610963	5.534582357998	-1.634434159604
H	-0.327737113105	4.866676422356	-1.040928896174
H	-0.989361025769	6.527011859710	-1.138066620305
H	-0.507305111000	5.673415623244	-2.627982072570
C	-3.268918309657	5.849099370774	-2.681076263596
H	-4.261579127236	5.390388895326	-2.857805082396
H	-2.789444777043	6.020384660792	-3.667334989962
H	-3.433381532979	6.838370383132	-2.204536567160
C	-3.747768209889	6.594207843930	2.808933594118
H	-3.350886230051	7.506547499427	2.311651106970
C	-2.785308666949	6.241687155045	3.956890499642
H	-3.103872946456	5.310277255573	4.469825908251
H	-2.751875222257	7.052569375039	4.713838306567
H	-1.755566308055	6.081347406558	3.579551894856
C	-5.149251190742	6.929863456961	3.341955539920

H	-5.587623213010	6.076202813946	3.899392412662
H	-5.844517104986	7.184604240807	2.517303773571
H	-5.109069648025	7.791110043106	4.039993721586
C	-5.247848672945	1.974420225470	1.244812373855
H	-4.713269196989	1.167103968134	0.699445628044
C	-5.336972493278	1.574342408491	2.723321802069
H	-4.336676634555	1.529095563267	3.201170102029
H	-5.814866593678	0.579675147841	2.828639131812
H	-5.952778456421	2.287675946218	3.308872017964
C	-6.657858469309	2.081591104367	0.634617655521
H	-7.224779019364	1.138340404752	0.779680579503
H	-6.608916173919	2.286350990723	-0.451670748555
H	-7.228907577021	2.903635228161	1.114508080797
C	2.697166191763	1.425141563829	1.283140628361
C	2.509250899370	0.631434955540	2.471577497178
C	3.400336903292	0.726354646241	3.549588639765
H	3.216797281777	0.123910329504	4.453301659058
C	4.495896231779	1.605203459656	3.507638310165
H	5.197713763849	1.665655039587	4.351538597047
C	4.640637434778	2.449702800542	2.393052018183
H	5.445312102133	3.202566625742	2.380857406953
C	3.757698504671	2.400765481365	1.301822972113
C	1.238905622932	-0.153687083245	2.497418487923
C	1.192560250297	-1.537574114738	2.154417022711
C	-0.067085581928	-2.181487004280	2.052925043088
H	-0.107119714304	-3.254895003437	1.813494533205
C	-1.278695940715	-1.505932888080	2.305207721419
C	-1.210945314071	-0.137595384996	2.650859064618
H	-2.143622511935	0.403450222270	2.861338243968
C	0.015344158374	0.547684622274	2.763970981336
C	2.477965263794	-2.338804346841	1.998549530999
H	3.238871872338	-1.638273449985	1.591789620186
C	2.346259922800	-3.540732162229	1.055417076122
H	3.345963420136	-3.949693020129	0.808158259759
H	1.844412813028	-3.269378063519	0.107335443206
H	1.764389340521	-4.364256782481	1.519048988693
C	2.979076538458	-2.810950361801	3.377667034478
H	3.163930528451	-1.960117696619	4.058408874059
H	3.927105593545	-3.377424544429	3.273671417139
H	2.231568950984	-3.478224821059	3.855364442777
C	-2.600910251373	-2.256813552248	2.334876967471
H	-2.418402068438	-3.248838289535	1.869479142046
C	-3.711910745726	-1.551242201585	1.547286178294
H	-4.609999498974	-2.197120236044	1.473246253923
H	-3.387542507159	-1.276885305809	0.525488710526
H	-4.020248305557	-0.615111468785	2.048595927870
C	-3.026994311708	-2.508256288849	3.793962147072
H	-2.243928866677	-3.053825180329	4.357532394061
H	-3.961306530178	-3.104916344408	3.831800073147
H	-3.216318050169	-1.549575750745	4.319842963823
C	0.052018429752	1.979040687863	3.285908304685
H	0.973617462206	2.443197859292	2.881643441142
C	-1.134606637635	2.854550055744	2.876008689882
H	-1.190681772756	2.996516995583	1.779677555755
H	-1.032171879655	3.860176795857	3.324571581727

H	-2.109631170299	2.455651557201	3.222950839655
C	0.196332686586	1.937151068314	4.820430082763
H	-0.694508072001	1.467099732168	5.287618930075
H	0.295566621214	2.964015373348	5.227770595502
H	1.090610577655	1.360071633457	5.125896768551
C	3.784821678179	3.454133102208	0.234847279256
C	3.089475576952	4.674478817310	0.465363134032
C	3.100731893298	5.669282077296	-0.530074271537
H	2.572769394447	6.619570703304	-0.348171869003
C	3.769818553634	5.497676059954	-1.751240168268
C	4.438987399209	4.279793053946	-1.968028935118
H	4.967472569443	4.125417483718	-2.920338913117
C	4.458520717857	3.257306351626	-1.002513793754
C	2.391229632921	4.949834756084	1.794317470471
H	2.291684240922	3.975261077250	2.313837843927
C	0.981549955802	5.534461807204	1.633975510249
H	0.329375724643	4.866470623897	1.039617125818
H	0.990497739549	6.526926863031	1.137678955681
H	0.506865788815	5.673017072406	2.626987948213
C	3.268503156696	5.849097517861	2.683261780217
H	4.261146869199	5.390617497156	2.860732027574
H	2.788044794104	6.019816450229	3.669136159024
H	3.433158463256	6.838647472586	2.207367718276
C	3.744660128654	6.593286316092	-2.809359947882
H	3.348829624084	7.505979340192	-2.311887802375
C	2.780095910066	6.240356329059	-3.955421794372
H	3.097434795581	5.308515566973	-4.468338762682
H	2.745632246655	7.050771360273	-4.712823570394
H	1.750953154671	6.080640041544	-3.576185910346
C	5.145296386259	6.928324293651	-3.345001534612
H	5.582550162870	6.074197030755	-3.902603825968
H	5.842015586025	7.183478643522	-2.521705273091
H	5.104094291476	7.789114437323	-4.043544243138
C	5.247036831021	1.974207407052	-1.245291057830
H	4.712440280820	1.166794991016	-0.700099909325
C	5.335835892976	1.574455758155	-2.723906664718
H	4.335431548840	1.528947344275	-3.201497472003
H	5.814012863472	0.579955365171	-2.829489865862
H	5.951269995535	2.288086360133	-3.309491970308
C	6.657233853296	2.081057509044	-0.635463593405
H	7.223923731984	1.137712059388	-0.780808829144
H	6.608673129502	2.285688223808	0.450859610407
H	7.228283003390	2.903033168645	-1.115468521740

S12. References

1. K. Izod, S. T. Liddle and W. Clegg, *Inorg. Chem.*, 2004, **43**, 214-218.
2. P. Girard, J. L. Namy and H. B. Kagan, *J. Am. Chem. Soc.*, 1980, **102**, 2693-2698.
3. B. R. Barnett, C. C. Mokhtarzadeh, P. Lummis, S. Wang, J. D. Queen, J. Gavenonis, N. Schüwer, T. D. Tilley, J. N. Boynton, N. Weidemann, D. W. Agnew, P. W. Smith, T. B. Ditri, A. E. Carpenter, J. K. Pratt, N. D. Mendelson, J. S. Figueroa and P. P. Power, in *Inorganic Syntheses*, 2018, pp. 85-122.
4. D. Parker, E. A. Suturina, I. Kuprov and N. F. Chilton, *Acc. Chem. Res.*, 2020, **53**, 1520-1534.
5. N. Muller, P. C. Lauterbur and J. Goldenson, *J. Am. Chem. Soc.*, 2002, **78**, 3557-3561.
6. Rigaku Oxford Diffraction, (2023), CrysAlisPro Software system, version 1.171.43, Rigaku Corporation, Wroclaw, Poland.; Rigaku Oxford Diffraction: 2023.
7. O. V. Dolomanov, L. J. Bourhis, R. J. Gildea, J. A. K. Howard and H. Puschmann, *J. Appl. Crystallogr.*, 2009, **42**, 339-341.
8. G. M. Sheldrick, *Acta Crystallogr. C*, 2015, **71**, 3-8.
9. G. M. Sheldrick, *Acta Crystallogr. A*, 2008, **64**, 112-122.
10. Persistence of Vision Raytracer (Version 3.6); Persistence of Vision Pty. Ltd.: 2004. https://www.povray.org.
11. GNU Image Manipulation Program (GIMP) (Version 2.10.38); The GIMP Team: 2024. https://www.gimp.org.
12. J. Liu, M. M. Bollmeyer, Y. Kim, D. Xiao, S. N. MacMillan, Q. Chen, X. Leng, S. H. Kim, L. Zhao, K. M. Lancaster and L. Deng, *J. Am. Chem. Soc.*, 2021, **143**, 10751-10759.
13. W. Clegg, A. J. Blake, J. M. Cole, J. S. O. Evans, P. Main, S. Parsons and D. J. Watkin, *Crystal Structure Analysis*, Oxford University Press, Oxford, 2nd edn., 2009.
14. B. S. Billow, B. N. Livesay, C. C. Mokhtarzadeh, J. McCracken, M. P. Shores, J. M. Boncella and A. L. Odom, *J. Am. Chem. Soc.*, 2018, **140**, 17369-17373.

15. W. T. Carnall and P. R. Fields, in *Lanthanide/Actinide Chemistry*, American Chemical Society, 1967, vol. 71, ch. 7, pp. 86-101.

16. R. Jena, F. Benner, F. Delano, D. Holmes, J. McCracken, S. Demir and A. L. Odom, *Chem. Sci.*, 2023, **14**, 4257-4264.

17. O. Kahn, *Molecular Magnetism*, VCH Publishers, Inc., New York, 1993.

18. S. T. Liddle and J. Van Slageren, *Chem. Soc. Rev.*, 2015, **44**, 6655-6669.

19. S. T. Liddle, *Angew. Chem., Int. Ed.*, 2015, **54**, 8604-8641.

20. K. R. McClain, C. A. Gould, D. A. Marchiori, H. Kwon, T. T. Nguyen, K. E. Rosenkoetter, D. Kuzmina, F. Tuna, R. D. Britt, J. R. Long and B. G. Harvey, *J. Am. Chem. Soc.*, 2022, **144**, 22193-22201.

21. C. A. Gould, K. R. McClain, J. M. Yu, T. J. Groshens, F. Furche, B. G. Harvey and J. R. Long, *J. Am. Chem. Soc.*, 2019, **141**, 12967-12973.

22. K. R. Meihaus, M. E. Fieser, J. F. Corbey, W. J. Evans and J. R. Long, *J. Am. Chem. Soc.*, 2015, **137**, 9855-9860.

23. D. M. Anderson, F. G. N. Cloke, P. A. Cox, N. Edelstein, J. C. Green, T. Pang, A. A. Sameh and G. Shalimoff, *J. Chem. Soc., Chem. Commun.*, 1989, 53-55.

24. P.-B. Jin, Q.-C. Luo, G. K. Gransbury, I. J. Vitorica-Yrezabal, T. Hajdu, I. Strashnov, E. J. L. McInnes, R. E. P. Winpenny, N. F. Chilton, D. P. Mills and Y.-Z. Zheng, *J. Am. Chem. Soc.*, 2023, **145**, 27993-28009.

25. H. M. Nicholas, M. Vonci, C. A. P. Goodwin, S. W. Loo, S. R. Murphy, D. Cassim, R. E. P. Winpenny, E. J. L. McInnes, N. F. Chilton and D. P. Mills, *Chem. Sci.*, 2019, **10**, 10493-10502.

26. J. A. Seed, L. Birnoschi, E. Lu, F. Tuna, A. J. Wooles, N. F. Chilton and S. T. Liddle, *Chem*, 2021, **7**, 1666-1680.

27. P. Dorenbos, *J. Condens. Matter Phys.*, 2003, **15**, 575-594.

28. N. F. Chilton, C. A. P. Goodwin, D. P. Mills and R. E. P. Winpenny, *Chem. Commun.*, 2015, **51**, 101-103.

29. I. L. Fedushkin, D. S. Yambulatov, A. A. Skatova, E. V. Baranov, S. Demeshko, A. S. Bogomyakov, V. I. Ovcharenko and E. M. Zueva, *Inorg. Chem.*, 2017, **56**, 9825-9833.
30. C. A. P. Goodwin, K. C. Joslin, S. J. Lockyer, A. Formanuik, G. A. Morris, F. Ortu, I. J. Vitorica-Yrezabal and D. P. Mills, *Organometallics*, 2015, **34**, 2314-2325.
31. C. A. P. Goodwin, N. F. Chilton, G. F. Vettese, E. Moreno Pineda, I. F. Crowe, J. W. Ziller, R. E. P. Winpenny, W. J. Evans and D. P. Mills, *Inorg. Chem.*, 2016, **55**, 10057-10067.
32. M. Xémard, M. Cordier, F. Molton, C. Duboc, B. Le Guennic, O. Maury, O. Cador and G. Nocton, *Inorg. Chem.*, 2019, **58**, 2872-2880.
33. F. Neese, *WIRES Comput. Mol. Sci.*, 2022, **12**, e1606.
34. V. N. Staroverov, G. E. Scuseria, J. Tao and J. P. Perdew, *J. Chem. Phys.*, 2004, **121**.
35. J. Tao, J. P. Perdew, V. N. Staroverov and G. E. Scuseria, *Phys. Rev. Lett.*, 2003, **91**, 146401.
36. S. Grimme, J. Antony, S. Ehrlich and H. Krieg, *J. Chem. Phys.*, 2010, **132**, 154104.
37. S. Grimme, *J. Comput. Chem.*, 2006, **27**, 1787-1799.
38. S. Grimme, S. Ehrlich and L. Goerigk, *J. Comput. Chem.*, 2011, **32**, 1456-1465.
39. F. Neese, F. Wennmohs, A. Hansen and U. Becker, *Chem. Phys.*, 2009, **356**, 98-109.
40. R. Izsak and F. Neese, *J. Chem. Phys.*, 2011, **135**, 144105.
41. D. A. Pantazis and F. Neese, *J. Chem. Theory. Comput.*, 2011, **7**, 677-684.
42. F. Weigend and R. Ahlrichs, *Phys. Chem. Chem. Phys.*, 2005, **7**, 3297-3305.
43. F. Weigend, *Phys. Chem. Chem. Phys.*, 2006, **8**, 1057-1065.
44. A. D. Becke, *J. Chem. Phys.*, 1993, **98**, 5648-5652.
45. T. Yanai, D. P. Tew and N. C. Handy, *Chem. Phys. Lett.*, 2004, **393**, 51-57.
46. M. Ernzerhof and G. E. Scuseria, *J. Chem. Phys.*, 1999, **110**, 5029-5036.
47. J. P. Perdew, K. Burke and M. Ernzerhof, *Phys. Rev. Lett.*, 1996, **77**, 3865-3868.
48. J. P. Perdew, M. Ernzerhof and K. Burke, *J. Chem. Phys.*, 1996, **105**, 9982-9985.
49. C. Adamo and V. Barone, *J. Chem. Phys.*, 1999, **110**, 6158-6170.
50. S. Grimme, *J. Phys. Chem. A*, 2005, **109**, 3067-3077.

51. D. A. Pantazis, X. Y. Chen, C. R. Landis and F. Neese, *J. Chem. Theory. Comput.*, 2008, **4**, 908-919.

52. J. D. Rolfes, F. Neese and D. A. Pantazis, *J. Comput. Chem.*, 2020, **41**, 1842-1849.

53. D. A. Pantazis and F. Neese, *J. Chem. Theory. Comput.*, 2009, **5**, 2229-2238.

54. T. Noro, M. Sekiya and T. Koga, *Theor. Chem. Acc.*, 2012, **131**, 1124.

55. D. Aravena, F. Neese and D. A. Pantazis, *J. Chem. Theory. Comput.*, 2016, **12**, 1148-1156.

56. A. Rajabi, R. Grotjahn, D. Rappoport and F. Furche, *Dalton Trans.*, 2024, **53**, 410-417.

57. F. Weigend and R. Ahlrichs, *Phys. Chem. Chem. Phys.*, 2005, **7**, 3297-3305.

58. D. Andrae, U. Häußermann, M. Dolg, H. Stoll and H. Preuß, *Theoret. Chim. Acta*, 1990, **77**, 123-141.

59. M. Dolg, H. Stoll, A. Savin and H. Preuss, *Theoret. Chim. Acta*, 1989, **75**, 173-194.

60. M. Dolg, H. Stoll and H. Preuss, *J. Chem. Phys.*, 1989, **90**, 1730-1734.

61. V. Barone and M. Cossi, *J. Phys. Chem. A*, 1998, **102**, 1995-2001.

62. C. Bannwarth and S. Grimme, *Comp. Theo. Chem.*, 2014, **1040-1041**, 45-53.

63. S. Grimme, *J. Chem. Phys.*, 2013, **138**, 244104.

64. G. Li Manni, I. Fdez. Galván, A. Alavi, F. Aleotti, F. Aquilante, J. Autschbach, D. Avagliano, A. Baiardi, J. J. Bao, S. Battaglia, L. Birnoschi, A. Blanco-González, S. I. Bokarev, R. Broer, R. Cacciari, P. B. Calio, R. K. Carlson, R. Carvalho Couto, L. Cerdán, L. F. Chibotaru, N. F. Chilton, J. R. Church, I. Conti, S. Coriani, J. Cuéllar-Zuquin, R. E. Daoud, N. Dattani, P. Decleva, C. de Graaf, M. G. Delcey, L. De Vico, W. Dobrautz, S. S. Dong, R. Feng, N. Ferré, M. Filatov, L. Gagliardi, M. Garavelli, L. González, Y. Guan, M. Guo, M. R. Hennefarth, M. R. Hermes, C. E. Hoyer, M. Huix-Rotllant, V. K. Jaiswal, A. Kaiser, D. S. Kaliakin, M. Khamesian, D. S. King, V. Kochetov, M. Krośnicki, A. A. Kumaar, E. D. Larsson, S. Lehtola, M.-B. Lepetit, H. Lischka, P. López Ríos, M. Lundberg, D. Ma, S. Mai, P. Marquetand, I. C. D. Merritt, F. Montorsi, M. Mörchen, A. Nenov, V. H. A. Nguyen, Y. Nishimoto, M. S. Oakley, M. Olivucci, M. Oppel, D. Padula, R. Pandharkar, Q. M. Phung, F. Plasser, G. Raggi, E. Rebolini, M. Reiher, I. Rivalta, D. Roca-Sanjuán, T. Romig, A. A. Safari, A. Sánchez-Mansilla, A. M. Sand, I. Schapiro, T. R. Scott, J. Segarra-Martí, F.

Segatta, D.-C. Sergentu, P. Sharma, R. Shepard, Y. Shu, J. K. Staab, T. P. Straatsma, L. K. Sørensen, B. N. C. Tenorio, D. G. Truhlar, L. Ungur, M. Vacher, V. Veryazov, T. A. Voß, O. Weser, D. Wu, X. Yang, D. Yarkony, C. Zhou, J. P. Zobel and R. Lindh, *J. Chem. Theory. Comput.*, 2023, **19**, 6933-6991.

65. B. O. Roos, R. Lindh, P.-Å. Malmqvist, V. Veryazov and P.-O. Widmark, *J. Phys. Chem. A*, 2004, **108**, 2851-2858.
66. B. O. Roos, V. Veryazov and P.-O. Widmark, *Theor. Chem. Acc.*, 2004, **111**, 345-351.
67. B. O. Roos, R. Lindh, P.-Å. Malmqvist, V. Veryazov and P.-O. Widmark, *J. Phys. Chem. A*, 2005, **109**, 6575-6579.
68. B. O. Roos, R. Lindh, P.-Å. Malmqvist, V. Veryazov and P.-O. Widmark, *Chem. Phys. Lett.*, 2005, **409**, 295-299.
69. M. Reiher, *Theor. Chem. Acc.*, 2006, **116**, 241-252.
70. G. Li Manni, R. K. Carlson, S. Luo, D. Ma, J. Olsen, D. G. Truhlar and L. Gagliardi, *J. Chem. Theory. Comput.*, 2016, **12**, 458-458.
71. G. Li Manni, R. K. Carlson, S. Luo, D. Ma, J. Olsen, D. G. Truhlar and L. Gagliardi, *J. Chem. Theory. Comput.*, 2014, **10**, 3669-3680.
